# *B*‑Alkyl-borabicyclo[3.3.1]nonane Reagents Promote Closed-Shell Nickel-Catalyzed Alkylarylation Toward Encoded Cyclooctene Monomers

**DOI:** 10.1021/acscentsci.5c02173

**Published:** 2026-02-20

**Authors:** Anne K. Ravn, Aimee L. Bangerter, Ethan M. Wagner, Shijia Li, Camille Z. Rubel, Steven R. Wisniewski, Peng Liu, Will. R. Gutekunst, Keary M. Engle

**Affiliations:** † Department of Chemistry, 4356 The Scripps Research Institute, 10550 N. Torrey Pines Rd., La Jolla, California 92037, United States; ⊥ School of Chemistry and Biochemistry, 1372 Georgia Institute of Technology, Atlanta, Georgia 30332, United States; § Department of Chemistry, 6614 University of Pittsburgh, 219 Parkman Ave., Pittsburgh, Pennsylvania 15260, United States; ∇ Chemical Process Development, Bristol Myers Squibb, 1 Squibb Drive, New Brunswick, New Jersey 08903, United States

## Abstract

Access to new tailored monomers is essential to explore unprecedented polymer structures and modulate their properties. In previous work, we disclosed an iteroselective diarylation of 1,5-cyclooctadiene as an attractive platform for preparing diverse cyclooctene monomers; however, it was limited to C­(sp^2^) coupling partners. Established difunctionalization methods with C­(sp^3^) fragments rely on carbon-based radical formation, which is incompatible with 1,5-cyclooctadiene. This work describes a two-electron redox manifold for the nickel-catalyzed alkylarylation of 1,5-cyclooctadiene and discloses *B*-alkyl-borabicyclo­[3.3.1]­nonane (alkyl-9-BBN) as an effective transmetalating reagent for maintaining a polar reaction mechanism. The method provides 5,6-alkylarylated cyclooctenes suitable for ring-opening metathesis polymerization to obtain new materials. The properties of these polymers are benchmarked and fine-tuned by variation of coupling partners in the nickel catalysis. Density functional theory calculations revealed that destabilization of the pretransmetalation complex promotes the reactivity of alkyl-9-BBN in transmetalation compared to alkylboronic esters.

## Graphical abstract

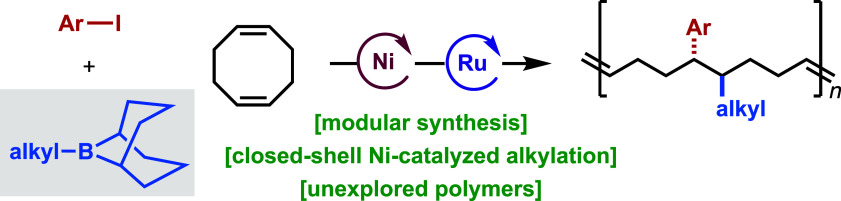

Recent advances in materials design highlight the dramatic effect of polymer side chain tailoring on materials properties.^1^ Specifically, sequence and stereocontrol are an established strategy for fine-tuning properties. As such, key performance parameters can be adjusted by modification of functional groups, tacticity, and spacing between appended groups.^2,3^ Ring-opening metathesis polymerization (ROMP) is a powerful tool for preparing well-defined polymers and tolerates various functional groups on monomer building blocks. Hillmyer and co-workers have described substituted cyclooctenes (COE) as versatile monomers in ROMP to encode sequences that are difficult to achieve through conventional homo- and copolymers of olefin monomers.^4^ However, the preparation of decorated COE building blocks typically requires multistep syntheses that are limited by available synthetic methodologies.

The transition metal-catalyzed dicarbofunctionalization of alkenes is a modular method to access new decorated molecular structures in one synthetic step from feedstock olefins.^5–11^ Directing group strategies are commonly applied to control regioselectivity in the nickel-catalyzed functionalization reactions of unactivated olefins.^12–23^ While these emerging methods are now well established tools in accessing stereodefined building blocks, their application toward preparation of monomers for polymer synthesis has been dormant until recently.^24–27^ Last year, we disclosed a nickel-catalyzed diarylation of 1,5-cyclooctadiene (COD), which furnishes decorated 5,6-diarylcyclooctene (DACOE) monomers in one step.^28^ For this reaction to occur iteratively, in which only one of the two identical carbon double bonds in COD is functionalized, the catalyst relies on transannular directivity of one of the alkenes (Figure 1A).

**Figure 1:**
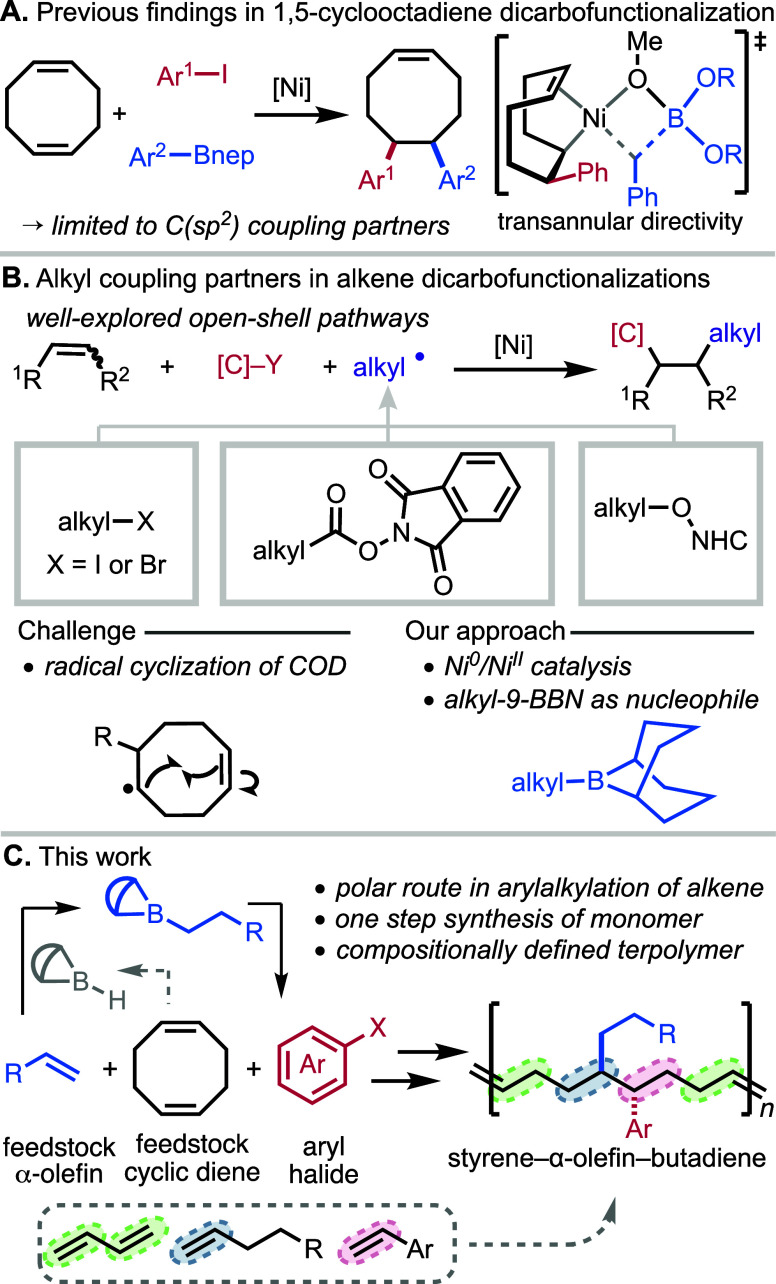
Background and synopsis of work. (A) The diarylation protocol of COD using transannular directivity to control the selectivity. (B) Literature precedent on alkylation of alkenes. (C) Our work presents a closed-shell nickel catalyzed alkylation method.

The resulting DACOE products have proven valuable as monomers in ROMP to prepare novel polymeric materials. However, the transformation was restricted to C­(sp^2^) coupling partners, substantially limiting the scope of side chains in the final polymer. Given the interesting structural space occupied by the polymers containing alkyl side chains as formal styrene−α-olefin–butadiene terpolymers, we were attracted to developing a catalytic transformation capable of accessing alkyl-substituted COE monomers. To this end, herein, we describe an alkylarylation protocol of COD that furnishes 5,6-alkylarylated cyclooctene (AACOE) monomers for ROMP to provide compositionally defined polymeric materials bearing alkyl side chains. The reaction is enabled by the discovery of *B*-alkyl-borabicyclo­[3.3.1]­nonane (alkyl-9-BBN) as a unique and facile nucleophilic coupling partner in closed-shell alkene dicarbofunctionalization that bypasses complications that arise from radical-based approaches (Figure 1C).

Recent advances in olefin dicarbofunctionalization with alkyl coupling partners have commonly relied on carbon-centered radicals generated from alkyl halides, redox-active esters, or NHC-activated alcohols (Figure 1B).^29–31^ This open-shell redox manifold takes advantage of the facile reductive elimination from Ni­(III) to construct the new C­(sp^3^)–C­(sp^3^) bond.^32^ However, under radical conditions, COD undergoes undesired ring-closure, which halts the desired catalytic difunctionalization toward COE synthesis.^33,34^ Thus, dicarbofunctionalization of COD with an alkyl coupling partner represents an interesting proving ground for identification of alkyl coupling partners predisposed toward polar two-electron redox manifolds.

To this end, the model three-component coupling of iodobenzene **1a**, COD **2a**, and alkylboron reagents was investigated. In pilot studies, we found that direct adaptation of conditions from our prior 1,2-diarylation method with an alkyl-Bnep ester was completely ineffective, pointing to potential problems in the transmetalation step coupling partners (Figure 2A). To overcome this issue, we tested other alkylboronic acid or ester coupling partners, which were likewise ineffective (Figure 2A). Control experiments with other alkyl organometallic nucleophiles precedented in olefin dicarbofuntionalization^35–37^ (Grignard and organozinc reagents) were likewise ineffective. Our attention then turned to *B*-alkyl-borabicyclo­[3.3.1]­nonane (alkyl-9-BBN) reagents, which are well established alkyl nucleophiles in Suzuki–Miyaura reactions.^38^ Alkyl-9-BBN reagents were first utilized in palladium-catalyzed C­(sp^2^)–C­(sp^3^) cross-couplings by Suzuki and co-workers,^39,40^ and later mechanistically studied by the Soderquist group and others.^41–43^ In nickel-catalysis, the Fu group has published comprehensive methodologies for application of alkyl-9-BBN in cross-couplings between alkyl–alkyl fragments.^44–49^ Although these investigations disclose broad utilization of the alkyl-9-BBN reagent in two-component reactions, it has not been studied in three-component conjunctive couplings. Given the reagent’s ability to proceed by a polar, two-electron redox manifold, its high Lewis acidity compared to other alkyl–boron nucleophiles, its ease to synthesize, and its general stability in solution, alkyl-9-BBN reagents are an intriguing and promising alkyl coupling partner.

**Figure 2:**
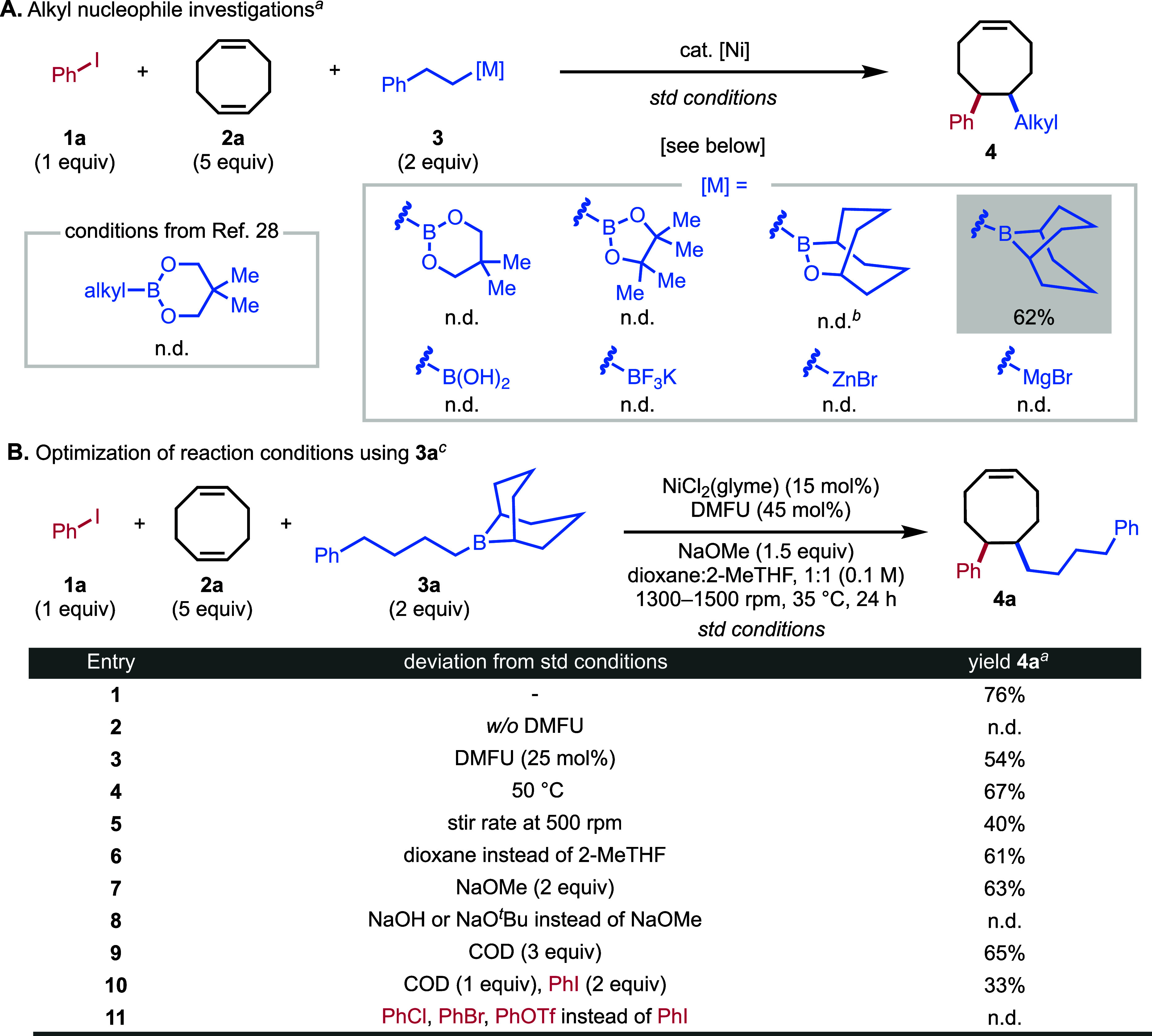
Optimization of the reaction. (A) Initial investigation of alkyl nucleophiles. (B) Optimization of reaction conditions using alkyl-9-BBN reagent **3a**. ^a^Reactions were performed under standard conditions with phenethyl-nucleophiles. ^b^Reaction performed with a 4-phenylbutyl substituent instead of phenethyl. ^c^Reaction performed on a 0.1 mmol scale. Yield based on ^1^H NMR analysis of crude reaction mixtures using 1,3,5-trimethoxybenzene as an internal standard.

Indeed, the use of 4-phenylbutyl-9-BBN (**3a**) led to an immediate increase in yield, and extensive optimization of reaction conditions eventually allowed us to arrive at a protocol that provided 76% yield using NiCl_2_(glyme) as precatalyst, dimethyl fumarate (DMFU) as ligand, and NaOMe as base in a 1:1 solvent mixture of 1,4-dioxane and 2-MeTHF (0.1 M) (Figure 2B, entry 1). The reaction yield was optimal at 35 °C and a stirring rate above 1300 rpm, pointing to the heterogeneous nature of the reaction and the importance of mass transfer effects. Full conversion of **1a** is obtained under optimized reaction conditions with the competing two-component product between **1a** and **3a** being the major side product. Notably, the tetrafunctionalization of **2a** is not observed. Extensive ligand screening did not improve the product yield (see SI Figures S1–S3). Generally, fumarate-derived ligands with steric and electronic profiles similar to those of DMFU performed comparably, whereas no reactivity was observed with other ligand classes. In the absence of DMFU, the desired product is not detected, and the predominant byproduct was from the two-component reaction (Figure 2B, entry 2). Decreasing the ligand loading to 25 mol % also decreased the yield to 54% (Figure 2B, entry 3). The addition of an electron-deficient olefin ligand, such as DMFU, has previously been showed to promote reductive elimination from a nickel­(II) center and suppress β-hydride elimination.^15,28,50,51^ The product yield decreased with higher reaction temperature, lower stir rate, without 2-MeTHF as cosolvent, or with an increased NaOMe amount (Figure 2B, entries 4–7). The reaction is sensitive to switching the base, and neither NaO^*t*^Bu nor NaOH provide detectable product formation by ^1^H NMR analysis (Figure 2B, entry 8). Less equivalences of COD also decreased the yield of **4a** (Figure 2B, entries 9 and 10). No desired product is obtained when using aryl chloride, bromide, or triflate instead of aryl iodide (Figure 2B, entry 11).

Having optimized the reaction conditions, we turned to investigating the substrate scope (Figure 3). The standard substrate **4a** was isolated in 73% yield. Generally, aryl iodides bearing electron-donating groups, such as **4b** and **4c**, provided higher yields than those having electron-withdrawing substituents, such as **4d**, **4e**, and **4f**. The reaction is sensitive to sterically hindered substrates (see SI Table S5), but it provided a moderate 38% isolated yield of the 1-naphthyl substituted product **4g**. Full conversion of aryl iodide is observed regardless of the product yield with the two-component coupling being the major side product. The reaction tolerates amines, affording the indole **4h** and morpholine **4i** substrates in 47% and 64% yields, respectively. Additionally, **4j** containing a methyl ester was obtained in a 46% yield. Next, we investigated the scope of alkyl coupling partner. Styrene derived phenethyl-9-BBN provided **4k** in 67% yield, and cyclohexylethyl substrate **4l** was obtained in 72% yield. Substrates containing long alkyl chains are well tolerated, giving **4m** in 52% yield and the perfluorinated substrate **4n** in 66% yield. Allyl derived alkyl-9-BBN reagents with silane or dimethoxybenzene afforded **4o** or **4p**, respectively, in good yields. Similarly, an ester in **4q** and a silane protected alcohol in **4r** were tolerated, obtaining 45% and 54% yield, respectively. Various amines were tolerated under the reaction conditions, such as Boc-protected piperidine **4s** in 56% yield, methyl sulfonamide **4t** in 63% yield, aniline **4u** in 37% yield, and phthalimide derived **4v** in 53% yield. Finally, we obtained the phenyl methyl substituted COE **4x** by utilizing a Grignard addition of MeMgBr to MeO-9-BBN. This addition provides the desired Me-9-BBN­(OMe) borate reagent, which is then added to the Ni-catalyzed reaction. The reaction could be scaled up to obtain bulk material for polymerization studies. The substrates **4a**, **4i**, **4n′**, **4s′**, and **4x** were all prepared on a larger scale (2.0 mmol), providing the desired monomers in yields similar to those of reactions carried out on 0.2 mmol scale.

**Figure 3:**
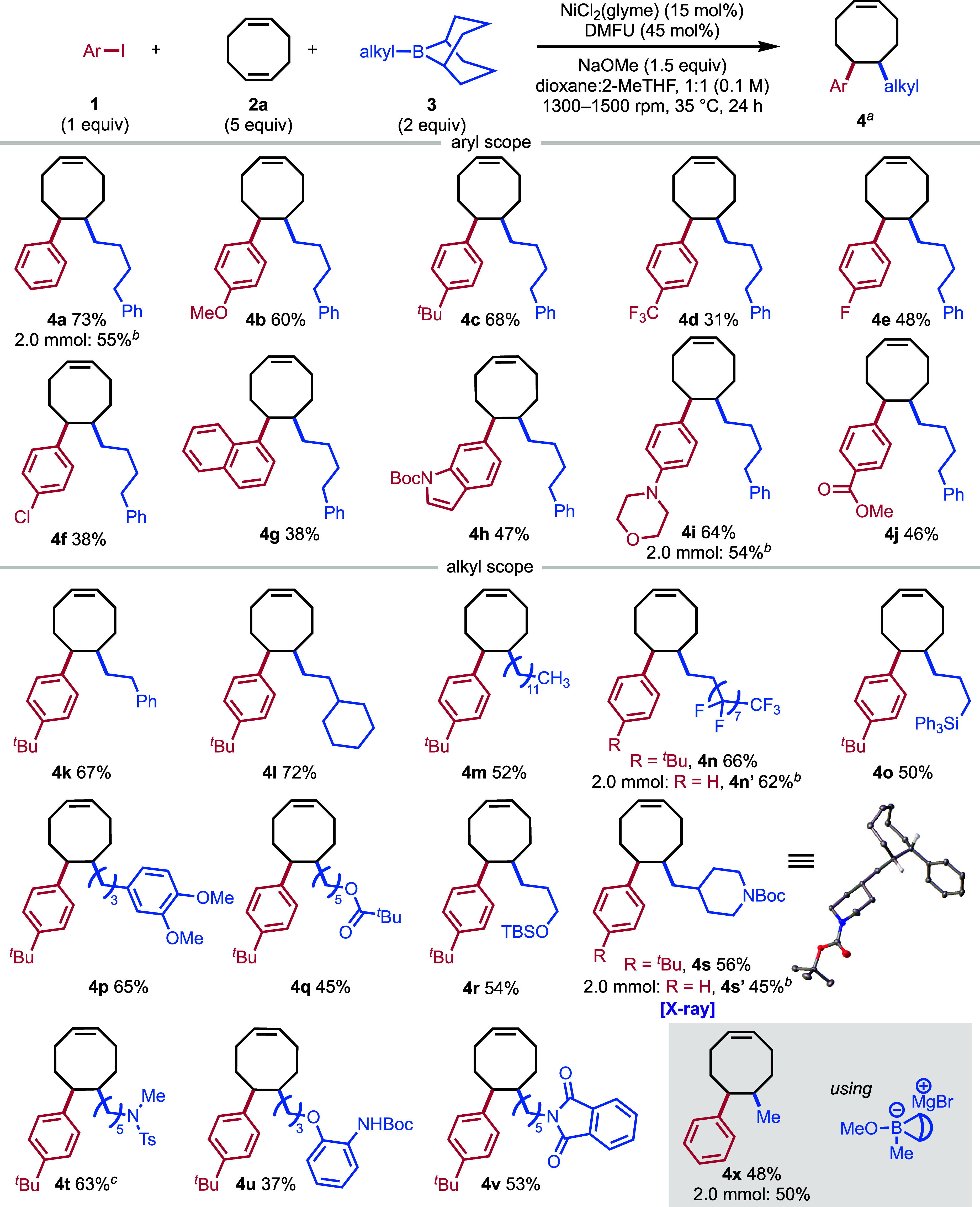
Scope of the nickel-catalyzed alkylarylation of 1,5-cyclooctadiene. The products are formed as a single diastereomer and isolated as a racemic mixture. ^a^Reaction performed on 0.2 mmol scale and percentages represent isolated yields (see SI for details). ^b^Reaction performed on 2.0 mmol scale and percentages represent the corrected yield of product in the mixture with the two-component coupling product yields (see SI for details). ^c^Reaction performed via Ni­(COD)_2_ instead of NiCl_2_(glyme).

To gain insight into the reaction mechanisms of the Ni-catalyzed COD alkylarylation and the unique roles of alkyl-9-BBN in promoting the three-component reaction, we turned to DFT calculations (Figure 4).^52,53^ We computed the reaction energy profiles of the reactions of COD (**2a**) and iodobenzene (**1a**) with ^*n*^Bu-9-BBN (**3a′**) and alkylboronic ester ^*n*^Bu-Bnep (**3b**) at the M06/6-311+G­(d,p)–SDD/SMD­(1,4-dioxane)//B3LYP-D3/6-31G­(d)–SDD level of theory (see SI Figure S11). Oxidative addition with iodobenzene (**1a**) and migratory insertion of COD into the Ni–Ph bond lead to alkylnickel­(II) iodide (**6**) with the transannular alkene coordinated to the T-shaped Ni center. Ligand exchange of **6** with borate **7a** derived from the reaction of ^*n*^Bu-9-BBN (**3a′**) with the base (NaOMe)^43^ replaces the iodide anion to form pretransmetalation complex **8a** with a μ^2^-OMe ligand bridging the Ni and B centers (Figure 4).^42^ In **8a**, a weak agostic interaction^54^ with a C–H bond on 9-BBN blocks the remaining binding site on the Ni center. Subsequent transmetalation occurs via a four-membered cyclic transition state (**TS1a**). This process is extremely facile with a low activation barrier of 6.1 kcal/mol with respect to pretransmetalation complex **8a** and 8.4 kcal/mol with respect to 5-cyclooctenylnickel­(II) iodide (**6**). After the formation of dialkylnickel­(II) intermediate **9a**, the DMFU ligand promotes C­(sp^3^)–C­(sp^3^) reductive elimination^50^ to form the AACOE product **4a′** (see Figure S11).

**Figure 4:**
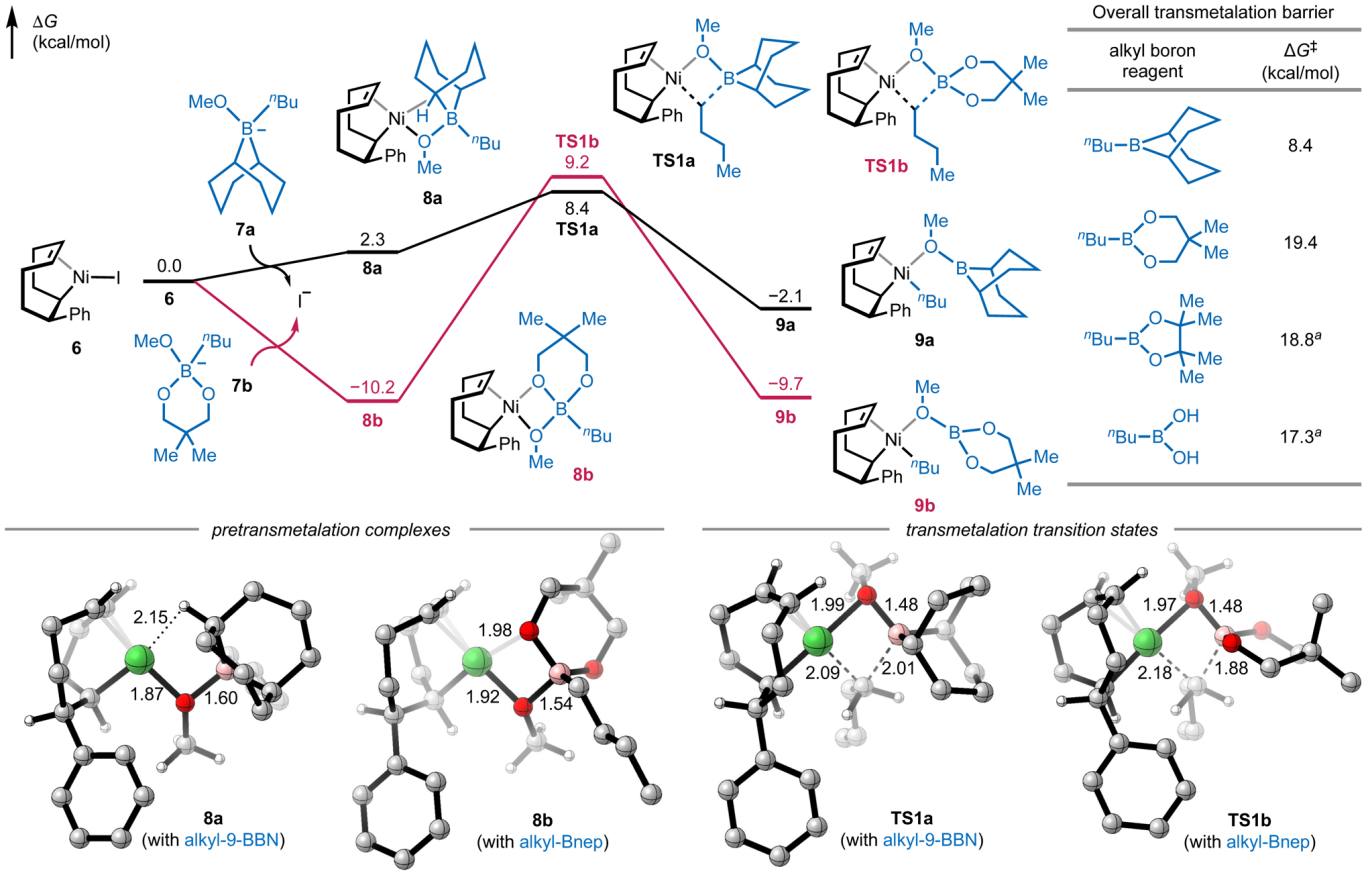
Computed reaction energy profiles of transmetalation with alkyl-9-BBN (black) and alkyl-Bnep (red). Distances are in Å. ^a^See SI Figures S11–S14 for details.

We next focused on understanding the reactivity difference between alkyl-9-BBN and the various unsuccessful alkylboronic esters in the key transmetalation step. In contrast to the pretransmetalation complex with alkyl-9-BBN, the transmetalation with borate **7b** derived from boronic ester ^*n*^Bu-Bnep forms a much more stable pretransmetalation complex **8b** (10.2 kcal/mol more stable than **6**) in which the OMe and one of the neopentylglycol ester oxygen atoms both coordinate to the Ni center to form a square planar complex. Transmetalation from **8b** requires the dissociation of the Bnep oxygen from Ni, whereas the μ^2^-OMe ligand remains bridging the Ni and B centers in the four-membered cyclic transition state (**TS1b**). This ground-state stabilization effect significantly increases the overall barrier to transmetalation with ^*n*^Bu-Bnep to 19.4 kcal/mol, compared with an overall barrier of 8.4 kcal/mol for the transmetalation with ^*n*^Bu-9-BBN. The alkyl transmetalation barrier with ^*n*^Bu-Bnep is significantly higher than the *aryl* transmetalation with Ph-Bnep (Δ*G* ^‡^ = 10.5 kcal/mol) from our previous study.^28^ Our calculations with other alkylboronic ester reagents lead to the same trend that the bidentate coordination of these borates leads to more stable pretransmetalation complexes and higher overall barriers to transmetalation (see Figure S13). In previous studies, the reactivity of alkyl-9-BBN is often attributed to its Lewis acidity.^43^ Here, our DFT calculations reveal that ground-state destabilization of the pretransmetalation complex also contributes to the faster rate of transmetalation with alkyl-9-BBN.

Having established a versatile nickel-catalyzed method to access diverse AACOEs, we next assessed their viability in ROMP and characterized the resulting polymers. Our previous studies have revealed DACOEs as competent monomers for ROMP to give compositionally defined materials.^28^ The DACOEs were found to polymerize at much slower rates compared to existing COE derivatives in the literature due to unfavorable 1,3-diaxial strain in the active boat–chair conformation during metathesis. On the other hand, this also led to greater control over the polymerization process in which polymer molecular weight could be readily targeted by varying the monomer to initiator ratio. To understand the impact of different alkyl substituents on the monomers in ROMP and on the resulting polymer, five AACOEs were chosen for polymerization based on the substituent size (long chain alkyl **4a**, methyl **4x**, and Boc-piperidyl **4s′**), Lewis basic groups (morpholino **4i**), and fluorophilicity (fluorinated **4n′**). To more readily compare to the DACOE monomers, diphenyl monomer **5a** was included in the study.

Using the standard reaction conditions developed for ROMP of DACOEs (1 M in DCM, 100:1 monomer:G3 ratio, room temperature), all AACOE monomers examined were found to polymerize to high conversion (>96%) after 2 h. These materials represent encoded terpolymers derived from styrene, α-olefin, and butadiene monomers. The terpolymers lack long-range regioregularity with respect to the styrene and α-olefin subunits but maintain a perfectly stoichiometric composition and regular distribution of subunits that would be difficult to obtain through direct polymerization.^55^ While slightly larger quantities of cyclic oligomers were observed in the AACOEs relative to **5a**, these oligomers were readily removed upon precipitation into methanol. The number-average molecular weights (*M*_n_’s) of the precipitated polymers ranged from 20 to 46 kg/mol and were in general agreement with monomer to initiator ratios (Figure 5A). The obtained polymers exhibited dispersities (*Đ*’s) ranging from 1.5 to 2.2 and monomodal elution peaks by SEC, consistent with chain transfer reactions occurring on the unhindered backbone alkenes during polymerization.

**Figure 5:**
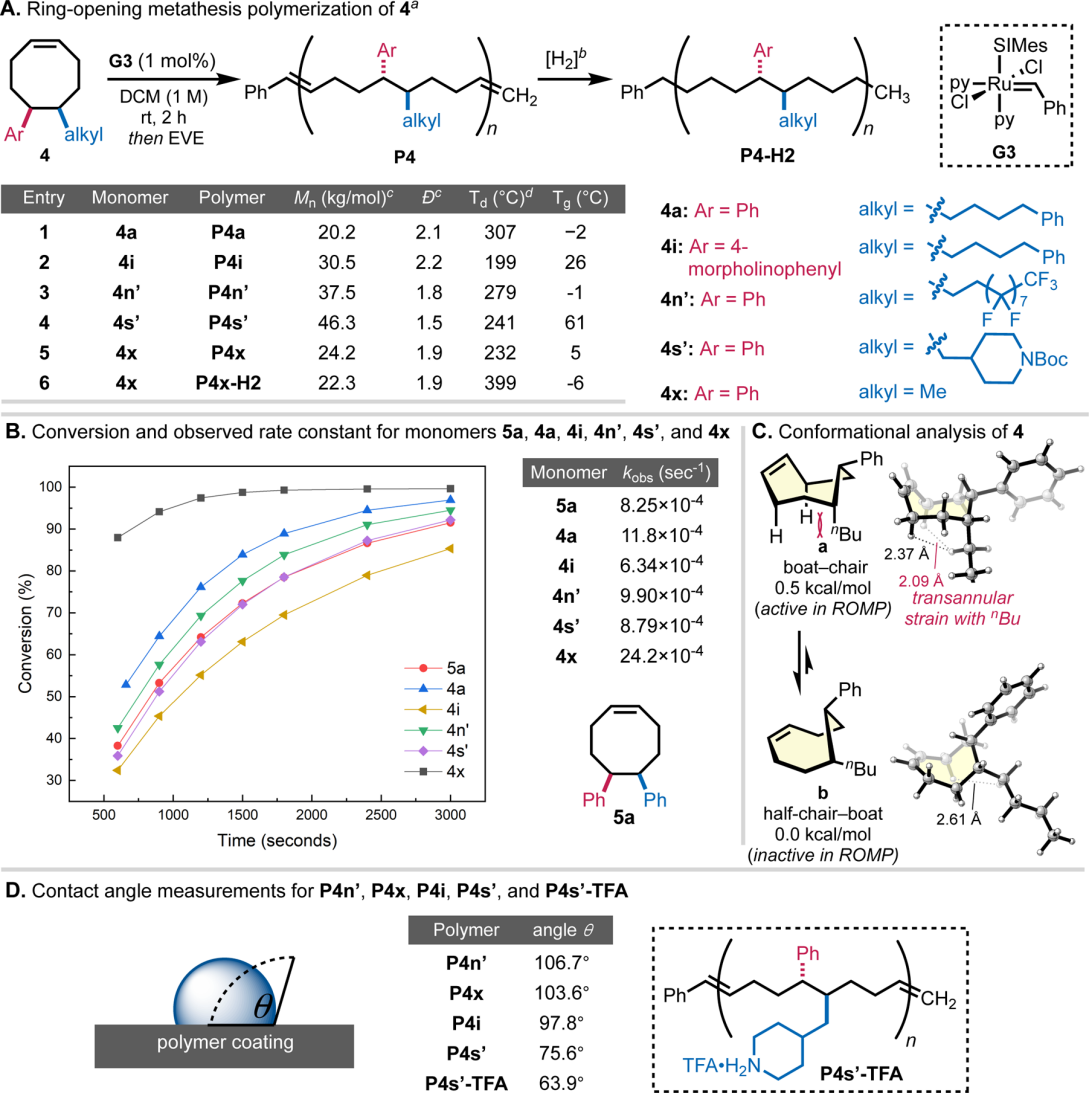
Polymerization of alkylarylated cyclooctenes. (A) Ring-opening metathesis polymerization for the 5,6-alkylarylated cyclooctenes **4a**, **4i**, **4n′**, **4s′**, and **4x**. (B) Conversion of monomers and observed rate of polymerization compared to diarylated cyclooctene **5a**. (C) Conformational analysis of alkylarylated cyclooctene. (D) Contact angle measurement for polymers **P4n′**, **P4x**, **P4i**, **P4s′**, and **P4s′-TFA**. ^a^Relative stereochemistry is shown that does not imply long-range tacticity. ^b^Backbone reduction using diimide (see SI for details). ^c^Number-average molecular weights (*M*_n_’s) and dispersities (*Đ*’s) of purified polymer products were determined by SEC using polystyrene standards. ^d^Temperature at 5% mass loss.

To evaluate the changes in polymerization rate, kinetic studies were conducted by monitoring monomer conversion over a 50 min period via in situ ^1^H NMR spectroscopy (Figure 5B). All of the polymerizations displayed the expected first-order reaction kinetics with *R* ^2^ values greater than 0.99 (see SI Figure S7), and a wide range of observed rate constants (*k*_obs_) were measured across the AACOE series (Figure 5B). Rates of polymerization were found to generally correlate with the size of the alkyl substituent with methylated **4x** polymerizing twice as fast as the long chain alkyl **4a**. The AACOE monomers displayed faster rate constants than the diphenyl **5a** reference with the exception of **4s′** and **4i**. Monomer **4s′** with a large piperidyl group polymerized at nearly the same rate as **5a**, suggesting a similar steric environment to a phenyl ring. Monomer **4i** contains a morpholine-substituted aryl ring, and the reduced rate of polymerization could be due to coordination of the nitrogen or oxygen atoms to the propagating ruthenium center. To gain further insight into the influence of transannular interactions on the polymerization of the AACOEs, a conformational analysis of 5-*n*-butyl-6-phenylcyclooctene was performed using density functional theory (DFT) calculations (Figure 5C). The boat–chair conformer (**a**), which has a more exposed C=C bond and is catalytically active in ROMP, is 0.5 kcal/mol higher in energy than the catalytically inactive half-chair–boat conformer (**b**) with a sterically shielded C=C bond. In **a**, the alkyl substituent is in a pseudoaxial position, introducing transannular strain within the eight-membered ring. These steric interactions with the relatively remote alkyl substituent are expected to affect the equilibrium of the active and inactive monomer conformations and influence the transannular strain in the propagation transition states with conformer **a**. This is consistent with the experimental observation that AACOEs with less hindered alkyl substituents, such as **4x**, have overall faster rates of ROMP (Figure 5B).

The thermal properties of the AACOE polymers were found to dramatically change based on the nature of the alkyl substituent. Glass transition temperatures (*T*_g_’s) measured by differential scanning calorimetry (DSC) ranged from −2 °C for the long chain alkyl **P4a** to 61 °C for Boc-piperidine **P4s′**. Since *T*_g_ is related to segmental motion of the polymer chains, this suggests that the bulky piperidine ring inhibits rotation around the C–C bond between the aryl and alkyl substituent in a manner similar to that of the DACOE series (*T*_g_ of **5a** = 59 °C). Overall thermal stability of the AACOE polymers could be significantly enhanced through hydrogenation with conversion of **P4x** to **P4x-H**_**2**_ leading to a change in onset of degradation (5% mass loss) from 232 to 399 °C. To further demonstrate the ability of these materials to generate tailorable polymer properties, contact angle measurements were performed on polymer films prepared from **P4i**, **P4n′**, **P4s′**, and **P4x** (Figure 5D). The perfluorinated **P4n′** was found to be the most hydrophobic material with a contact angle of 106.7°, and the piperidine-containing **P4s′** was the least hydrophobic with a contact angle of 75.6°. The hydrophilicity of **P4s′** could readily be increased upon Boc deprotection with TFA, **P4s′-TFA**, which further decreases the contact angle to 63.9°. Collectively, these studies highlight the tailorable structure–property relationships of the AACOE materials with further application possibilities enabled through polymer postfunctionalization.

In conclusion, this work demonstrates alkyl-9-BBN reagents as effective nucleophiles to promote the closed-shell nickel-catalyzed alkylarylation of 1,5-cyclooctadiene. The method tolerates a wide variety of functional groups and various alkyl chain lengths. ROMP of the resulting AACOE monomers generates previously unexplored materials. The properties of these polymers were found to be significantly influenced by the nature of the encoded side chain size and chemistry. Specifically, hydrophobicity analyses show great promise for fine-tuning the material properties. DFT calculations for nickel catalysis highlight that destabilization of the ground state in pretransmetalation facilitates the observed reactivity for alkyl-9-BBN in comparison to the inefficient alkylboronic esters. We expect that these insights into the alkyl-9-BBN nucleophile will have broad application in three-component coupling reactions.

## Supplementary Material







## Notes

Experimental procedures, characterization data for all new compounds, and details for DFT calculations (PDF)
Crystallographic data for **4s′** (CIF)
Transparent Peer Review report available (PDF)

## Abbreviations

9-BBN: 9-borabicyclo­[3.3.1]­nonane
COD: 1,5-cyclooctadiene
COE: cyclooctene
AACOE: 5,6-alkylarylated cyclooctene
DACOE: 5,6-diarylated cyclooctene, ROMP, ring-opening metathesis polymerization
SEC: size-exclusion chromatography
DSC: differential scanning calorimetryl
DFT: density functional theory
