# Supplementary material for: B‑Alkyl-borabicyclo[3.3.1]nonane Reagents Promote Closed-Shell Nickel-Catalyzed Alkylarylation Toward Encoded Cyclooctene Monomers
Source: ACS Cent Sci. 2026 Feb 20;12(3):324–32. doi: 10.1021/acscentsci.5c02173 (PMC13022722; doi:10.1021/acscentsci.5c02173)

Supporting Information

B-Alkyl-borabicyclo[3.3.1]nonane Reagents Promote Closed-Shell Nickel-Catalyzed Alkylarylation towards Encoded Cyclooctene Monomers

Anne K. Ravn[†], Aimee L. Bangerter[†‡], Ethan M. Wagner[⊥‡], Shijia Li[§‡], Camille Z. Rubel[†], Steven R. Wisniewski[∇], Peng Liu [§*], Will. R. Gutekunst[⊥*], Keary M. Engle[†*]

[†]Department of Chemistry, The Scripps Research Institute, 10550 N. Torrey Pines Rd., La Jolla, CA 92037, USA

[⊥]School of Chemistry and Biochemistry, Georgia Institute of Technology, Atlanta, GA 30332, USA

[§]Department of Chemistry, University of Pittsburgh,219 Parkman Ave, Pittsburgh, PA 15260, USA

[∇]Chemical Process Development, Bristol Myers Squibb, 1 Squibb Drive, New Brunswick, NJ 08903, USA

[‡]Authors contributed equally

Table of Contents

General Information for the Nickel-Catalyzed Arylalkylation of 1,5-Cyclooctadiene S3

Reaction Optimization Data for the Nickel Catalyzed Arylalkylation of 1,5-Cyclooctadiene S4

General Procedure 1: Preparation of alkyl-9-borabicyclo[3.3.1]nonane solutions S9

Characterization of alkyl–9-BBN solutions in 1,4-dioxane S9

General Procedure 2: Nickel-Catalyzed Arylalkylation of 1,5-Cyclooctadiene S12

General Procedure 3: Scale Up Procedure for Nickel-Catalyzed Arylalkylation of 1,5-Cyclooctadiene S12

Characterization of 5,6-Arylalkylated Cyclooctenes (4) S13

General Information for the Ring-Opening Metathesis Polymerization (ROMP) of the 5,6-Arylalkylated Cyclooctenes (AACOE) S23

General Procedure 4: ROMP of (Z)-5,6-Arylalkylated Cyclooctenes (4) S24

Characterization of Polymers S24

Kinetic Studies for the ROMP of AACOE S29

Thermal Properties of the Polymers P4 .... S30

Contact Angle Measurements of Polymers S31

Computational Details S32

Reaction energy profiles of the Ni-catalyzed alkylarylation of COD S33

Conformations of 5-n-butyl-6-phenylcyclooctene monomer S33

DFT studies of the reactivity of different boryl reagents in transmetalation S35

Cartesian coordinates (Å) and energies of optimized structures S36

X-Ray Crystallography of 4s’ S80

References S85

NMR Spectra S87

General Information for the Nickel-Catalyzed Arylalkylation of 1,5-Cyclooctadiene

Unless stated otherwise, all materials were received new from commercial sources and were used without further purification. All materials previously opened were filtered through a silica plug and sparged with nitrogen prior to use. All glassware and stirring bars were dried in an oven at 100 °C overnight prior to use unless otherwise stated. Solvents (1,4-dioxane and 2-methyl-tetrahydrofuran) were purchased from MilliporeSigma in 100-mL Sure/Seal bottles and used as received. $NiCl_2$(glyme) was stored in a nitrogen-filled glovebox, and $Ni(COD)_2$ was kept in the glovebox freezer (-35 °C). The 9-borabicyclo[3.3.1]nonane dimer was purchased from commercial sources and stored in a nitrogen-filled glovebox. The following compounds were synthesized according to literature procedures, 2-(pent-4-en-1-yl)isoindoline-1,3-dione[1], *N*,4-dimethyl-*N*-(pent-4-en-1-yl)benzenesulfonamide[2], pent-4-en-1-yl pivalate[3], (allyloxy)(*tert*-butyl)dimethylsilane[4] and (*1Z,5Z*)-1-methylcycloocta-1,5-diene and (*1Z,5Z*)-1-phenylcycloocta-1,5-diene[5]. All alkoxide bases were stored under inert atmosphere to ensure high purity and avoid decomposition.[6] The 1-dram reaction vials, 40-mL vials, and caps with TFE septa were purchased from ChemGlass (Cat#: CG-4904-05). Ambient (room) temperature refers to 21–24 °C. Elevated temperatures were maintained by an IKA heating block for 1-dram vials or a silicon oil bath for larger vessels. Thin-layer chromatography (TLC) was performed using EMD Millipore 250 mm silica gel F-254 plates (250 µm) with F-254 fluorescent indicator and visualized by UV fluorescence quenching, iodine, Seebach's stain, or potassium permanganate stain. Preparative thin-layer chromatography (PTLC) plates with 1,000-micron- or 500-micron-sized particles were acquired from MilliporeSigma. Flash column chromatography was performed using silica gel with 40–63-micron-sized particles from SILICYCLE. ^{1}H, ^{13}C, and ^{19}F NMR spectra were recorded on Bruker AVIII 400 MHz, Bruker AV NEO 500 MHz, Bruker AV NEO 600, Bruker AV NEO 399 MHz, or JEOL JNM-ECZ400R 400 MHz NMR instruments. The ^{1}H and ^{13}C spectra were reported relative to residual solvent signals according to the literature.[7] At room temperature significant broadening of the peaks in the ^{13}C NMR spectra was observed for indicated compounds leading to poorly resolved signals in the spectra. This observation has previously been reported for similar products.[8] The following abbreviations, or combinations thereof, are used to describe multiplicities: b = broad, s = singlet, d = doublet, t = triplet, q = quartet, and m = multiplet. High-resolution mass spectra (HRMS) were recorded on an Agilent LC/MSD TOF mass spectrometer by electrospray ionization (positive mode) time of flight experiments. In select cases, HRMS data could not be collected for products due to their inability to be ionized via typical ionization methods. In these instances, gas chromatography mass spectrometry (GCMS) data is provided. GCMS data were recorded on Agilent 7890A GC System using an Agilent 5975C GC/MSD or an Agilent 8890 GC System coupled to an Agilent 5973 MSD.

Reaction Optimization Data for the Nickel Catalyzed Arylalkylation of 1,5-Cyclooctadiene

Table S1. Ligand Screen for the Nickel-Catalyzed Arylalkylation of 1,5-Cyclooctadiene

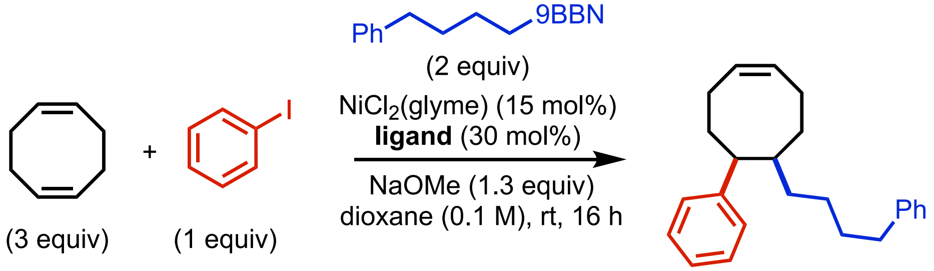

Entry	ligand	^{1}H NMR yield	RM PhI
1	DMFU	56%	7%
2	DMFU (45 mol%)	61%	trace
3	dimethylmaleate	30%	12%
4	di-*tert*-butyl fumarate	58%	18%
5	fumaronitrile	n.d.	51%
6	**L1**	n.d.	51%
7	*trans*-stilbene	n.d.	88%
8	phenylethylene sulfonyl fluoride	trace	47%
9	4-trifluoromethyl styrene	n.d.	67%
10	**L2**	n.d.	25%
11	duroquinone	n.d.	82%
12	BQ^{t}Bu	n.d.	96%
13	TO^{CF3}	n.d.	93%
14	**L3**	n.d.	88%
15	**L4**	n.d.	67%

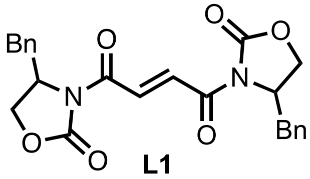

L1

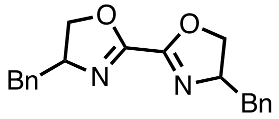

L2

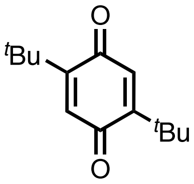

BQ^{tBu}

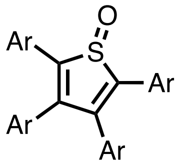

Ar=4-trifluoromethylphenyl
TO^{CF3}

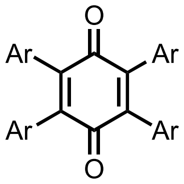

L3, Ar=4-methoxyphenyl
L4, Ar=4-trifluoromethylphenyl

Reaction conditions: PhI (0.1 mmol), alkyl–9-BBN (0.2 mmol in 0.5 mL 1,4-dioxane), NaOMe (0.13 mmol), 1,5-cyclooctadiene (0.3 mmol), $NiCl_2$(glyme) (0.015 mmol), ligand (0.030 mmol), 1,4-dioxane (0.5 mL). Yields are determined by ^{1}H NMR of the crude reaction mixture using CH_2Br_2 as internal standard.

Table S2. Ligand Screen for the Nickel-Catalyzed Arylalkylation of 1,5-Cyclooctadiene

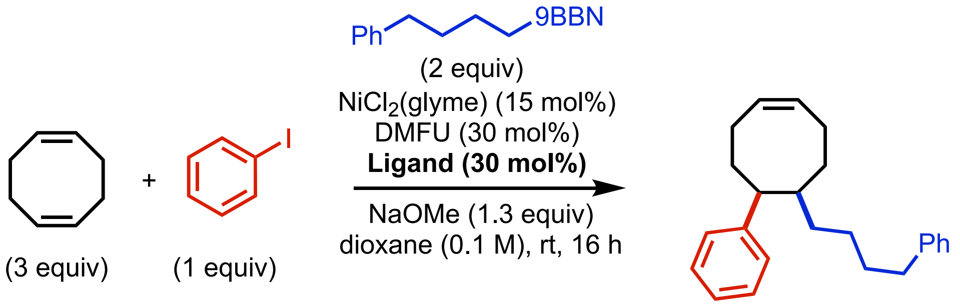

**Entry**	**Ligand** (15 or 30 mol%)	% Yield
**1**	*N*,*N'*-dimethylcyclohexane-1,2-diamine	n.d.
**2**	*N*,*N'*-dimethylethane-1,2-diamine	n.d.
**3**	4,7-diphenyl-1,10-phenanthroline	n.d.

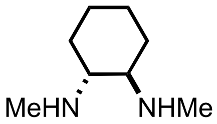

N,*N'*-dimethylcyclohexane-1,2-diamine

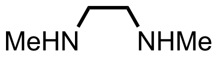

N,*N'*-dimethylethane-1,2-diamine

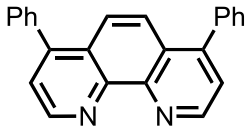

4,7-diphenyl-1,10-phenanthroline

Reaction conditions: PhI (0.1 mmol), alkyl–9-BBN (0.2 mmol in 0.5 mL 1,4-dioxane), NaOMe (0.13 mmol), 1,5-cyclooctadiene (0.3 mmol), $NiCl_2$(glyme) (0.015 mmol), DMFU (0.030 mmol), ligand (0.030 mmol), 1,4-dioxane (0.5 mL). Yields are determined by ^{1}H NMR of the crude reaction mixture using CH_2Br_2 as internal standard.

Table S3. Ligand Screen for the Nickel-Catalyzed Arylalkylation of 1,5-Cyclooctadiene

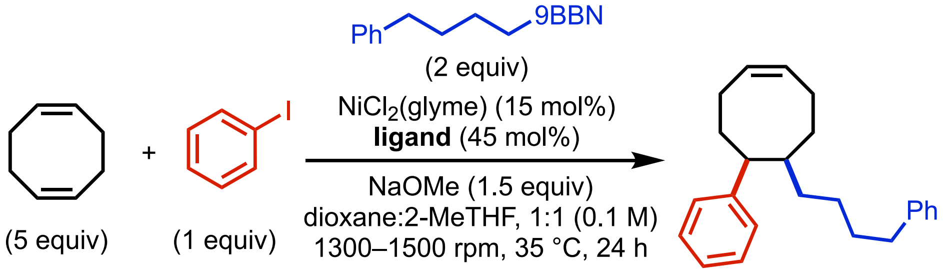

entry	ligand	^{1}H NMR yield
1	none	0%
2	dimethyl fumarate (DMFU)	75%
3	di-*tert*-butyl fumarate	35%
4	bis(1,1,1,3,3,3-hexafluoropropan-2-yl)-fumarate	25%
5	bis(2,6-diethylphenyl) fumarate	64%
6	fumaronitrile	22%
7	dimethyl cyclobutene dicarboxylate	3%
8	dimethyl 2,3-diphenyl maleate	0%
9	*tert*-butyl methyl fumarate	62%
10	diethyl fumarate	65%

Reaction conditions: PhI (0.1 mmol), alkyl–9-BBN (0.2 mmol in 0.5 mL 2-MeTHF), NaOMe (0.15 mmol), 1,5-cyclooctadiene (0.5 mmol), $NiCl_2$(glyme) (0.015 mmol), ligand (0.045 mmol), 1,4-dioxane (0.5 mL). Yields are determined by ^{1}H NMR of the crude reaction mixture using CH_2Br_2 as internal standard.

Table S4. Unsuccessful Olefin Substrates

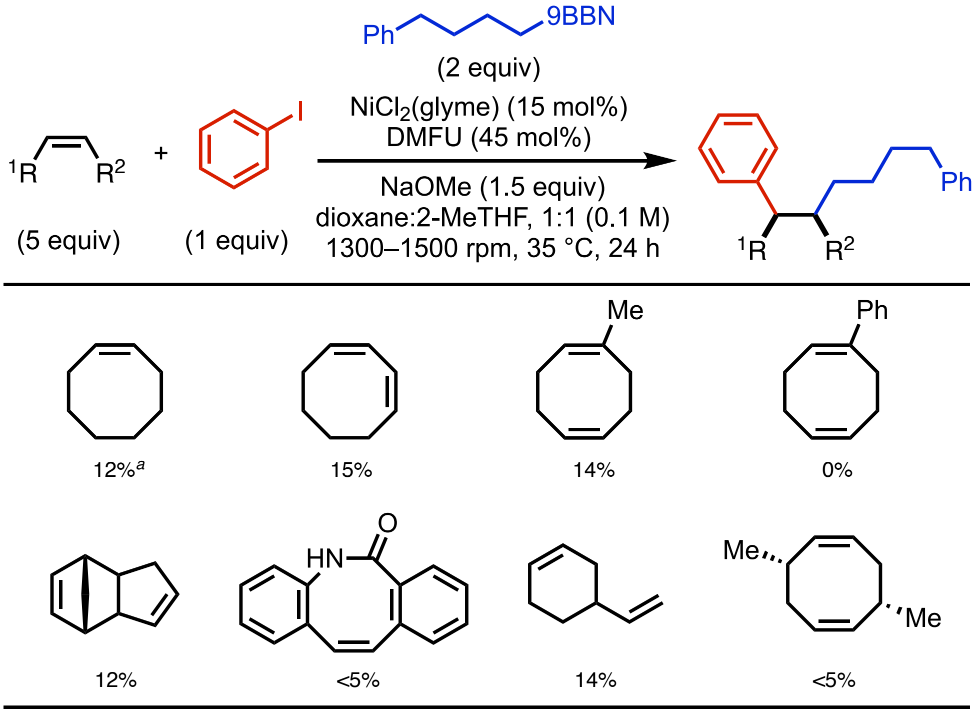

Reaction conditions: PhI (0.1 mmol), alkyl–9-BBN (0.2 mmol in 0.5 mL 2-MeTHF), NaOMe (0.15 mmol), olefin (0.5 mmol), $NiCl_2$(glyme) (0.015 mmol), ligand (0.045 mmol), 1,4-dioxane (0.5 mL). Yields are determined by ^{1}H NMR of the crude reaction mixture using CH_2Br_2 as internal standard. [a] The main products after end reaction is the two-component Suzuki–Miyaura and remaining aryl iodide.

Table S5: Limitations in Coupling Partner for the Ni-catalyzed Arylalkylation of 1,5-COD

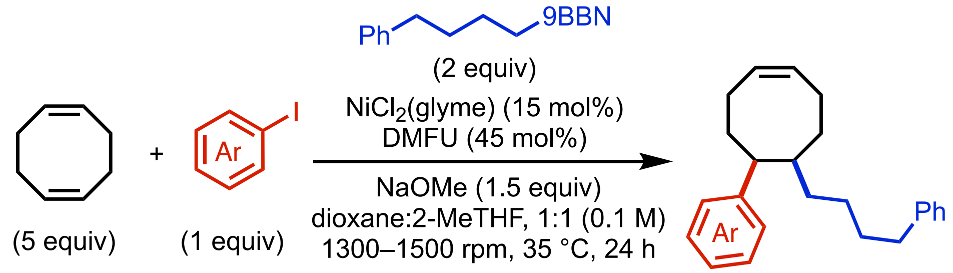

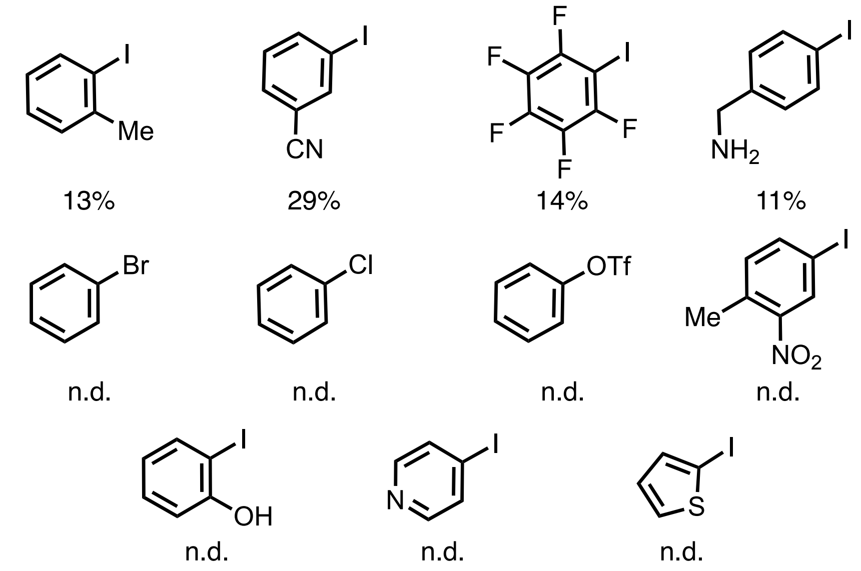

Reaction conditions: PhI (0.2 mmol), alkyl–9-BBN (0.4 mmol in 1 mL 2-MeTHF), NaOMe (0.30 mmol), 1,5-cyclooctadiene (1.0 mmol), $NiCl_2$(glyme) (0.030 mmol), ligand (0.090 mmol), 1,4-dioxane (1 mL). Yields are determined by ^{1}H NMR of the crude reaction mixture using CH_2Br_2 as internal standard.

General Procedure 1: Preparation of alkyl-9-borabicyclo[3.3.1]nonane solutions

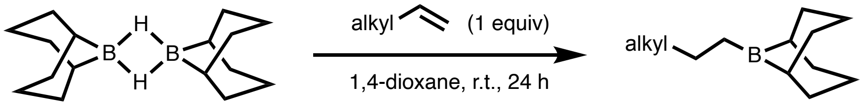

To a 20-mL vial with an x-shaped stir bar was added 9-borabicyclo[3.3.1]nonane dimer (0.5 equiv), 1,4-dioxane (0.5 M), and the corresponding alkene (1 equiv). The mixture was stirred at room temperature for 24 h unless stated otherwise. Hydroborylation was confirmed by ^{1}H NMR (C_6D_6) of an aliquot before the solution was diluted to reach the desired 0.4 M concentration in 1,4-dioxane.

Characterization of alkyl–9-BBN solutions in 1,4-dioxane

9-(4-Phenylbutyl)-9-borabicyclo[3.3.1]nonane (**3a**)

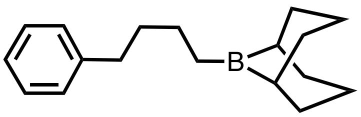

Following General Procedure 1 with but-3-en-1-ylbenzene (1 equiv) provided the desired solution of **3a** in 1,4-dioxane (0.4 M). **^{1}H NMR** (400 MHz, C_6D_6) δ 7.24–7.16 (m, 4H), 7.11–7.06 (m, 1H), 2.62–2.52 (m, 2H), 1.89–1.79 (m, 6H), 1.76–1.70 (m, 2H), 1.70–1.59 (m, 6H), 1.56–1.47 (m, 2H), 1.39–1.32 (m, 2H), 1.23–1.13 (m, 2H).

9-Phenethyl-9-borabicyclo[3.3.1]nonane (**S3k**)

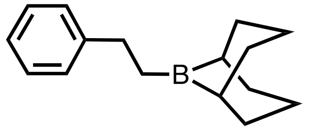

Following General Procedure 1 with styrene (1 equiv) provided the desired solution of **S3k** in 1,4-dioxane (0.4 M). ^{1}H NMR (400 MHz, C_6D_6) δ 7.23–7.17 (m, 4H), 7.11–7.06 (m, 1H), 2.85–2.77 (m, 2H), 1.86–1.67 (m, 10H), 1.65–1.54 (m, 4H), 1.19–1.10 (m, 2H).

9-(2-Cyclohexylethyl)-9-borabicyclo[3.3.1]nonane (**S3l**)

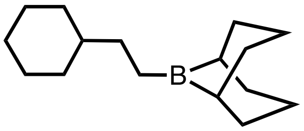

Following Reneral Procedure 1 with vinylcyclohexane (1 equiv) provided the desired solution of **S3l** in 1,4-dioxane (0.4 M). **^{1}H NMR** (400 MHz, C_6D_6) δ 1.91–1.63 (m, 17H), 1.50–1.36 (m, 4H), 1.31–1.10 (m, 6H), 0.97–0.85 (m, 2H).

9-Dodecyl-9-borabicyclo[3.3.1]nonane (**S3m**)

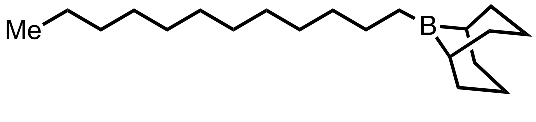

Following General Procedure 1 with dodec-1-ene (1 equiv) provided the desired solution of **S3m** in 1,4-dioxane (0.4 M). **^{1}H NMR** (400 MHz, C_6D_6) δ 1.90–1.80 (m, 6H), 1.80–1.73 (m, 2H), 1.74–1.62 (m, 4H), 1.62–1.51 (m, 2H), 1.44–1.14 (m, 22H), 0.93–0.84 (m, 3H).

9-(3,3,4,4,5,5,6,6,7,7,8,8,9,9,10,10,10-Heptadecafluorodecyl)-9-borabicyclo[3.3.1]nonane (**S3n**)

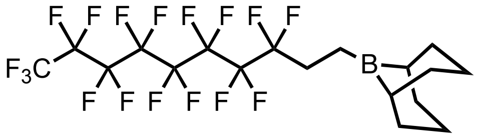

Following General Procedure 1 at 60 °C for 48 h with 3,3,4,4,5,5,6,6,7,7,8,8,9,9,10,10,10-heptadecafluorodec-1-ene (1 equiv) provided the desired solution of **S3n** in 1,4-dioxane (0.4 M). **^{1}H NMR** (400 MHz, C_6D_6) δ 2.20–2.02 (m, 2H), 1.78–1.68 (m, 6H), 1.57–1.45 (m, 6H), 1.45–1.38 (m, 2H), 1.49–1.03 (m, 2H). **^{19}F NMR** (376

MHz, C_6D_6) δ -80.90–-81.79 (m, 3F), -115.06–-116.13 (m, 2F), -121.23–-122.57 (m, 6F), -122.73–123.85 (m, 4F), -126.19–-126.80 (m, 2F).

(3-(9-Borabicyclo[3.3.1]nonan-9-yl)propyl)triphenylsilane (**S3o**)

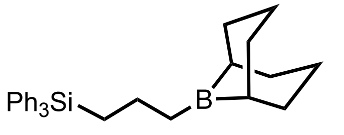

Following General Procedure 1 with allyltriphenylsilane (1 equiv) provided the desired solution of **S3o** in 1,4-dioxane (0.4 M). **^{1}H NMR** (400 MHz, C_6D_6) δ 7.61–7.57 (m, 6H), 7.15–7.11 (m, 9H), 1.84–1.76 (m, 2H), 1.76–1.66 (m, 6H), 1.64–1.58 (m, 2H), 1.58–1.47 (m, 4H), 1.47–1.40 (m, 4H), 1.11–1.01 (m, 2H).

9-(3-(3,4-Dimethoxyphenyl)propyl)-9-borabicyclo[3.3.1]nonane (**S3p**)

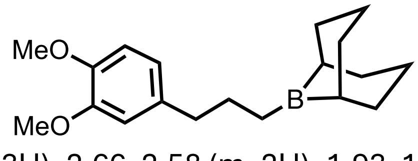

Following General Procedure 1 with 4-allyl-1,2-dimethoxybenzene (1 equiv) provided the desired solution of **S3p** in 1,4-dioxane (0.4 M). **^{1}H NMR** (400 MHz, C_6D_6) δ 6.79–6.76 (m, 1H), 6.72–6.66 (m, 2H), 3.48 (s, 3H), 3.46 (s, 3H), 2.66–2.58 (m, 2H), 1.93–1.74 (m, 10H), 1.72–1.61 (m, 4H), 1.45 (t, *J* = 7.8 Hz, 2H), 1.23–1.14 (m, 2H).

5-(-9-Borabicyclo[3.3.1]nonan-9-yl)pentyl pivalate (**S3q**)

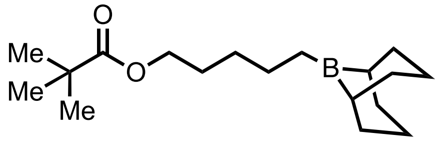

Following General Procedure 1 with pent-4-en-1-yl pivalate (1 equiv) provided the desired solution of **S3q** in 1,4-dioxane (0.4 M). **^{1}H NMR** (400 MHz, C_6D_6) δ 4.14–4.08 (m, 2H), 1.95–1.85 (m, 6H), 1.81–1.66 (m, 6H), 1.62–1.53 (m, 2H), 1.53–1.45 (m, 2H), 1.39–1.31 (m, 4H), 1.29–1.22 (m, 11H).

(3-(-9-Borabicyclo[3.3.1]nonan-9-yl)propoxy)(*tert*-butyl)dimethylsilane (**S3r**)

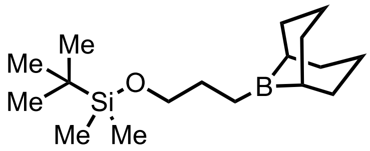

Following General Procedure 1 with (allyloxy)(*tert*-butyl)dimethylsilane (1 equiv) provided the desired solution of **S3r** in 1,4-dioxane (0.4 M). **^{1}H NMR** (400 MHz, C_6D_6) δ 3.52 (t, *J* = 6.3 Hz, 2H), 1.83–1.72 (m, 5H), 1.71–1.65 (m, 2H), 1.65–1.55 (m, 3H), 1.55–1.41 (m, 3H), 1.41–1.25 (m, 3H), 1.17–1.06 (m, 2H), 0.92 (s, 9H), 0.00 (s, 6H).

tert-Butyl 4-((9-borabicyclo[3.3.1]nonan-9-yl)methyl)piperidine-1-carboxylate (**S3s**)

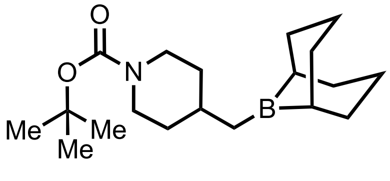

Following General Procedure 1 with *tert*-butyl 4-methylenepiperidine-1-carboxylate (1 equiv) provided the desired solution of **S3s** in 1,4-dioxane (0.4 M). **^{1}H NMR** (400 MHz, C_6D_6) δ 2.65–2.49 (m, 2H), 1.87–1.79 (m, 7H), 1.68–1.54 (m, 8H), 1.51 (s, 9H), 1.43–1.33 (m, 2H), 1.23–1.13 (m, 4H), 1.10–0.98 (m, 2H).

N-(5-(-9-Borabicyclo[3.3.1]nonan-9-yl)pentyl)-*N*,4-dimethylbenzenesulfonamide (**S3t**)

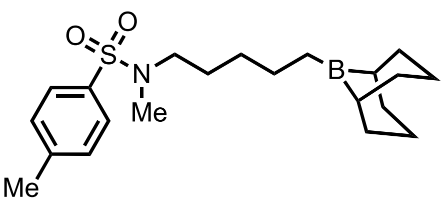

Following General Procedure 1 with *N*,4-dimethyl-*N*-(pent-4-en-1-yl)benzenesulfonamide (1 equiv) provided the desired solution of **S3t** in 1,4-dioxane (0.4 M). **^{1}H NMR** (400 MHz, C_6D_6) δ 7.61 (d, *J* = 8.2 Hz, 2H), 6.75 (d, *J*

= 7.8 Hz, 2H), 2.80 (t, J = 6.9 Hz, 2H), 2.36 (s, 3H), 1.87–1.73 (m, 9H), 1.72–1.56 (m, 6H), 1.47–1.33 (m, 2H), 1.33–1.09 (m, 2H).

tert-Butyl (2-(3-(-9-borabicyclo[3.3.1]nonan-9-yl)propoxy)phenyl)carbamate (**S3u**)

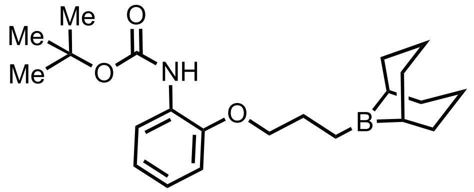

Following General Procedure 1 with *tert*-butyl (2-(3-(9-borabicyclo[3.3.1]nonan-9-yl)propoxy)phenyl)carbamate (1 equiv) provided the desired solution of **S3u** in 1,4-dioxane (0.4 M). ^{1}**H NMR** (400 MHz, C_6D_6) δ 8.74 (s, 1H), 7.43 (s, 1H), 6.97–6.92 (m, 1H), 6.88–6.82 (m, 1H), 6.65 (dd, J = 8.1, 1.4 Hz, 1H), 3.59 (t, J = 6.9 Hz, 2H), 1.88–1.75 (m, 6H), 1.71–1.55 (m, 8H), 1.41 (s, 9H), 1.24–1.11 (m, 4H).

2-(5-(-9-Borabicyclo[3.3.1]nonan-9-yl)pentyl)isoindoline-1,3-dione (**S3v**)

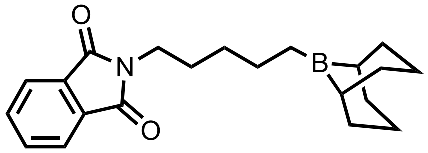

Following General Procedure 1 with 2-(5-(9-borabicyclo[3.3.1]nonan-9-yl)pentyl)isoindoline-1,3-dione (1 equiv) provided the desired solution of **S3v** in 1,4-dioxane (0.4 M). ^{1}**H NMR** (400 MHz, C_6D_6) δ 7.50–7.43 (m, 2H), 6.92–6.85 (m, 2H), 3.63–3.54 (m, 2H), 1.86–1.70 (m, 7H), 1.70–1.51 (m, 9H), 1.46–1.35 (m, 2H), 1.31–1.22 (m, 2H), 1.19–1.11 (m, 2H).

9-Methoxy-9-borabicyclo[3.3.1]nonane (**S3x**)

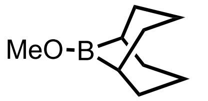

In a nitrogen-filled glovebox to a 20-mL vial containing an x-shaped stir bar was added 9-borabicyclo[3.3.1]nonane dimer (1.22 g, 5 mmol, 1 equiv) and THF (4 mL). Anhydrous MeOH (405 μL, 2 equiv) was added dropwise while stirring. Gas evolution was observed. After the gas evolution had ceased, additional MeOH (810 μL) was added. The reaction was stirred for 2 h at room temperature before the solution was concentrated under vacuum to obtain the desired compound **S3x**. The product was used without further purification. ^{1}**H NMR** (400 MHz, C_6D_6) δ 3.44 (s, 3H), 1.88–1.69 (m, 10H), 1.43–1.28 (m, 4H). The data is in accordance with literature report.[9]

General Procedure 2: Nickel-Catalyzed Arylalkylation of 1,5-Cyclooctadiene

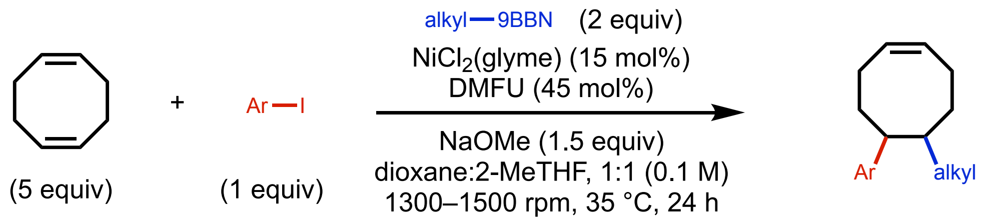

In a nitrogen-filled glovebox, to an oven-dried 1-dram vial containing a stir bar were added in order $NiCl_2$(glyme) (6.6 mg, 15 mol%) (unless otherwise stated), dimethyl fumarate (13.0 mg, 45 mol%), 2-methyltetrahydrofuran (1 mL), 1,5-cyclooctadiene (123 μL, 5 equiv), and aryl iodide (0.2 mmol, 1 equiv). In the meantime, a solution of NaOMe (16.2 mg, 1.5 equiv) and alkyl-9-borabicyclo[3.3.1]nonane solution (1 mL, 0.4 M in 1,4-dioxane) was pre-stirred in a 1-dram vial for 5–10 min at rt before being added to the first reaction mixture. The vial was sealed and transferred out of the glovebox to a heating block to stir at 35 °C and >1300 rpm for 24 h. The reaction was quenched with 1M HCl (aqueous) solution and diluted with Et_2O (5 mL). The aqueous phase was extracted with Et_2O (5 mL). The combined organic phases were washed with brine before being dried over Na_2SO_4 and concentrated under reduced pressure. The desired product was isolated by PTLC followed by eluction through a $AgNO_3$-impregnated silica gel plug. The latter step separated the two-component coupling product from the desired three-component coupling product.

General Procedure 3: Scale Up Procedure for Nickel-Catalyzed Arylalkylation of 1,5-Cyclooctadiene

In a nitrogen-filled glovebox, to an oven-dried 40-mL vial equipped with an x-shaped stir bar (to ensure vigorous stirring) were added in order $NiCl_2$(glyme) (65.9 mg, 15 mol%), dimethyl fumarate (130.0 mg, 45 mol%), 2-methyltetrahydrofuran (10 mL), 1,5-cyclooctadiene (1.23 mL, 5 equiv), and aryl iodide (1 equiv). In the meantime, a solution of NaOMe (162 mg, 1.5 equiv) and alkyl-9-borabicyclo[3.3.1]nonane solution (10 mL, 0.4 M in 1,4-dioxane) was pre-stirred in a 20-dram vial for 5–10 min at rt before being added to the first reaction mixture. The vial was sealed and transferred out of the glovebox to an oil bath to stir at 35 °C and >1300 rpm for 24 h. The reaction was quenched with 1M HCl (aqueous) solution and diluted with Et_2O (20 mL). The aqueous phase was extracted with Et_2O (20 mL). The combined organic phases were washed with brine before being dried over Na_2SO_4 and concentrated under reduced pressure. The product was isolated by column chromatography.

Characterization of 5,6-Arylalkylated Cyclooctenes (4)

(±,*Z*)-5-Phenyl-6-(4-phenylbutyl)cyclooct-1-ene (**4a**)

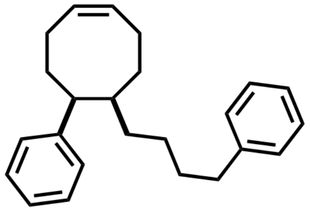

Following General Procedure 2 with iodobenzene (41 mg) and 9-(4-phenylbutyl)-9-borabicyclo[3.3.1]nonane (1 mL, 0.4 M in 1,4-dioxane). PTLC (hexanes) followed by elution through a $AgNO_3$ impregnated silica plug (2 cm^3 silica, hexanes then 10% Et_2O in hexanes) afforded the desired product as a colorless oil (47 mg, 73%). **Scale up**: Following General Procedure 3 with 4-(4-iodophenyl)morpholine (578 mg) and 9-(4-phenylbutyl)-9-borabicyclo[3.3.1]nonane (10 mL, 0.4 M in 1,4-dioxane). Silica gel column chromatography (hexanes) afforded a pure fraction of the desired product (150 mg, 24%). The remaining product was isolated in a fraction containing a mixture with the two-component coupling product. Corrected yield: 54% including the pure fraction. **^{1}H NMR** (400 MHz, $CDCl_3$) δ 7.30–7.07 (m, 10H), 5.72 (dt, *J* = 12.5, 6.5 Hz, 1H), 5.61 (dt, *J* = 12.7, 6.4 Hz, 1H), 3.06 (ddd, *J* = 12.8, 5.0, 2.7 Hz, 1H), 2.75–2.55 (m, 2H), 2.51 (t, *J* = 7.7 Hz, 2H), 2.38–2.25 (m, 1H), 2.15–2.04 (m, 2H), 1.98–1.85 (m, 1H), 1.78–1.46 (m, 5H), 1.40–1.31 (m, 2H), 1.26–1.13 (m, 2H). **^{13}C NMR** (101 MHz, $CDCl_3$) δ 146.57, 142.81, 129.91, 129.15, 128.41, 128.35, 128.22, 128.03, 125.66, 125.56, 46.14, 41.52, 35.84, 31.59, 30.51, 30.27, 27.51, 27.18, 25.93. Low resolution due to significant peak broadening hampered the assignment of carbon peaks. **GCMS** calcd. for $C_{24}H_{30}$: 318.2, Found: 318.2.

(±,*Z*)-5-(4-Methoxyphenyl)-6-(4-phenylbutyl)cyclooct-1-ene (**4b**)

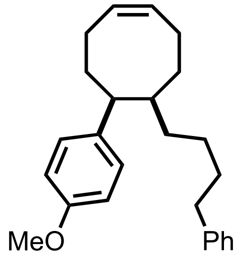

Following General Procedure 2 with 4-iodoanisole (47 mg) and 9-(4-phenylbutyl)-9-borabicyclo[3.3.1]nonane (1 mL, 0.4 M in dioxane). PTLC (2% EtOAc in hexanes) followed by a $AgNO_3$ impregnated silica plug (2 cm^3 silica, hexanes then 10% Et_2O in hexanes) afforded the desired product as a colorless oil (42 mg, 60%). **^{1}H NMR** (400 MHz, $CDCl_3$) δ 7.28–7.22 (m, 2H), 7.20–7.09 (m, 5H), 6.82 (d, *J* = 8.7 Hz, 2H), 5.76–5.65 (m, 1H), 5.62 (dt, *J* = 12.0, 5.9 Hz, 1H), 3.79 (s, 3H), 3.09–2.96 (m, 1H), 2.75–2.54 (m, 2H), 2.53 (t, *J* = 7.7 Hz, 2H), 2.36–2.23 (m, 1H), 2.16–2.02 (m, 2H), 1.87 (app bs, 1H), 1.67 (m, 2H), 1.60–1.48 (m, 3H), 1.41–1.30 (m, 2H), 1.22–1.14 (m, 2H). **^{13}C NMR** (101 MHz, $CDCl_3$) δ 157.57, 142.83, 138.49, 129.19, 128.40, 128.21, 125.56, 113.38, 55.22, 45.50, 41.22, 35.86, 31.61, 30.58, 30.44, 27.54, 25.94. Low resolution due to significant peak broadening hampered the assignment of carbon peaks. **HRMS** calcd. for $[C_{25}H_{33}O]^+$ 349.2531, found: 349.2526.

(±,*Z*)-5-(4-(*tert*-Butyl)phenyl)-6-(4-phenylbutyl)cyclooct-1-ene (**4c**)

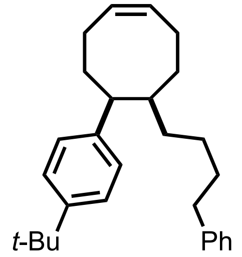

Following General Procedure 2 with 1-(tert-butyl)-4-iodobenzene (52 mg) and 9-(4-phenylbutyl)-9-borabicyclo[3.3.1]nonane (1 mL, 0.4 M in dioxane). PTLC (hexanes) followed by a $AgNO_3$ impregnated silica plug (2 cm^3 silica, hexanes then 10% Et_2O in hexanes) afforded the desired product as a colorless liquid (51 mg, 68%). **^{1}H NMR** (400 MHz, $CDCl_3$) δ 7.31–7.20 (m, 4H), 7.19–7.06 (m, 5H), 5.76–5.65 (m, 1H), 5.66–5.53 (m, 1H), 3.12–2.90 (m, 1H), 2.73–2.55 (m, 2H), 2.52 (t, *J* = 7.7 Hz, 2H), 2.36–2.23 (m, 1H), 2.15–2.02 (m, 2H), 1.94–1.85 (m, 1H), 1.78–1.61 (m, 2H), 1.61–1.48 (m, 3H), 1.42–1.33 (m, 2H), 1.31 (s, 9H), 1.27–1.13 (m, 2H). **^{13}C NMR** (101 MHz, $CDCl_3$) δ 148.25, 142.85, 128.39, 128.19, 127.88, 125.53, 124.84, 35.80, 34.30, 31.56, 31.44, 30.51, 27.45,

25.76. Low resolution due to significant peak broadening hampered the assignment of carbon peaks. **HRMS** calcd. for $[C_{28}H_{39}]^+$ 375.3052, found: 375.3056.

(±,*Z*)-5-(4-Phenylbutyl)-6-(4-(trifluoromethyl)phenyl)cyclooct-1-ene (**4d**)

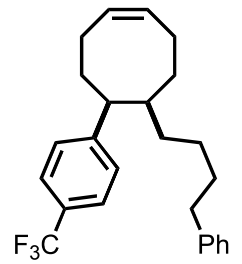

Following General Procedure 2 with 1-iodo-4-(trifluoromethyl) benzene (54.4 mg) and 9-(4-phenylbutyl)-9-borabicyclo[3.3.1]nonane (1 mL, 0.4 M in dioxane). PTLC (hexanes) followed by a $AgNO_3$ impregnated silica plug (2 cm^3 silica, hexanes then 10% Et_2O in hexanes) afforded the desired product as a colorless oil (24 mg, 31%). **^{1}H NMR** (400 MHz, $CDCl_3$) δ 7.52 (d, *J* = 7.9 Hz, 2H), 7.33–7.20 (m, 4H), 7.20–7.13 (m, 1H), 7.10 (d, *J* = 7.5 Hz, 2H), 5.79–5.68 (m, 1H), 5.66–5.55 (m, 1H), 3.18–3.09 (m, 1H), 2.71–2.56 (m, 2H), 2.52 (t, *J* = 7.6 Hz, 2H), 2.37–2.23 (m, 1H), 2.18–2.05 (m, 2H), 1.95–1.84 (m, 1H), 1.79–1.68 (m, 1H), 1.68–1.57 (m, 2H), 1.56–1.47 (m, 2H), 1.42–1.27 (m, 2H), 1.23–1.10 (m, 2H). **^{13}C NMR** (151 MHz, $CDCl_3$) δ 150.77, 142.61, 130.12, 128.77, 128.52, 128.34, 128.21, 127.91 (q, J_{C-F} = 32.2 Hz), 125.59, 124.91 (q, J_{C-F} = 3.8 Hz), 124.40 (q, J_{C-F} = 271.7 Hz), 45.82, 41.65, 35.77, 31.48, 30.39, 29.89, 29.71, 27.39, 26.73, 25.89. Low resolution due to significant peak broadening hampered the assignment of carbon peaks. **^{19}F NMR** (376 MHz, $CDCl_3$) δ -62.19. **GCMS** calcd. for $C_{25}H_{29}F_3$: 386.2, Found: 386.3.

(±,*Z*)-5-(4-Fluorophenyl)-6-(4-phenylbutyl)cyclooct-1-ene (**4e**)

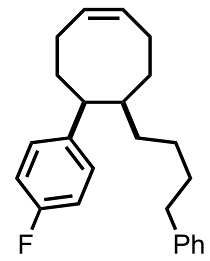

Following General Procedure 2 with 1-fluoro-4-iodobenzene (44.4 mg) and 9-(4-phenylbutyl)-9-borabicyclo[3.3.1]nonane (1 mL, 0.4 M in dioxane). PTLC (hexanes) followed by a $AgNO_3$ impregnated silica plug (2 cm^3 silica, hexanes then 10% Et_2O in hexanes) afforded the desired product as a colorless oil (32 mg, 48%). **^{1}H NMR** (400 MHz, $CDCl_3$) δ 7.29–7.21 (m, 2H), 7.18–7.07 (m, 5H), 7.01–6.90 (m, 2H), 5.76–5.66 (m, 1H), 5.65–5.55 (m, 1H), 3.10–3.01 (m, 1H), 2.72–2.56 (m, 2H), 2.52 (t, *J* = 7.7 Hz, 2H), 2.32–2.20 (m, 1H), 2.15–2.04 (m, 2H), 1.90–1.81 (m, 1H), 1.76–1.45 (m, 4H), 1.42–1.10 (m, 5H).**^{13}C NMR** (101 MHz, $CDCl_3$) δ 161.07 (d, J_{C-F} = 243.4 Hz), 142.73, 129.55 (d, J_{C-F} = 7.6 Hz), 128.31 (d, J_{C-F} = 16.1 Hz), 125.59, 114.67 (d, J_{C-F} = 20.7 Hz), 35.82, 31.55, 30.37, 29.74, 27.46, 25.99. Low resolution due to significant peak broadening hampered the assignment of carbon peaks. **^{19}F NMR** (376 MHz, $CDCl_3$) δ -118.07. **GCMS** calcd. for $C_{24}H_{29}F$: 336.2, Found: 336.2.

(±,*Z*)-5-(4-Chlorophenyl)-6-(4-phenylbutyl)cyclooct-1-ene (**4f**)

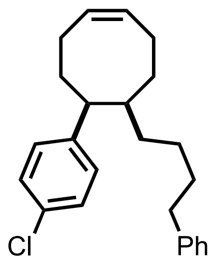

Following General Procedure 2 with 1-chloro-4-iodobenzene (48 mg) and 9-(4-phenylbutyl)-9-borabicyclo[3.3.1]nonane (1 mL, 0.4 M in dioxane). PTLC (hexanes) followed by a $AgNO_3$ impregnated silica plug (2 cm^3 silica, hexanes then 10% Et_2O in hexanes) afforded the desired product as a colorless oil (26 mg, 37%). ^{1}H NMR (400 MHz, $CDCl_3$) δ 7.29–7.20 (m, 4H), 7.19–7.14 (m, 1H), 7.15–7.08 (m, 4H), 5.72 (dt, *J* = 12.7, 6.5 Hz, 1H), 5.61 (dt, *J* = 11.3, 6.4 Hz, 1H), 3.10–2.98 (m, 1H), 2.71–2.56 (m, 2H), 2.52 (t, *J* = 7.7 Hz, 2H), 2.33–2.20 (m, 1H), 2.16–2.04 (m, 2H), 1.85 (m, 1H), 1.76–1.56 (m, 3H), 1.53–1.46 (m, 2H), 1.42–1.27 (m, 2H), 1.24–1.10 (m, 2H). ^{13}C NMR (101 MHz, $CDCl_3$) δ 144.99, 142.70, 131.27, 129.65, 128.97, 128.39, 128.24, 128.10, 125.60, 45.41, 41.53, 35.82, 31.53, 30.39, 30.13, 29.74, 27.46, 26.95, 25.99. Low resolution due to significant peak broadening hampered the assignment of carbon peaks. **GCMS** calcd. for $C_{24}H_{29}Cl$: 352.2, Found: 352.8.

1-(±,*Z*)-8-(4-Phenylbutyl)cyclooct-4-en-1-yl)naphthalene (**4g**)

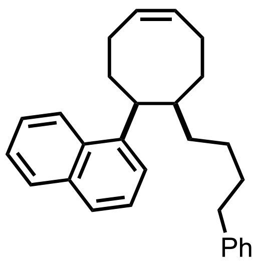

Following General Procedure 2 with 1-iodonaphthalene (51 mg) and 9-(4-phenylbutyl)-9-borabicyclo[3.3.1]nonane (1 mL, 0.4 M in dioxane). PTLC (hexanes) followed by a $AgNO_3$ impregnated silica plug (2 cm^3 silica, hexanes then 10% Et_2O in hexanes) afforded the desired product as a colorless solid (28 mg, 38%). **^{1}H NMR** (400 MHz, $CDCl_3$) δ 8.11 (d, *J* = 8.2 Hz, 1H), 7.85 (d, *J* = 8.1 Hz, 1H), 7.72–7.65 (m, 1H), 7.55–7.38 (m, 4H), 7.23–7.07 (m, 3H), 6.98 (d, *J* = 7.7 Hz, 2H), 5.94 (dt, *J* = 11.5, 6.2 Hz, 1H), 5.71–5.58 (m, 1H), 4.13–4.01 (m, 1H), 2.81–2.66 (m, 1H), 2.65–2.53 (m, 1H), 2.47–2.25 (m, 4H), 2.22–2.10 (m, 1H), 2.01–1.76 (m, 3H), 1.71–1.58 (m, 1H), 1.51–1.31 (m, 5H), 1.02–0.92 (m, 1H). **^{13}C NMR** (101 MHz, $CDCl_3$) δ 143.59, 142.72, 134.17, 132.31, 131.65, 129.08, 128.35, 128.16, 127.64, 126.28, 125.71, 125.50, 125.24, 125.10, 124.01, 123.57, 42.47, 37.37, 35.66, 31.54, 30.64, 29.76, 28.36, 27.33, 26.18, 25.39. **GCMS** calcd. for $C_{28}H_{32}$: 368.2, Found: 368.1.

tert-Butyl 6-((±,*Z*)-8-(4-phenylbutyl)cyclooct-4-en-1-yl)-1*H*-indole-1-carboxylate (**4h**)

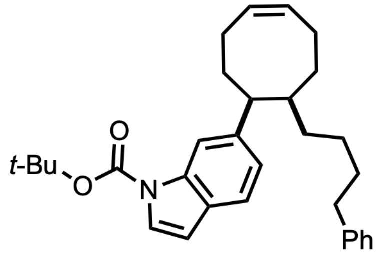

Following General Procedure 2 with *tert*-butyl 6-iodo-1*H*-indole-1-carboxylate (69 mg) and 9-(4-phenylbutyl)-9-borabicyclo[3.3.1]nonane (1 mL, 0.4 M in dioxane). PTLC (5% EtOAc in hexanes) followed by a $AgNO_3$ impregnated silica column (2 cm^3 silica, hexanes to 10% EtOAc in hexanes) afforded the desired product as a colorless oil (43 mg, 47%). **^{1}H NMR** (400 MHz, $CDCl_3$) δ 7.56 (bs, 1H), 7.38 (bs, 1H), 7.25–7.19 (m, 2H), 7.20–7.10 (m, 2H), 7.08 (d, *J* = 7.3 Hz, 2H), 6.52 (bs, 1H), 5.73 (dt, *J* = 12.9, 7.1 Hz, 1H), 5.63 (dt, *J* = 12.2, 6.2 Hz, 1H), 3.21–3.09 (m, 1H), 2.81–2.56 (m, 2H), 2.50 (t, *J* = 7.6 Hz, 2H), 2.45–2.38 (m, 1H), 2.20–2.08 (m, 2H), 1.98–1.89 (m, 1H), 1.79–1.62 (m, 12H), 1.53–1.45 (m, 2H), 1.42–1.32 (m, 2H), 1.23–1.12 (m, 2H). **^{13}C NMR** (101 MHz, $CDCl_3$) δ 150.00, 142.93, 141.02, 133.57, 130.63, 129.94, 129.34, 128.49, 128.29, 125.94, 125.63, 125.30, 120.20, 114.64, 107.48, 83.55, 35.97, 31.71, 30.74, 30.59, 29.85, 28.35, 27.68, 26.19. **HRMS** calcd. for $[C_{31}H_{39}NO_2]^+$ 458.3059, found: 458.3065.

4-(4-(±,*Z*)-8-(4-Phenylbutyl)cyclooct-4-en-1-yl)phenyl)morpholine (**4i**)

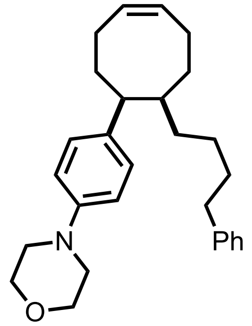

Following General Procedure 2 with 4-(4-iodophenyl)morpholine (58 mg) and 9-(4-phenylbutyl)-9-borabicyclo[3.3.1]nonane (1 mL, 0.4 M in dioxane). PTLC (10% EtOAc in hexanes) followed by a $AgNO_3$ impregnated silica column (2 cm^3 silica, hexanes to 50% Et_2O in hexanes) afforded the desired product as a colorless oil (52 mg, 64%).

Scale up: Following General Procedure 3 using 4-(4-iodophenyl)morpholine (578 mg) and 9-(4-phenylbutyl)-9-borabicyclo[3.3.1]nonane (10 mL, 0.4 M in dioxane). Silica gel column chromatography (2–7% EtOAc in hexanes) afforded a pure fraction of the desired product (270 mg, 33%). The remaining product was isolated in a fraction containing a mixture with the two-component coupling product. Corrected yield: 66% including pure fraction. **^{1}H NMR** (400 MHz, $CDCl_3$) δ 7.28–7.20 (m, 2H), 7.19–7.07 (m, 5H), 6.83 (d, *J* = 8.7 Hz, 2H), 5.74–5.64 (m, 1H), 5.66–5.55 (m, 1H), 3.84 (dd, *J* = 5.7, 3.9 Hz, 4H),

3.18–3.04 (m, 4H), 3.02–2.94 (m, 1H), 2.75–2.47 (m, 4H), 2.35–2.22 (m, 1H), 2.15–2.01 (m, 2H), 1.92–1.80 (m, 1H), 1.75–1.60 (m, 2H), 1.59–1.45 (m, 3H), 1.40–1.29 (m, 2H), 1.22–1.09 (m, 2H). **^{13}C NMR** (101 MHz, $CDCl_3$) δ 149.14, 142.86, 138.04, 129.39, 129.03, 128.41, 128.21, 125.56, 115.41, 67.05, 49.61, 35.88, 31.64, 30.46, 29.75, 27.56, 25.92. **HRMS** calcd. for $[C_{28}H_{37}NO]^+$ 404.2953, found: 404.2961

Methyl 4-((±,*Z*)-8-(4-phenylbutyl)cyclooct-4-en-1-yl)benzoate (**4j**)

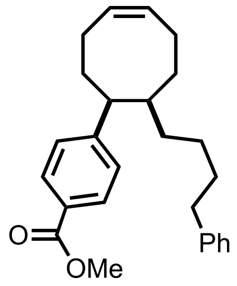

Following General Procedure 2 with methyl 4-iodobenzoate (52.4 mg) and 9-(4-phenylbutyl)-9-borabicyclo[3.3.1]nonane (1 mL, 0.4 M in dioxane). PTLC (2% EtOAc in hexanes) followed by a $AgNO_3$ impregnated silica plug (2 cm^3 silica, hexanes then 10% Et_2O in hexanes) afforded the desired product as a white solid (35 mg, 46%). **^{1}H NMR** (400 MHz, $CDCl_3$) δ 7.94 (d, *J* = 8.4 Hz, 2H), 7.30–7.18 (m, 4H), 7.18–7.11 (m, 1H), 7.12–7.05 (m, 2H), 5.77–5.66 (m, 1H), 5.66–5.54 (m, 1H), 3.89 (s, 3H), 3.18–3.11 (m, 1H), 2.71–2.56 (m, 2H), 2.51 (t, *J* = 7.6 Hz, 2H), 2.37–2.24 (m, 1H), 2.17–2.05 (m, 2H), 1.93–1.82 (m, 1H), 1.78–1.55 (m, 4H), 1.54–1.44 (m, 2H), 1.38–1.28 (m, 2H), 1.20–1.10 (m, 1H). **^{13}C NMR** (100 MHz, $CDCl_3$) δ 167.31, 152.40, 142.74, 129.46, 128.45, 128.39, 128.30, 127.69, 125.66, 52.06, 35.87, 31.58, 31.09, 30.54, 29.83, 27.51, 26.10. **HRMS** calcd. for $[C_{26}H_{33}O_2]^+$ 377.2481, found: 377.2486.

(±,*Z*)-5-(4-(*tert*-Butyl)phenyl)-6-phenethylcyclooct-1-ene (**4k**)

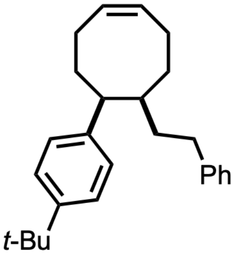

Following General Procedure 2 with 1-*tert*-butyl-4-iodobenzene (52 mg) and 9-phenethyl-9-borabicyclo[3.3.1]nonane (1 mL, 0.4 M in dioxane). PTLC (5% EtOAc hexanes) followed by a $AgNO_3$ impregnated silica column (2 cm^3 silica, 1-20% EtOAc in hexanes) afforded the desired product as a colorless oil (39 mg, 67%). **^{1}H NMR** (600 MHz, $CDCl_3$) δ 7.31–7.26 (m, 2H), 7.23 (t, *J* = 7.5 Hz, 2H), 7.19–7.11 (m, 3H), 7.08 (d, *J* = 7.5 Hz, 2H), 5.77–5.69 (m, 1H), 5.65–5.58 (m, 1H), 3.11–3.04 (m, 1H), 2.75–2.55 (m, 3H), 2.49–2.42 (m, 1H), 2.35–2.26 (m, 1H), 2.15–2.06 (m, 2H), 2.02–1.96 (m, 1H), 1.86–1.78 (m, 1H), 1.76–1.61 (m, 3H), 1.54–1.47 (m, 1H), 1.31 (s, 9H). **^{13}C NMR** (151 MHz, $CDCl_3$) δ 148.30, 142.91, 128.30, 128.18, 127.82, 125.52, 124.87, 34.27, 31.42, 30.57, 30.48, 25.60. **GCMS** calcd. for $C_{26}H_{34}$: 346.7, Found: 346.

(±,*Z*)-5-(4-(*tert*-Butyl)phenyl)-6-(2-cyclohexylethyl)cyclooct-1-ene (**4l**)

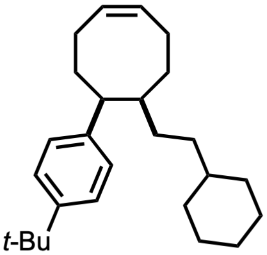

Following General Procedure 2 with 1-*tert*-butyl-4-iodobenzene (52 mg) and 9-(2-cyclohexylethyl)-9-borabicyclo[3.3.1]nonane (1 mL, 0.4 M in dioxane). PTLC (5% EtOAc hexanes) followed by a $AgNO_3$ impregnated silica column (2 cm^3 silica, hexanes to 5% Et_2O in hexanes) afforded the desired product as a colorless oil (51 mg, 72%). **^{1}H NMR** (400 MHz, $CDCl_3$) δ 7.30 (d, *J* = 8.5 Hz, 2H), 7.16 (d, *J* = 8.4 Hz, 2H), 5.78–5.69 (m, 1H), 5.67–5.58 (m, 1H), 3.06 (ddd, *J* = 12.7, 5.0, 2.5 Hz, 1H), 2.76–2.54 (m, 2H), 2.37–2.24 (m, 1H), 2.16–2.05 (m, 2H), 1.92–1.82 (m, 1H), 1.78–1.56 (m, 8H), 1.46–1.36 (m, 1H), 1.33 (s, 9H), 1.25–1.03 (m, 7H), 0.90–0.73 (m, 2H). **^{13}C NMR** (101 MHz, $CDCl_3$) δ 148.19, 130.06, 129.21, 127.84, 124.81, 37.92, 35.86, 34.30, 33.79, 33.21, 31.46, 30.68, 30.63, 26.79, 26.50, 26.44, 25.63. **GCMS** calcd. for $C_{26}H_{40}$: 352.3, Found: 352.2.

(±,*Z*)-5-(4-(*tert*-Butyl)phenyl)-6-dodecylcyclooct-1-ene (**4m**)

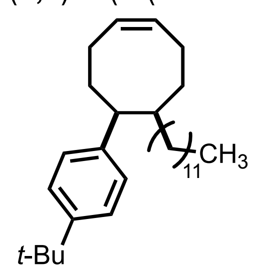

Following General Procedure 2 with 1-*tert*-butyl-4-iodobenzene (52 mg) and 9-dodecyl-9-borabicyclo[3.3.1]nonane (1 mL, 0.4 M in dioxane). PTLC (hexanes) followed by a $AgNO_3$ impregnated silica column (2 cm^3 silica, hexanes then 10% Et_2O in hexanes) afforded the desired product as a colorless oil (43 mg, 52%). **^{1}H NMR** (400 MHz, $CDCl_3$) δ 7.27 (t, *J* = 8.2 Hz, 2H), 7.14 (d, *J* = 8.0 Hz, 2H), 5.78–5.67 (m, 1H), 5.66–5.55 (m, 1H), 3.08–2.97 (m, 1H), 2.72–2.53 (m, 2H), 2.35–2.23 (m, 1H), 2.13–2.02 (m, 2H), 1.94–1.85 (m, 1H), 1.79–1.54 (m, 3H), 1.38–1.13 (m, 31H), 0.90–0.84 (m, 3H). **^{13}C NMR** (101 MHz, $CDCl_3$) δ 148.18, 130.47, 129.84, 129.30, 127.88, 124.82, 34.30, 31.97, 31.46, 30.65, 30.60, 29.95, 29.71, 29.40, 28.03, 25.65, 25.34, 22.73, 14.16. Low resolution due to significant peak broadening hampered the assignment of carbon peaks. **GCMS** calcd. for $C_{30}H_{50}$: 410.39, Found: 410.

(±,*Z*)-5-(4-(*tert*-Butyl)phenyl)-6-(3,3,4,4,5,5,6,6,7,7,8,8,9,9,10,10,10-heptadecafluorodecyl)cyclooct-1-ene (**4n**)

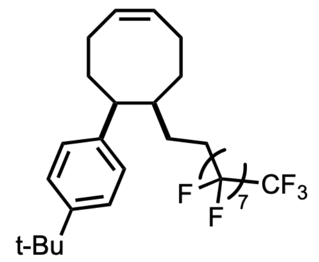

Following General Procedure 2 with 1-*tert*-butyl-4-iodobenzene (52 mg) and 9-(3,3,4,4,5,5,6,6,7,7,8,8,9,9,10,10,10-heptadecafluorodecyl)-9-borabicyclo[3.3.1]nonane (1 mL, 0.4 M in dioxane). Preparatory TLC (5% EtOAc hexanes) followed by a $AgNO_3$ impregnated silica column (2 cm^3 silica, hexanes) afforded the desired product as a colorless solid (91 mg, 66%). **^{1}H NMR** (400 MHz, $CDCl_3$) δ 7.32 (d, *J* = 8.4 Hz, 2H), 7.15 (d, *J* = 8.4 Hz, 2H), 5.78–5.60 (m, 2H), 3.16–3.03 (m, 1H), 2.83–2.68 (m, 1H), 2.66–2.54 (m, 1H), 2.44–2.32 (m, 1H), 2.21–1.83 (m, 5H), 1.83–1.65 (m, 3H), 1.64–1.56 (m, 1H), 1.51–1.40 (m, 1H), 1.32 (s, 9H). **^{13}C NMR** (151 MHz, $CDCl_3$) δ 148.99, 142.22, 129.78, 129.20, 127.97, 125.29, 118.73 (tt, *J* = 254.7, 31.4 Hz), 117.30 (qt, *J* = 288.3, 33.0 Hz), 113.60–111.89 (m, $J_{C\text{–}F}$), 111.87–109.87 (m, $J_{C\text{–}F}$), 109.87–106.92 (m, $J_{C\text{–}F}$), 46.13, 40.90, 34.47, 31.49, 30.45, 30.37, 29.65 (t, $J_{C\text{–}F}$ = 22.0 Hz), 27.65, 25.67, 21.79. **^{19}F NMR** (376 MHz, $CDCl_3$) δ -80.73 (t, $J_{F\text{–}F}$ = 9.5 Hz, 3F), -114.64 (t, $J_{F\text{–}F}$ = 14.7 Hz, 2F), -121.75 (bs, 2F), -121.94 (bs, 4F), -122.73 (bs, 2F), -123.50 (bs, 2F), -126.11 (t, $J_{F\text{–}F}$ = 15.6 Hz, 2F). **GCMS** calcd. for $C_{28}H_{29}F_{17}$: 688.20, Found: 688.

(±,*Z*)-5-(3,3,4,4,5,5,6,6,7,7,8,8,9,9,10,10,10-Heptadecafluorodecyl)-6-phenylcyclooct-1-ene (**4n'**)

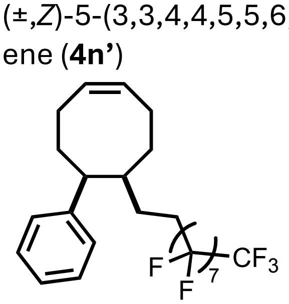

Scale up: Following General Procedure 3 with iodobenzene (408 mg) and 9-(3,3,4,4,5,5,6,6,7,7,8,8,9,9,10,10,10-heptadecafluorodecyl)-9-borabicyclo[3.3.1]nonane (10 mL, 0.4 M in dioxane). Silica gel column chromatography in hexanes afforded the desired product as a white solid (787 mg, 62%). **^{1}H NMR** (600 MHz, $CDCl_3$) δ 7.32–7.28 (m, 2H), 7.23–7.18 (m, 3H), 5.75–5.68 (m, 1H), 5.69–5.54 (m, 1H), 3.13–3.06 (m, 1H), 2.80–2.70 (m, 1H), 2.65–2.55 (m, 1H), 2.43–2.34 (m, 1H), 2.20–2.12 (m, 2H), 2.09–2.01 (m, 1H), 2.01–1.94 (m, 1H), 1.92–1.82 (m, 1H), 1.80–1.64 (m, 3H), 1.63–1.57 (m, 1H), 1.46–1.38 (m, 1H). **^{13}C NMR** (151 MHz, $CDCl_3$) δ 145.20, 129.58, 129.06, 128.30, 128.24, 126.12, 118.53 (tt, *J* = 254.2, 31.6 Hz), 117.14 (qt, *J* = 288.3, 33.6 Hz), 113.37–111.68 (m, $J_{C\text{–}F}$), 111.66–109.73 (m, $J_{C\text{–}F}$), 109.70–105.99 (m, $J_{C\text{–}F}$), 46.60, 40.83, 30.14, 29.74, 29.53

(t, J_{C-F} = 22.0 Hz), 27.46, 25.59, 21.62. **^{19}F NMR** (376 MHz, $CDCl_3$) δ -80.72 (t, J_{F-F} = 9.7 Hz, 3F), -114.14–114.87 (m, 2F), -121.72 (bs, 2F), -121.92 (bs, 4F), -122.71 (bs, 2F), -123.46 (bs, 2F), -126.09 (bs, 2F). **GCMS** calcd. for $C_{24}H_{21}F_{17}$: 632.1, Found: 632.

(3-((±,*Z*)-8-(4-(*tert*-Butyl)phenyl)cyclooct-4-en-1-yl)propyl)triphenylsilane (**4o**)

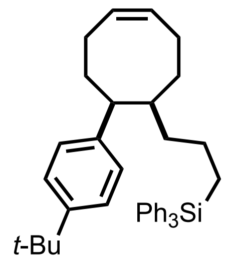

Following General Procedure 2 with 1-*tert*-butyl-4-iodobenzene (52 mg) and (3-(9-borabicyclo[3.3.1]nonan-9-yl)propyl)triphenylsilane (1 mL, 0.4 M in dioxane). PTLC (hexanes) followed by a $AgNO_3$ impregnated silica column (2 cm^3 silica, hexanes then 10% Et_2O in hexanes) afforded the desired product as a white solid (55 mg, 50%). **^{1}H NMR** (400 MHz, $CDCl_3$) δ 7.50–745 (m, 6H), 7.43–7.28 (m, 10H), 7.27–7.20 (m, 2H), 7.07–7.00 (m, 2H), 5.73–5.63 (m, 1H), 5.62–5.53 (m, 1H), 2.99–2.90 (m, 1H), 2.69–2.47 (m, 2H), 2.29–2.16 (m 1H), 2.10–1.97 (m, 2H), 1.93–1.83 (m, 1H), 1.69–1.38 (m, 5H), 1.36–1.22 (m, 11H). **^{13}C NMR** (101 MHz, $CDCl_3$) δ 148.20, 135.79, 135.67, 135.46, 129.35, 127.92, 127.85, 124.85, 34.33, 31.49, 30.57, 25.72, 22.20, 13.43. Low resolution due to significant peak broadening hampered the assignment of carbon peaks. Unable to obtain mass spectrometry data by HRMS and GCMS. Derivatization to the epoxide was performed to confirm the spectroscopic assignment and obtain MS data.

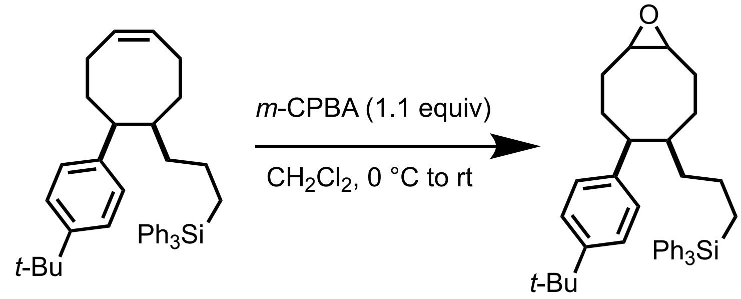

The product **4o** (38 mg, 79 μmol) was dissolved in CH_2Cl_2 (2 mL) in a 20-mL vial equipped with a stir bar. The reaction was cooled to 0 °C before a stock solution of *m*-CPBA (21 mg, 1.1 equiv) in CH_2Cl_2 (3 mL) was added dropwise. The reaction was allowed to reach rt and stirred until full conversion of alkene was observed by TLC analysis. The reaction was quenched with $NaHCO_3$ (sat. aq.) and the organic phase extracted with CH_2Cl_2 (5 mL). The combined organic phases were washed with $NaHCO_3$ (sat. aq.) and brine before dried over Na_2SO_4. PTLC (10% EtOAc in hexanes) afforded the desired epoxide of **4o** in 90% purity. The diastereomeric ratio was not assigned. **^{1}H NMR** (600 MHz, $CDCl_3$) δ 7.49–7.45 (m, 6H), 7.41–7.37 (m, 3H), 7.36–7.31 (m, 6H), 7.27–7.23 (m, 2H), 7.02 (d, *J* = 8.4 Hz, 2H), 3.07–3.01 (m, 1H), 3.01–2.93 (m, 1H), 2.88–2.81 (m, 1H), 2.15–2.08 (m, 1H), 2.08–2.00 (m, 1H), 1.98–1.90 (m, 1H), 1.90–1.84 (m, 1H), 1.70–1.46 (m, 7H), 1.39–1.23 (m, 31H). **^{13}C NMR** (151 MHz, $CDCl_3$) δ 148.49, 135.56, 135.21, 129.35, 127.82, 125.12, 56.38, 55.67, 34.28, 31.38, 27.83, 27.07, 25.85, 22.45, 13.44. Low resolution due to significant peak broadening hampered the assignment of carbon peaks. **HRMS** calcd. for $[C_{39}H_{47}OSi]^+$ 559.3396, found: 559.3406

(±,*Z*)-5-(4-(*tert*-Butyl)phenyl)-6-(3-(3,4-dimethoxyphenyl)propyl)cyclooct-1-ene (**4p**)

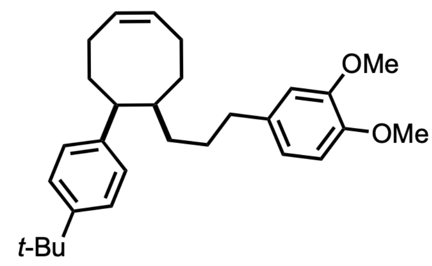

Following General Procedure 2 with 1-*tert*-butyl-4-iodobenzene (52 mg) and 9-(3-(3,4-dimethoxyphenyl)propyl)-9-borabicyclo[3.3.1]nonane (1 mL, 0.4 M in dioxane). PTLC (5% EtOAc hexanes) followed by a $AgNO_3$ impregnated silica column (2 cm^3 silica, 1-20% EtOAc in hexanes) afforded the desired product as a colorless oil (55 mg, 65%). **^{1}H NMR** (400 MHz, $CDCl_3$) δ 7.30 (d, *J* = 8.4 Hz, 2H), 7.14 (d, *J* = 8.4 Hz, 2H), 6.77 (d, *J* = 8.0 Hz, 1H), 6.71–6.62 (m, 2H), 5.73 (dt, *J* = 13.1, 6.0 Hz, 1H), 5.69–5.58 (m, 1H), 3.87 (s, 3H), 3.86 (s, 3H), 3.05 (ddd, *J* = 12.6, 5.1, 2.5 Hz, 1H), 2.77–2.44 (m, 4H), 2.39–2.25 (m, 1H), 2.18–2.04 (m, 2H), 2.01–1.95 (m, 1H), 1.81–1.54 (m, 4H), 1.52–1.40 (m, 2H), 1.33 (s, 12H), 1.31–1.21 (m, 1H). **^{13}C NMR** (101 MHz, $CDCl_3$) δ 148.80, 148.35, 147.07, 143.32, 135.63, 129.52, 128.00, 124.93, 120.19, 111.80, 111.22, 56.00, 55.88, 45.88, 41.15, 35.84, 34.39, 31.53, 30.68, 30.62, 30.24, 27.53, 25.68. **HRMS** calcd. for $[C_{29}H_{41}O_2–C(CH_3)_3]^+$ 365.2481, found: 365.2482.

5-((±,*Z*)-8-(4-(*tert*-Butyl)phenyl)cyclooct-4-en-1-yl)pentyl pivalate (**4q**)

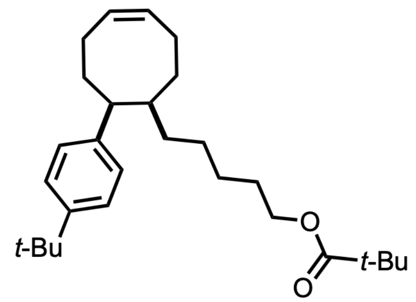

Following General Procedure 2 with 1-*tert*-butyl-4-iodobenzene (52 mg) and 5-(9-borabicyclo[3.3.1]nonan-9-yl)pentyl pivalate (1 mL, 0.4 M in dioxane). PTLC (5% EtOAc hexanes) followed by a $AgNO_3$ impregnated silica column (2 cm^3 silica, 1-20% EtOAc in hexanes) afforded the desired product as a colorless oil (37 mg, 45%). **^{1}H NMR** (400 MHz, $CDCl_3$) δ 7.29 (d, *J* = 8.4 Hz, 2H), 7.14 (d, *J* = 8.3 Hz, 2H), 5.77–5.68 (m, 1H), 5.66–5.58 (m, 1H), 4.00 (t, *J* = 6.7 Hz, 2H), 3.08–3.00 (m, 1H), 2.76–2.53 (m, 2H), 2.37–2.24 (m, 1H), 2.15–2.05 (m, 2H), 1.97–1.85 (m, 1H), 1.78–1.50 (m, 5H), 1.41–1.20 (m, 15 H), 1.19 (s, 9H). **^{13}C NMR** (101 MHz, $CDCl_3$) δ 178.66, 148.27, 127.88, 124.84, 64.44, 38.74, 34.31, 31.45, 30.52, 28.63, 27.61, 27.23, 26.18, 25.68. Low resolution due to significant peak broadening hampered the assignment of carbon peaks. **HRMS** calcd. for [C28H44NaO2]$^+$ 435.3239, found: 435.3234.

tert-Butyl (2-((±,*Z*)-8-(4-(*tert*-butyl)phenyl)cyclooct-4-en-1-yl)ethoxy)dimethylsilane (**4r**)

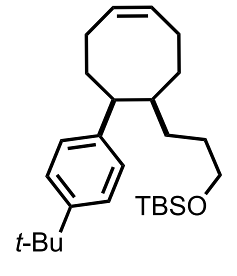

Following General Procedure 2 with 1-*tert*-butyl-4-iodobenzene (52 mg) and (2-(9-borabicyclo[3.3.1]nonan-9-yl)ethoxy)(*tert*-butyl)dimethylsilane (1 mL, 0.4 M in dioxane). PTLC (10% EtOAc in hexanes) followed by a $AgNO_3$ impregnated silica column (2 cm^3 silica, hexanes and then 10% Et_2O) afforded the desired product as a white solid (45 mg, 54%). **^{1}H NMR** (400 MHz, $CDCl_3$) δ 7.23 (d, *J* = 4.1 Hz, 2H), 7.10 (d, *J* = 8.3 Hz, 2H), 5.74–5.63 (m, 1H), 5.63–5.58 (m, 1H), 3.53 (t, *J* = 6.6 Hz, 2H), 3.04–2.95 (m, 1H), 2.72–2.51 (m, 2H), 2.33–2.20 (m, 1H), 2.13–2.01 (m, 2H), 1.92–1.82 (m, 1H), 1.75–1.57 (m, 2H), 1.47–1.38 (m, 2H), 1.38–1.09 (m, 18H), 0.85 (s, 9H). **^{13}C NMR** (101 MHz, $CDCl_3$) δ 127.86, 124.82, 63.32, 34.30, 32.89, 31.44, 30.54, 27.82, 26.10, 26.01, 25.63, 18.41, -5.23. Low resolution due to significant peak broadening hampered the assignment of carbon peaks. **GCMS** calcd. for $C_{27}H_{46}OSi$: 414.33, Found: 414.

tert-Butyl 4-(((±,*Z*)-8-(4-(*tert*-butyl)phenyl)cyclooct-4-en-1-yl)methyl)piperidine-1-carboxylate (**4s**)

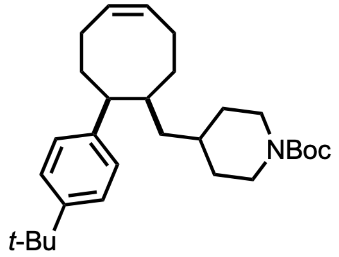

Following General Procedure 2 with 1-*tert*-butyl-4-iodobenzene (52 mg) and *tert*-butyl 4-((9-borabicyclo[3.3.1]nonan-9-yl)methyl)piperidine-1-carboxylate (1 mL, 0.4 M in dioxane). PTLC (5% EtOAc hexanes) followed by a $AgNO_3$ impregnated silica column (2 cm^3 silica, 1-20% EtOAc in hexanes) afforded the desired product as a colorless oil (49 mg, 56%). **^{1}H NMR** (400 MHz, $CDCl_3$) δ 7.28 (d, *J* = 8.4 Hz, 2H), 7.12 (d, *J* = 8.4 Hz, 2H), 5.76–5.67 (m, 1H), 5.67–5.57 (m, 1H), 3.99 (s, 2H), 3.09–2.97 (m, 1H), 2.75–2.52 (m, 4H), 2.38–2.24 (m, 1H), 2.17–2.03 (m, 3H), 1.76–1.62 (m, 2H), 1.58–1.48 (m, 3H), 1.43 (s, 9H), 1.31 (s, 9H), 1.28–0.98 (m, 4H), 0.87–0.74 (m, 1H). **^{13}C NMR** (101 MHz, $CDCl_3$) δ 154.98, 148.52, 129.44, 128.01, 124.97, 79.23, 45.81, 44.03, 37.85, 34.43, 33.42, 31.55, 30.87, 30.43, 28.60, 26.01. Low resolution due to significant peak broadening hampered the assignment of carbon peaks. **HRMS** calcd. for $[C_{28}H_{45}NNaO_2]^+$ 462.3348, found: 462.3363.

tert-Butyl 4-(((±,*Z*)-8-phenylcyclooct-4-en-1-yl)methyl)piperidine-1-carboxylate (**4s'**)

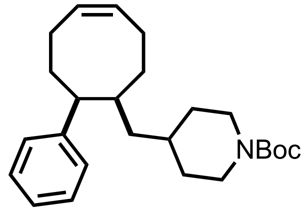

Scale up: Following General Procedure 3 with iodobenzene (408 mg) and *tert*-butyl 4-((9-borabicyclo[3.3.1]nonan-9-yl)methyl)piperidine-1-carboxylate (10 mL, 0.4 M in dioxane). Silica gel column chromatography (0-10% EtOAc in hexanes) followed by a $AgNO_3$ impregnated silica column (4 cm^3 silica, 1-10% EtOAc in hexanes) afforded a pure fraction of the desired product (180 mg, 24%). The remaining product was isolated in a fraction containing a mixture with the two-component coupling product. Corrected yield: 45% including pure fraction. **^{1}H NMR** (400 MHz, $CDCl_3$) δ 7.32–7.23 (m, 2H), 7.23–7.13 (m, 3H), 5.72 (dt, *J* = 12.7, 5.7 Hz, 1H), 5.68–5.58 (m, 1H), 3.99 (bs, 2H), 3.10–3.00 (m, 1H), 2.76–2.52 (m, 4H), 2.39–2.26 (m, 1H), 2.18–2.01 (m, 3H), 1.77–1.47 (m, 5H), 1.43 (s, 9H), 1.40–1.32 (m, 1H), 1.23–1.12 (m, 2H), 1.09–0.97 (m, 1H), 0.83–0.69 (m, 1H). **^{13}C NMR** (101 MHz, $CDCl_3$) δ 154.83, 128.30, 128.03, 125.74, 79.12, 33.27, 31.34, 30.66, 30.07, 28.47, 26.02. Low resolution due to significant peak broadening hampered the assignment of carbon peaks. **HRMS** calcd. for $[C_{25}H_{39}NO_2–C_5H_9O_2]^+$ 284.2378, found: 284.2385. **X-ray** (single-crystal) Colorless needles suitable for X-ray diffraction were grown by vapor diffusion in CH_2Cl_2:hexanes (CCDC 2472616).[10]

N-(5-((±,*Z*)-8-(4-(*tert*-Butyl)phenyl)cyclooct-4-en-1-yl)pentyl)-*N*,4-dimethylbenzenesulfonamide (**4t**)

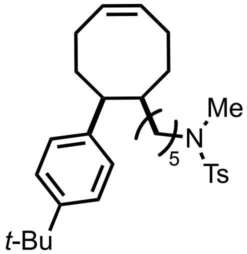

Following General Procedure 2 with $Ni(COD)_2$ (8.3 mg, 15 mol%), 1-*tert*-butyl-4-iodobenzene (52 mg) and *N*-(5-(9-borabicyclo[3.3.1]nonan-9-yl)pentyl)-*N*,4-dimethylbenzenesulfonamide (1 mL, 0.4 M in dioxane). PTLC (10% EtOAc in hexanes) followed by a $AgNO_3$ impregnated silica column (2 cm^3 silica, 0-10% EtOAc in hexanes) followed by PTLC (12% EtOAc in hexanes) afforded the desired product as a white solid (62 mg, 63%). **^{1}H NMR** (400 MHz, $CDCl_3$) δ 7.69–7.61 (m, 2H), 7.35–7.23 (m, 4H), 7.13 (d, *J* = 8.3 Hz, 2H), 5.77–5.66 (m, 1H), 5.66–5.56 (m, 1H), 3.07–2.99 (m, 1H), 2.92 (t, *J* = 7.3 Hz, 2H), 2.72–2.53 (m, 5H), 2.42 (s, 3H), 2.36–2.22 (m, 1H), 2.16–2.03 (m, 2H), 1.88 (s, 1H), 1.76–1.63 (m, 2H), 1.61–1.49 (m, 1H), 1.34–1.29 (m, 11H), 1.27–1.14 (m, 4H). **^{13}C NMR** (101 MHz, $CDCl_3$) δ 148.25, 143.15, 134.62, 129.61, 127.87, 127.42, 124.86, 50.12, 34.56, 34.31, 31.45, 30.51,

27.55, 26.78, 25.69, 21.52. Low resolution due to significant peak broadening hampered the assignment of carbon peaks. **HRMS** calcd. for $[C_{31}H_{46}NO_2S]^+$ 496.3249, found: 496.3266.

tert-Butyl (±,*Z*)-(2-(3-(8-(4-(*tert*-butyl)phenyl)cyclooct-4-en-1-yl)propoxy)phenyl)carbamate (**4u**)

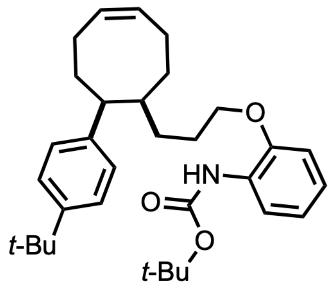

Following General Procedure 2 with 1-*tert*-butyl-4-iodobenzene (52 mg) and *tert*-butyl (2-(3-(9-borabicyclo[3.3.1]nonan-9-yl)propoxy)phenyl)carbamate (1 mL, 0.4 M in dioxane). PTLC (5% EtOAc hexanes) followed by a $AgNO_3$ impregnated silica column (2 cm^3 silica, 1-20% EtOAc in hexanes) afforded the desired product as a colorless oil (36 mg, 37%). **^{1}H NMR** (400 MHz, $CDCl_3$) δ 8.06 (bs, 1H), 7.28 (d, *J* = 8.3 Hz, 2H), 7.14 (d, *J* = 8.3 Hz, 2H), 7.06 (bs, 1H), 6.92 (dd, *J* = 6.0, 3.6 Hz, 2H), 6.77 (dd, *J* = 6.0, 3.5 Hz, 1H), 5.74 (m, 1H), 5.63 (m, 1H), 3.95–3.85 (m, 2H), 3.13–3.01 (m, 1H), 2.79–2.51 (m, 2H), 2.40–2.25 (m, 1H), 2.19–2.07 (m, 2H), 2.03–1.93 (s, 1H), 1.92–1.57 (m, 6H), 1.53 (s, 9H), 1.41–1.34 (m, 1H), 1.30 (s, 9H). **^{13}C NMR** (101 MHz, $CDCl_3$) δ 152.79, 148.48, 146.81, 128.25, 127.77, 124.99, 122.23, 120.93, 118.06, 110.97, 80.27, 68.76, 34.32, 31.43, 30.60, 30.54, 28.41, 27.65, 25.44. Low resolution due to significant peak broadening hampered the assignment of carbon peaks. **HRMS** calcd. for $[C_{32}H_{45}NNaO_3]^+$ 514.3297, found: 514.3311.

2-(5-((±,*Z*)-8-(4-(*tert*-Butyl)phenyl)cyclooct-4-en-1-yl)pentyl)isoindoline-1,3-dione (**4v**)

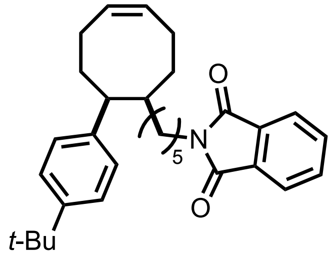

Following General Procedure 2 with 1-*tert*-butyl-4-iodobenzene (52 mg) and 2-(5-(9-borabicyclo[3.3.1]nonan-9-yl)pentyl)isoindoline-1,3-dione (1 mL, 0.4 M in dioxane). PTLC (10% EtOAc in hexanes) and then loaded with celite on a $AgNO_3$ impregnated silica column (2 cm^3 silica, 0-10% EtOAc in hexanes) afforded the desired product as a white solid (47 mg, 53%). **^{1}H NMR** (400 MHz, $CDCl_3$) δ 7.83 (dd, *J* = 5.4, 3.1 Hz, 2H), 7.69 (dd, *J* = 5.5, 3.0 Hz, 2H), 7.32–7.22 (m, 2H), 7.15–7.07 (m, 2H), 5.75–5.66 (m, 1H), 5.64–5.55 (m, 1H), 3.62 (t, *J* = 7.3 Hz, 2H), 3.06–2.98 (m, 1H), 2.71–2.52 (m, 2H), 2.34–2.22 (m, 1H), 2.13–2.03 (m, 2H), 1.92–1.83 (m, 1H), 1.77–1.50 (m, 6H), 1.40–1.33 (m, 2H), 1.31–1.28 (m, 9H), 1.27–1.16 (m, 3H). **^{13}C NMR** (101 MHz, $CDCl_3$) δ 168.46, 148.27, 133.85, 132.19, 127.84, 124.88, 123.16, 38.11, 34.29, 31.43, 30.42, 29.73, 28.93, 28.24, 25.74, 25.33. Low resolution due to significant peak broadening hampered the assignment of carbon peaks. **HRMS** $[C_{31}H_{39}NO_2+H_3O]^+$ 476.3165, found: 476.3153.

(±,*Z*)-5-Methyl-6-phenylcyclooct-1-ene (**4x**)

In a nitrogen-filled glovebox to an oven-dried 1-dram vial containing a stir bar was added 9-methoxy-9-borabicyclo[3.3.1]nonane (62.3 mg, 2.05 equiv) and 1,4-dioxane (1 mL). Under stirring was added methylmagnesium bromide (133 μL, 3 M in THF). The resulting slurry was allowed to stir for 30 min before NaOMe (16.2 mg, 1.5 equiv) was added and the solution was stirred for 5-10 min. In the meantime, in a separate 1-dram vial was added in order $NiCl_2$(glyme) (6.6 mg, 15 mol%), dimethyl fumarate (13.0 mg, 45 mol%), 2-methyltetrahydrofuran (1 mL), and 1,5-cyclooctadiene (123 μL, 5 equiv). The solution was pre-stirred until homogeneous and then added to the first reaction vial. Finally, iodobenzene (41 mg, 0.2 mmol, 1 equiv) was added to the reaction and the vial was sealed and transferred

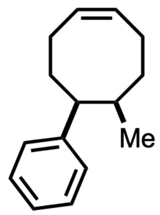

out of the glovebox to a heating block to stir at 35 °C and >1300 rpm for 24 h. The reaction was quenched with 1M HCl (aqueous) and diluted with Et_2O (5 mL). The aqueous phase was extracted with Et_2O (5 mL). The combined organic phases were washed with brine before dried over Na_2SO_4 and concentrated under reduced pressure. PTLC (hexanes) afforded the desired product as a colorless oil (19 mg, 48%).

Scale up: In a nitrogen-filled glovebox to an oven-dried 40-mL vial containing a x-shaped stir bar was added 9-methoxy-9-borabicyclo[3.3.1]nonane (**S3x**) (623 mg, 2.05 equiv) and 1,4-dioxane (10 mL). Under stirring was added methylmagnesium bromide (1.33 mL, 3 M in THF). The resulting slurry was allowed to stir for 30 min before NaOMe (162 mg, 1.5 equiv) was added and the solution was stirred for 5-10 min. In the meantime, in a separate 20-mL vial was added in order $NiCl_2$(glyme) (66 mg, 15 mol%), dimethyl fumarate (130 mg, 45 mol%), 2-methyltetrahydrofuran (10 mL), and 1,5-cyclooctadiene (1.23 mL, 5 equiv). The solution was pre-stirred until homogeneous and then added to the first reaction vial. Finally, iodobenzene (408 mg, 0.2 mmol, 1 equiv) was added to the reaction mixture and the vial was sealed and transferred out of the glovebox to an oil bath to stir at 35 °C and >1300 rpm for 48 h. The reaction was quenched with 1M HCl (aqueous) and diluted with Et_2O (10 mL). The aqueous phase was extracted with Et_2O (10 mL). The combined organic phases were washed with brine before dried over Na_2SO_4 and concentrated under reduced pressure. Silica gel column chromatography (hexanes) afforded the desired product as a colorless oil (201 mg, 50%). **^{1}H NMR** (400 MHz, $CDCl_3$) δ 7.30–7.26 (m, 3H), 7.24–7.13 (m, 2H), 5.78–5.70 (m, 1H), 5.65–5.55 (m, 1H), 3.06 (ddd, *J* = 12.7, 4.6, 2.5 Hz, 1H), 2.70–2.57 (m, 2H), 2.36–2.24 (m, 1H), 2.18–2.04 (m, 3H), 1.80–1.72 (m, 1H), 1.69–1.56 (m, 2H), 0.88 (d, *J* = 7.1 Hz, 3H). ^{13}C NMR (101 MHz, $CDCl_3$) δ 130.45, 128.62, 128.26, 127.99, 125.62, 45.97, 37.19, 33.67, 29.69, 26.67, 26.34, 16.52. Low resolution due to significant peak broadening hampered the assignment of carbon peaks. **HRMS** calcd. for $[C_{15}H_{21}]^+$ 201.1643, found: 201.1641.

General Information for the Ring-Opening Metathesis Polymerization (ROMP) of the 5,6-Arylalkylated Cyclooctenes (AACOE)

All reactions were carried out under a nitrogen atmosphere with dry solvents using anhydrous conditions unless otherwise stated. Dry, degassed dichloromethane (DCM) was obtained from a JC Meyer solvent purification system. 3rd Generation Grubbs catalyst (**G3**) was prepared according to a literature method[11]. Unless otherwise stated, all other reagents were purchased at the highest commercial quality and used without further purification. Yields refer to chromatographically and spectroscopically (^{1}H-NMR) homogeneous materials, unless otherwise stated. NMR spectra were recorded on Bruker Avance 400 or 500 MHz instruments and calibrated using residual undeuterated solvent as an internal reference ($CHCl_3$ at 7.26 ppm ^{1}H NMR and $CDCl_3$ at 77.16 ppm ^{13}C NMR) and analyzed by MestReNova. The following abbreviations (or combinations thereof) were used to explain the multiplicities: s = singlet, d = doublet, t = triplet, q = quartet, m = multiplet, br = broad. Mass spectra (MS) were recorded on time-of-flight matrix assisted laser desorption/ionization (MALDI-TOF) using a *trans*-2-[3-(4-tert-butylphenyl)-2-methyl-2-propenylidene]malononitrile (DCTB) matrix. Polymer samples were analyzed using a Tosoh EcoSEC HLC 8320GPC system with TSKgel SuperHZ-L columns eluting $CHCl_3$ containing 0.25% NEt_3 at a flow rate of 0.45 mL/min. All number-average molecular weights and dispersities were calculated from refractive index chromatograms using PStQuick Mp-M polystyrene standards. Thermogravimetric analyses (TGA) were performed under nitrogen atmosphere on a TGA2 STAR System (Mettler Toledo) at a heating rate of 10 °C/min. Differential scanning calorimetry (DSC) analyses were measured on a DSC Q200 (TA Instruments) under nitrogen atmosphere, and the reported data were obtained from the second or third heating cycle at a heating rate of 10 °C/min.

General Procedure 4: ROMP of (*Z*)-5,6-Arylalkylated Cyclooctenes (4)

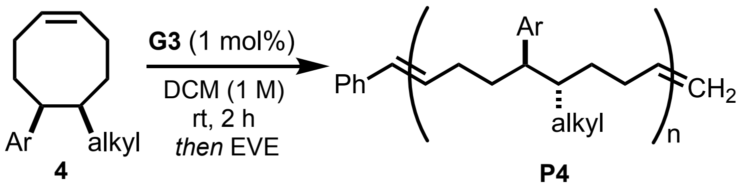

In a 4-mL vial equipped with a stirbar under nitrogen atmosphere was added Grubb's 3rd generation catalyst, **G3** (1 mol%). The vial was evacuated and backfilled with nitrogen 3 times. To this vial was then added a solution of the (*Z*)-5,6-diarylcyclooctene **4** (1 equiv) in CH_2Cl_2 (1 M). The reaction was allowed to proceed for 2 hours at room temperature under nitrogen, after which the catalyst was quenched with ethyl vinyl ether. The solvent was removed under vacuum to yield the crude polymer. Precipitation was accomplished by dissolving the crude polymer in chloroform and slowly adding it to a solution of methanol under vigorous stirring. The suspension was centrifuged and supernatant removed, followed by drying the collected powder to afford the precipitated polymer **P4**.

Characterization of Polymers

Preparation of polymer **P4a** from ROMP of monomer **4a**

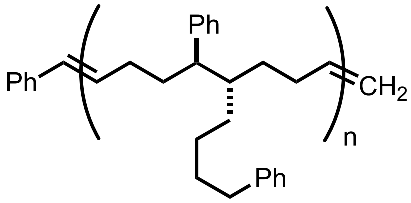

Following General Procedure 4 with **4a** (50 mg, 0.157 mmol) in CH_2Cl_2 (0.16 mL) and **G3** (1.14 mg, 0.00157 mmol). The crude polymer was dissolved in $CHCl_3$ (1 mL) and precipitated in MeOH (2 mL) under vigorous stirring. The precipitated polymer **P4a** was characterized using ^{1}H-NMR and SEC analyses (M_n: 20.2 kg/mol, *Đ*: 2.1). ^{1}**H NMR** (500 MHz, $CDCl_3$) δ 7.30–6.98 (m, 10H), 5.30–5.12 (m, 2H), 2.64–2.44 (m, 3H), 1.99–1.19 (m, 15H). ^{13}**C NMR** (126 MHz, $CDCl_3$) δ 144.21, 142.77, 130.53, 130.32, 130.06, 129.81, 129.49, 128.69, 128.39, 128.25, 127.95, 125.74, 125.61, 47.52, 42.65, 35.89, 35.85, 32.01, 31.84, 30.93, 30.74, 30.13, 29.87, 26.21, 25.51, 24.70.

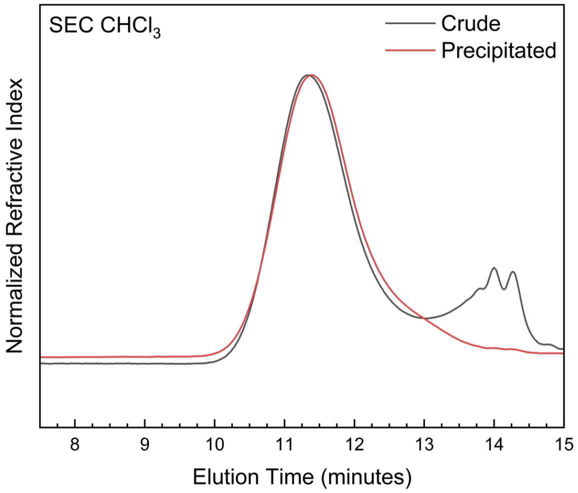

Figure S1. Crude and Precipitated SEC of **P4a**

Preparation of polymer **P4i** from ROMP of monomer **4i**

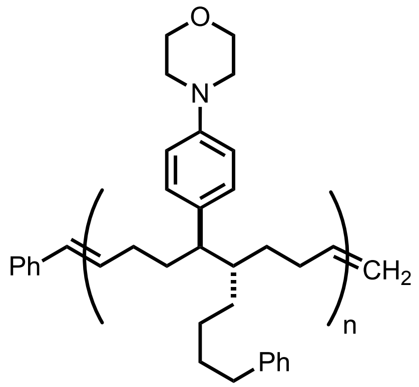

Following General Procedure 4 with **4i** (31.8 mg, 0.078 mmol) in CH_2Cl_2 (0.8 mL) and **G3** (0.57 mg, 0.00078 mmol). The crude polymer was dissolved in $CHCl_3$ (1 mL) and precipitated in MeOH (2 mL) under vigorous stirring. The precipitated polymer **P4i** was characterized using ^{1}H-NMR and SEC analyses (M_n: 30.5 kg/mol, *Đ*: 2.2). **^{1}H NMR** (500 MHz, $CDCl_3$) δ 7.24 (d, J = 7.4 Hz, 2H), 7.18–7.10 (m, 3H), 7.02– 6.87 (m, 2H), 6.86–6.70 (m, 2H), 5.20 (m, 2H), 3.88–3.76 (m, 4H), 3.15–3.02 (m, 4H), 2.59–2.52 (m, 2H), 2.50–2.36 (m, 1H), 1.95–1.38 (m, 9H), 1.34–1.20 (m, 5H), 0.98 (m, 1H). **^{13}C NMR** (126 MHz, $CDCl_3$) δ 149.13, 142.78, 130.51, 130.38, 130.13, 129.85, 129.32, 128.37, 128.25, 125.61, 115.32, 66.99, 49.56, 46.57, 42.76, 35.91, 35.87, 32.19, 31.86, 30.99, 30.71, 30.27, 29.94, 26.28, 25.57, 24.83.

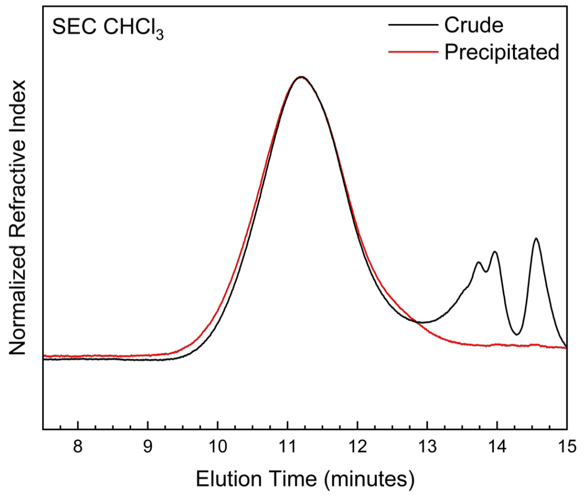

Figure S2. Crude and Precipitated SEC of **P4i**.

Preparation of polymer **P4n‘** from ROMP of monomer **4n‘**

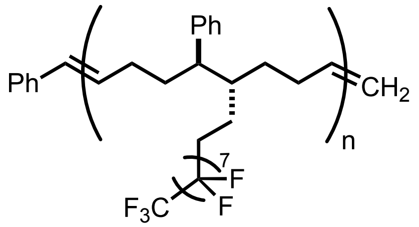

Following General Procedure 4 with **4n‘** (50.0 mg, 0.079 mmol) in CH_2Cl_2 (0.8 mL) and **G3** (0.58 mg, 0.00079 mmol). The crude polymer was dissolved in $CHCl_3$ (1 mL) and precipitated in MeOH (2 mL) under vigorous stirring. The precipitated polymer **P4n‘** was characterized using ^{1}H-NMR and SEC analyses (M_n: 37.5 kg/mol, *Đ*: 1.8). **^{1}H NMR** (500 MHz, $CDCl_3$) δ 7.26–7.13 (m, 3H), 7.08–6.98 (m, 2H), 5.30–5.08 (m, 2H), 2.54–2.38 (m, 1H), 2.02–1.48 (m, 11H), 1.35–1.24 (m, 1H), 1.05–0.96 (m, 1H). **^{13}C NMR** (126 MHz, $CDCl_3$) δ 143.06, 130.17, 129.65, 128.44, 128.18, 126.17, 118.81–117.78 (m, $J_{C\text{–}F}$), 117.74–115.57 (m, $J_{C\text{–}F}$), 111.50–109.91 (m, $J_{C\text{–}F}$), 47.30, 47.21, 41.84, 31.72, 30.59, 30.50, 30.26, 29.72, 27.98, 25.20, 24.30, 20.08.

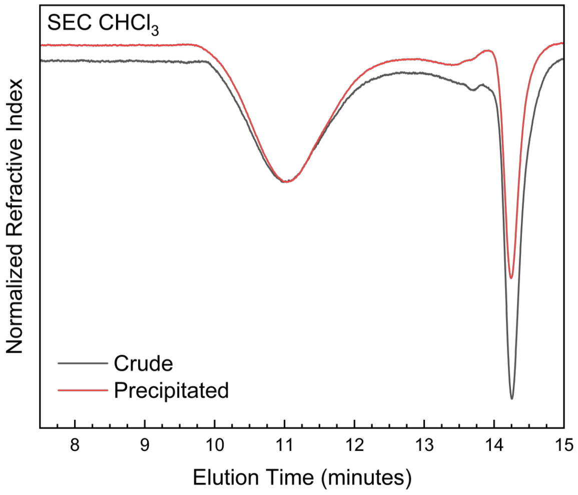

Figure S3 Crude and Precipitated SEC of **P4n'**

Preparation of polymer **P4s'** from ROMP of monomer **4s'**

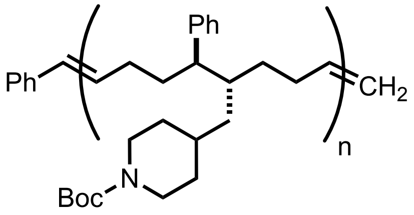

Following General Procedure 4 with **4s'** (50.0 mg, 0.135 mmol) in CH_2Cl_2 (0.14 mL) and **G3** (0.98 mg, 0.00135 mmol). The crude polymer was dissolved in $CHCl_3$ (1 mL) and precipitated in MeOH (2 mL) under vigorous stirring. The crude polymer was dissolved in $CHCl_3$ (1 mL) and precipitated in MeOH (2 mL) under vigorous stirring. The precipitated polymer **P4s'** was characterized using ^{1}H-NMR and SEC analyses (M_n: 46.3 kg/mol, $Đ$: 1.5). **^{1}H NMR** (500 MHz, $CDCl_3$) δ 7.25–7.11 (m, 3H), 7.10–6.97 (m, 2H), 5.32–5.13 (m, 2H), 4.01 (app bs, 2H), 2.72–2.41 (m, 3H), 1.94–1.29 (m, 20H), 1.21–0.81 (m, 5H). **^{13}C NMR** (126 MHz, $CDCl_3$) δ 154.82, 143.63, 143.50, 143.42, 130.40, 130.35, 130.24, 130.20, 130.06, 129.79, 129.54, 128.68, 127.97, 125.88, 79.18, 47.49, 44.01, 40.12, 39.90, 39.69, 37.76, 33.74, 32.82, 32.12, 31.49, 31.17, 31.03, 29.91, 29.85, 28.50, 25.56, 24.45.

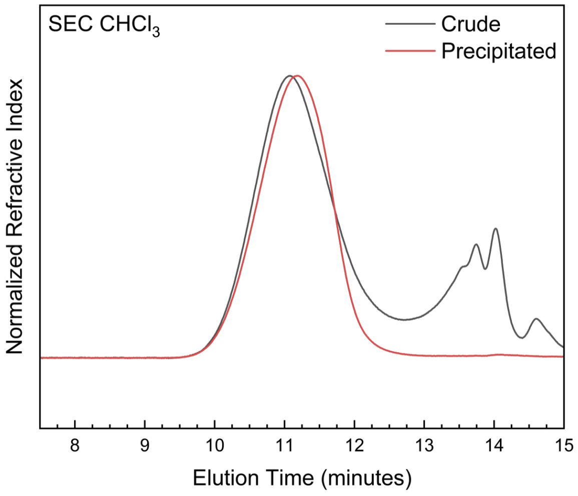

Figure S4 Crude and Precipitated SEC of **P4s'**

Preparation of polymer **P4x** from ROMP of monomer **4x**

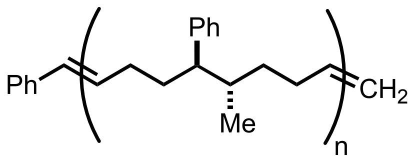

Following General Procedure 4 with **4x** (50.0 mg, 0.25 mmol) in CH_2Cl_2 (0.25 mL) and **G3** (1.81 mg, 0.0025 mmol). The crude polymer was dissolved in $CHCl_3$ (1 mL) and precipitated in MeOH (2 mL) under vigorous stirring. The precipitated polymer **P4x** was characterized using ^{1}H-NMR and SEC analyses (M_n: 24.2 kg/mol, *Đ*: 1.9). **^{1}H NMR** (500 MHz, $CDCl_3$) δ 7.25–7.21 (m, 2H), 7.19–7.13 (m, 1H), 7.11–6.99 (m, 2H), 5.28–5.12 (m, 2H), 2.41–2.28 (m, 1H), 2.01–1.91 (m, 1H), 1.87–1.47 (m, 5H), 1.37–1.20 (m, 2H), 0.99–0.78 (m, 4H). **^{13}C NMR** (126 MHz, $CDCl_3$) δ 144.46, 130.43, 130.26, 130.08, 128.60, 127.96, 125.72, 50.86, 37.89, 34.21, 31.70, 30.80, 30.22, 16.91.

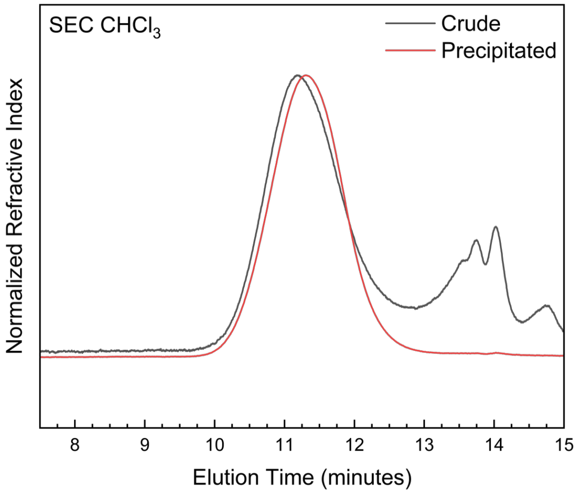

Figure S5 Crude and Precipitated SEC of **P4x**

Synthesis of **P4x-H2** by Reduction of **P4x**

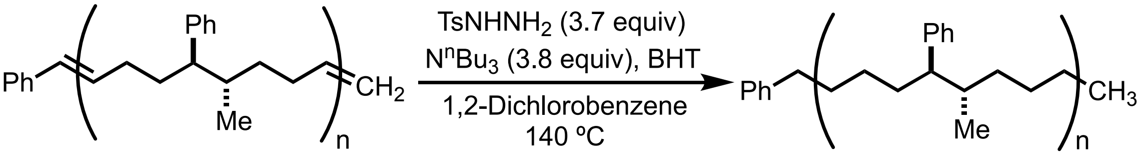

P4x **$P4x-H_2$**

The reduction of **P4x** (Mn: kg/mol, Đ:) with diimide was performed according to a literature method[12]. To a vial was added **P4x** (120 mg, 0.6 mmol), *p*-toluenesulfonylhydrazine (413 mg, 2.22 mmol), tributylamine (0.54 mL, 2.28 mmol), butylated hydroxytoluene (BHT) (20 mg, 0.09 mmol), and 1,2 dichlorobenzene (6 mL). This reaction mixture was heated under nitrogen at 140 °C overnight. The following morning the reaction mixture was precipitated into methanol (~15 mL). The polymer was isolated via centrifugation and redissolved in chloroform (~3 mL) and reprecipitated into methanol (~ 10 mL) to remove any remaining 1,2-dichlorobenzene. This polymer was again isolated via centrifugation and dried under vacuum. The reduced polymer **$P4x-H_2$** was characterized using NMR and SEC analyses (M_n:

22.3 kg/mol, Đ: 1.9). **^{1}H NMR** (500 MHz, $CDCl_3$) δ 7.32–7.23 (m, 2H), 7.22–7.15 (m, 1H), 7.15–7.06 (m, 2H), 2.47–2.30 (m, 1H), 1.71–1.47 (m, 3H), 1.34– 0.71 (m, 13H). **^{13}C NMR** (126 MHz, $CDCl_3$) δ 146.42, 145.07, 145.05, 128.53, 128.13, 127.87, 127.66, 125.67, 125.56, 51.55, 46.06, 38.49, 36.99, 34.33, 31.76, 31.71, 29.86, 29.81, 29.77, 29.73, 29.71, 29.69, 27.85, 27.61, 27.15, 17.04.

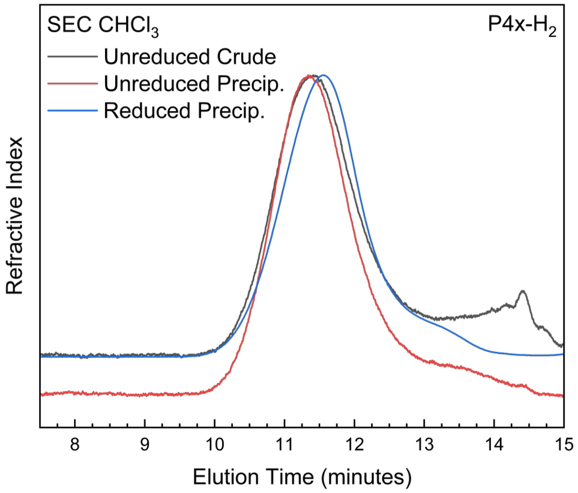

Figure S6. SEC traces of crude reaction mixture and purified polymer **P4x-H₂**.

Synthesis of **P4s'-TFA** from **P4s'**

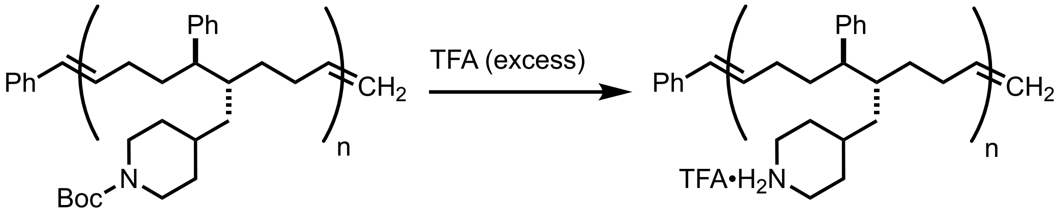

To a 4-mL vial equipped with a stir bar was added **P4s'** (50 mg, 0.168 mmol) before purged with argon 3 times. Then, the polymer was dissolved in a mixture of CH_2Cl_2 (1 mL) and TFA (1mL, 13 mmol). This mixture was allowed to stir overnight before the solution was dried under vacuum. The TFA salt of the polymer was collected and used without further purification. The sample was not analyzed by GPC because the polymer was insoluble in chloroform. Partial deprotection of the Boc group was observed by ^{1}H NMR. **^{1}H NMR** (500 MHz, CD_3OD) δ 7.33–7.00 (m, 5H), 5.37–5.13 (m, 2H), 3.29–3.20 (m, 2H), 2.99–2.78 (m, 2H), 2.75–2.42 (s, 1H), 1.97–1.47 (m, 12H), 1.19–1.01 (m, 3H). Remaining Boc-group observed in the spectrum at ^{1}H NMR (500 MHz, CD_3OD) δ 1.29 (s) ppm.

Kinetic Studies for the ROMP of AACOE

Kinetic experiments were conducted in a sealed NMR tube. To this was added **G3** (0.51 mg, 0.0007 mmol). The tube was purged 3 times with nitrogen before the corresponding monomer **4** (0.07 mmol) dissolved in $CDCl_3$ (0.7 mL) was added to the tube. The reaction was monitored by ^{1}H NMR to determine the conversion of monomer.

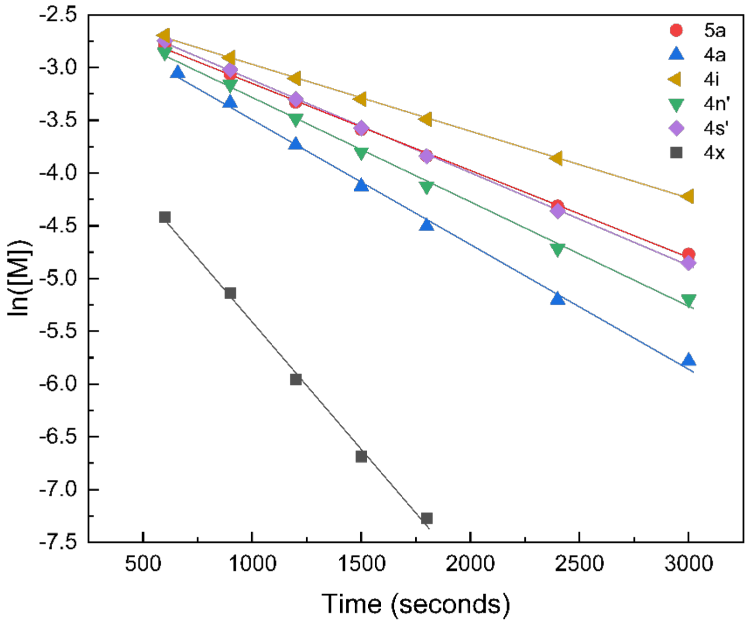

**5a**	8.25×10^{-4}
**4a**	11.8×10^{-4}
**4i**	6.34×10^{-4}
**4n'**	9.90×10^{-4}
**4s'**	8.79×10^{-4}
**4x**	24.2×10^{-4}

Figure S7. First-Order Kinetics Plots for ROMP of AACOE and Observed Rate Constants.

Thermal Properties of the Polymers P4

TGA curves of purified **P4a, P4i, P4n‘, P4s‘, P4x,** and **P4x-H₂** samples were obtained in a nitrogen atmosphere at a heating rate of 10 °C/min.

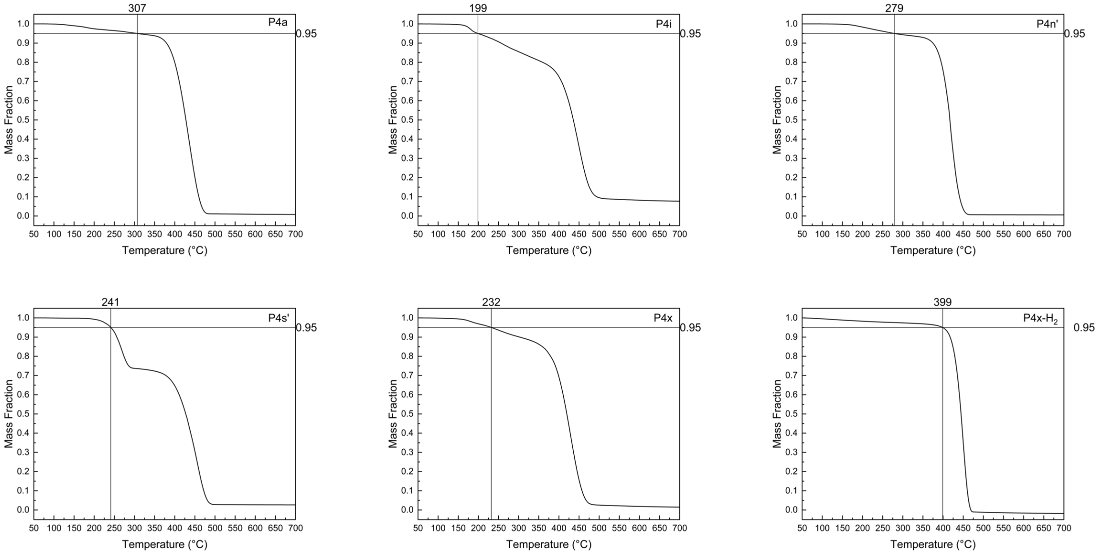

Figure S8. TGA of **P4a, P4i, P4n‘, P4s‘, P4x** and **P4x-H₂**. Temperatures at 95% of initial sample weight (T95) were displayed.

DSC analysis of purified **P4a, P4i, P4n‘, P4s‘, P4x** and **P4x-H₂** samples were performed under nitrogen flow (-80 °C to 1200 °C, heating rate: 10 °C/min, cooling rate: 10 °C/min).

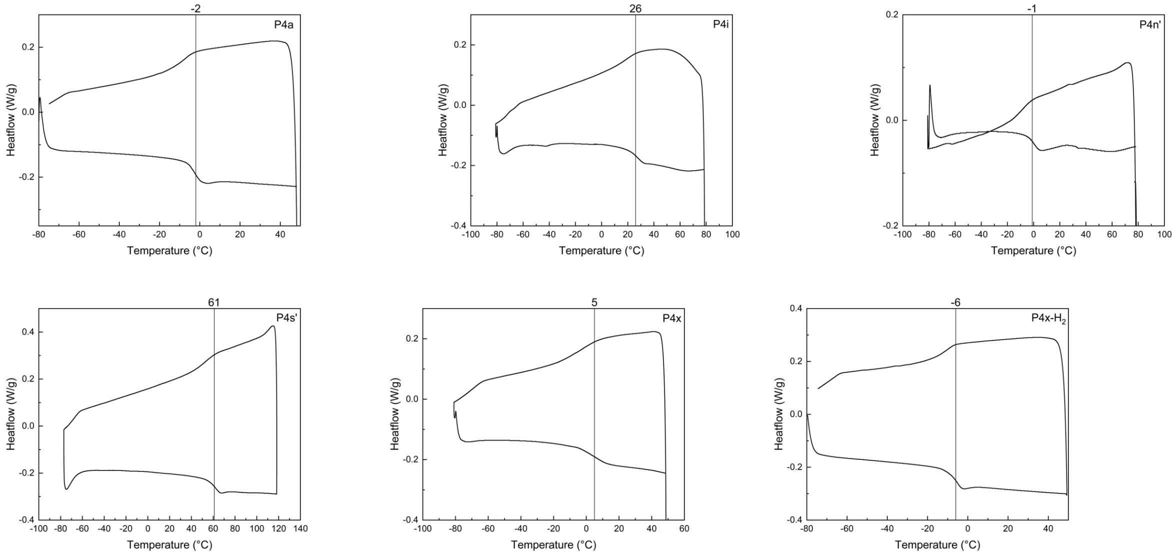

Figure S9. (A) DSC of **P4a, P4i, P4n‘, P4s‘, P4x** and **P4x-H₂**; Glass transition temperatures (T_g) are displayed.

Contact Angle Measurements of Polymers

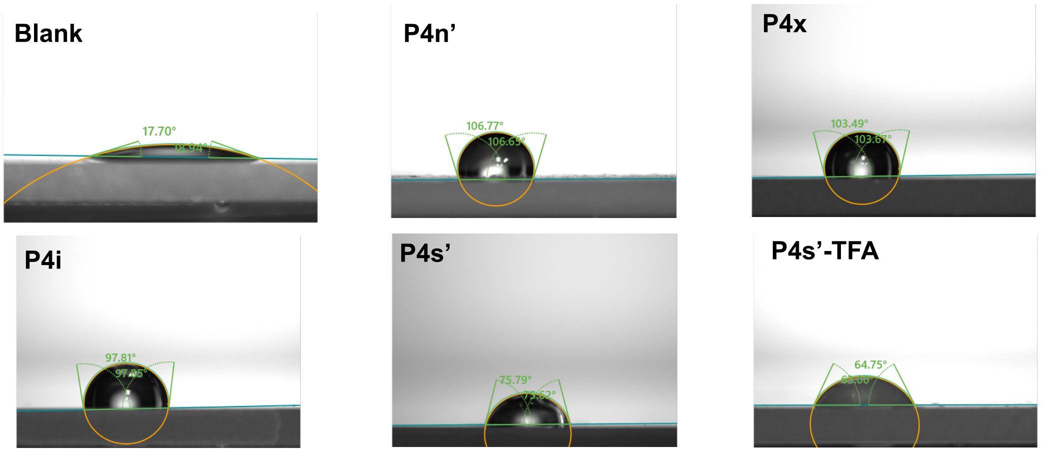

Figure S10. Contact angle measurements of polymers **P4n’**, **P4x**, **P4i**, **P4s’**, and **P4s’-TFA**.

Computational Details

All density functional theory (DFT) calculations were carried out using the Gaussian 16 program[13]. Geometries of intermediates and transition states were optimized using the dispersion-corrected B3LYP-D3[14] functional with a mixed basis set of SDD for Ni[15], and I[16], and 6-31G(d) for other atoms in the gas phase. Vibrational frequency calculations were performed for all stationary points to confirm if each optimized structure is a local minimum or a transition state structure. All optimized transition state structures have only one imaginary (negative) frequency, and all minima (reactants, products, and intermediates) have no imaginary frequencies. The M06 functional[17] with a mixed basis set of SDD for Ni, I and 6-311+G(d,p) for other atoms was used for single-point energy calculations in solution using the SMD continuum solvation model[18]. The 1,4-dioxane solvent was used in single-point energy calculations. Considering the similar dielectric constants for 1,4-dioxane (2.21) and 2-MeTHF (6.97), single-point energies computed in 2-MeTHF are expected to be similar to those computed in 1,4-dioxane. Gibbs free energies were calculated at 308.15 K and a concentration of 1 M. Quasiharmonic approximation from Grimme was applied for vibrational entropy calculations using 100 cm^{-1} as the frequency cut-off[19]. Quasiharmonic approximations and translation entropy calculations were computed using GoodVibes[20]. The 3D images of optimized structures were prepared using CYLView[21]

Reaction energy profiles of the Ni-catalyzed alkylarylation of COD

DFT calculations were performed at the M06/6-311+G(d,p)–SDD(Ni,I)/SMD(1,4-dioxane)//B3LYP-D3/6-31G(d)–SDD(Ni,I) level of theory.

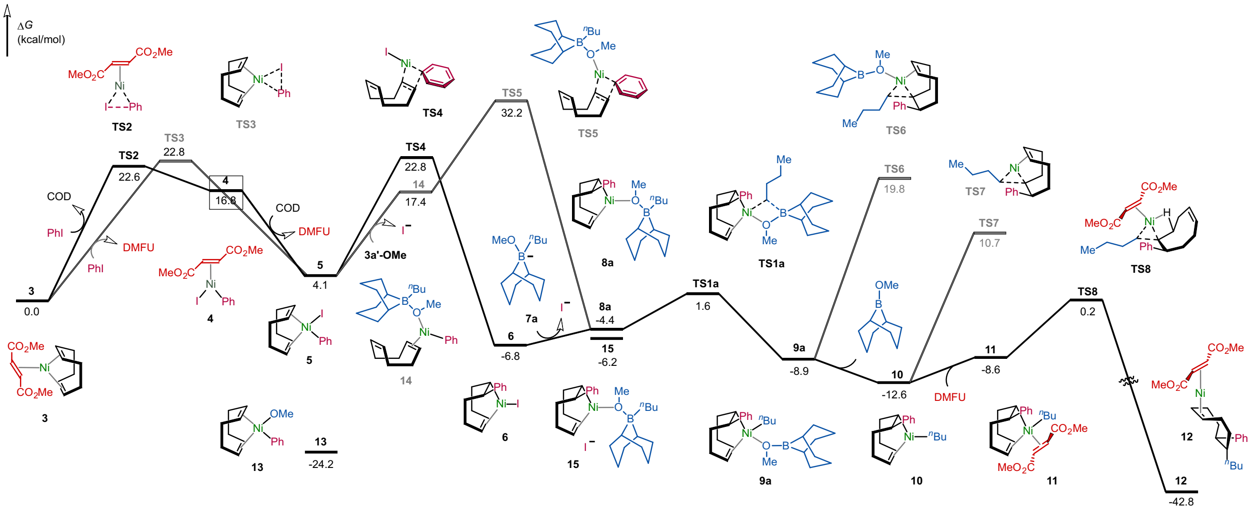

Figure S11. Reaction energy profiles of the Ni-catalyzed alkylarylation of COD.

Conformations of 5-*n*-butyl-6-phenylcyclooctene monomer

DFT calculations were performed at the M06/6-311+G(d,p)/SMD(1,4-dioxane)//B3LYP-D3/6-31G(d) level of theory. The boat-chair conformer of **a** is 0.5 kcal/mol less stable than the half-chair-boat conformer **b**.

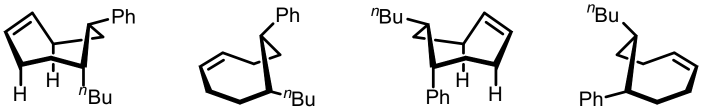

	**a**	**b**	**c**	**d**
	boat–chair	half-chair–boat	boat–chair	half-chair–boat
Δ*G* (kcal/mol)	0.5	0.0	3.8	0.2

Figure S12. Relative stability of low-energy conformers of 5-*n*-butyl-6-phenylcyclooctene. All energies are Gibbs free energies (in kcal/mol) with respect to **b**.

DFT studies of the reactivity of different boryl reagents in transmetalation

DFT calculations were performed at the M06/6-311+G(d,p)–SDD/SMD(1,4-dioxane)//B3LYP-D3/6-31G(d)–SDD level of theory. Thermal corrections were calculated at 308.15 K using GoodVibes. Alkyl 9-BBN borate (**7a**) is the most reactive boryl reagent. In addition, other borate complexes have two binding sites, forming stable four-coordinated intermediates, which may inhibit the reaction.

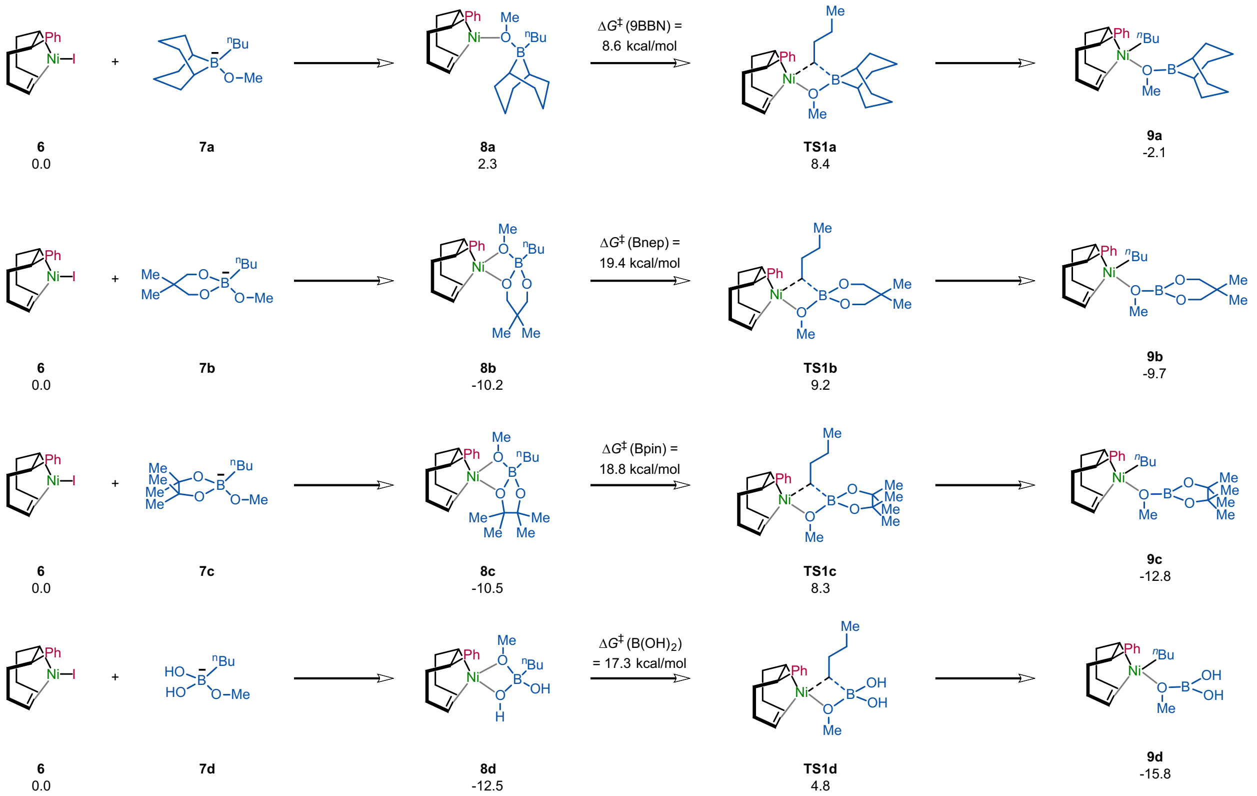

Figure S13. Comparison of transmetalation with different boron reagents.

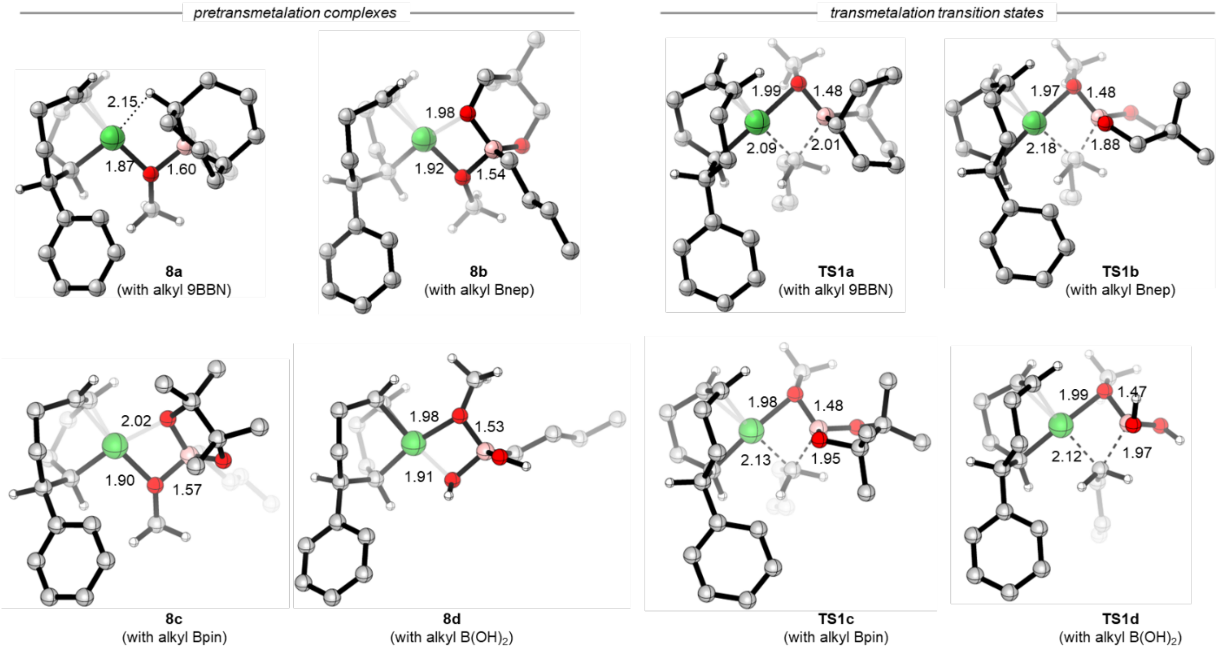

Figure S14. Structures of key intermediates and transition states.

Cartesian coordinates (Å) and energies of optimized structures

```
10
B3LYP-D3 SCF energy:               -872.529245797 a.u.
B3LYP-D3 enthalpy:                 -872.110537 a.u.
B3LYP-D3 free energy:              -872.180718 a.u.
M06 SCF energy in solution:        -872.118095963 a.u.
M06 enthalpy in solution:          -871.699387166 a.u.
M06 free energy in solution:       -871.769568166 a.u.

Cartesian coordinates
ATOM  X      Y      Z
C    -0.066041    -1.461794    -1.535988
C    -0.995230    -1.765810    1.988807
H    0.574780    -2.013524    -2.236582
H    -0.017454    -0.406150    -1.830299
H    -0.327454    -2.524812    2.429421
H    -1.375151    -1.158599    2.819610
C    -2.569688    -1.222988    -0.904662
C    -2.923308    -1.488985    0.400916
C    -1.510806    -1.973117    -1.699292
C    -2.184478    -2.469128    1.288545
H    -3.251168    -0.599538    -1.490556
H    -3.851702    -1.065552    0.787469
H    -1.538531    -3.038507    -1.431912
H    -1.776792    -1.916425    -2.761303
H    -1.805148    -3.301143    0.682603
H    -2.864147    -2.902292    2.030242
C    2.001361    -1.249072    -0.132896
C    2.987867    -2.201125    0.150484
C    2.408754    0.058773    -0.440052
C    4.343443    -1.862422    0.130369
H    2.691508    -3.220088    0.391074
C    3.760020    0.401749    -0.461915
H    1.656282    0.811745    -0.654887
C    4.734728    -0.558780    -0.176775
H    5.091791    -2.618359    0.355087
H    4.053400    1.421159    -0.700169
H    5.788134    -0.291903    -0.192874
C    0.527461    -1.618921    -0.110624
H    0.468665    -2.693575    0.124081
C    -0.225328    -0.854470    1.023590
H    0.483009    -0.244981    1.589323
Ni    -1.634030    0.229003    0.316259
C    -0.830173    1.886178    0.776236
H    0.204202    2.028726    0.438264
H    -0.834723    2.011156    1.871244
C    -1.772836    2.899487    0.106810
H    -1.712010    2.804597    -0.988762
H    -2.825170    2.688606    0.371860
C    -1.483631    4.365868    0.485419
H    -0.441839    4.595558    0.222782
H    -1.556050    4.468370    1.576884
C    -2.425989    5.363229    -0.196268
```

H	-2.195721	6.396084	0.090732
H	-2.348974	5.294757	-1.288831
H	-3.471138	5.166307	0.074843

11

B3LYP-D3 SCF energy: -1406.90881132 a.u.
B3LYP-D3 enthalpy: -1406.335179 a.u.
B3LYP-D3 free energy: -1406.429745 a.u.
M06 SCF energy in solution: -1406.33423581 a.u.
M06 enthalpy in solution: -1405.76060349 a.u.
M06 free energy in solution: -1405.85516949 a.u.

Cartesian coordinates

ATOM	X	Y	Z
Ni	0.887104	-0.053845	-0.325629
C	-1.056249	-2.850121	-1.303096
C	0.574472	-0.519091	-2.849991
H	-0.571800	-3.311332	-2.167027
H	-1.930349	-3.479898	-1.099116
H	0.042104	-0.621634	-3.809781
H	1.174419	0.392299	-2.938373
C	1.229373	-2.236315	-0.172225
C	1.998747	-1.859184	-1.244519
C	-0.105806	-2.945345	-0.102449
C	1.543381	-1.728719	-2.680137
H	1.759272	-2.259348	0.784447
H	3.042185	-1.641745	-1.037964
H	0.113980	-4.011609	0.068320
H	-0.608665	-2.605719	0.809270
H	1.066784	-2.650847	-3.024206
H	2.415549	-1.572765	-3.322917
C	-2.837964	-0.987147	-1.021752
C	-3.594669	0.027270	-1.631645
C	-3.335239	-1.542690	0.163038
C	-4.780804	0.493002	-1.067424
H	-3.241741	0.460493	-2.565480
C	-4.524293	-1.081789	0.735719
H	-2.794009	-2.340011	0.661456
C	-5.249973	-0.057670	0.128232
H	-5.341333	1.281801	-1.562496
H	-4.879381	-1.526068	1.661992
H	-6.172354	0.302632	0.575103
C	-1.547934	-1.435842	-1.707885
H	-1.835290	-1.539302	-2.768316
C	-0.453017	-0.342433	-1.718190
H	-0.931073	0.636840	-1.824354
C	1.406249	2.638111	-1.221646
C	1.734714	1.815250	-0.040711
C	2.775231	0.881968	-0.109243
C	3.318021	0.273018	1.110523
H	1.398567	2.179767	0.922978
H	3.332730	0.761038	-1.031869
O	0.519021	3.611949	-0.910074
O	1.847056	2.480404	-2.349588
O	2.905773	0.437090	2.248062
O	4.346520	-0.573254	0.816447
C	0.080613	4.418479	-2.012963

H	-0.619981	5.138074	-1.588266
H	0.927630	4.931057	-2.477872
H	-0.414671	3.799196	-2.767030
C	4.905056	-1.263073	1.943985
H	5.710225	-1.879362	1.542002
H	5.294143	-0.552258	2.678470
H	4.148307	-1.888140	2.428518
C	-0.510469	0.299454	0.975160
H	-1.107518	-0.606586	1.086857
H	0.193772	0.363300	1.822758
C	-1.419302	1.514422	0.907168
H	-2.062706	1.443726	0.023987
H	-0.839422	2.435833	0.790543
C	-2.318305	1.639743	2.151989
H	-2.864309	0.698605	2.295857
H	-1.688985	1.779082	3.042658
C	-3.319798	2.793308	2.032537
H	-3.990244	2.631350	1.179732
H	-3.937849	2.886877	2.933635
H	-2.804595	3.750054	1.878317

12

B3LYP-D3 SCF energy: -1406.95192251 a.u.
B3LYP-D3 enthalpy: -1406.376072 a.u.
B3LYP-D3 free energy: -1406.473723 a.u.
M06 SCF energy in solution: -1406.38958231 a.u.
M06 enthalpy in solution: -1405.81373180 a.u.
M06 free energy in solution: -1405.91138280 a.u.

Cartesian coordinates

ATOM	X	Y	Z
C	-4.328187	-0.681231	1.842011
C	-3.302196	-0.121770	2.620811
C	-5.510048	-1.062327	2.494404
C	-3.453931	0.041502	3.999412
H	-2.380902	0.209461	2.156495
C	-5.667506	-0.899350	3.871898
H	-6.320188	-1.495425	1.911849
C	-4.636279	-0.346712	4.632849
H	-2.641760	0.474524	4.578547
H	-6.596210	-1.202972	4.348323
H	-4.753181	-0.217361	5.705507
C	-3.760450	0.376597	-0.400561
C	-3.644896	0.243943	-1.932675
C	-1.780963	-1.852094	0.137311
C	-2.332722	-0.165858	-2.596827
C	-1.005831	-2.027299	-1.167878
C	-1.300564	-1.061790	-2.301157
H	-2.819599	0.762861	-0.000711
H	-4.434600	-0.432273	-2.299344
H	-1.589599	-0.841578	0.508274
H	0.072351	-1.960051	-0.968939
H	-4.510717	1.146521	-0.181330
H	-3.883490	1.223508	-2.364177
H	-1.354245	-2.525748	0.889644
H	-2.339931	0.135635	-3.650097
H	-1.177325	-3.044517	-1.555771

H	-0.669141	-1.285159	-3.169893
C	-3.298384	-2.125302	-0.023674
H	-3.485034	-2.306228	-1.092423
C	-4.189943	-0.905651	0.341781
H	-5.194955	-1.155976	-0.029057
Ni	-0.679217	0.803276	-1.863223
C	-0.900521	2.700375	0.185191
C	0.088230	1.702954	-0.286962
C	0.858264	1.974330	-1.435693
C	2.059082	1.160717	-1.718705
H	0.422460	0.959817	0.434143
H	0.763985	2.929168	-1.949194
O	-1.424687	2.331143	1.382350
O	-1.245117	3.706561	-0.407662
O	2.426693	0.180449	-1.091977
O	2.710549	1.623091	-2.815497
C	-2.472015	3.181596	1.880618
H	-2.812010	2.715798	2.804910
H	-2.090455	4.189955	2.062079
H	-3.292076	3.233721	1.158952
C	3.866160	0.864498	-3.200980
H	4.267128	1.367061	-4.081990
H	4.605874	0.851164	-2.395168
H	3.588389	-0.166929	-3.439364
C	-3.745396	-3.399094	0.716117
H	-3.497692	-3.305354	1.781971
H	-4.841646	-3.476247	0.667671
C	-3.135539	-4.690158	0.156586
H	-2.038171	-4.637936	0.199348
H	-3.393605	-4.783288	-0.909438
C	-3.601101	-5.947146	0.901945
H	-4.698035	-6.007074	0.859694
H	-3.342242	-5.851231	1.965908
C	-2.993112	-7.235520	0.338201
H	-1.897765	-7.213205	0.398468
H	-3.340127	-8.118533	0.887057
H	-3.262906	-7.369805	-0.716870

13

B3LYP-D3 SCF energy: -829.805096552 a.u.
B3LYP-D3 enthalpy: -829.470344 a.u.
B3LYP-D3 free energy: -829.533325 a.u.
M06 SCF energy in solution: -829.455494832 a.u.
M06 enthalpy in solution: -829.120742280 a.u.
M06 free energy in solution: -829.183723280 a.u.

Cartesian coordinates

ATOM	X	Y	Z
Ni	-0.466331	0.246273	0.226531
C	-1.222576	2.115565	-0.445945
C	-1.200281	2.059681	0.937792
C	-2.346027	1.754275	1.881620
C	-2.451432	1.962240	-1.333710
H	-0.352129	2.559301	-0.924360
H	-0.332699	2.496049	1.429460
H	-2.833698	2.694107	2.184106
H	-1.907255	1.333365	2.794937

```
H    -3.323984    2.388089    -0.830221
H    -2.303316    2.563727    -2.237428
C    -2.763705    -0.417986    0.647315
C    -2.502332    -0.529799    -0.679085
C    -3.404293    0.762975    1.340673
C    -2.734888    0.501964    -1.762042
H    -2.516163    -1.274695    1.269927
H    -2.099490    -1.480762    -1.015976
H    -4.098501    1.272413    0.667920
H    -4.004079    0.401924    2.183877
H    -3.759558    0.423699    -2.154722
H    -2.070001    0.248467    -2.595661
C    1.320259    0.836435    0.258068
C    2.005694    1.043697    -0.945704
C    1.996295    1.055238    1.464849
C    3.340547    1.464172    -0.943638
H    1.501508    0.865314    -1.893557
C    3.330051    1.477200    1.469976
H    1.484479    0.881458    2.409547
C    4.005838    1.683090    0.264768
H    3.859731    1.617762    -1.887346
H    3.841285    1.640857    2.416306
H    5.042503    2.010091    0.267464
O    -0.021348    -1.486781    0.336871
C    1.250400    -2.068285    0.444427
H    1.787602    -1.752512    1.352238
H    1.125206    -3.161568    0.487348
H    1.897358    -1.833359    -0.415052
```

14

```
B3LYP-D3 SCF energy:                 -1325.86066357 a.u.
B3LYP-D3 enthalpy:                   -1325.173031 a.u.
B3LYP-D3 free energy:                -1325.269329 a.u.
M06 SCF energy in solution:          -1325.20157831 a.u.
M06 enthalpy in solution:            -1324.51394574 a.u.
M06 free energy in solution:         -1324.61024374 a.u.
```

```
Cartesian coordinates
ATOM  X      Y      Z
Ni    -0.943533    1.576427    1.961666
C    0.379116    1.998246    0.726131
C    1.714603    1.632350    0.915123
C    -0.012129    2.596737    -0.477509
C    2.664144    1.907180    -0.076704
H    2.020050    1.128486    1.828058
C    0.939017    2.871557    -1.465564
H    -1.058063    2.830705    -0.657540
C    2.279601    2.533227    -1.264115
H    3.702366    1.624514    0.080363
H    0.627093    3.341984    -2.394963
H    3.017662    2.745933    -2.032717
O    -1.359556    -0.050930    1.177949
C    -0.683135    -0.759309    0.145797
H    -1.293944    -1.611876    -0.157202
H    -0.498168    -0.123189    -0.723496
H    0.277854    -1.114767    0.531966
C    1.047663    2.991308    3.874289
```

C	1.348922	4.111271	4.887623
C	-0.972137	4.753676	1.901821
C	1.091151	5.531461	4.450439
C	0.447612	5.348574	1.945977
C	0.749976	6.041813	3.259879
H	1.603966	3.164136	2.956585
H	0.780749	3.926111	5.811869
H	-1.226960	4.488880	0.869366
H	1.176552	4.577452	1.689784
H	1.417899	2.043693	4.283838
H	2.406396	4.027963	5.182804
H	-1.691638	5.533001	2.191546
H	1.226220	6.250337	5.260120
H	0.513265	6.091503	1.142787
H	0.620971	7.123971	3.229214
C	-1.262754	3.565520	2.786838
H	-2.333267	3.415334	2.943954
C	-0.431866	2.827494	3.607775
H	-0.938486	2.211157	4.355929
B	-2.949000	-0.063898	1.421710
C	-3.596168	1.080132	0.443798
H	-3.197509	2.080462	0.712728
H	-4.677095	1.168329	0.626005
C	-3.385345	0.887752	-1.064727
H	-2.311969	0.822336	-1.285390
H	-3.814919	-0.075850	-1.376761
C	-3.981539	2.005952	-1.929544
H	-5.062437	2.074422	-1.740204
H	-3.558092	2.970431	-1.607190
C	-3.727838	1.812997	-3.428393
H	-4.159787	2.625983	-4.024352
H	-2.651427	1.776331	-3.641706
H	-4.164163	0.870419	-3.782123
C	-3.084436	0.285609	3.030895
H	-2.690241	1.288830	3.317856
C	-3.550979	-1.566909	1.272388
H	-3.484410	-1.930831	0.233576
C	-2.309336	-0.733094	3.902410
H	-2.447567	-0.510363	4.972843
H	-1.235193	-0.607675	3.697942
C	-4.595487	0.380890	3.364290
H	-4.740065	0.568625	4.439902
H	-5.002617	1.258671	2.846388
C	-2.778318	-2.580147	2.151783
H	-3.229188	-3.581952	2.071236
H	-1.758321	-2.678221	1.755964
C	-5.065030	-1.496696	1.599680
H	-5.555124	-0.917460	0.806041
H	-5.513085	-2.501872	1.556967
C	-2.672060	-2.209673	3.645992
H	-3.613288	-2.444936	4.151714
H	-1.917378	-2.849969	4.124078
C	-5.422770	-0.865511	2.967653
H	-6.487653	-0.594344	2.968959
H	-5.318699	-1.622680	3.750571

15

B3LYP-D3 SCF energy: -1337.44552625 a.u.
B3LYP-D3 enthalpy: -1336.753571 a.u.
B3LYP-D3 free energy: -1336.851267 a.u.
M06 SCF energy in solution: -1336.79165762 a.u.
M06 enthalpy in solution: -1336.09970237 a.u.
M06 free energy in solution: -1336.19739837 a.u.

Cartesian coordinates

ATOM	X	Y	Z
C	2.440998	-0.945796	-1.685238
C	0.041608	-2.736877	0.394160
H	3.531008	-0.977648	-1.821904
H	2.126043	0.082506	-1.883367
H	0.670858	-3.281677	1.118624
H	-0.955117	-2.630809	0.830889
C	0.265349	-1.843327	-2.758992
C	-0.583662	-2.641043	-2.017877
C	1.784581	-1.888852	-2.710680
C	-0.103414	-3.551985	-0.909903
H	-0.161966	-1.301525	-3.605085
H	-1.641574	-2.688769	-2.269947
H	2.118694	-2.916229	-2.510170
H	2.160510	-1.624925	-3.706779
H	0.861116	-4.000096	-1.185918
H	-0.822914	-4.362135	-0.761130
C	2.885324	-0.327334	0.689680
C	4.137782	-0.676678	1.209820
C	2.378739	0.950935	0.974829
C	4.870059	0.220298	1.992007
H	4.543625	-1.665101	1.001077
C	3.110076	1.851295	1.749736
H	1.409772	1.239531	0.578462
C	4.358866	1.490715	2.263203
H	5.837940	-0.075903	2.390675
H	2.699653	2.838094	1.950159
H	4.926185	2.191869	2.870705
C	2.120442	-1.291433	-0.206936
H	2.530212	-2.300218	-0.033705
C	0.609884	-1.338419	0.138101
H	0.408038	-0.713896	1.012106
Ni	-0.528606	-0.601849	-1.226464
B	-1.125916	1.954061	-1.883502
O	-0.673394	1.179377	-0.538445
C	-1.491813	1.261023	0.637711
H	-1.809702	2.294334	0.797140
H	-2.370715	0.612714	0.568002
H	-0.888617	0.941521	1.495023
C	-2.301497	1.022012	-2.575469
H	-2.319073	1.207155	-3.660266
H	-2.063826	-0.073918	-2.534244
C	-3.738229	1.146309	-2.044832
H	-4.030457	2.206835	-2.035748
H	-3.785111	0.800085	-1.007105
C	-4.765330	0.348790	-2.856647
H	-4.755430	0.700396	-3.901130
H	-4.458429	-0.705125	-2.865872
C	-6.180978	0.443609	-2.280231
H	-6.517125	1.488533	-2.219173

H	-6.906377	-0.107611	-2.892727
H	-6.200660	0.015586	-1.271510
C	-1.543911	3.492847	-1.541339
H	-2.438083	3.547435	-0.899504
C	0.215982	2.088964	-2.825699
H	0.606781	1.098569	-3.122653
C	-1.937290	4.193955	-2.866375
H	-2.218991	5.244133	-2.679523
H	-2.842930	3.703725	-3.249368
C	-0.403003	4.206019	-0.774138
H	-0.306509	3.728969	0.210924
H	-0.666574	5.258721	-0.573907
C	-0.154651	2.804876	-4.148792
H	-0.809285	2.142500	-4.730700
H	0.745620	2.954451	-4.768834
C	1.356801	2.801229	-2.066727
H	1.683850	2.144593	-1.256305
H	2.236790	2.937580	-2.719125
C	-0.864726	4.166879	-3.978046
H	-0.117432	4.941836	-3.778494
H	-1.329980	4.453193	-4.933466
C	0.984572	4.168329	-1.452724
H	1.033152	4.942176	-2.226253
H	1.752310	4.448649	-0.715214
I	-3.814619	-2.267873	0.013323

3

B3LYP-D3 SCF energy: -1017.42379574 a.u.
B3LYP-D3 enthalpy: -1017.078660 a.u.
B3LYP-D3 free energy: -1017.150968 a.u.
M06 SCF energy in solution: -1017.09950468 a.u.
M06 enthalpy in solution: -1016.75436894 a.u.
M06 free energy in solution: -1016.82667694 a.u.

Cartesian coordinates

ATOM	X	Y	Z
C	-1.521223	-1.681949	0.410393
C	-0.703733	-1.519718	1.505300
C	0.532081	-2.321856	1.862499
C	-1.398776	-2.762562	-0.649799
H	-2.433143	-1.085644	0.385832
H	-1.044202	-0.832838	2.280241
H	0.257406	-3.204464	2.460035
H	1.146213	-1.688989	2.513348
H	-0.995632	-3.677282	-0.207427
H	-2.399583	-3.016545	-1.016031
C	1.518993	-1.684111	-0.410461
C	0.701897	-1.520424	-1.505410
C	1.394887	-2.764927	0.649339
C	-0.535179	-2.320506	-1.862805
H	2.431712	-1.089039	-0.385504
H	1.043292	-0.833615	-2.280000
H	0.990211	-3.678811	0.206647
H	2.395317	-3.020691	1.015368
H	-0.261950	-3.203415	-2.460562
H	-1.148281	-1.686488	-2.513501
Ni	-0.000138	-0.223987	0.000150

C	-1.909822	1.740271	-0.179267
C	-0.573860	1.611095	0.419933
C	0.576052	1.610348	-0.419682
C	1.912182	1.738239	0.179379
H	-0.478592	1.822030	1.482298
H	0.480860	1.821381	-1.482040
O	-2.880688	1.741439	0.780818
O	-2.172499	1.785595	-1.371360
O	2.174979	1.783085	1.371477
O	2.882941	1.738752	-0.780761
C	-4.222659	1.803270	0.281992
H	-4.865110	1.814694	1.163856
H	-4.375086	2.707069	-0.315319
H	-4.446564	0.932321	-0.342954
C	4.225029	1.799401	-0.282070
H	4.867365	1.811076	-1.164012
H	4.378076	2.702649	0.315905
H	4.448477	0.927837	0.342184

4

B3LYP-D3 SCF energy: -948.430695688 a.u.
B3LYP-D3 enthalpy: -948.177746 a.u.
B3LYP-D3 free energy: -948.250374 a.u.
M06 SCF energy in solution: -948.117837888 a.u.
M06 enthalpy in solution: -947.864888200 a.u.
M06 free energy in solution: -947.937516200 a.u.

Cartesian coordinates

ATOM	X	Y	Z
C	2.662745	1.106892	-0.496875
C	1.316833	1.606808	-0.824335
C	0.591303	2.233186	0.180039
C	-0.739373	2.824866	-0.148798
H	0.936859	1.570744	-1.839870
H	1.053270	2.537001	1.117482
O	3.537383	1.066415	-1.487655
O	2.869044	0.680171	0.647057
O	-1.204143	2.890506	-1.266267
O	-1.345028	3.267317	0.966709
C	4.806790	0.434077	-1.182356
H	5.361464	0.443718	-2.119381
H	5.331220	1.003385	-0.411412
H	4.636853	-0.586798	-0.832465
C	-2.691736	3.742356	0.776774
H	-3.024510	4.073391	1.760429
H	-2.707825	4.568539	0.061082
H	-3.317494	2.925537	0.406714
Ni	0.678101	0.178501	0.539543
I	0.789690	-1.961032	1.773904
C	-1.099193	-0.123060	0.037891
C	-1.361309	-0.498845	-1.279960
C	-2.146481	0.144236	0.922034
C	-2.685285	-0.579223	-1.724957
H	-0.546738	-0.726448	-1.962478
C	-3.468144	0.064090	0.468659
H	-1.936989	0.409482	1.953408
C	-3.738869	-0.293658	-0.854505

H	-2.888297	-0.866233	-2.753663
H	-4.283954	0.267040	1.158655
H	-4.766079	-0.357892	-1.202941

5

B3LYP-D3 SCF energy: -726.139707858 a.u.
B3LYP-D3 enthalpy: -725.847364 a.u.
B3LYP-D3 free energy: -725.909656 a.u.
M06 SCF energy in solution: -725.804316856 a.u.
M06 enthalpy in solution: -725.511972998 a.u.
M06 free energy in solution: -725.574264998 a.u.

Cartesian coordinates

ATOM	X	Y	Z
Ni	-0.346003	0.139406	-0.001854
C	-1.145806	2.125513	-0.361296
C	-1.063097	1.862755	0.992042
C	-2.157622	1.398146	1.927495
C	-2.397061	2.079204	-1.223134
H	-0.284621	2.616610	-0.808523
H	-0.163905	2.206643	1.499142
H	-2.626921	2.274051	2.401061
H	-1.673711	0.838748	2.737565
H	-3.264891	2.396878	-0.639555
H	-2.288478	2.812676	-2.029278
C	-2.640807	-0.563663	0.399122
C	-2.411077	-0.482621	-0.936967
C	-3.243090	0.503250	1.284862
C	-2.648859	0.692005	-1.859397
H	-2.415206	-1.514296	0.877128
H	-2.069767	-1.390923	-1.428223
H	-3.960038	1.109127	0.726181
H	-3.809466	0.018290	2.087402
H	-3.668058	0.649806	-2.270932
H	-1.971943	0.572953	-2.713555
C	1.435016	0.774516	0.013561
C	2.000995	1.176318	-1.201808
C	2.168060	0.929027	1.194950
C	3.281997	1.739323	-1.232476
H	1.453326	1.037315	-2.131500
C	3.446775	1.494902	1.163111
H	1.752352	0.586300	2.139319
C	4.006133	1.903867	-0.049958
H	3.712667	2.043130	-2.183946
H	4.008281	1.605799	2.087971
H	5.001474	2.339516	-0.074206
I	0.569506	-2.226165	-0.015490

6

B3LYP-D3 SCF energy: -726.160858261 a.u.
B3LYP-D3 enthalpy: -725.867573 a.u.
B3LYP-D3 free energy: -725.928204 a.u.
M06 SCF energy in solution: -725.824579971 a.u.
M06 enthalpy in solution: -725.531294710 a.u.
M06 free energy in solution: -725.591925710 a.u.

Cartesian coordinates

ATOM	X	Y	Z
Ni	-1.116413	0.494079	-0.339211
C	1.523823	1.229720	0.741471
C	0.059005	0.958339	1.120207
C	-0.702495	2.134149	1.746193
C	1.741486	2.380742	-0.273427
H	1.966143	1.594759	1.684741
H	0.024097	0.085234	1.777647
H	-0.102933	2.599688	2.544498
H	-1.602158	1.731948	2.223990
H	1.468655	3.331164	0.192819
H	2.814451	2.447366	-0.488266
C	-1.433659	2.510692	-0.627426
C	-0.506020	2.038549	-1.551163
C	-1.136548	3.192284	0.698623
C	0.997989	2.210139	-1.603860
H	-2.474686	2.539929	-0.955595
H	-0.927047	1.724068	-2.512190
H	-0.369732	3.960583	0.567314
H	-2.035632	3.709991	1.046487
H	1.209357	3.080279	-2.244681
H	1.411239	1.344073	-2.134461
C	2.330197	-0.005926	0.358307
C	3.587149	-0.203431	0.949764
C	1.903755	-0.934925	-0.600872
C	4.390838	-1.289002	0.599930
H	3.940950	0.503956	1.697447
C	2.701672	-2.024946	-0.953482
H	0.927709	-0.831536	-1.063560
C	3.949731	-2.206133	-0.356053
H	5.357909	-1.420458	1.078382
H	2.338475	-2.735667	-1.690897
H	4.569477	-3.056395	-0.627589
I	-1.956571	-1.780843	0.130425

7a

B3LYP-D3 SCF energy: -611.225666879 a.u.
B3LYP-D3 enthalpy: -610.832558 a.u.
B3LYP-D3 free energy: -610.892251 a.u.
M06 SCF energy in solution: -610.936803924 a.u.
M06 enthalpy in solution: -610.543695045 a.u.
M06 free energy in solution: -610.603388045 a.u.

Cartesian coordinates

ATOM	X	Y	Z
C	-3.211046	-0.293597	4.431843
H	-2.753735	-1.146877	4.950603
H	-3.248497	0.538084	5.148039
C	-2.394211	0.093053	3.191860
H	-2.394083	-0.745311	2.477974
H	-2.890698	0.928065	2.673860
C	-0.945419	0.489703	3.509221
H	-0.459731	-0.347730	4.035237
H	-0.946465	1.322292	4.228035
C	-0.098583	0.885363	2.291916
H	-0.040738	0.018007	1.613695

H	-0.655721	1.649406	1.717211
B	1.400011	1.464748	2.694392
C	2.269438	1.942955	1.366487
H	1.716516	2.693247	0.770985
C	2.371788	0.344975	3.416573
H	1.892743	-0.054497	4.328291
C	2.504830	0.743381	0.419833
H	1.529726	0.447593	0.006297
C	3.570225	2.631335	1.837968
H	3.278336	3.539649	2.382104
C	3.674699	1.033449	3.884119
H	3.386640	1.764725	4.649564
H	4.363988	0.315865	4.369701
C	2.600508	-0.858214	2.475175
H	1.636431	-1.372256	2.346257
H	3.279418	-1.604449	2.929895
H	3.124126	1.031292	-0.451362
H	4.190338	2.963418	0.982996
C	4.455531	1.783494	2.780309
H	5.208640	2.436955	3.251706
H	5.034294	1.064773	2.187146
C	3.150213	-0.501924	1.073527
H	4.233407	-0.349312	1.142507
H	3.025053	-1.368390	0.402877
H	-4.245751	-0.568757	4.182905
O	1.250943	2.592491	3.699275
C	0.544170	3.720425	3.319072
H	0.953075	4.218201	2.415577
H	-0.526721	3.523573	3.102844
H	0.571810	4.466204	4.134574

7b

B3LYP-D3 SCF energy: -645.011696330 a.u.
B3LYP-D3 enthalpy: -644.675033 a.u.
B3LYP-D3 free energy: -644.736010 a.u.
M06 SCF energy in solution: -644.781230779 a.u.
M06 enthalpy in solution: -644.444567449 a.u.
M06 free energy in solution: -644.505544449 a.u.

Cartesian coordinates

ATOM	X	Y	Z
C	-2.839200	0.244526	0.601512
H	-2.963162	-0.843392	0.516813
H	-3.115870	0.522440	1.627425
C	-1.391257	0.652276	0.298518
H	-1.148697	0.390213	-0.743008
H	-1.300399	1.748087	0.360870
C	-0.362697	0.004086	1.236010
H	-0.456028	-1.090109	1.175579
H	-0.608120	0.263455	2.277037
C	1.096515	0.385832	0.963508
H	1.360269	0.111139	-0.071225
H	1.188826	1.485532	1.011584
B	2.142487	-0.352932	1.992758
H	-3.560035	0.718263	-0.080087
O	3.565486	0.054545	1.730279
O	1.795787	-0.051541	3.429741

```
O    2.027643    -1.793991    1.788197
C    2.821456    -2.601858    2.601739
H    2.597172    -2.470819    3.675424
H    2.641011    -3.661016    2.347075
H    3.900485    -2.403925    2.469275
C    3.935649    1.315106    2.179685
H    5.016568    1.464930    1.991644
H    3.409874    2.126666    1.629367
C    2.146308    1.212602    3.885409
H    1.571791    2.015459    3.372190
H    1.897036    1.293596    4.961218
C    3.651939    1.519342    3.689078
C    3.961231    2.961692    4.100506
H    3.740389    3.127818    5.164723
H    5.021974    3.202570    3.940168
H    3.364575    3.678568    3.519935
C    4.490261    0.531296    4.510210
H    4.278247    0.630872    5.584766
H    4.254988    -0.489567    4.202052
H    5.565700    0.704590    4.358864
```

7c

```
B3LYP-D3 SCF energy:              -684.336820255 a.u.
B3LYP-D3 enthalpy:                -683.971431 a.u.
B3LYP-D3 free energy:             -684.034972 a.u.
M06 SCF energy in solution:       -684.081333404 a.u.
M06 enthalpy in solution:         -683.715944149 a.u.
M06 free energy in solution:      -683.779485149 a.u.
```

```
Cartesian coordinates
ATOM  X      Y      Z
C    -1.239247    0.513849    -0.226660
C    0.159752    0.606991    -0.970308
O    0.701900    -0.678667    -0.823721
O    -1.044921    -0.489415    0.736282
B    0.182472    -1.312947    0.431614
O    -0.106107    -2.738475    0.217180
C    -1.000963    -3.092204    -0.784134
H    -0.730461    -2.662798    -1.766616
H    -1.010852    -4.191154    -0.894566
H    -2.037598    -2.774346    -0.564323
C    1.222738    -1.276589    1.699548
H    2.104022    -1.900442    1.472807
H    1.608371    -0.257836    1.869301
C    0.572520    -1.776267    2.995490
H    0.195786    -2.796161    2.830503
H    -0.314127    -1.157939    3.203111
C    1.489155    -1.769792    4.227247
H    1.867721    -0.749047    4.393322
H    2.376234    -2.389144    4.022699
C    0.802285    -2.270706    5.504682
H    0.440912    -3.299627    5.374743
H    1.474036    -2.258626    6.374870
H    -0.070612    -1.649681    5.746739
C    1.105728    1.642581    -0.316768
H    2.108932    1.501749    -0.735583
H    0.790876    2.679204    -0.499760
```

H	1.170045	1.481897	0.761539
C	0.062778	0.934222	-2.469351
H	-0.435131	1.897528	-2.647036
H	1.071750	0.986638	-2.897279
H	-0.482656	0.151995	-3.003845
C	-1.665686	1.810193	0.486016
H	-0.951809	2.075109	1.270220
H	-1.755532	2.654724	-0.211812
H	-2.641099	1.657784	0.964936
C	-2.386207	0.098506	-1.176021
H	-2.111700	-0.785054	-1.755468
H	-3.259147	-0.160469	-0.565374
H	-2.671751	0.900170	-1.871629

7d

B3LYP-D3 SCF energy: -449.630558681 a.u.
B3LYP-D3 enthalpy: -449.420656 a.u.
B3LYP-D3 free energy: -449.471673 a.u.
M06 SCF energy in solution: -449.545331881 a.u.
M06 enthalpy in solution: -449.335429200 a.u.
M06 free energy in solution: -449.386446200 a.u.

Cartesian coordinates

ATOM	X	Y	Z
C	-3.037859	-0.945015	0.859786
H	-2.670542	-1.662651	0.113758
H	-3.063020	-1.467939	1.825380
C	-2.120413	0.283378	0.930279
H	-2.136332	0.805747	-0.039272
H	-2.525631	0.998985	1.662887
C	-0.668167	-0.053459	1.300057
H	-0.264897	-0.768797	0.567945
H	-0.655891	-0.576888	2.268024
C	0.281465	1.146992	1.380282
H	0.273541	1.673434	0.407985
H	-0.102037	1.867604	2.122763
B	1.814563	0.721795	1.778970
H	-4.072339	-0.683986	0.592933
O	2.739016	1.885366	1.936372
H	2.998978	2.106842	1.030761
O	1.811223	-0.011354	3.057712
H	2.673301	0.190169	3.449235
O	2.303811	-0.125746	0.620736
C	3.477047	-0.827740	0.851814
H	3.392023	-1.534036	1.700663
H	3.749540	-1.415519	-0.044839
H	4.341302	-0.167872	1.082644

8a

B3LYP-D3 SCF energy: -1325.89370883 a.u.
B3LYP-D3 enthalpy: -1325.205043 a.u.
B3LYP-D3 free energy: -1325.297462 a.u.
M06 SCF energy in solution: -1325.24051574 a.u.
M06 enthalpy in solution: -1324.55184991 a.u.
M06 free energy in solution: -1324.64426891 a.u.

Cartesian coordinates

ATOM	X	Y	Z
C	2.957602	-0.796688	-1.660071
C	1.292439	-3.277739	-0.081287
H	4.011145	-0.776591	-1.963632
H	2.580277	0.225145	-1.782677
H	2.178696	-3.666653	-0.593279
H	1.209376	-3.851982	0.848841
C	0.652212	-1.519514	-2.457252
C	-0.271024	-2.337012	-1.822547
C	2.152365	-1.712105	-2.599511
C	0.037914	-3.528233	-0.943244
H	0.241524	-0.770241	-3.136115
H	-1.320230	-2.212809	-2.098218
H	2.432043	-2.759515	-2.452830
H	2.412028	-1.465295	-3.636215
H	0.136802	-4.444439	-1.544122
H	-0.831526	-3.678147	-0.292717
C	3.339107	-0.090600	0.755182
C	4.305043	-0.352215	1.735932
C	2.797005	1.201763	0.690218
C	4.716494	0.640678	2.628175
H	4.739981	-1.347488	1.803657
C	3.200030	2.196042	1.581613
H	2.019783	1.427022	-0.030090
C	4.163806	1.920467	2.554485
H	5.468732	0.412893	3.379025
H	2.750523	3.183562	1.517931
H	4.479559	2.694813	3.248398
C	2.870617	-1.207267	-0.167310
H	3.577481	-2.042176	-0.034695
C	1.486259	-1.773133	0.246481
H	1.332808	-1.605530	1.313970
Ni	0.083984	-0.759919	-0.593075
B	-1.731916	0.902308	0.290455
O	-0.285215	0.442415	0.789490
C	-0.065611	0.260123	2.181546
H	-0.535284	1.084499	2.724946
H	-0.496256	-0.686066	2.531098
H	1.007901	0.274868	2.380865
C	-2.802327	-0.275665	0.699193
H	-3.627412	-0.304390	-0.028390
H	-2.310294	-1.263608	0.592137
C	-3.442297	-0.219729	2.098228
H	-3.994443	0.723938	2.209688
H	-2.670190	-0.197471	2.879320
C	-4.396305	-1.385517	2.390325
H	-5.181894	-1.408258	1.620839
H	-3.846951	-2.334001	2.289701
C	-5.039136	-1.310182	3.779488
H	-5.619661	-0.385939	3.893451
H	-5.714981	-2.154280	3.962667
H	-4.274956	-1.316626	4.567457
C	-2.039638	2.399432	0.849141
H	-2.187252	2.391954	1.941384
C	-1.535592	1.108748	-1.348673
H	-1.318534	0.181139	-1.925085
C	-3.381224	2.886227	0.246431

H	-3.607396	3.910813	0.581364
H	-4.181397	2.253212	0.653400
C	-0.840518	3.334947	0.571542
H	0.018488	2.960665	1.145690
H	-1.041093	4.350864	0.947080
C	-2.898101	1.563939	-1.937049
H	-3.620111	0.747394	-1.815210
H	-2.802599	1.725759	-3.022100
C	-0.386063	2.098915	-1.675818
H	0.572774	1.610103	-1.452284
H	-0.359542	2.305331	-2.757975
C	-3.474649	2.842962	-1.295047
H	-2.969074	3.715740	-1.719084
H	-4.527118	2.943728	-1.594793
C	-0.423018	3.441584	-0.911151
H	-1.098541	4.129781	-1.427730
H	0.569815	3.909652	-0.975313

8b

B3LYP-D3 SCF energy: -1359.71191094 a.u.
B3LYP-D3 enthalpy: -1359.079494 a.u.
B3LYP-D3 free energy: -1359.172108 a.u.
M06 SCF energy in solution: -1359.10582584 a.u.
M06 enthalpy in solution: -1358.47340890 a.u.
M06 free energy in solution: -1358.56602290 a.u.

Cartesian coordinates

ATOM	X	Y	Z
C	1.248138	-1.430745	-2.512699
C	3.277925	-0.865446	0.479229
H	1.450634	-1.511853	-3.588392
H	0.230182	-1.033451	-2.414303
H	4.272218	-0.593010	0.089551
H	3.215178	-0.452293	1.494448
C	0.833582	-2.874050	-0.440688
C	1.648704	-2.731030	0.667877
C	1.299650	-2.832510	-1.882578
C	3.125263	-2.396390	0.565681
H	-0.174700	-3.255812	-0.283439
H	1.270301	-3.044379	1.639922
H	2.321995	-3.224330	-1.963898
H	0.657912	-3.503799	-2.464882
H	3.549951	-2.865073	-0.330492
H	3.670320	-2.801743	1.424602
C	2.083850	0.917126	-2.599091
C	3.090348	1.443287	-3.415454
C	0.896826	1.652770	-2.440387
C	2.924677	2.674391	-4.057103
H	4.015003	0.885723	-3.550178
C	0.729111	2.881281	-3.078142
H	0.109362	1.263590	-1.799205
C	1.743299	3.397618	-3.890670
H	3.720657	3.066482	-4.685254
H	-0.195141	3.436885	-2.939986
H	1.612682	4.355321	-4.387490
C	2.250862	-0.421321	-1.895914
H	3.259053	-0.802202	-2.126814

C	2.145875	-0.246895	-0.352843
H	2.107719	0.822229	-0.128408
Ni	0.539259	-0.983808	0.384234
C	-2.133210	-0.427950	3.640563
C	-1.475827	-1.825849	3.578766
C	-1.669233	-2.348825	2.141968
H	-1.997638	0.000907	4.642736
H	-3.221085	-0.538242	3.476678
H	-1.123056	-3.290769	1.992409
H	-2.736067	-2.553439	1.966843
O	-1.226762	-1.423489	1.158865
O	-1.564195	0.454706	2.705567
C	0.019208	-1.713455	3.913953
H	0.160286	-1.306932	4.922583
H	0.505158	-2.697436	3.878745
H	0.527395	-1.046953	3.210333
C	-2.172184	-2.779187	4.558205
H	-1.743007	-3.787732	4.501845
H	-2.057319	-2.427093	5.590365
H	-3.246075	-2.858101	4.347891
B	-1.645246	0.045592	1.322827
O	-0.445454	0.657754	0.582538
C	0.072244	1.904567	1.052135
H	0.707040	1.759957	1.932083
H	0.646993	2.365089	0.245272
H	-0.759129	2.558115	1.324631
C	-3.037228	0.333494	0.568048
H	-2.991470	-0.074868	-0.453654
H	-3.846211	-0.222364	1.072539
C	-3.425031	1.818317	0.507286
H	-2.663599	2.368951	-0.066566
H	-3.405011	2.240188	1.523223
C	-4.797771	2.086918	-0.122283
H	-5.566839	1.548557	0.450722
H	-4.818975	1.658549	-1.135062
C	-5.152411	3.576501	-0.185785
H	-4.415409	4.130992	-0.781002
H	-6.138245	3.742024	-0.636836
H	-5.164591	4.020482	0.817838

8c

B3LYP-D3 SCF energy: -1399.03651407 a.u.
B3LYP-D3 enthalpy: -1398.375575 a.u.
B3LYP-D3 free energy: -1398.471412 a.u.
M06 SCF energy in solution: -1398.40593015 a.u.
M06 enthalpy in solution: -1397.74499108 a.u.
M06 free energy in solution: -1397.84082808 a.u.

Cartesian coordinates

ATOM	X	Y	Z
C	3.399883	-0.569480	-1.441272
C	1.447210	-3.117766	-0.384822
H	4.472649	-0.586847	-1.670138
H	3.104174	0.486845	-1.423861
H	2.346092	-3.500477	-0.880794
H	1.243129	-3.815739	0.435919
C	1.115600	-0.966434	-2.487699

C	0.096137	-1.802282	-2.058976
C	2.604459	-1.258688	-2.563966
C	0.258799	-3.137841	-1.369126
H	0.804787	-0.098605	-3.065888
H	-0.917885	-1.540338	-2.361845
H	2.797036	-2.335830	-2.565999
H	2.960067	-0.879798	-3.530356
H	0.352170	-3.948973	-2.107316
H	-0.671071	-3.328100	-0.820434
C	3.620638	-0.274948	1.073753
C	4.504419	-0.730277	2.060594
C	3.128447	1.033303	1.183259
C	4.882138	0.090921	3.125759
H	4.898923	-1.742322	1.996090
C	3.496645	1.857758	2.245954
H	2.427023	1.402872	0.443426
C	4.377880	1.389145	3.223575
H	5.570623	-0.284833	3.878415
H	3.088149	2.862936	2.311462
H	4.668013	2.029162	4.052485
C	3.166401	-1.199958	-0.043841
H	3.807008	-2.095286	0.004767
C	1.709575	-1.692083	0.177216
H	1.499172	-1.683831	1.250492
Ni	0.432455	-0.459137	-0.578465
C	-1.305867	2.068239	-1.599815
C	-1.297394	2.997385	-0.327515
O	-1.881677	2.170731	0.675401
O	-1.121638	0.744070	-1.034608
B	-1.544314	0.802749	0.430640
O	-0.136184	0.417919	1.011511
C	-0.000982	0.090026	2.384726
H	-0.480059	0.874657	2.979803
H	-0.472726	-0.874277	2.614065
H	1.061086	0.051832	2.636602
C	-2.684150	-0.262730	0.827847
H	-3.403939	-0.285416	-0.005706
H	-2.262272	-1.281710	0.876325
C	-3.455229	0.041036	2.121860
H	-3.837463	1.069553	2.069082
H	-2.774444	0.021382	2.985469
C	-4.616571	-0.923619	2.393134
H	-5.309634	-0.900059	1.539377
H	-4.228847	-1.952314	2.439875
C	-5.379320	-0.603244	3.683156
H	-5.800492	0.409479	3.648368
H	-6.205638	-1.304109	3.852820
H	-4.714934	-0.652278	4.555495
C	-2.159159	4.254083	-0.474563
H	-2.082028	4.855270	0.437286
H	-1.825318	4.869393	-1.318791
H	-3.211057	3.996830	-0.616388
C	-2.671544	2.038678	-2.298077
H	-2.882375	2.977532	-2.821199
H	-2.677455	1.223922	-3.029659
H	-3.466122	1.851936	-1.570779
C	-0.203627	2.375606	-2.609040
H	0.788026	2.203045	-2.182109

H	-0.312388	1.746493	-3.499619
H	-0.264848	3.421029	-2.931595
C	0.123278	3.405945	0.100978
H	0.569513	4.130920	-0.589238
H	0.064833	3.862022	1.094425
H	0.770926	2.532414	0.176405

8d

B3LYP-D3 SCF energy: -1164.33836620 a.u.
B3LYP-D3 enthalpy: -1163.833567 a.u.
B3LYP-D3 free energy: -1163.918011 a.u.
M06 SCF energy in solution: -1163.87170033 a.u.
M06 enthalpy in solution: -1163.36690113 a.u.
M06 free energy in solution: -1163.45134513 a.u.

Cartesian coordinates

ATOM	X	Y	Z
C	2.078572	-1.266777	-2.642916
C	3.623752	-1.535557	0.674653
H	2.428840	-1.279668	-3.683144
H	1.197471	-0.613086	-2.614588
H	4.709917	-1.457786	0.504038
H	3.446646	-1.244393	1.717888
C	0.986101	-2.784628	-0.881674
C	1.632840	-2.980898	0.327384
C	1.661297	-2.690650	-2.237367
C	3.144063	-2.988206	0.475080
H	-0.091014	-2.944975	-0.910340
H	1.043716	-3.311803	1.184168
H	2.547232	-3.339453	-2.262377
H	0.965660	-3.081072	-2.988925
H	3.601987	-3.409276	-0.428622
H	3.445144	-3.626780	1.312208
C	3.606372	0.687827	-2.283135
C	4.900089	0.914253	-2.766628
C	2.694879	1.755576	-2.304630
C	5.277776	2.168045	-3.254923
H	5.621392	0.099813	-2.758849
C	3.064744	3.008229	-2.791405
H	1.683615	1.600202	-1.935173
C	4.361196	3.219811	-3.269540
H	6.289115	2.320791	-3.623163
H	2.341902	3.820114	-2.798144
H	4.652202	4.195765	-3.648533
C	3.192623	-0.672906	-1.746484
H	4.059626	-1.344765	-1.850408
C	2.829283	-0.588895	-0.235298
H	2.960953	0.439539	0.114863
Ni	1.006200	-1.011783	0.191534
O	0.531488	0.741829	0.783900
O	-1.855945	1.152280	0.510085
B	-0.912044	0.391334	1.266628
O	-0.890292	-1.049326	0.743875
C	-1.734939	-2.038927	1.287599
H	-2.783407	-1.731555	1.195036
H	-1.596651	-2.971980	0.728566
H	-1.510696	-2.227252	2.346855

C	-0.961507	0.516254	2.879130
H	-0.337936	-0.273727	3.328853
H	-0.464185	1.459229	3.156200
C	-2.354182	0.493983	3.531720
H	-2.876648	-0.442960	3.291561
H	-2.982030	1.291430	3.101160
C	-2.329313	0.666757	5.056013
H	-1.819311	1.609251	5.302086
H	-1.715033	-0.132783	5.495194
C	-3.723815	0.652644	5.691069
H	-4.241502	-0.292763	5.484125
H	-3.675241	0.775471	6.779540
H	-4.347011	1.462648	5.291008
H	-2.316879	1.783068	1.077017
H	0.459255	1.397981	0.071323

9a

B3LYP-D3 SCF energy: -1325.90413724 a.u.
B3LYP-D3 enthalpy: -1325.214544 a.u.
B3LYP-D3 free energy: -1325.309577 a.u.
M06 SCF energy in solution: -1325.24683208 a.u.
M06 enthalpy in solution: -1324.55723884 a.u.
M06 free energy in solution: -1324.65227184 a.u.

Cartesian coordinates

ATOM	X	Y	Z
C	1.824198	-0.880605	-1.979979
C	2.678232	-1.882999	1.424083
H	2.375797	-0.688078	-2.910149
H	0.960268	-0.205694	-1.984596
H	3.779025	-1.846104	1.491355
H	2.299408	-1.829199	2.452963
C	0.399474	-2.718603	-0.867103
C	0.798587	-3.155427	0.366190
C	1.327245	-2.337738	-2.004558
C	2.245320	-3.229280	0.802604
H	-0.655707	-2.805717	-1.115633
H	0.051216	-3.586411	1.033031
H	2.199071	-3.006159	-2.018643
H	0.794967	-2.504603	-2.948930
H	2.879785	-3.448223	-0.065126
H	2.388760	-4.050617	1.513966
C	3.240643	0.909250	-0.980973
C	4.599499	1.183553	-1.173878
C	2.341343	1.988180	-0.970051
C	5.053469	2.494049	-1.347953
H	5.311862	0.361098	-1.185852
C	2.788532	3.296967	-1.144095
H	1.284724	1.793444	-0.812154
C	4.149123	3.556485	-1.334211
H	6.114352	2.682461	-1.493895
H	2.074501	4.116977	-1.129090
H	4.499168	4.576642	-1.468830
C	2.740783	-0.512607	-0.785509
H	3.617374	-1.178183	-0.853927
C	2.119311	-0.684106	0.641186
H	2.355866	0.217746	1.211925

Ni	0.226289	-1.010012	0.615140
B	-2.708275	-0.690770	-0.187540
O	-1.922587	-1.473271	0.637165
C	-2.365725	-1.838786	1.956961
H	-3.326467	-2.358202	1.905743
H	-1.606779	-2.501481	2.375462
H	-2.451459	-0.948847	2.585176
C	-0.105028	0.610188	1.623314
H	-1.182216	0.841265	1.537675
C	0.246809	0.429766	3.104640
H	1.322473	0.228624	3.205328
H	-0.258681	-0.460573	3.511834
C	-0.110629	1.638016	3.986714
H	-1.188196	1.842161	3.897271
H	0.401484	2.527340	3.592756
C	0.256061	1.439224	5.461447
H	1.333180	1.264125	5.577091
H	-0.007631	2.313546	6.068591
H	-0.265592	0.570042	5.883146
C	-2.190984	-0.243731	-1.600391
H	-1.210950	-0.666970	-1.845370
C	-4.127722	-0.108531	0.159043
H	-4.523940	-0.420573	1.134344
C	-3.207803	-0.736872	-2.667354
H	-2.920057	-0.359982	-3.659306
H	-3.127468	-1.833311	-2.725418
C	-2.016128	1.302355	-1.525653
H	-1.167445	1.498689	-0.857246
H	-1.728908	1.690620	-2.513503
C	-5.126235	-0.599427	-0.929302
H	-5.280137	-1.678876	-0.779070
H	-6.107648	-0.129849	-0.770605
C	-3.940671	1.441310	0.216989
H	-3.344046	1.671226	1.111129
H	-4.918025	1.920496	0.372336
C	-4.680055	-0.365378	-2.388687
H	-4.850454	0.679972	-2.659298
H	-5.328097	-0.950114	-3.054804
C	-3.249666	2.077106	-1.011016
H	-2.941971	3.097675	-0.748641
H	-3.976121	2.190231	-1.820499
H	0.433137	1.482942	1.229329

9b

B3LYP-D3 SCF energy: -1359.70485685 a.u.
B3LYP-D3 enthalpy: -1359.071475 a.u.
B3LYP-D3 free energy: -1359.168519 a.u.
M06 SCF energy in solution: -1359.10312311 a.u.
M06 enthalpy in solution: -1358.46974126 a.u.
M06 free energy in solution: -1358.56678526 a.u.

Cartesian coordinates

ATOM	X	Y	Z
C	1.546581	-0.746010	-2.044419
C	2.760465	-1.942299	1.175997
H	2.002549	-0.440252	-2.996256
H	0.623028	-0.167152	-1.933681

H	3.859751	-1.855945	1.127658
H	2.496894	-1.974454	2.241045
C	0.339370	-2.730952	-0.971110
C	0.838993	-3.225630	0.200529
C	1.174631	-2.235568	-2.132879
C	2.318430	-3.268718	0.517838
H	-0.728870	-2.809918	-1.152789
H	0.157616	-3.716038	0.897127
H	2.093674	-2.829532	-2.227607
H	0.604232	-2.394871	-3.055911
H	2.887490	-3.422097	-0.407188
H	2.545663	-4.119031	1.171074
C	2.834356	1.114567	-1.016698
C	4.129721	1.572452	-1.283125
C	1.814822	2.068385	-0.858342
C	4.405985	2.938898	-1.386595
H	4.933267	0.849815	-1.409786
C	2.084714	3.432203	-0.959640
H	0.807766	1.727480	-0.635691
C	3.383967	3.875014	-1.225665
H	5.421060	3.269791	-1.592703
H	1.280918	4.152923	-0.827272
H	3.596017	4.938175	-1.304317
C	2.524481	-0.368873	-0.899720
H	3.461041	-0.916991	-1.098463
C	2.069117	-0.715223	0.558440
H	2.320645	0.142832	1.188902
Ni	0.201544	-1.131218	0.653076
C	-3.845522	1.397330	-0.165403
C	-3.728286	1.303290	-1.696576
C	-2.300300	0.829751	-2.016895
H	-4.881232	1.592224	0.131940
H	-3.221925	2.217127	0.215204
H	-2.187887	0.634833	-3.088300
H	-1.566494	1.597281	-1.734287
O	-1.968491	-0.382978	-1.333763
O	-3.438139	0.191293	0.494615
C	-4.757729	0.302840	-2.251592
H	-5.777683	0.629141	-2.017570
H	-4.668424	0.222689	-3.341088
H	-4.618630	-0.697120	-1.829860
C	-3.953158	2.693446	-2.307889
H	-3.862318	2.655981	-3.399674
H	-4.957025	3.064994	-2.070892
H	-3.224792	3.422434	-1.932850
B	-2.464186	-0.573486	-0.075306
O	-1.941579	-1.648143	0.629249
C	-2.490859	-1.979312	1.913899
H	-3.545598	-2.250872	1.811769
H	-1.919153	-2.829911	2.289123
H	-2.393114	-1.136614	2.601595
C	-0.120507	0.390943	1.812178
H	0.414743	1.296795	1.499236
H	-1.196994	0.642553	1.795049
C	0.283314	0.045650	3.251464
H	1.360971	-0.168305	3.286868
H	-0.212777	-0.883165	3.576116
C	-0.031080	1.148883	4.276184

H   -1.109982   1.363437   4.253507
H   0.470971   2.075534   3.963262
C   0.393018   0.786564   5.703767
H   1.472749   0.597129   5.755477
H   0.159800   1.588272   6.414936
H   -0.117057   -0.123127   6.046744

9c

B3LYP-D3 SCF energy: -1399.03318164 a.u.
B3LYP-D3 enthalpy: -1398.371355 a.u.
B3LYP-D3 free energy: -1398.468944 a.u.
M06 SCF energy in solution: -1398.40899602 a.u.
M06 enthalpy in solution: -1397.74716938 a.u.
M06 free energy in solution: -1397.84475838 a.u.

Cartesian coordinates

ATOM	X	Y	Z
C	1.599350	-0.601941	-1.999706
C	2.700907	-2.098013	1.140840
H	2.063091	-0.299289	-2.948607
H	0.769885	0.090042	-1.814278
H	3.803104	-2.135749	1.099929
H	2.425419	-2.156897	2.201975
C	0.176634	-2.489855	-1.003268
C	0.645736	-3.108195	0.122346
C	1.028183	-2.020866	-2.164762
C	2.116483	-3.325589	0.405397
H	-0.898275	-2.450432	-1.158807
H	-0.071817	-3.563159	0.806425
H	1.858227	-2.719778	-2.336968
H	0.409747	-2.041743	-3.069937
H	2.655067	-3.471669	-0.538972
H	2.262892	-4.237146	0.996141
C	3.226805	0.958221	-0.945272
C	4.564607	1.183543	-1.289589
C	2.414621	2.072779	-0.679398
C	5.081855	2.480031	-1.365157
H	5.210393	0.333393	-1.499919
C	2.924678	3.367935	-0.754337
H	1.378908	1.912288	-0.399295
C	4.263283	3.578245	-1.098843
H	6.125272	2.629643	-1.632117
H	2.277370	4.215361	-0.540639
H	4.663119	4.587344	-1.156461
C	2.652576	-0.446574	-0.871516
H	3.470160	-1.145154	-1.116141
C	2.159862	-0.772884	0.578128
H	2.511018	0.027931	1.235113
Ni	0.256162	-0.987453	0.700683
C	-3.819127	1.421373	-0.452437
C	-2.619900	1.294699	-1.471490
O	-2.116049	-0.044982	-1.186686
O	-3.388796	0.556019	0.642981
B	-2.468726	-0.304793	0.111512
O	-1.916140	-1.358041	0.805446
C	-2.415258	-1.654231	2.121632
H	-3.482322	-1.888770	2.071464

H	-1.855356	-2.518569	2.481935
H	-2.253086	-0.805785	2.789783
C	0.109094	0.524696	1.903876
H	0.700321	1.392674	1.582702
H	-0.945533	0.857009	1.925879
C	0.536622	0.129132	3.322815
H	1.593195	-0.173846	3.314574
H	-0.021845	-0.759997	3.656895
C	0.353034	1.240696	4.369952
H	-0.704305	1.544335	4.390058
H	0.918653	2.126651	4.047776
C	0.797750	0.825469	5.776643
H	1.859156	0.546795	5.785623
H	0.658202	1.634199	6.504152
H	0.226745	-0.043375	6.129393
C	-1.474431	2.270588	-1.179414
H	-0.616554	2.009707	-1.805035
H	-1.762904	3.302961	-1.401443
H	-1.156962	2.206613	-0.134531
C	-3.014705	1.357944	-2.942407
H	-3.472984	2.325434	-3.176018
H	-2.122128	1.243375	-3.565206
H	-3.716015	0.563348	-3.205982
C	-4.043525	2.822535	0.104277
H	-3.162496	3.184019	0.638453
H	-4.279526	3.523637	-0.704050
H	-4.884812	2.811422	0.804353
C	-5.129084	0.832782	-0.985507
H	-4.972791	-0.174430	-1.384927
H	-5.848912	0.766631	-0.164123
H	-5.558027	1.458385	-1.774665

9d

B3LYP-D3 SCF energy: -1164.34146868 a.u.
B3LYP-D3 enthalpy: -1163.835275 a.u.
B3LYP-D3 free energy: -1163.920944 a.u.
M06 SCF energy in solution: -1163.87733024 a.u.
M06 enthalpy in solution: -1163.37113656 a.u.
M06 free energy in solution: -1163.45680556 a.u.

Cartesian coordinates

ATOM	X	Y	Z
C	2.095590	-0.975777	-2.103800
C	2.607931	-1.874953	1.393737
H	2.730110	-0.788757	-2.980413
H	1.220244	-0.322196	-2.211224
H	3.692077	-1.802668	1.583964
H	2.115762	-1.809686	2.373385
C	0.614200	-2.829773	-1.092713
C	0.897696	-3.245063	0.177772
C	1.641792	-2.446146	-2.139441
C	2.290747	-3.252627	0.765353
H	-0.409455	-2.938196	-1.445791
H	0.095864	-3.677887	0.776316
H	2.529084	-3.086780	-2.045130
H	1.214221	-2.652909	-3.127721
H	3.022433	-3.466856	-0.023218

H	2.387639	-4.049184	1.511572
C	3.391429	0.857725	-1.015595
C	4.762348	1.130164	-1.091436
C	2.495035	1.934066	-1.115402
C	5.230255	2.436537	-1.258218
H	5.473015	0.309558	-1.016657
C	2.956135	3.239109	-1.282553
H	1.428271	1.741883	-1.047729
C	4.328256	3.496663	-1.355021
H	6.299969	2.623624	-1.311836
H	2.243751	4.057407	-1.353917
H	4.688958	4.513758	-1.484171
C	2.879066	-0.561477	-0.832655
H	3.762268	-1.220413	-0.793574
C	2.111954	-0.707278	0.522338
H	2.267402	0.212869	1.092434
Ni	0.237578	-1.091520	0.349089
O	-1.908397	-0.368875	-1.791316
O	-3.925316	-1.222861	-0.847895
B	-2.569894	-1.062392	-0.821566
O	-1.843941	-1.643703	0.211245
C	-2.506636	-2.113338	1.394799
H	-3.405408	-2.675016	1.129982
H	-1.799856	-2.754348	1.924693
H	-2.771645	-1.263110	2.030015
C	-0.247077	0.581340	1.175640
H	0.347020	1.442569	0.845531
H	-1.299523	0.791856	0.913534
C	-0.124161	0.433110	2.697749
H	0.923280	0.231077	2.963825
H	-0.693167	-0.444345	3.044324
C	-0.601590	1.663933	3.487425
H	-1.651885	1.868610	3.233059
H	-0.029859	2.541451	3.154032
C	-0.459780	1.498280	5.004421
H	0.586790	1.322108	5.283626
H	-0.804713	2.387932	5.544769
H	-1.044993	0.641615	5.363531
H	-4.310162	-0.757795	-1.604769
H	-0.976846	-0.224823	-1.536304

a

B3LYP-D3 SCF energy: -701.593815914 a.u.
B3LYP-D3 enthalpy: -701.173635 a.u.
B3LYP-D3 free energy: -701.238093 a.u.
M06 SCF energy in solution: -701.177302756 a.u.
M06 enthalpy in solution: -700.757121842 a.u.
M06 free energy in solution: -700.821579842 a.u.

Cartesian coordinates

ATOM	X	Y	Z
C	-1.384815	3.281280	-0.005523
C	-0.987788	1.905119	0.545466
C	-0.704149	0.838058	-0.529819
C	-0.048671	-0.470797	0.050548
C	1.471288	-0.334036	0.027634
C	2.191140	-0.841658	-1.064735

```
C    3.575819    -0.695452    -1.155509
C    4.277228    -0.032459    -0.146398
C    3.578291    0.476735    0.949119
C    2.192438    0.325737    1.034656
C    -0.529566    -0.957577    1.438848
C    -2.042377    -1.248704    1.595352
C    -2.625520    -2.048559    0.461994
C    -3.124679    -1.499161    -0.650580
C    -3.196248    -0.014878    -0.931793
C    -1.885532    0.578215    -1.500088
H    -0.651046    3.598883    -0.761537
H    -2.350604    3.216429    -0.525268
H    -0.079749    2.027108    1.150916
H    -1.766979    1.565018    1.238101
H    0.069554    1.273499    -1.178355
H    -0.295356    -1.270222    -0.657620
H    1.653915    -1.359926    -1.856456
H    4.106593    -1.102762    -2.012432
H    5.355941    0.082505    -0.211257
H    4.112118    0.991886    1.743890
H    1.674677    0.723511    1.902067
H    0.032598    -1.874045    1.661620
H    -0.249583    -0.237761    2.216655
H    -2.582719    -0.300898    1.689546
H    -2.187294    -1.777436    2.546133
H    -2.577769    -3.134418    0.542596
H    -3.475723    -2.155339    -1.447108
H    -3.993883    0.166984    -1.662816
H    -3.484144    0.539157    -0.029263
H    -1.524280    -0.081052    -2.301595
H    -2.127875    1.530969    -1.987556
C    -1.483331    4.353445    1.087062
H    -0.514260    4.437803    1.599280
H    -2.203941    4.026236    1.850479
C    -1.898584    5.724350    0.543367
H    -2.880421    5.674092    0.056131
H    -1.959446    6.472867    1.341815
H    -1.178855    6.086708    -0.201249
```

b

```
B3LYP-D3 SCF energy:               -701.593840877 a.u.
B3LYP-D3 enthalpy:                 -701.173599 a.u.
B3LYP-D3 free energy:              -701.238103 a.u.
M06 SCF energy in solution:        -701.178053320 a.u.
M06 enthalpy in solution:          -700.757811443 a.u.
M06 free energy in solution:       -700.822315443 a.u.
```

```
Cartesian coordinates
ATOM  X      Y      Z
C    -0.92225000    3.44543500    0.37205300
C    -0.56698400    2.01263100    0.79159100
C    -0.79830300    0.94677600   -0.29631700
C    -0.13915600   -0.42487300    0.06932600
C     1.38104500   -0.31279600   -0.01926700
C     2.21002800   -0.11083400    1.09299700
C     3.59440300    0.01720200    0.94792000
C     4.18086600   -0.05447700   -0.31551200
```

C	3.36966800	-0.25812400	-1.43484900
C	1.98988700	-0.38619200	-1.28242500
C	-0.61219800	-1.05335600	1.39288900
C	-2.13237900	-1.32420000	1.48509600
C	-2.70030600	-2.09577000	0.31298200
C	-2.92565100	-1.66743200	-0.93656200
C	-2.73134600	-0.30849900	-1.57208900
C	-2.29513200	0.88080900	-0.69937600
H	-0.40114600	3.68788400	-0.56652600
H	-1.99635200	3.52402100	0.15535400
H	0.48901800	1.99151200	1.09018200
H	-1.14658300	1.74956400	1.68884700
H	-0.25997400	1.28118900	-1.19630800
H	-0.42650700	-1.12451600	-0.72332200
H	1.77947800	-0.05459400	2.08776400
H	4.21360400	0.17214100	1.82798600
H	5.25738000	0.04307900	-0.42788500
H	3.81246600	-0.32328900	-2.42557800
H	1.36564800	-0.54860800	-2.15926000
H	-0.07099500	-1.99927200	1.52500700
H	-0.33519300	-0.41982200	2.24409800
H	-2.65822300	-0.37497500	1.61772400
H	-2.31624800	-1.89275200	2.40473200
H	-2.91613600	-3.14590000	0.51041100
H	-3.31588900	-2.40666100	-1.63895500
H	-2.02123100	-0.41999500	-2.40871700
H	-3.68371600	-0.04243700	-2.05585200
H	-2.51060400	1.79233900	-1.27019500
H	-2.93913500	0.93952800	0.18425100
C	-0.55450200	4.48621500	1.43712100
H	0.52235200	4.42180700	1.64831600
H	-1.06521400	4.23585100	2.37817300
C	-0.91305100	5.91719400	1.02323700
H	-1.99005300	6.01584900	0.83789500
H	-0.63951500	6.64172700	1.79898100
H	-0.39206300	6.20199000	0.10056800

c

B3LYP-D3 SCF energy: -701.589832152 a.u.
B3LYP-D3 enthalpy: -701.169568 a.u.
B3LYP-D3 free energy: -701.233573 a.u.
M06 SCF energy in solution: -701.172478043 a.u.
M06 enthalpy in solution: -700.752213891 a.u.
M06 free energy in solution: -700.816218891 a.u.

Cartesian coordinates

ATOM	X	Y	Z
C	1.49702200	-0.48345700	-2.13989300
C	2.72761700	-1.14598700	-1.46744000
C	-0.08244100	-1.25915900	0.97646700
C	2.48388300	-2.48534600	-0.80670100
C	1.41797100	-1.51229200	1.24724800
C	1.95083900	-2.64388500	0.40964800
H	1.04005900	-1.21850600	-2.81540000
H	3.15945700	-0.45724900	-0.73393500
H	-0.64740300	-2.12816200	1.33582400
H	1.54658800	-1.73309000	2.31466000

H	1.86365400	0.32282400	-2.78929000
H	3.49029800	-1.27316900	-2.24616900
H	-0.40650400	-0.40707500	1.58522800
H	2.76884900	-3.36980300	-1.37618900
H	1.97339700	-0.58916000	1.04945000
H	1.80176400	-3.65278500	0.79535400
C	0.35213900	0.09572600	-1.26154300
H	-0.35683600	0.48444200	-2.00846300
C	-0.45187600	-1.03303100	-0.50426200
H	-0.19695500	-1.96188200	-1.02924600
C	0.73473100	1.32950200	-0.43336800
C	-0.21420600	1.93827700	0.40805400
C	1.98633800	1.95800300	-0.53370500
C	0.08039300	3.08789600	1.13965800
H	-1.20855600	1.51452900	0.48769800
C	2.28841600	3.11174300	0.19521900
H	2.74707200	1.56097900	-1.19485700
C	1.34074200	3.68069000	1.04367700
H	-0.68118100	3.52377300	1.78148300
H	3.27143800	3.56448100	0.09232200
H	1.57510800	4.57591500	1.61326100
C	-1.98051700	-0.86770600	-0.68748800
H	-2.17743100	-0.65089900	-1.74756500
H	-2.34858400	0.00473200	-0.13076600
C	-2.81680600	-2.09207300	-0.28923200
H	-2.74295800	-2.26969400	0.79179700
H	-2.40394800	-2.98892600	-0.77561100
C	-4.29766700	-1.94875500	-0.66421400
H	-4.70434400	-1.04336800	-0.19107700
H	-4.38351700	-1.79007400	-1.74867500
C	-5.13721700	-3.16321900	-0.25452000
H	-4.76856600	-4.07725600	-0.73666600
H	-6.18957200	-3.03832200	-0.53456400
H	-5.09611300	-3.32285700	0.83021500

d

B3LYP-D3 SCF energy: -701.594926743 a.u.
B3LYP-D3 enthalpy: -701.174667 a.u.
B3LYP-D3 free energy: -701.238767 a.u.
M06 SCF energy in solution: -701.178148393 a.u.
M06 enthalpy in solution: -700.757888650 a.u.
M06 free energy in solution: -700.821988650 a.u.

Cartesian coordinates

ATOM	X	Y	Z
C	-0.63425000	2.07187700	-2.55177000
C	-0.59450500	2.08436500	-1.01817900
C	-1.09512700	0.79518700	-0.32694200
C	-0.18167100	-0.42814500	-0.66420700
C	1.27257800	-0.22314800	-0.25964300
C	2.24174700	0.06764600	-1.23110500
C	3.57793400	0.28029400	-0.88661400
C	3.97736900	0.20210300	0.44825700
C	3.02859100	-0.09501700	1.42873500
C	1.69469400	-0.30671600	1.07702500
C	-0.69886400	-1.81556500	-0.20021600
C	-2.17722800	-2.11422500	-0.50175200

C	-3.25477500	-1.50660000	0.36810800
C	-3.17699300	-0.67125200	1.41262000
C	-1.94312700	-0.04073000	2.02223900
C	-1.32455400	1.09146000	1.16772500
H	0.02746600	1.29086300	-2.94809800
H	-1.64896400	1.81051500	-2.88858100
H	0.42703600	2.30772500	-0.68163400
H	-1.21801800	2.92061100	-0.67101700
H	-2.07713600	0.56332100	-0.75983300
H	-0.19374700	-0.48471000	-1.76048900
H	1.94522000	0.12634400	-2.27558200
H	4.30576100	0.50386700	-1.66253700
H	5.01626500	0.36623400	0.72162800
H	3.32675700	-0.16512900	2.47180700
H	0.97760100	-0.54521900	1.85615600
H	-0.09253900	-2.56254100	-0.72909400
H	-0.49799300	-1.98747000	0.85905300
H	-2.31516500	-3.20605700	-0.46915600
H	-2.39039300	-1.84089100	-1.54867000
H	-4.25758300	-1.82286400	0.07399400
H	-4.12289500	-0.37310800	1.86516900
H	-2.20922300	0.38434500	2.99778500
H	-1.18901100	-0.80278800	2.23560600
H	-1.99759800	1.95807300	1.22128400
H	-0.38236400	1.41181500	1.63136400
C	-0.22334600	3.41524800	-3.16775400
H	-0.88776200	4.20589500	-2.79098500
H	0.78691100	3.67464300	-2.82055200
C	-0.25369100	3.40444500	-4.69955800
H	-1.25974300	3.17434700	-5.07224000
H	0.04349700	4.37406200	-5.11559900
H	0.42856900	2.64480000	-5.10164100

COD (2a)

B3LYP-D3 SCF energy: -312.040162014 a.u.
B3LYP-D3 enthalpy: -311.850482 a.u.
B3LYP-D3 free energy: -311.890284 a.u.
M06 SCF energy in solution: -311.857843482 a.u.
M06 enthalpy in solution: -311.668163468 a.u.
M06 free energy in solution: -311.707965468 a.u.

Cartesian coordinates

ATOM	X	Y	Z
C	0.032151	-1.701727	-0.220846
C	-1.921170	-0.012342	-0.021104
C	1.094365	-1.093952	0.669883
H	0.332565	-2.622573	-0.721862
H	-2.732850	-0.322202	0.657004
H	-2.429782	0.429991	-0.890723
H	0.666895	-0.713291	1.600339
H	1.791444	-1.886716	0.969244
C	-0.032153	1.701726	-0.220845
C	1.195346	1.246471	-0.499767
C	-1.094361	1.093949	0.669889
C	1.921171	0.012347	-0.021102
H	-0.332575	2.622566	-0.721866
H	1.792113	1.853288	-1.183049

H	-0.666888	0.713280	1.600339
H	-1.791437	1.886713	0.969259
H	2.732842	0.322209	0.657016
H	2.429794	-0.429982	-0.890717
C	-1.195349	-1.246470	-0.499764
H	-1.792122	-1.853292	-1.183037

DMFU
B3LYP-D3 SCF energy: -534.348928523 a.u.
B3LYP-D3 enthalpy: -534.197482 a.u.
B3LYP-D3 free energy: -534.246988 a.u.
M06 SCF energy in solution: -534.192613211 a.u.
M06 enthalpy in solution: -534.041166688 a.u.
M06 free energy in solution: -534.090672688 a.u.

Cartesian coordinates

ATOM	X	Y	Z
C	1.900628	-0.228808	0.000211
C	0.547885	0.383805	-0.000089
C	-0.547921	-0.383527	0.000036
C	-1.900692	0.229000	-0.000179
H	0.470575	1.466618	-0.000392
H	-0.470532	-1.466335	0.000334
O	2.853912	0.728103	-0.000153
O	2.135852	-1.420984	0.000445
O	-2.136030	1.421131	-0.000828
O	-2.853909	-0.728045	0.000199
C	4.206070	0.241262	0.000018
H	4.838026	1.129710	0.000497
H	4.393413	-0.367836	0.889141
H	4.393861	-0.367144	-0.889491
C	-4.206105	-0.241294	0.000045
H	-4.838004	-1.129784	0.001694
H	-4.393340	0.368859	0.888464
H	-4.394107	0.366019	-0.890170

I^-
B3LYP-D3 SCF energy: -11.5189090900 a.u.
B3LYP-D3 enthalpy: -11.516549 a.u.
B3LYP-D3 free energy: -11.535757 a.u.
M06 SCF energy in solution: -11.5323873511 a.u.
M06 enthalpy in solution: -11.5300272611 a.u.
M06 free energy in solution: -11.5492352611 a.u.

Cartesian coordinates

ATOM	X	Y	Z
I	-2.101167	-0.116732	0.000000

OMe^-
B3LYP-D3 SCF energy: -115.069876871 a.u.
B3LYP-D3 enthalpy: -115.031448 a.u.
B3LYP-D3 free energy: -115.057512 a.u.
M06 SCF energy in solution: -115.125842144 a.u.
M06 enthalpy in solution: -115.087413273 a.u.
M06 free energy in solution: -115.113477273 a.u.

Cartesian coordinates

ATOM	X	Y	Z
O	2.112406	0.206820	-0.000010
C	1.675480	1.440503	-0.000105
H	1.980013	2.123940	-0.892274
H	0.523216	1.608059	-0.000010
H	1.980256	2.123506	0.892399

PhI (1a)

B3LYP-D3 SCF energy: -243.061506572 a.u.
B3LYP-D3 enthalpy: -242.964356 a.u.
B3LYP-D3 free energy: -243.002964 a.u.
M06 SCF energy in solution: -242.903950215 a.u.
M06 enthalpy in solution: -242.806799643 a.u.
M06 free energy in solution: -242.845407643 a.u.

Cartesian coordinates

ATOM	X	Y	Z
C	-0.588417	0.000015	0.000122
C	-1.268987	1.216879	0.000057
C	-2.666695	1.207637	-0.000001
C	-3.366712	-0.000012	-0.000073
C	-2.666673	-1.207649	-0.000003
C	-1.268966	-1.216869	0.000059
H	-0.726815	2.156069	-0.000042
H	-3.204206	2.152042	-0.000042
H	-4.452960	-0.000014	-0.000111
H	-3.204166	-2.152064	-0.000052
H	-0.726791	-2.156055	-0.000031
I	1.571201	0.000000	-0.000013

TS1a

B3LYP-D3 SCF energy: -1325.88197570 a.u.
B3LYP-D3 enthalpy: -1325.194073 a.u.
B3LYP-D3 free energy: -1325.285040 a.u.
M06 SCF energy in solution: -1325.23143364 a.u.
M06 enthalpy in solution: -1324.54353094 a.u.
M06 free energy in solution: -1324.63449794 a.u.
Imaginary frequency: -215.9013 cm-1

Cartesian coordinates

ATOM	X	Y	Z
C	1.912386	-0.802154	-2.065609
C	2.498745	-2.045704	1.327867
H	2.532030	-0.525328	-2.928581
H	1.018891	-0.170002	-2.101553
H	3.580338	-1.934851	1.512289
H	2.016099	-2.080074	2.314541
C	0.483262	-2.761032	-1.184792
C	0.802328	-3.315977	0.038272
C	1.482076	-2.272251	-2.217594
C	2.211233	-3.368904	0.589793
H	-0.540786	-2.879900	-1.535255
H	0.035864	-3.880933	0.566661
H	2.379258	-2.905899	-2.198225

H	1.028519	-2.401090	-3.207103
H	2.929164	-3.495453	-0.230243
H	2.333645	-4.229771	1.255887
C	3.203053	0.915125	-0.727563
C	4.524993	1.188769	-0.351269
C	2.356776	2.002267	-0.998746
C	4.989554	2.500891	-0.240258
H	5.198300	0.360655	-0.139523
C	2.815625	3.315484	-0.888277
H	1.327640	1.826544	-1.297966
C	4.134658	3.571744	-0.507011
H	6.020193	2.685203	0.052603
H	2.139894	4.139817	-1.102196
H	4.492379	4.594366	-0.421955
C	2.701091	-0.520439	-0.768627
H	3.591894	-1.168400	-0.806033
C	1.927534	-0.852981	0.546814
H	1.970004	0.023192	1.201007
Ni	0.062046	-1.334924	0.313136
B	-2.119299	-0.386487	0.082272
O	-1.862710	-1.829838	0.317842
C	-2.225437	-2.431953	1.562251
H	-3.306540	-2.379675	1.713357
H	-1.923993	-3.482379	1.525512
H	-1.723280	-1.944311	2.405494
C	-0.589507	0.532354	1.003963
H	-1.336669	1.290942	0.771478
C	-0.448131	0.519030	2.536156
H	0.279384	-0.239696	2.861722
H	-1.407464	0.227053	2.986776
C	-0.021130	1.877964	3.111825
H	-0.750774	2.640522	2.802372
H	0.936172	2.168985	2.656516
C	0.108559	1.871900	4.638055
H	0.853921	1.135687	4.965403
H	0.416430	2.851326	5.022180
H	-0.845420	1.610597	5.113599
C	-2.069070	-0.031022	-1.489450
H	-1.138942	-0.369227	-1.970053
C	-3.501985	0.191502	0.682847
H	-3.607730	0.029330	1.766957
C	-3.217221	-0.829511	-2.162791
H	-3.254359	-0.608367	-3.240350
H	-2.972671	-1.897283	-2.072949
C	-2.151351	1.499065	-1.703702
H	-1.207470	1.941084	-1.354155
H	-2.205599	1.728897	-2.778509
C	-4.642434	-0.612707	-0.009460
H	-4.585437	-1.659716	0.316264
H	-5.620202	-0.244191	0.335075
C	-3.632449	1.718117	0.447724
H	-2.960845	2.236620	1.147138
H	-4.643439	2.051327	0.725452
C	-4.615836	-0.596396	-1.553491
H	-5.019689	0.353109	-1.916228
H	-5.303358	-1.367142	-1.928145
C	-3.331078	2.205277	-0.992209
H	-3.135879	3.285907	-0.966878

H	-4.231255	2.090860	-1.602682
H	0.287333	0.992315	0.539422

TS1b

B3LYP-D3 SCF energy:	-1359.67206948 a.u.
B3LYP-D3 enthalpy:	-1359.041183 a.u.
B3LYP-D3 free energy:	-1359.134674 a.u.
M06 SCF energy in solution:	-1359.07317574 a.u.
M06 enthalpy in solution:	-1358.44228926 a.u.
M06 free energy in solution:	-1358.53578026 a.u.
Imaginary frequency:	-205.7657 cm-1

Cartesian coordinates

ATOM	X	Y	Z
C	1.854683	-1.074608	-2.158615
C	2.807239	-1.951727	1.257383
H	2.397046	-0.847165	-3.085512
H	0.893895	-0.550638	-2.214967
H	3.901415	-1.817294	1.271026
H	2.470577	-1.865577	2.299144
C	0.623999	-2.966359	-0.983124
C	0.998544	-3.355146	0.285910
C	1.575919	-2.584920	-2.095355
C	2.442149	-3.358988	0.745820
H	-0.414268	-3.104435	-1.276638
H	0.261192	-3.839888	0.926087
H	2.525693	-3.126199	-1.996561
H	1.127473	-2.900121	-3.044595
H	3.095257	-3.633494	-0.091278
H	2.594410	-4.113037	1.525422
C	2.867568	0.968201	-1.125244
C	4.144469	1.541377	-1.118016
C	1.756519	1.819676	-1.249380
C	4.314705	2.924198	-1.226566
H	5.016612	0.897537	-1.024077
C	1.922579	3.200097	-1.355977
H	0.755812	1.397472	-1.256360
C	3.203271	3.759767	-1.344862
H	5.316536	3.346464	-1.219587
H	1.048452	3.840177	-1.449342
H	3.332513	4.835554	-1.429441
C	2.679580	-0.533167	-0.963544
H	3.674975	-1.003318	-1.030908
C	2.106445	-0.843228	0.456017
H	2.199849	0.070508	1.052252
Ni	0.245446	-1.366439	0.383509
C	-3.649320	1.305541	0.467076
C	-3.643286	1.533716	-1.058155
C	-2.187315	1.350736	-1.532533
H	-4.675799	1.334780	0.854295
H	-3.094483	2.125630	0.958825
H	-2.130619	1.423245	-2.626012
H	-1.567334	2.168328	-1.120520
O	-1.657520	0.092885	-1.158586
O	-3.094431	0.054650	0.823029
C	-4.552042	0.502905	-1.746276
H	-5.588355	0.607836	-1.401819

H	-4.540897	0.641074	-2.834484
H	-4.217548	-0.514227	-1.527142
C	-4.112103	2.960293	-1.369151
H	-4.097944	3.149597	-2.449519
H	-5.138968	3.119977	-1.017944
H	-3.471585	3.710218	-0.887688
B	-1.872387	-0.317442	0.199241
O	-1.677938	-1.779967	0.272360
C	-2.307164	-2.518918	1.319071
H	-3.381211	-2.320441	1.310423
H	-2.123591	-3.581108	1.130269
H	-1.901088	-2.248239	2.300399
C	-0.524388	0.479167	1.248053
H	0.478943	0.811814	0.973829
H	-1.075979	1.398345	1.007764
C	-0.606236	0.246651	2.764502
H	0.017942	-0.614618	3.053437
H	-1.637593	-0.018045	3.031156
C	-0.167878	1.461681	3.594565
H	-0.791090	2.324270	3.316878
H	0.862928	1.729604	3.321242
C	-0.258836	1.223367	5.105223
H	0.380222	0.384381	5.409541
H	0.055548	2.105694	5.674905
H	-1.286646	0.980485	5.403192

TS1c

B3LYP-D3 SCF energy: -1398.99801004 a.u.
B3LYP-D3 enthalpy: -1398.338545 a.u.
B3LYP-D3 free energy: -1398.433512 a.u.
M06 SCF energy in solution: -1398.37490063 a.u.
M06 enthalpy in solution: -1397.71543559 a.u.
M06 free energy in solution: -1397.81040259 a.u.
Imaginary frequency: -164.2279 cm-1

Cartesian coordinates

ATOM	X	Y	Z
C	1.649065	-0.928904	-2.057673
C	2.670415	-2.137664	1.234047
H	2.162171	-0.713855	-3.004195
H	0.770625	-0.275841	-2.013698
H	3.771724	-2.145162	1.182868
H	2.411032	-2.084273	2.299503
C	0.244161	-2.722998	-0.918134
C	0.640226	-3.237000	0.297740
C	1.166800	-2.388991	-2.070053
C	2.096300	-3.449361	0.660913
H	-0.819100	-2.712066	-1.147264
H	-0.115922	-3.663116	0.958648
H	2.036690	-3.058790	-2.076046
H	0.620120	-2.570935	-3.002634
H	2.659183	-3.742231	-0.233678
H	2.196824	-4.270000	1.379380
C	3.034127	0.863950	-0.986249
C	4.385966	1.226894	-0.995880
C	2.070130	1.885457	-1.025076
C	4.771426	2.569624	-1.037519

H	5.146649	0.449251	-0.968061
C	2.451279	3.226319	-1.062389
H	1.015813	1.626658	-1.016323
C	3.804634	3.575653	-1.069380
H	5.827636	2.827557	-1.045353
H	1.687550	4.000243	-1.084890
H	4.101668	4.620708	-1.100221
C	2.613515	-0.594947	-0.891906
H	3.515278	-1.210641	-1.047080
C	2.076004	-0.899929	0.543780
H	2.309311	-0.038790	1.177388
Ni	0.165340	-1.176305	0.561467
C	-3.526313	1.476805	-0.169610
C	-2.341573	1.748527	-1.170655
O	-1.436560	0.665459	-0.878696
O	-2.851792	0.886201	0.958820
B	-1.716922	0.196362	0.438491
O	-1.806150	-1.277732	0.498813
C	-2.533647	-1.886366	1.566098
H	-3.549629	-1.484866	1.588914
H	-2.560136	-2.963613	1.376544
H	-2.054586	-1.702295	2.533985
C	-0.196660	0.659422	1.571547
H	0.675659	1.156413	1.142626
H	-0.911756	1.483134	1.656085
C	0.070483	0.205296	3.022654
H	0.751275	-0.657443	3.066898
H	-0.870516	-0.132870	3.477851
C	0.667464	1.322693	3.892365
H	-0.010457	2.188111	3.875770
H	1.607851	1.661313	3.434760
C	0.921491	0.886938	5.338836
H	1.618547	0.039897	5.377301
H	1.351029	1.699368	5.936431
H	-0.010454	0.570813	5.824570
C	-1.613740	3.072345	-0.890783
H	-0.707314	3.114059	-1.502773
H	-2.236109	3.938003	-1.141568
H	-1.317710	3.148652	0.159263
C	-2.720354	1.673360	-2.649070
H	-3.479844	2.423849	-2.898433
H	-1.835397	1.865724	-3.265006
H	-3.102902	0.684268	-2.910248
C	-4.263070	2.726162	0.307533
H	-3.589415	3.404590	0.835925
H	-4.713401	3.261150	-0.536816
H	-5.063853	2.441196	0.997991
C	-4.531170	0.446989	-0.711488
H	-4.013962	-0.452531	-1.059044
H	-5.209611	0.160450	0.098823
H	-5.129644	0.850331	-1.535496

TS1d

B3LYP-D3 SCF energy: -1164.30020809 a.u.
B3LYP-D3 enthalpy: -1163.796689 a.u.
B3LYP-D3 free energy: -1163.879955 a.u.
M06 SCF energy in solution: -1163.84343749 a.u.

M06 enthalpy in solution: -1163.33991840 a.u.
M06 free energy in solution: -1163.42318440 a.u.
Imaginary frequency: -260.3952 cm-1

Cartesian coordinates

ATOM	X	Y	Z
C	1.981648	-0.897798	-2.137252
C	2.582487	-2.017710	1.305580
H	2.584027	-0.589522	-3.001763
H	1.041306	-0.336211	-2.182144
H	3.648482	-1.833708	1.519313
H	2.070174	-2.052036	2.277009
C	0.682547	-2.929005	-1.236987
C	1.003559	-3.423594	0.012065
C	1.666118	-2.399997	-2.263688
C	2.398268	-3.375054	0.599115
H	-0.322301	-3.127608	-1.606131
H	0.259307	-4.017855	0.540636
H	2.606595	-2.965195	-2.208645
H	1.244729	-2.587183	-3.258026
H	3.143458	-3.478984	-0.199482
H	2.555013	-4.208972	1.291732
C	3.202020	0.897252	-0.819650
C	4.498381	1.218770	-0.394246
C	2.337936	1.950918	-1.156977
C	4.921179	2.545959	-0.299884
H	5.184535	0.416252	-0.131112
C	2.755728	3.279403	-1.063303
H	1.327968	1.736925	-1.493646
C	4.049104	3.583931	-0.633229
H	5.932495	2.767838	0.031798
H	2.067509	4.078039	-1.328583
H	4.374139	4.618442	-0.561393
C	2.746509	-0.553771	-0.840288
H	3.659139	-1.171348	-0.852832
C	1.965725	-0.887936	0.469610
H	1.933329	0.009936	1.093746
Ni	0.140238	-1.484187	0.192250
O	-2.034008	-0.265790	-1.396563
O	-3.223009	-0.322434	0.752294
B	-2.091041	-0.659761	-0.025650
O	-1.755410	-2.083310	0.143619
C	-2.205140	-2.745732	1.332472
H	-3.293908	-2.688458	1.392453
H	-1.886276	-3.789738	1.272317
H	-1.774586	-2.280404	2.226870
C	-0.639848	0.407723	0.759155
H	0.254014	0.830326	0.291720
H	-1.385082	1.136226	0.417509
C	-0.559014	0.494225	2.289618
H	0.198501	-0.203833	2.678530
H	-1.519919	0.170032	2.710615
C	-0.224789	1.901962	2.804702
H	-0.986360	2.605477	2.437265
H	0.728069	2.225941	2.361882
C	-0.141151	1.980431	4.332377
H	0.636339	1.308003	4.717427
H	0.096298	2.994676	4.674366

H	-1.091707	1.684466	4.794097
H	-3.613561	0.487910	0.398676
H	-2.395897	-0.987437	-1.931251

TS2

B3LYP-D3 SCF energy: -948.400652995 a.u.
B3LYP-D3 enthalpy: -948.149366 a.u.
B3LYP-D3 free energy: -948.221299 a.u.
M06 SCF energy in solution: -948.107918991 a.u.
M06 enthalpy in solution: -947.856631996 a.u.
M06 free energy in solution: -947.928564996 a.u.
Imaginary frequency: -26.7184 cm-1

Cartesian coordinates

ATOM	X	Y	Z
Ni	-0.137083	0.217572	0.399826
C	-0.400255	-1.648908	0.749864
C	-1.456698	-1.225988	1.600932
C	-0.654295	-1.850812	-0.634992
C	-2.760050	-1.131400	1.082608
H	-1.274593	-1.070269	2.658204
C	-1.967286	-1.748438	-1.117275
H	0.150123	-2.158362	-1.293751
C	-3.019632	-1.414933	-0.259560
H	-3.565636	-0.830075	1.745461
H	-2.156534	-1.928201	-2.171283
H	-4.031928	-1.346312	-0.644799
I	1.506733	-2.287156	1.608776
C	1.748911	2.266965	0.227506
C	0.404244	1.965194	-0.316068
C	-0.726591	2.091176	0.513769
C	-2.085930	2.121588	-0.073702
H	0.293552	1.957960	-1.398710
H	-0.617302	2.456588	1.532789
O	2.697595	2.123033	-0.731974
O	2.005858	2.574228	1.377760
O	-2.367362	1.986656	-1.250941
O	-3.024900	2.292681	0.894331
C	4.046247	2.331269	-0.286890
H	4.672520	2.183297	-1.167506
H	4.170433	3.343696	0.108504
H	4.306904	1.613154	0.496571
C	-4.380623	2.256151	0.431479
H	-4.998773	2.433902	1.312593
H	-4.555699	3.027467	-0.323943
H	-4.607887	1.278833	-0.007094

TS3

B3LYP-D3 SCF energy: -726.095982178 a.u.
B3LYP-D3 enthalpy: -725.806880 a.u.
B3LYP-D3 free energy: -725.867152 a.u.
M06 SCF energy in solution: -725.772720911 a.u.
M06 enthalpy in solution: -725.483618733 a.u.
M06 free energy in solution: -725.543890733 a.u.
Imaginary frequency: -54.9100 cm-1

Cartesian coordinates

ATOM	X	Y	Z
Ni	-0.518520	0.095974	0.235550
C	-1.454018	-0.831784	-1.511924
C	-1.678404	0.527624	-1.493808
C	-2.876747	1.254985	-0.914121
C	-2.403818	-1.900774	-0.997449
H	-0.608107	-1.187282	-2.097157
H	-1.024419	1.152810	-2.098601
H	-3.681335	1.328516	-1.662568
H	-2.555967	2.282402	-0.705468
H	-3.439879	-1.583078	-1.146765
H	-2.277778	-2.808366	-1.598487
C	-2.374154	0.167974	1.352634
C	-1.838315	-1.097987	1.397258
C	-3.450882	0.642789	0.392277
C	-2.164441	-2.270562	0.491702
H	-2.132420	0.843645	2.171645
H	-1.226523	-1.348259	2.265398
H	-4.128206	-0.182811	0.157864
H	-4.061916	1.404448	0.889810
H	-3.034601	-2.823429	0.878689
H	-1.313135	-2.958295	0.543321
C	1.169561	0.843838	-0.072593
C	1.527725	1.678198	-1.165000
C	0.861816	1.394837	1.214129
C	1.382711	3.043220	-1.026281
H	1.839872	1.235559	-2.105142
C	0.735397	2.811614	1.302706
H	0.993891	0.824594	2.128456
C	0.960974	3.614606	0.202830
H	1.589003	3.689849	-1.875743
H	0.515590	3.258096	2.269886
H	0.872050	4.694060	0.287562
I	2.034576	-1.316715	0.002179

TS4

B3LYP-D3 SCF energy: -726.106899222 a.u.
B3LYP-D3 enthalpy: -725.816030 a.u.
B3LYP-D3 free energy: -725.879288 a.u.
M06 SCF energy in solution: -725.772864165 a.u.
M06 enthalpy in solution: -725.481994943 a.u.
M06 free energy in solution: -725.545252943 a.u.
Imaginary frequency: -161.1615 cm-1

Cartesian coordinates

ATOM	X	Y	Z
Ni	0.571858	0.255349	-0.171473
C	-2.800345	-3.164565	-0.086193
C	-3.591089	-2.320302	-0.760347
C	-3.898519	-0.867975	-0.447670
C	-2.019946	-2.824011	1.158611
H	-2.688449	-4.176654	-0.473260
H	-4.098657	-2.705998	-1.644138
H	-4.758001	-0.809094	0.237853
H	-4.239153	-0.395350	-1.378009
H	-2.633802	-2.189867	1.806806

```
H    -1.819334    -3.734835    1.733722
C    -1.484528    -0.176518    -0.646308
C    -0.607756    -1.268600    -0.380031
C    -2.769328    0.024802    0.138654
C    -0.653426    -2.113346    0.887120
H    -1.580672    0.092732    -1.698646
H    -0.202023    -1.777589    -1.254325
H    -2.599011    -0.186067    1.196531
H    -3.102654    1.064058    0.078069
H    -0.403365    -1.505349    1.764496
H    0.148333    -2.851357    0.803568
C    -0.579929    1.707836    -0.078959
C    -0.570330    2.602436    -1.159832
C    -0.797170    2.177330    1.227373
C    -0.702372    3.973159    -0.921650
H    -0.438223    2.231999    -2.172848
C    -0.924902    3.548965    1.454018
H    -0.849834    1.477387    2.056953
C    -0.879165    4.445782    0.381589
H    -0.672248    4.669783    -1.755369
H    -1.065627    3.916931    2.467003
H    -0.990115    5.511398    0.561493
I    2.823506    -0.759593    -0.042040
```

TS5

```
B3LYP-D3 SCF energy:               -1325.82696518 a.u.
B3LYP-D3 enthalpy:                 -1325.141744 a.u.
B3LYP-D3 free energy:              -1325.235028 a.u.
M06 SCF energy in solution:        -1325.17772707 a.u.
M06 enthalpy in solution:          -1324.49250589 a.u.
M06 free energy in solution:       -1324.58578989 a.u.
Imaginary frequency:               -301.9534 cm-1
```

```
Cartesian coordinates
ATOM  X      Y      Z
Ni    0.351595    -0.622418    0.141749
C    1.259496    0.876774    -0.674413
C    1.006370    2.051037    0.059437
C    1.464342    0.971007    -2.060602
C    0.904549    3.280275    -0.589858
H    0.858194    1.996607    1.134252
C    1.387498    2.205973    -2.705291
H    1.676344    0.073204    -2.635877
C    1.099799    3.361172    -1.972958
H    0.670563    4.174272    -0.018276
H    1.544533    2.265728    -3.779157
H    1.032518    4.321784    -2.475953
O    -1.382606    -0.215690    -0.542830
C    -1.658105    0.333864    -1.815054
H    -2.708902    0.158217    -2.068942
H    -1.458433    1.412159    -1.837611
H    -1.027678    -0.154722    -2.565593
C    3.669452    0.589367    0.487017
C    5.039153    -0.116639    0.673055
C    1.927647    -0.956688    2.530539
C    5.121045    -1.362218    1.534254
C    3.319757    -0.832673    3.235822
```

C	4.407733	-1.677528	2.622357
H	3.361105	1.119965	1.389253
H	5.409006	-0.382072	-0.326542
H	1.375203	-0.026698	2.715986
H	3.631176	0.216827	3.231458
H	3.785635	1.344305	-0.295451
H	5.748399	0.637096	1.049475
H	1.344301	-1.746682	3.012406
H	5.882022	-2.073196	1.212555
H	3.191487	-1.100618	4.290663
H	4.611141	-2.634903	3.100904
C	1.961951	-1.263452	1.040977
H	1.967073	-2.327946	0.793105
C	2.606689	-0.424769	0.085462
H	2.853279	-0.924801	-0.851506
B	-2.438786	-0.006792	0.636333
C	-2.680630	1.594711	0.855312
H	-1.714647	2.109676	0.716050
H	-2.949222	1.783375	1.907322
C	-3.735609	2.306083	-0.009671
H	-3.528003	2.157074	-1.078260
H	-4.721728	1.849660	0.160913
C	-3.836642	3.814558	0.254594
H	-4.057484	3.979638	1.319466
H	-2.853101	4.275007	0.074999
C	-4.895394	4.514270	-0.604713
H	-4.945799	5.590384	-0.397424
H	-4.679026	4.389508	-1.673751
H	-5.891438	4.091534	-0.421241
C	-1.716256	-0.730722	1.922265
H	-0.837998	-0.158433	2.277589
C	-3.744266	-0.932024	0.310246
H	-4.317964	-0.543851	-0.547415
C	-1.230519	-2.136848	1.507337
H	-0.715379	-2.657728	2.328554
H	-0.433731	-2.106012	0.702569
C	-2.678922	-0.770178	3.135277
H	-2.224116	-1.321663	3.974745
H	-2.794838	0.263817	3.484753
C	-3.292708	-2.360785	-0.077612
H	-4.158657	-3.027300	-0.219520
H	-2.791890	-2.297946	-1.052666
C	-4.703890	-0.883824	1.523323
H	-5.046888	0.153362	1.642705
H	-5.608803	-1.483000	1.332470
C	-2.321571	-3.051409	0.909227
H	-2.881337	-3.478100	1.747145
H	-1.842895	-3.907786	0.413609
C	-4.084725	-1.346052	2.858719
H	-4.750714	-1.062194	3.685808
H	-4.048453	-2.440331	2.883983

TS6

B3LYP-D3 SCF energy: -1325.83661866 a.u.
B3LYP-D3 enthalpy: -1325.149335 a.u.
B3LYP-D3 free energy: -1325.243813 a.u.
M06 SCF energy in solution: -1325.19965903 a.u.

M06 enthalpy in solution: -1324.51237537 a.u.
M06 free energy in solution: -1324.60685337 a.u.
Imaginary frequency: -304.4063 cm-1

Cartesian coordinates

ATOM	X	Y	Z
C	-3.303957	-0.023474	-1.382639
C	-1.265326	2.426339	0.360145
H	-4.363496	-0.294171	-1.492525
H	-2.738649	-0.961600	-1.408319
H	-1.867130	3.006540	1.082509
H	-0.221391	2.529037	0.685386
C	-1.407975	1.116781	-2.769000
C	-0.750124	2.164448	-2.086139
C	-2.897772	0.813902	-2.611734
C	-1.423766	3.037207	-1.040937
H	-0.975239	0.792682	-3.717913
H	0.164527	2.568682	-2.526341
H	-3.477876	1.750078	-2.604245
H	-3.233750	0.262088	-3.498794
H	-2.493179	3.156986	-1.263230
H	-0.996533	4.047743	-1.042844
C	-3.903965	-0.130911	1.087010
C	-4.424342	0.573365	2.185010
C	-4.068983	-1.523235	1.066726
C	-5.077546	-0.083583	3.226971
H	-4.312854	1.655542	2.218449
C	-4.721075	-2.187890	2.109056
H	-3.689812	-2.101552	0.230539
C	-5.227570	-1.472503	3.194197
H	-5.474601	0.488734	4.061664
H	-4.834483	-3.268390	2.067887
H	-5.737694	-1.988965	4.002909
C	-3.146353	0.637456	0.005971
H	-3.637932	1.619466	-0.062428
C	-1.685323	0.932826	0.504681
H	-1.724771	0.823236	1.593201
Ni	-0.378854	0.482877	-1.214202
B	2.917475	0.285054	-1.609920
C	4.195537	-0.606462	-1.355408
H	4.448059	-0.746061	-0.295285
C	2.584891	0.440668	-3.138629
H	1.678410	1.034943	-3.300944
C	5.393651	0.136604	-2.020683
H	5.602708	1.034633	-1.419541
C	3.898947	-2.015389	-1.947466
H	3.131486	-2.486437	-1.315416
C	2.342118	-0.966239	-3.748934
H	1.366766	-1.312472	-3.381808
H	2.243580	-0.889527	-4.841907
C	3.791222	1.207667	-3.757630
H	3.787336	2.229961	-3.350066
H	3.648976	1.314059	-4.842997
H	6.298723	-0.485366	-1.958630
H	4.792208	-2.651111	-1.859054
C	3.408251	-2.033365	-3.411967
H	2.996381	-3.027720	-3.630971
H	4.266319	-1.920853	-4.080678

C	5.171881	0.571163	-3.486982
H	5.312728	-0.287934	-4.148483
H	5.957084	1.286760	-3.765236
O	2.170671	0.928108	-0.658392
C	2.540162	0.898985	0.721564
H	2.503664	-0.121020	1.120556
H	3.548298	1.301663	0.867453
H	1.823158	1.519526	1.263426
C	-0.603945	-0.594822	0.534624
H	0.109378	-0.324680	1.316289
H	-1.404187	-1.171283	0.998900
C	0.080200	-1.522854	-0.515421
H	1.137084	-1.645618	-0.253110
H	0.125625	-1.124984	-1.589514
C	-0.585950	-2.899467	-0.644905
H	-1.640580	-2.761040	-0.913550
H	-0.580100	-3.391935	0.337459
C	0.095700	-3.793565	-1.684901
H	-0.388709	-4.774442	-1.749443
H	0.060335	-3.334259	-2.680421
H	1.151326	-3.956758	-1.434461

TS7

B3LYP-D3 SCF energy: -872.475961571 a.u.
B3LYP-D3 enthalpy: -872.058196 a.u.
B3LYP-D3 free energy: -872.123892 a.u.
M06 SCF energy in solution: -872.083034214 a.u.
M06 enthalpy in solution: -871.665268643 a.u.
M06 free energy in solution: -871.730964643 a.u.
Imaginary frequency: -299.1734 cm-1

Cartesian coordinates

ATOM	X	Y	Z
C	0.163540	-1.221609	-1.436556
C	-0.694047	-1.496159	2.092345
H	0.966649	-1.545793	-2.113140
H	-0.157940	-0.238304	-1.799817
H	0.123890	-1.938575	2.687252
H	-1.334797	-0.959816	2.804824
C	-2.325083	-1.845870	-0.910948
C	-2.533885	-2.035517	0.469783
C	-1.013302	-2.211014	-1.607037
C	-1.501157	-2.615759	1.419933
H	-3.200618	-1.797745	-1.559704
H	-3.568902	-2.052299	0.823173
H	-0.679290	-3.209310	-1.284693
H	-1.203673	-2.292410	-2.684164
H	-0.814383	-3.283273	0.882722
H	-1.983627	-3.229016	2.191202
C	2.144950	-0.357824	-0.099066
C	3.159838	-0.717028	0.802530
C	2.403644	0.693942	-0.989319
C	4.384098	-0.049832	0.821862
H	2.982656	-1.534014	1.499425
C	3.627935	1.367628	-0.973783
H	1.648722	0.993340	-1.708365
C	4.623384	1.000325	-0.068169

H	5.153221	-0.353197	1.527646
H	3.801675	2.180003	-1.675018
H	5.576498	1.522150	-0.058116
C	0.801846	-1.081678	-0.030837
H	1.028486	-2.104353	0.305321
C	-0.060817	-0.452671	1.127257
H	0.666806	0.038643	1.779555
Ni	-1.994026	-0.206115	0.124504
C	-0.715569	1.257194	0.787957
H	-0.915447	1.593743	1.808908
H	0.270825	1.614949	0.494303
C	-1.743640	1.898780	-0.201947
H	-2.431860	2.541111	0.358782
H	-2.468610	1.186717	-0.748344
C	-1.072932	2.698482	-1.328696
H	-0.398380	2.033199	-1.882041
H	-0.440436	3.478487	-0.882922
C	-2.080530	3.327393	-2.294317
H	-1.575814	3.899648	-3.080828
H	-2.693120	2.556827	-2.778789
H	-2.759457	4.008693	-1.766707

TS8

B3LYP-D3 SCF energy: -1406.87487028 a.u.
B3LYP-D3 enthalpy: -1406.302177 a.u.
B3LYP-D3 free energy: -1406.399246 a.u.
M06 SCF energy in solution: -1406.31832653 a.u.
M06 enthalpy in solution: -1405.74563325 a.u.
M06 free energy in solution: -1405.84270225 a.u.
Imaginary frequency: -304.6211 cm-1

Cartesian coordinates

ATOM	X	Y	Z
C	-2.362485	-1.028714	-0.728304
C	-1.980476	-0.444326	-1.945687
C	-3.646759	-0.753597	-0.239630
C	-2.848398	0.395707	-2.643242
H	-0.991963	-0.625120	-2.354696
C	-4.526689	0.072588	-0.942183
H	-3.962660	-1.194058	0.703791
C	-4.129039	0.650016	-2.149361
H	-2.513498	0.867107	-3.561228
H	-5.518756	0.267286	-0.542582
H	-4.804889	1.302833	-2.695040
C	-1.400924	-3.365009	-0.603511
C	-0.707226	-4.464564	0.219173
C	0.987677	-1.619577	-0.779201
C	0.797814	-4.626381	0.338695
C	2.172993	-2.480167	-0.342268
C	1.898307	-3.887333	0.128753
H	-0.972257	-3.310505	-1.610219
H	-1.107188	-4.426324	1.246786
H	0.506506	-2.041732	-1.661353
H	2.900765	-2.532146	-1.165827
H	-2.442138	-3.679590	-0.748406
H	-1.068927	-5.425472	-0.173634
H	1.458721	-0.680255	-1.215243

H	1.020705	-5.622787	0.726408
H	2.698058	-1.951799	0.468923
H	2.831455	-4.400353	0.369480
C	-0.045806	-1.415980	0.363779
H	0.352163	-1.957685	1.221244
C	-1.453899	-1.965239	0.054967
H	-1.937257	-2.120248	1.028472
Ni	0.561612	0.412575	-0.216773
C	-0.776710	2.848675	-0.746866
C	0.526414	2.379375	-0.239175
C	1.488028	1.864934	-1.144600
C	2.898550	1.753510	-0.723598
H	0.837867	2.760350	0.730455
H	1.288485	1.884644	-2.213666
O	-1.583859	3.221090	0.285137
O	-1.131592	2.887939	-1.911998
O	3.339607	1.959934	0.395820
O	3.683456	1.325899	-1.750674
C	-2.909557	3.621592	-0.092308
H	-3.398324	3.926243	0.834506
H	-2.873908	4.455839	-0.799226
H	-3.443243	2.785942	-0.552559
C	5.061740	1.116732	-1.414899
H	5.546347	0.803660	-2.340847
H	5.514111	2.038179	-1.037117
H	5.159670	0.339851	-0.649524
C	-0.360488	0.100330	1.621488
H	-0.918802	1.013089	1.389762
H	-1.091604	-0.570453	2.072361
C	0.796024	0.339895	2.592916
H	1.559013	0.994888	2.155041
H	1.301231	-0.614433	2.802596
C	0.310440	0.955163	3.916578
H	-0.453465	0.303127	4.364520
H	-0.185969	1.911877	3.703346
C	1.453877	1.177545	4.912164
H	2.214886	1.844695	4.489742
H	1.092362	1.625737	5.844822
H	1.946487	0.229926	5.163799

X-Ray Crystallography of 4s’

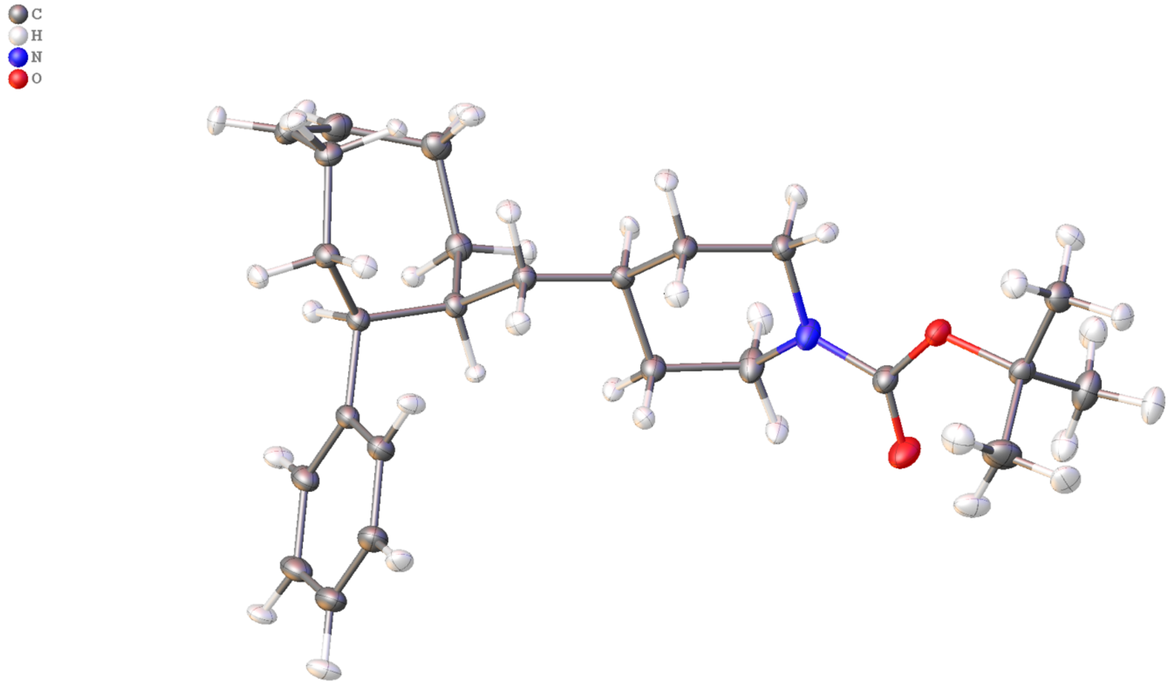

Experimental Summary

The single crystal X-ray diffraction studies were carried out on a Bruker D8 Venture Ultra diffractometer equipped with Mo $K_{□}$ radiation (□ = 0.71073 Å). Crystals of the subject compound were used as received (grown from DCM / Hexanes).

A 0.140 x 0.125 x 0.125 mm crystal was mounted on a Cryoloop with Paratone oil.

Data were collected in a nitrogen gas stream at 100(2) K using □ and □ scans. Crystal-to-detector distance was 50 mm using exposure time 3.0 s with a scan width of 0.70°. Data collection was 99.9 % complete to 25.242° in □.

A total of 69090 reflections were collected. 6512 reflections were found to be symmetry independent, with a R_{int} of 0.0556. Indexing and unit cell refinement indicated a **Monoclinic** lattice. The space group was found to be ***Cc***. The data were integrated using the Bruker SAINT Software program and scaled using the SADABS software program. Solution by direct methods (SHELXT) produced a complete phasing model consistent with the proposed structure.

All nonhydrogen atoms were refined anisotropically by full-matrix least-squares (SHELXL-2014). All carbon bonded hydrogen atoms were placed using a riding model. Their positions were constrained relative to their parent atom using the appropriate HFIX command in SHELXL-2014.

Crystallographic data are summarized in **Table S6.**

Notes: Excellent data and refinement

Absolute structure parameter 0.1(2) Conclusive

NoSpherA2 Scattering Factors Used

Table S6. Crystal data and structure refinement for **4s'**.

Identification code	Engle757	
Empirical formula	C25 H37 N O2	
Formula weight	383.578	
Temperature	100.00 K	
Wavelength	0.71073 Å	
Crystal system	Monoclinic	
Space group	Cc	
Unit cell dimensions	a = 6.0167(2) Å	a= 90°.
	b = 49.5442(18) Å	b= 106.801(1)°.
	c = 7.8157(3) Å	g = 90°.
Volume	2230.35(14) $Å^3$	
Z	4	
Density (calculated)	1.142 Mg/m^3	
Absorption coefficient	0.071 mm^{-1}	
F(000)	840.462	
Crystal size	0.14 x 0.125 x 0.125 mm^3	
Theta range for data collection	2.47 to 30.10°.	
Index ranges	-8<=h<=8, -69<=k<=69, -10<=l<=11	
Reflections collected	69090	
Independent reflections	6512 [R(int) = 0.0556]	
Completeness to theta = 25.2417°	99.90 %	
Absorption correction	Semi-empirical from equivalents	
Max. and min. transmission	0.6469 and 0.6054	
Refinement method	Full-matrix least-squares on F^2	
Data / restraints / parameters	6512 / 584 / 586	
Goodness-of-fit on F^2	1.1030	
Final R indices [I>2sigma(I)]	R1 = 0.0190, wR2 = 0.0325	
R indices (all data)	R1 = 0.0208, wR2 = 0.0332	
Absolute structure parameter	0.1(2)	
Largest diff. peak and hole	0.0969 and -0.0865 e.$Å^{-3}$	

Table S7. Bond lengths [Å] and angles [°] for **4s'**.

O(1)-C(21)	1.3472(6)	C(12)-C(13)	1.3939(8)
O(1)-C(22)	1.4652(6)	C(13)-H(13)	1.078(7)
O(2)-C(21)	1.2209(6)	C(13)-C(14)	1.3967(7)
N(1)-C(18)	1.4611(6)	C(14)-H(14)	1.062(7)
N(1)-C(19)	1.4595(6)	C(15)-H(15a)	1.089(6)
N(1)-C(21)	1.3578(6)	C(15)-H(15b)	1.103(6)
C(1)-H(1)	1.089(6)	C(15)-C(16)	1.5331(6)
C(1)-C(2)	1.5693(6)	C(16)-H(16)	1.100(6)
C(1)-C(8)	1.5442(6)	C(16)-C(17)	1.5341(6)
C(1)-C(9)	1.5225(6)	C(16)-C(20)	1.5282(6)
C(2)-H(2)	1.110(6)	C(17)-H(17a)	1.098(6)
C(2)-C(3)	1.5468(7)	C(17)-H(17b)	1.105(7)
C(2)-C(15)	1.5391(6)	C(17)-C(18)	1.5276(7)
C(3)-H(3a)	1.087(6)	C(18)-H(18a)	1.078(6)
C(3)-H(3b)	1.094(6)	C(18)-H(18b)	1.074(6)
C(3)-C(4)	1.5409(7)	C(19)-H(19a)	1.112(7)
C(4)-H(4a)	1.093(7)	C(19)-H(19b)	1.081(7)
C(4)-H(4b)	1.081(7)	C(19)-C(20)	1.5238(7)
C(4)-C(5)	1.5102(7)	C(20)-H(20a)	1.093(6)
C(5)-H(5)	1.076(7)	C(20)-H(20b)	1.082(6)
C(5)-C(6)	1.3365(8)	C(22)-C(23)	1.5207(7)
C(6)-H(6)	1.083(6)	C(22)-C(24)	1.5182(7)
C(6)-C(7)	1.5024(7)	C(22)-C(25)	1.5188(7)
C(7)-H(7a)	1.088(6)	C(23)-H(23a)	1.089(7)
C(7)-H(7b)	1.069(6)	C(23)-H(23b)	1.059(7)
C(7)-C(8)	1.5422(7)	C(23)-H(23c)	1.085(7)
C(8)-H(8a)	1.094(6)	C(24)-H(24a)	1.079(7)
C(8)-H(8b)	1.089(6)	C(24)-H(24b)	1.057(8)
C(9)-C(10)	1.4019(7)	C(24)-H(24c)	1.098(8)
C(9)-C(14)	1.3999(7)	C(25)-H(25a)	1.086(8)
C(10)-H(10)	1.066(7)	C(25)-H(25b)	1.072(8)
C(10)-C(11)	1.3958(7)	C(25)-H(25c)	1.104(8)
C(11)-H(11)	1.090(7)		
C(11)-C(12)	1.3936(8)	C(22)-O(1)-C(21)	120.45(4)
C(12)-H(12)	1.081(7)	C(19)-N(1)-C(18)	115.39(4)

C(21)-N(1)-C(18)	124.72(4)	C(8)-C(7)-H(7b)	107.4(4)
C(21)-N(1)-C(19)	119.05(4)	C(7)-C(8)-C(1)	116.76(4)
C(2)-C(1)-H(1)	105.7(3)	H(8a)-C(8)-C(1)	105.4(3)
C(8)-C(1)-H(1)	107.7(3)	H(8a)-C(8)-C(7)	110.0(4)
C(8)-C(1)-C(2)	117.78(4)	H(8b)-C(8)-C(1)	110.8(4)
C(9)-C(1)-H(1)	105.5(3)	H(8b)-C(8)-C(7)	107.9(3)
C(9)-C(1)-C(2)	109.34(4)	H(8b)-C(8)-H(8a)	105.4(5)
C(9)-C(1)-C(8)	109.99(4)	C(10)-C(9)-C(1)	122.60(4)
H(2)-C(2)-C(1)	104.1(3)	C(14)-C(9)-C(1)	119.63(4)
C(3)-C(2)-C(1)	114.24(4)	C(14)-C(9)-C(10)	117.75(4)
C(3)-C(2)-H(2)	103.4(3)	H(10)-C(10)-C(9)	120.8(4)
C(15)-C(2)-C(1)	113.41(4)	C(11)-C(10)-C(9)	121.14(5)
C(15)-C(2)-H(2)	106.6(3)	C(11)-C(10)-H(10)	118.0(4)
C(15)-C(2)-C(3)	113.75(4)	H(11)-C(11)-C(10)	118.7(4)
H(3a)-C(3)-C(2)	108.0(3)	C(12)-C(11)-C(10)	120.35(5)
H(3b)-C(3)-C(2)	108.0(3)	C(12)-C(11)-H(11)	120.9(4)
H(3b)-C(3)-H(3a)	106.8(5)	H(12)-C(12)-C(11)	120.9(4)
C(4)-C(3)-C(2)	118.71(4)	C(13)-C(12)-C(11)	119.27(5)
C(4)-C(3)-H(3a)	107.2(4)	C(13)-C(12)-H(12)	119.9(4)
C(4)-C(3)-H(3b)	107.6(3)	H(13)-C(13)-C(12)	120.6(4)
H(4a)-C(4)-C(3)	110.0(3)	C(14)-C(13)-C(12)	120.10(5)
H(4b)-C(4)-C(3)	106.9(4)	C(14)-C(13)-H(13)	119.3(4)
H(4b)-C(4)-H(4a)	105.2(5)	C(13)-C(14)-C(9)	121.39(5)
C(5)-C(4)-C(3)	113.38(4)	H(14)-C(14)-C(9)	120.3(3)
C(5)-C(4)-H(4a)	110.9(4)	H(14)-C(14)-C(13)	118.3(3)
C(5)-C(4)-H(4b)	110.1(4)	H(15a)-C(15)-C(2)	110.8(3)
H(5)-C(5)-C(4)	115.6(4)	H(15b)-C(15)-C(2)	108.8(3)
C(6)-C(5)-C(4)	124.83(5)	H(15b)-C(15)-H(15a)	104.1(5)
C(6)-C(5)-H(5)	119.5(4)	C(16)-C(15)-C(2)	115.16(4)
H(6)-C(6)-C(5)	120.6(4)	C(16)-C(15)-H(15a)	108.4(3)
C(7)-C(6)-C(5)	123.27(5)	C(16)-C(15)-H(15b)	108.9(3)
C(7)-C(6)-H(6)	116.0(4)	H(16)-C(16)-C(15)	108.7(3)
H(7a)-C(7)-C(6)	108.3(4)	C(17)-C(16)-C(15)	110.60(4)
H(7b)-C(7)-C(6)	110.3(4)	C(17)-C(16)-H(16)	107.2(3)
H(7b)-C(7)-H(7a)	106.2(5)	C(20)-C(16)-C(15)	113.04(4)
C(8)-C(7)-C(6)	113.31(4)	C(20)-C(16)-H(16)	107.8(3)
C(8)-C(7)-H(7a)	111.0(3)	C(20)-C(16)-C(17)	109.19(4)

H(17a)-C(17)-C(16)	109.5(3)
H(17b)-C(17)-C(16)	109.3(3)
H(17b)-C(17)-H(17a)	107.8(5)
C(18)-C(17)-C(16)	112.37(4)
C(18)-C(17)-H(17a)	108.8(3)
C(18)-C(17)-H(17b)	108.9(3)
C(17)-C(18)-N(1)	110.30(4)
H(18a)-C(18)-N(1)	109.7(3)
H(18a)-C(18)-C(17)	111.4(3)
H(18b)-C(18)-N(1)	108.3(4)
H(18b)-C(18)-C(17)	111.0(4)
H(18b)-C(18)-H(18a)	106.1(5)
H(19a)-C(19)-N(1)	108.6(4)
H(19b)-C(19)-N(1)	107.6(3)
H(19b)-C(19)-H(19a)	106.2(6)
C(20)-C(19)-N(1)	110.90(4)
C(20)-C(19)-H(19a)	110.0(4)
C(20)-C(19)-H(19b)	113.3(4)
C(19)-C(20)-C(16)	111.06(4)
H(20a)-C(20)-C(16)	107.6(3)
H(20a)-C(20)-C(19)	110.1(3)
H(20b)-C(20)-C(16)	111.5(3)
H(20b)-C(20)-C(19)	108.6(3)
H(20b)-C(20)-H(20a)	107.9(5)
O(2)-C(21)-O(1)	124.75(4)
N(1)-C(21)-O(1)	111.34(4)
N(1)-C(21)-O(2)	123.91(4)
C(23)-C(22)-O(1)	102.60(4)
C(24)-C(22)-O(1)	110.89(4)
C(24)-C(22)-C(23)	110.21(4)
C(25)-C(22)-O(1)	109.79(4)
C(25)-C(22)-C(23)	110.15(4)
C(25)-C(22)-C(24)	112.74(5)
H(23a)-C(23)-C(22)	108.5(4)
H(23b)-C(23)-C(22)	110.2(4)
H(23b)-C(23)-H(23a)	112.1(6)
H(23c)-C(23)-C(22)	110.5(4)
H(23c)-C(23)-H(23a)	108.5(6)
H(23c)-C(23)-H(23b)	107.1(6)
H(24a)-C(24)-C(22)	112.3(4)
H(24b)-C(24)-C(22)	110.3(5)
H(24b)-C(24)-H(24a)	108.2(7)
H(24c)-C(24)-C(22)	108.7(4)
H(24c)-C(24)-H(24a)	109.6(6)
H(24c)-C(24)-H(24b)	107.5(6)
H(25a)-C(25)-C(22)	111.4(4)
H(25b)-C(25)-C(22)	110.0(4)
H(25b)-C(25)-H(25a)	109.9(6)
H(25c)-C(25)-C(22)	108.7(4)
H(25c)-C(25)-H(25a)	107.0(6)
H(25c)-C(25)-H(25b)	109.8(6)

References

[1] You, F.; Zhang, X.; Wang, X.; Guo, G.; Wang, Q.; Song, H.; Qu, R.; Lian, Z. Mechanochemical Vicinal Dibromination of Unactivated Alkenes and Alkynes Using Piezoelectric Material as a Redox Catalyst. *Org. Lett.* **2024**, *26*, 4240–4245.

[2] Liu, G.; Qi, X.; Yu, F.; Chen, P. Palladium-Catalyzed Intermolecular Oxidative Fluorocarbonylation of Unactivated Alkenes: Efficient Access of β-Fluorocarboxylic Esters. *Angew. Chem. Int. Ed.* **2017**, *56*, 12692–12696.

[3] Bratz, M.; Bullock, W. H.; Overman, L. E.; Takemoto, T. Total Synthesis of (+)-Laurencin. Use of Acetal-Vinyl Sulfide Cyclizations for Forming Highly Functionalized Eight-Membered Cyclic Ethers. *J. Am. Chem. Soc.* **1995**, *117*, 5958–5966.

[4] Migliorini, F.; Dei, F.; Calamante, M.; Maramai, S.; Petricci, E. Micellar Catalysis for Sustainable Hydroformylation. *ChemCatChem* **2021**, *13*, 2794–2806.

[5] Enders, M.; Görling, B.; Braun, A. B.; Seltenreich, J. E.; Reichenbach, L. F.; Rissanen, K.; Nieger, M.; Luy, B.; Schepers, U.; Bräse, S. Cytotoxicity and NMR Studies of Platinum Complexes with Cyclooctadiene Ligands. *Organometallics* **2014**, *33*, 4027–4034.

[6] Wethman, R.; Derosa, J.; Tran, V. T.; Kang, T.; Apolinar, O.; Abraham, A.; Kleinmans, R.; Wisniewski, S. R.; Coombs, J. R.; Engle, K. M. An Under-Appreciated Source of Reproducibility Issues in Cross-Coupling: Solid-State Decomposition of Primary Sodium Alkoxides in Air. *ACS Catal.* **2021**, *11*, 502–508.

[7] Fulmer, G. R.; Miller, A. J. M.; Sherden, N. H.; Gottlieb, H. E.; Nudelman, A.; Stoltz, B. M.; Bercaw, J. E.; Goldberg, K. I. NMR Chemical Shifts of Trace Impurities: Common Laboratory Solvents, Organics, and Gases in Deuterated Solvents Relevant to the Organometallic Chemist. *Organometallics* **2010**, *29*, 2176–2179.

[8] Tran, V. T.; Ravn, A. K.; Rubel, C. Z.; Xu, M.; Fu, Y.; Wagner, E. M.; Wisniewski, S. R.; Liu, P.; Gutekunst, W. R.; Engle, K. M. Ni-Catalysed Dicarbofunctionalization for the Synthesis of Sequence-Encoded Cyclooctene Monomers. *Nat. Synth.* **2024**, *3*, 1369–1376.

[9] Kramer, G. W.; Brown, H. C. Organoboranes. *J. Organomet. Chem* **1974**, *73*, 1–15.

[10] CCDC 2472616 (**4s'**) contains the supplementary crystallographic data for this paper. These data can be obtained free of charge from The Cambridge Crystallographic Data Centre via www.ccdc.cam.ac.uk/data_request/cif.

[11] Love, J. A.; Morgan, J. P.; Trnka, T. M.; Grubbs R. H. A Practical and Highly Active Ruthenium-Based Catalyst that Effects the Cross Metathesis of Acrylonitrile. *Angew. Chem. Int. Ed.* **2002**, *41*, 4035–4037.

[12] Kobayashi, S.; Lu, C.; Hoye, T. R.; Hillmyer, M. A. Controlled Polymerization of a Cyclic Diene Prepared from the Ring-Closing Metathesis of a Naturally Occurring Monoterpene. *J. Am. Chem. Soc.* **2009**, *131*, 7960–7961.

[13] Frisch, M. J., Trucks, G. W., Schlegel, H. B., Scuseria, G. E., Robb, M. A., Cheeseman, J. R., Scalmani, G., Barone, V., Petersson, G. A., Nakatsuji, H., Li, X., Caricato, M., Marenich, A. V., Bloino, J., Janesko, B. G., Gomperts, R., Mennucci, B., Hratchian, H. P., Ortiz, J. V., Izmaylov, A. F., Sonnenberg, J. L., Williams-Young, D., Ding, F., Lipparini, F., Egidi, F., Goings, J., Peng, B., Petrone, A., Henderson, T., Ranasinghe, D., Zakrzewski, V. G., Gao, J., Rega, N., Zheng, G., Liang, W., Hada, M., Ehara, M., Toyota, K., Fukuda, R., Hasegawa, J., Ishida, M.,

Nakajima, T., Honda, Y., Kitao, O., Nakai, H., Vreven, T., Throssell, K., Montgomery, J. A., Peralta, J. E., Ogliaro, F., Bearpark, M. J., Heyd, J. J., Brothers, E. N., Kudin, K. N., Staroverov, V. N., Keith, T. A., Kobayashi, R., Normand, J., Raghavachari, K., Rendell, A. P., Burant, J. C., Iyengar, S. S., Tomasi, J., Cossi, M., S155 Millam, J. M., Klene, M., Adamo, C., Cammi, R., Ochterski, J. W., Martin, R. L., Morokuma, K., Farkas, O., Foresman, J. B., Fox, D. J., Gaussian 16 Rev. C.01, Wallingford, CT, 2016.

[14] a) Lee, C., Yang, W., Parr, R. G. Development of the Colle-Salvetti Correlation-Energy Formula into a Functional of the Electron Density. *Phys. Rev. B* **1988**, *37*, 785–789. b) Becke, A. D. Density-Functional Thermochemistry. III. The Role of Exact Exchange. *J.Chem. Phys.* **1993**, *98*, 5648–5652.

[15] Grimme, S., Antony, J., Ehrlich, S., Krieg, H. A Consistent and Accurate Ab Initio Parametrization of Density Functional Dispersion Correction (DFT-D) for the 94 Elements H-Pu. *J. Chem. Phys.* **2010**, *132*, 154104.

[16] a) Igelmann, G., Stoll, H. & Preuss, H. Pseudopotentials for Main Group Elements (Iiia through Viia). *Mol. Phys.* **1988**, *65*, 1321–1328. b) Bergner, A., Dolg, M., Kuchle, W., Stoll, H. & Preuss, H. Ab-Initio Energy-Adjusted Pseudopotentials for Elements of Groups 13-17. *Mol. Phys.* **1992**, *80*, 1431–1441.

[17] Zhao, Y. & Truhlar, D. G. The M06 Suite of Density Functionals for Main Group Thermochemistry, Thermochemical Kinetics, Noncovalent Interactions, Excited States, and Transition Elements: Two New Functionals and Systematic Testing of Four M06-Class Functionals and 12 Other Functionals. *Theor. Chem. Acc.* **2008**, *120*, 215–241.

[18] Marenich, A. V, Cramer, C. J. & Truhlar, D. G. Universal Solvation Model Based on Solute Electron Density and on a Continuum Model of the Solvent Defined by the Bulk Dielectric Constant and Atomic Surface Tensions. *J. Phys. Chem. B* **2009**, *113*, 6378–6396.

[19] Grimme, S. Supramolecular Binding Thermodynamics by Dispersion-Corrected Density Functional Theory. *Chem. Eur. J.* **2012**, *18*, 9955–9964.

[20] Luchini, G., Alegre-Requena, J. V., Funes-Ardoiz, I. & Paton, R. S. GoodVibes: Automated Thermochemistry for Heterogeneous Computational Chemistry Data. *F1000Research* **2020**, *9*, 291.

[21] CYLview20; Legault, C. Y., Université de Sherbrooke, 2020 (http://www.cylview.org).

NMR Spectra

^{1}H NMR (400 MHz, C_6D_6) of **3a** in 1,4-dioxane (0.4M):

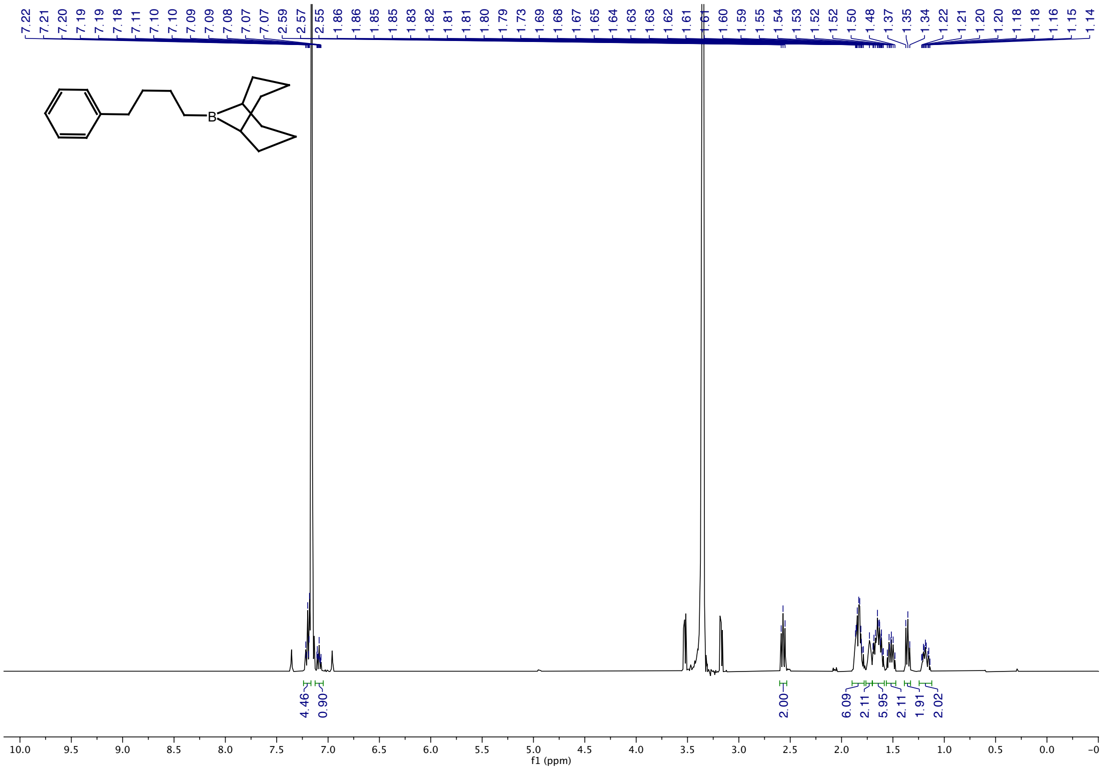

^{1}H NMR (400 MHz, C_6D_6) of **S3k** in 1,4-dioxane (0.4 M):

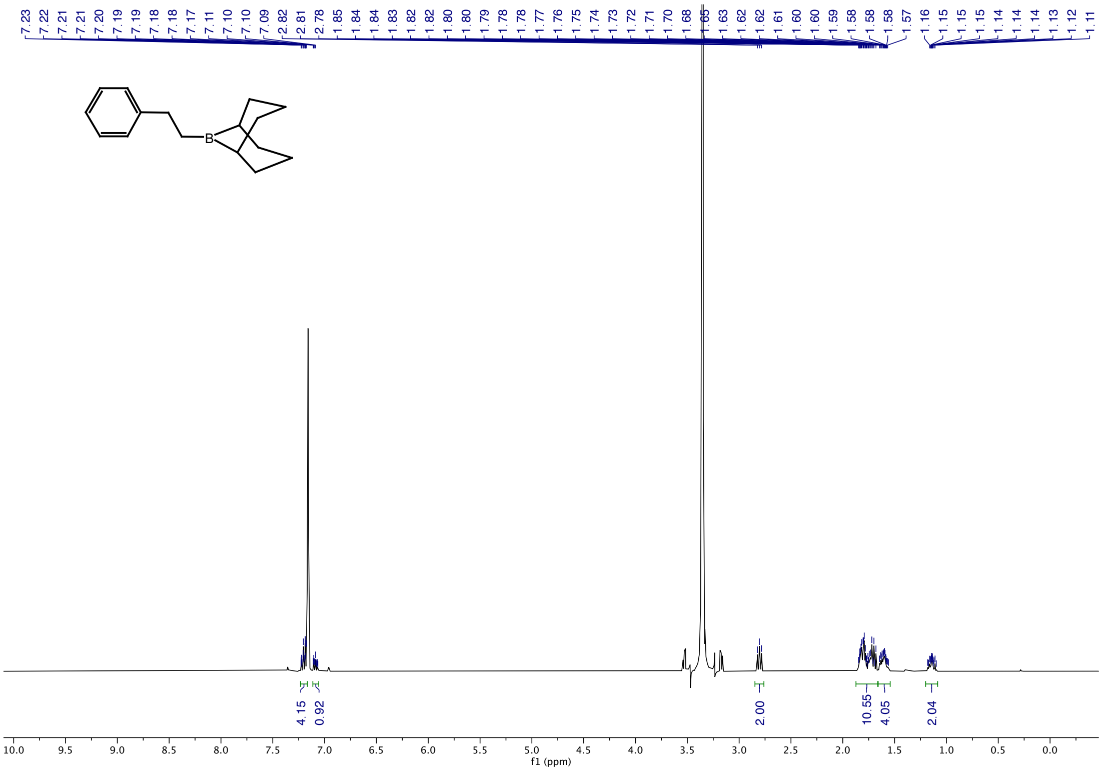

^{1}H NMR (400 MHz, C_6D_6) of **S3l** in 1,4-dioxane (0.4 M):

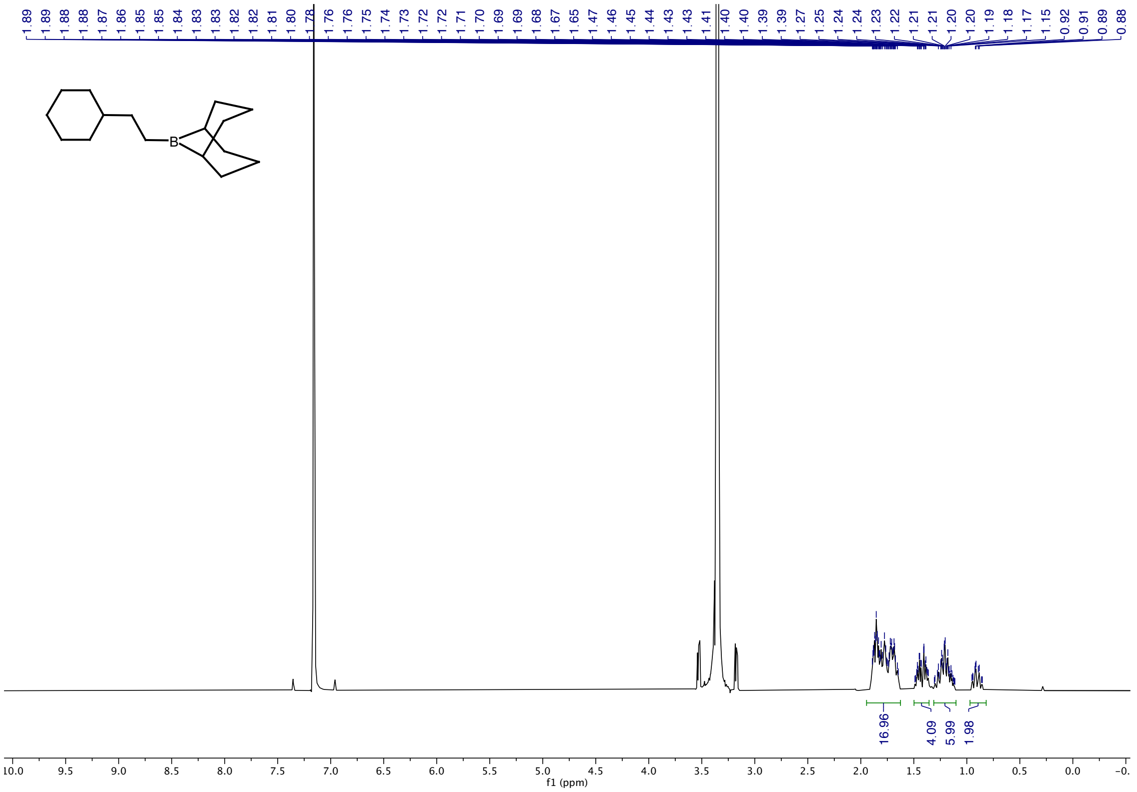

^{1}H NMR (400 MHz, C_6D_6) of **S3m** in 1,4-dioxane (0.4M):

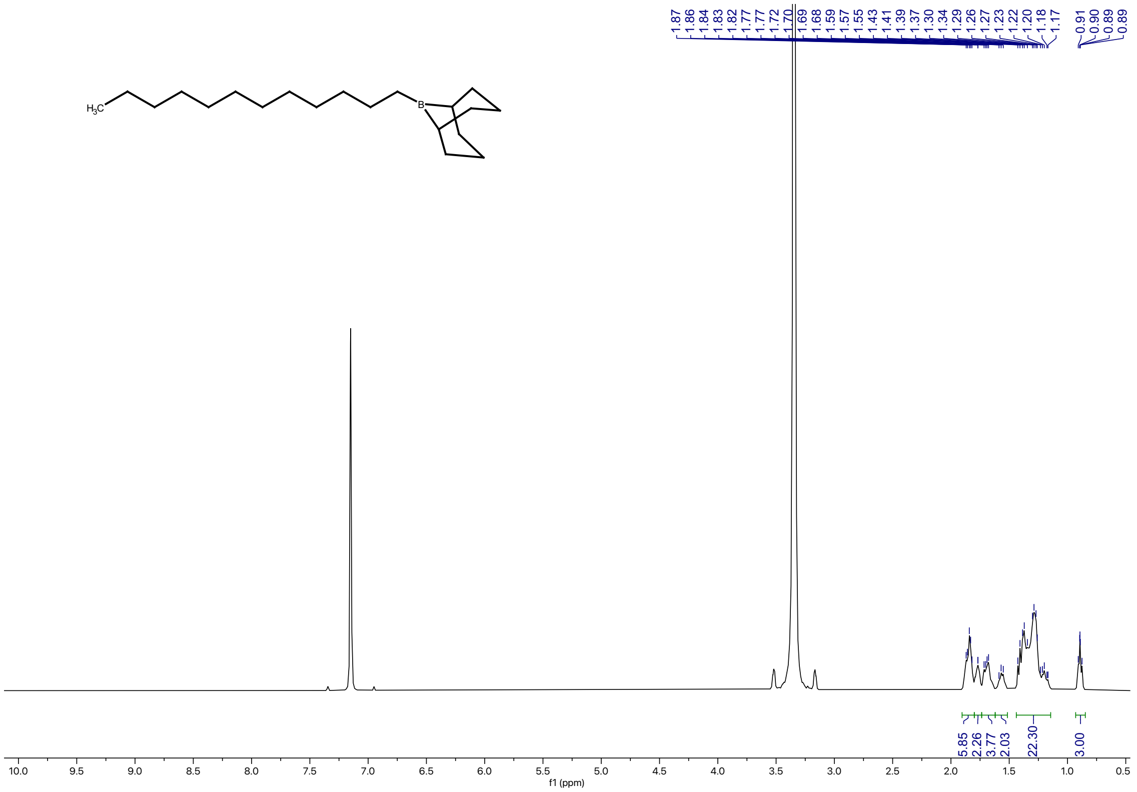

^{1}H NMR (400 MHz, C_6D_6) of **S3n** in 1,4-dioxane (0.4M):

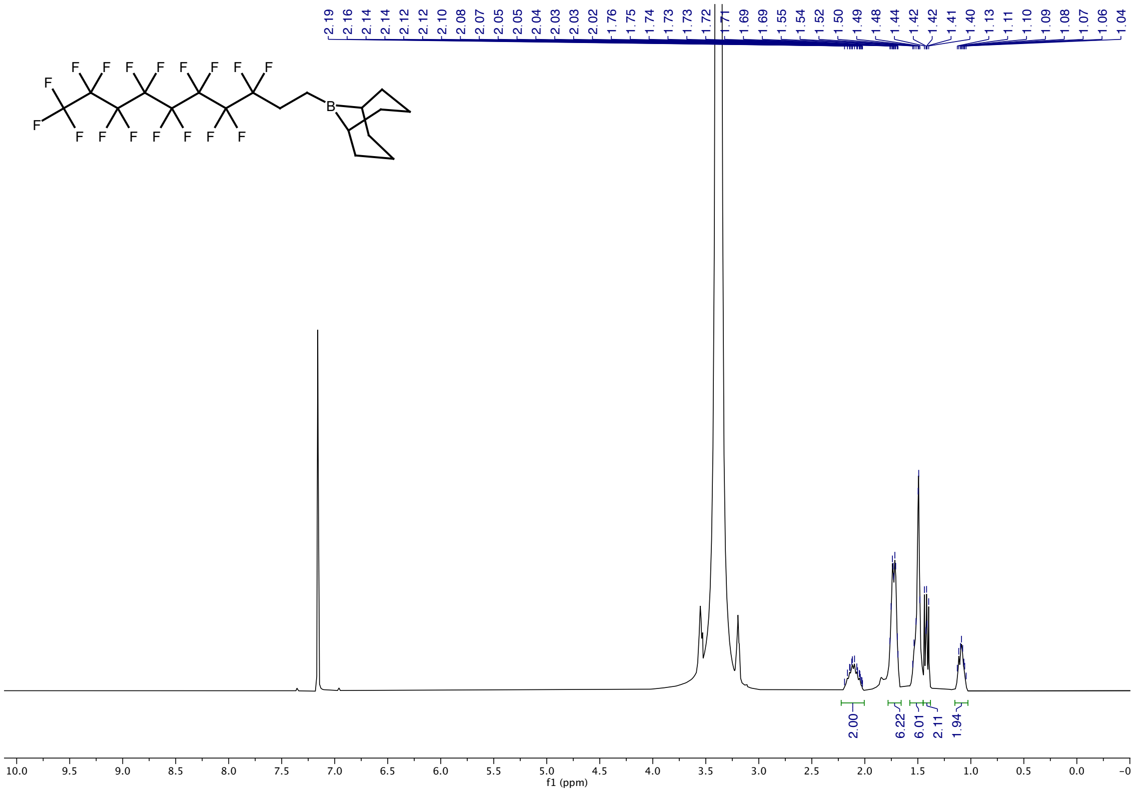

^{19}F NMR (400 MHz, C_6D_6) of **S3n** in 1,4-dioxane (0.4M):

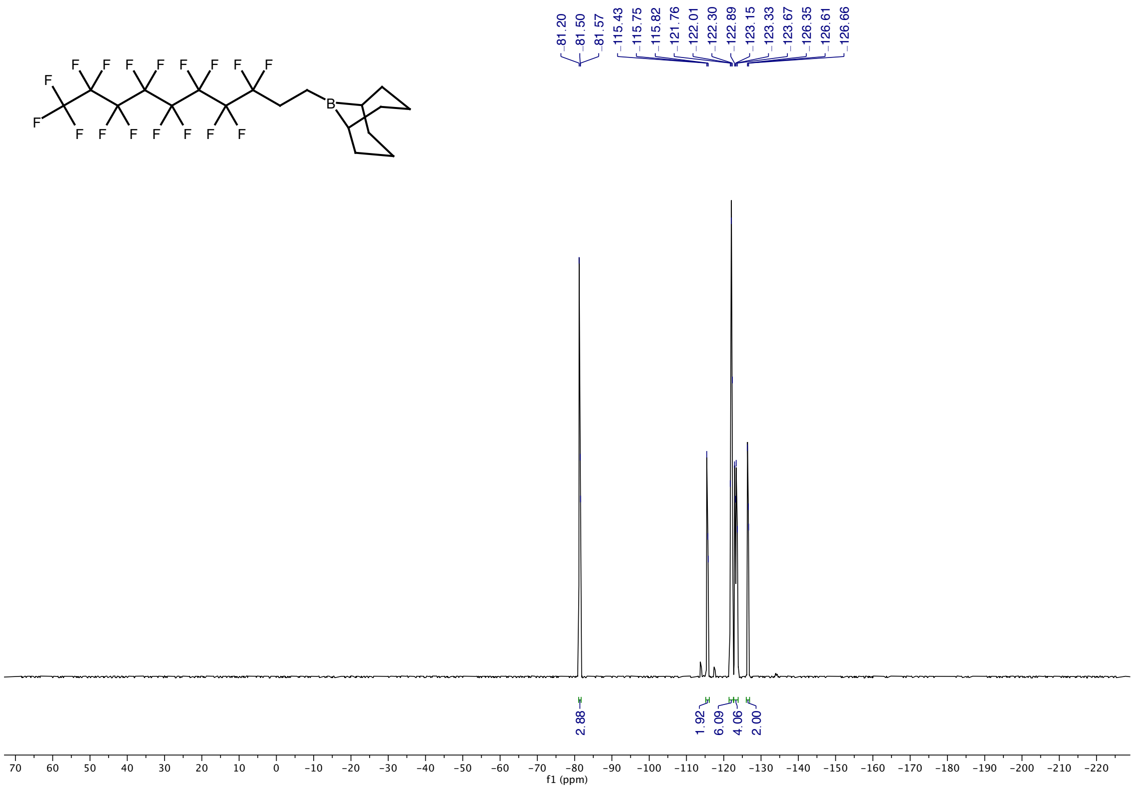

^{1}H NMR (400 MHz, C_6D_6) of **S3o** in 1,4-dioxane (0.4 M):

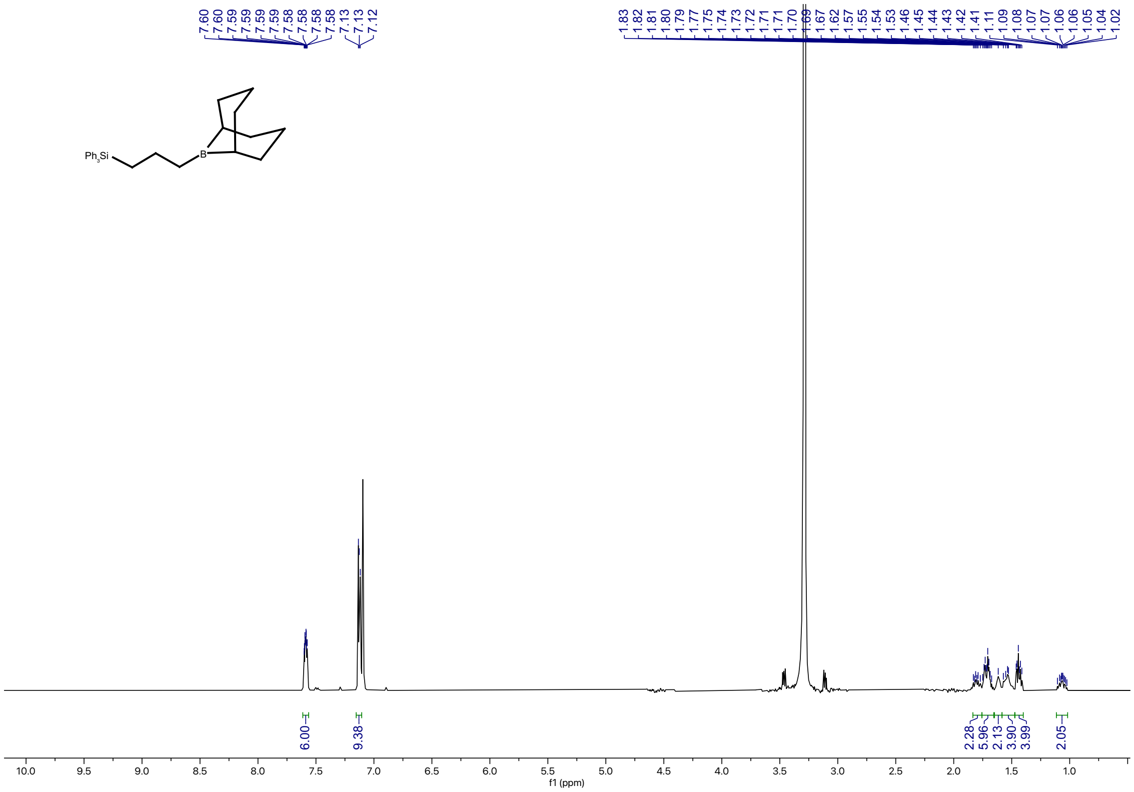

^{1}H NMR (400 MHz, C_6D_6) of **S3p** in 1,4-dioxane (0.4 M):

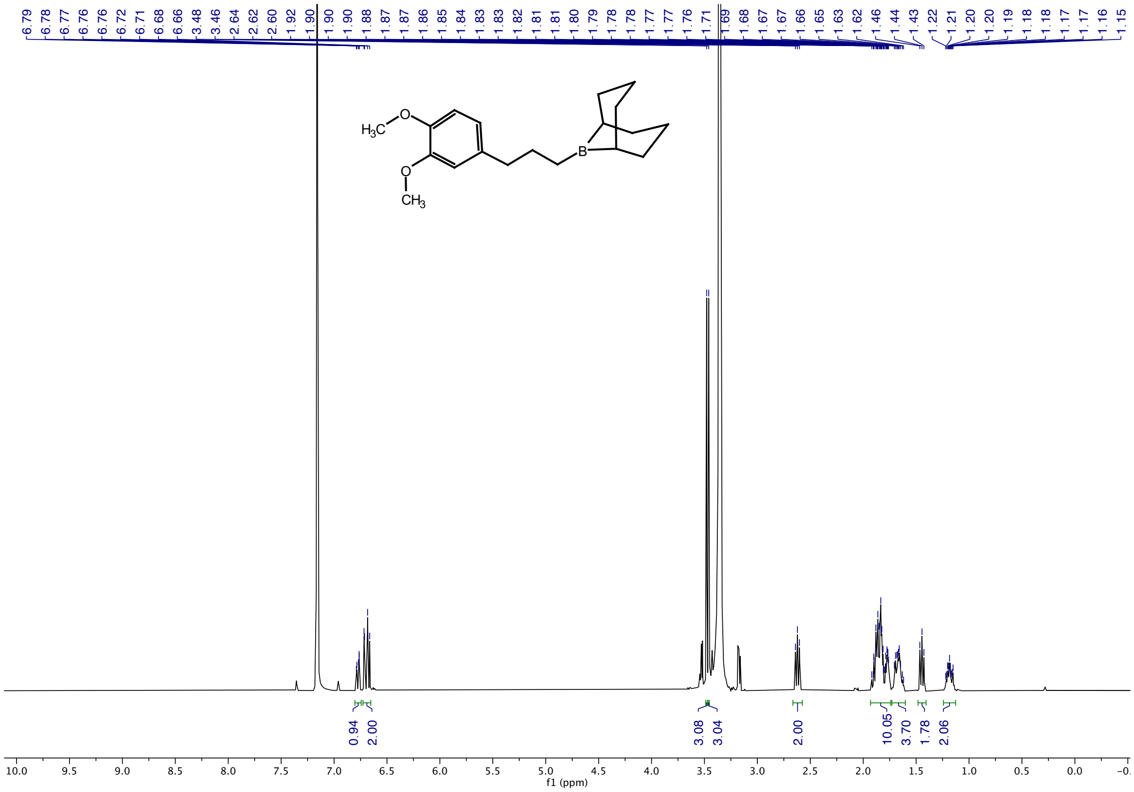

¹H NMR (400 MHz, C_6D_6) of **S3q** in 1,4-dioxane (0.4 M):

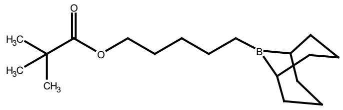

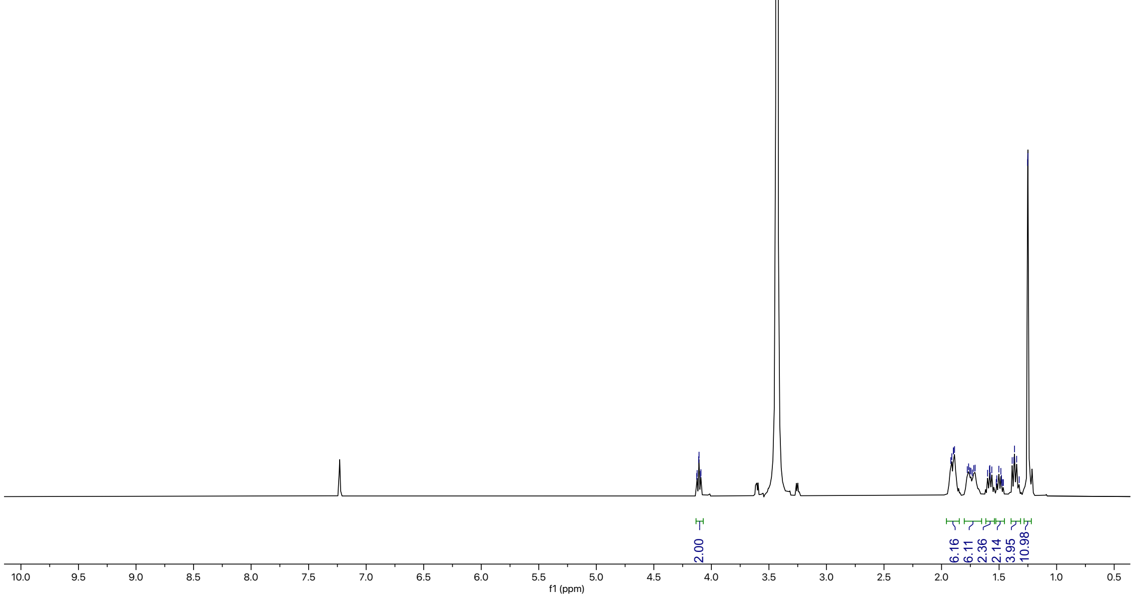

¹H NMR (400 MHz, C_6D_6) of **S3r** in 1,4-dioxane (0.4 M):

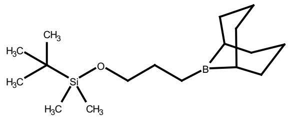

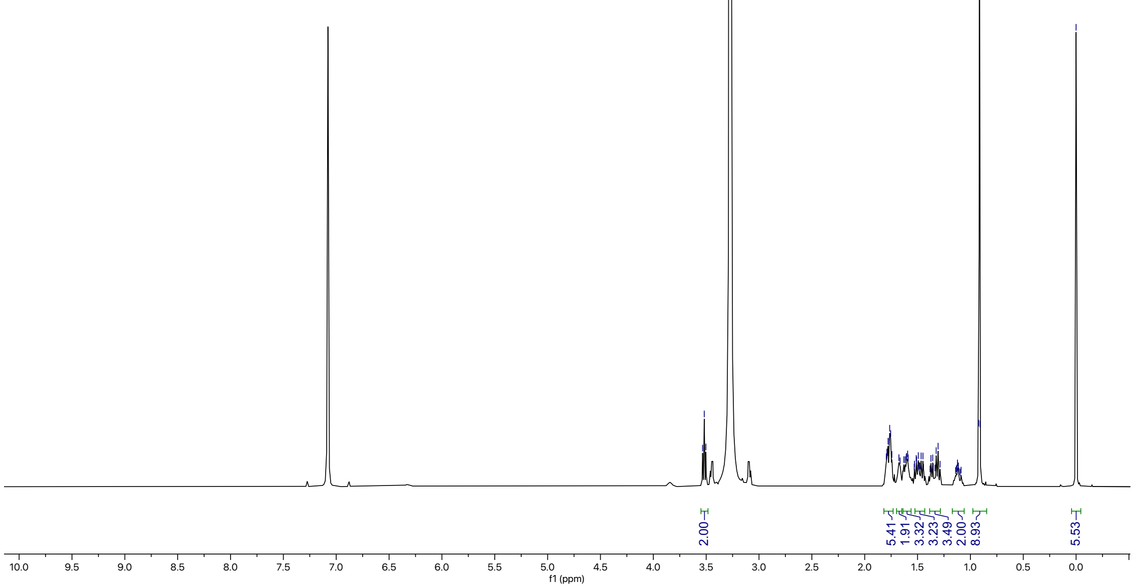

^{1}H NMR (400 MHz, C_6D_6) of **S3s** in 1,4-dioxane (0.4 M):

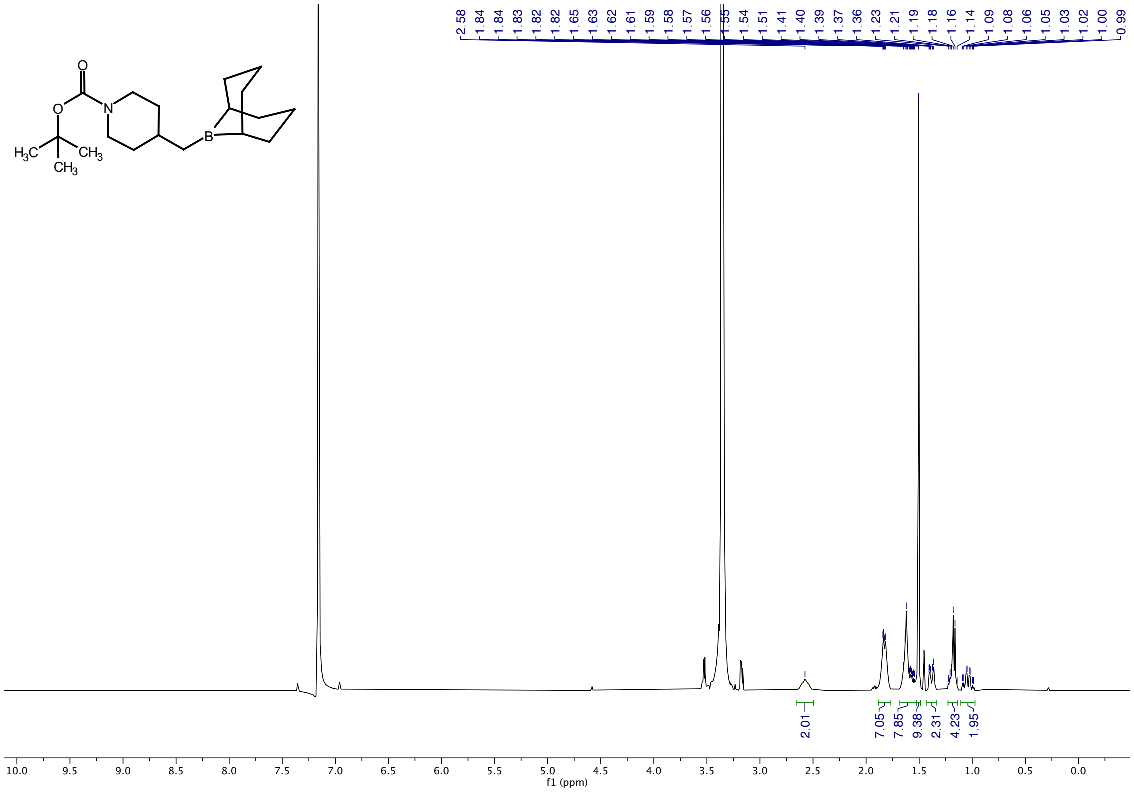

^{1}H NMR (400 MHz, C_6D_6) of **S3t** in 1,4-dioxane (0.4 M):

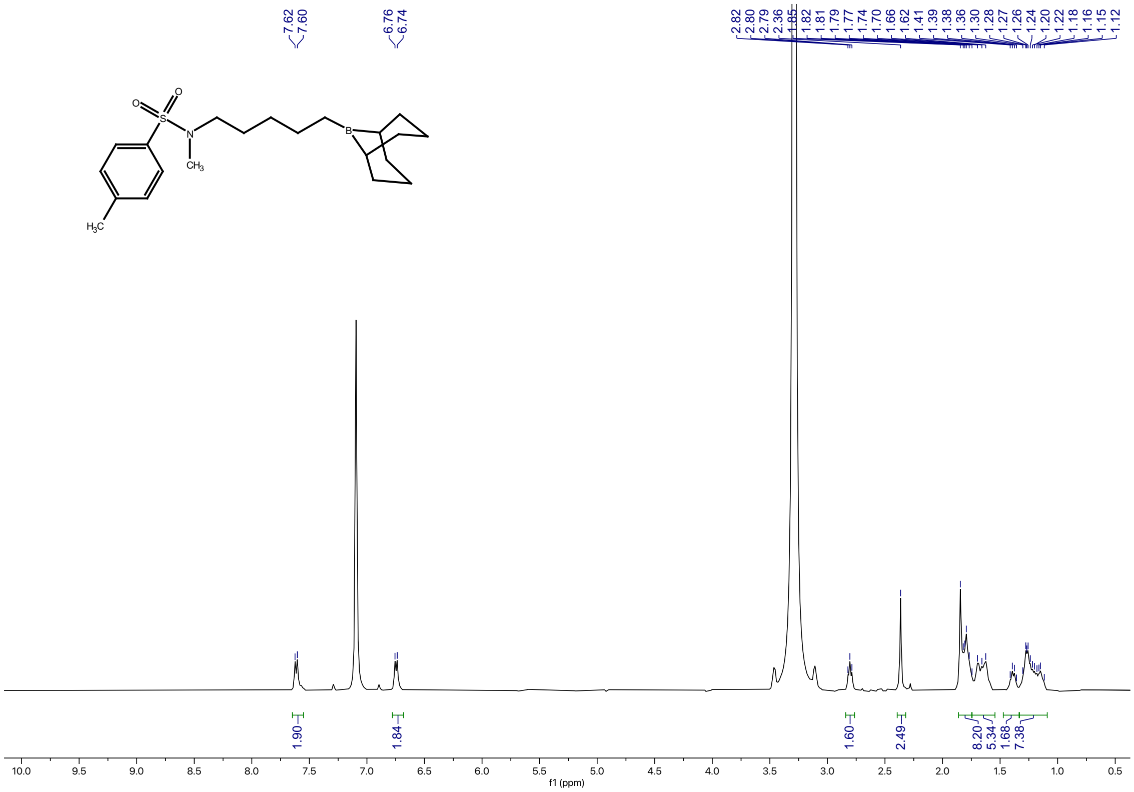

^{1}H NMR (400 MHz, C_6D_6) of **S3u** in 1,4-dioxane (0.4 M):

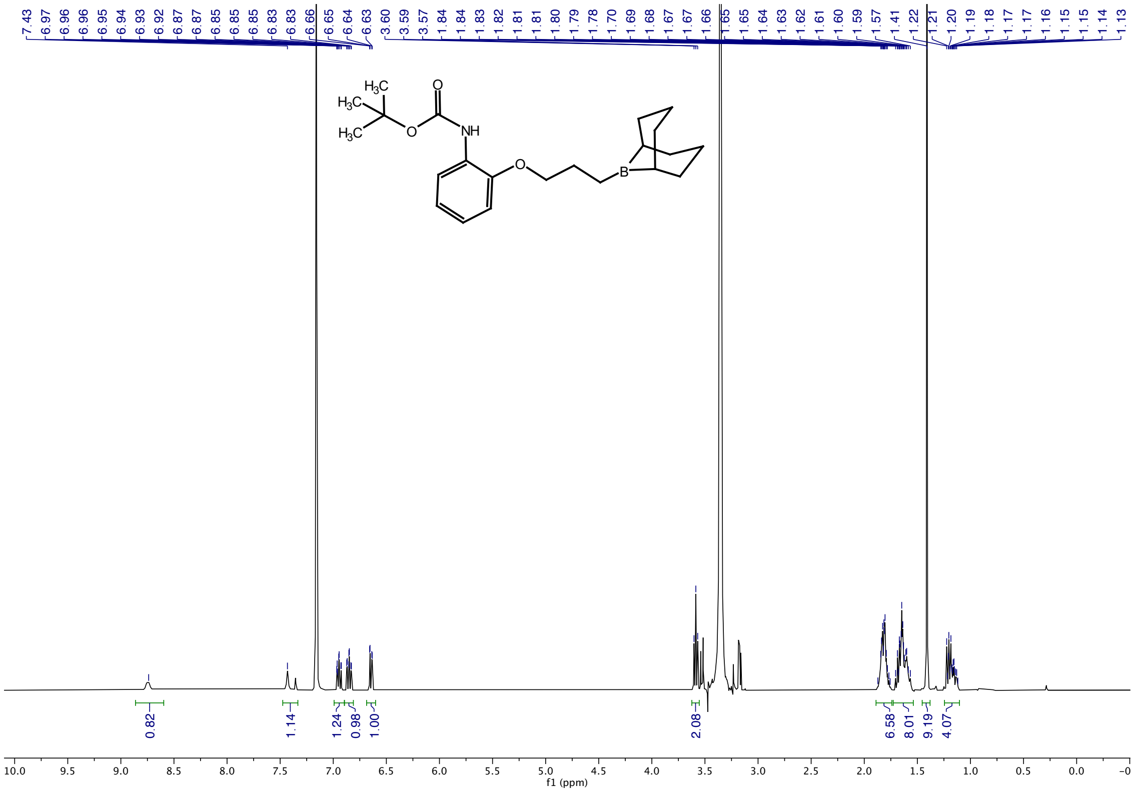

^{1}H NMR (400 MHz, C_6D_6) of **S3v** in 1,4-dioxane (0.4 M):

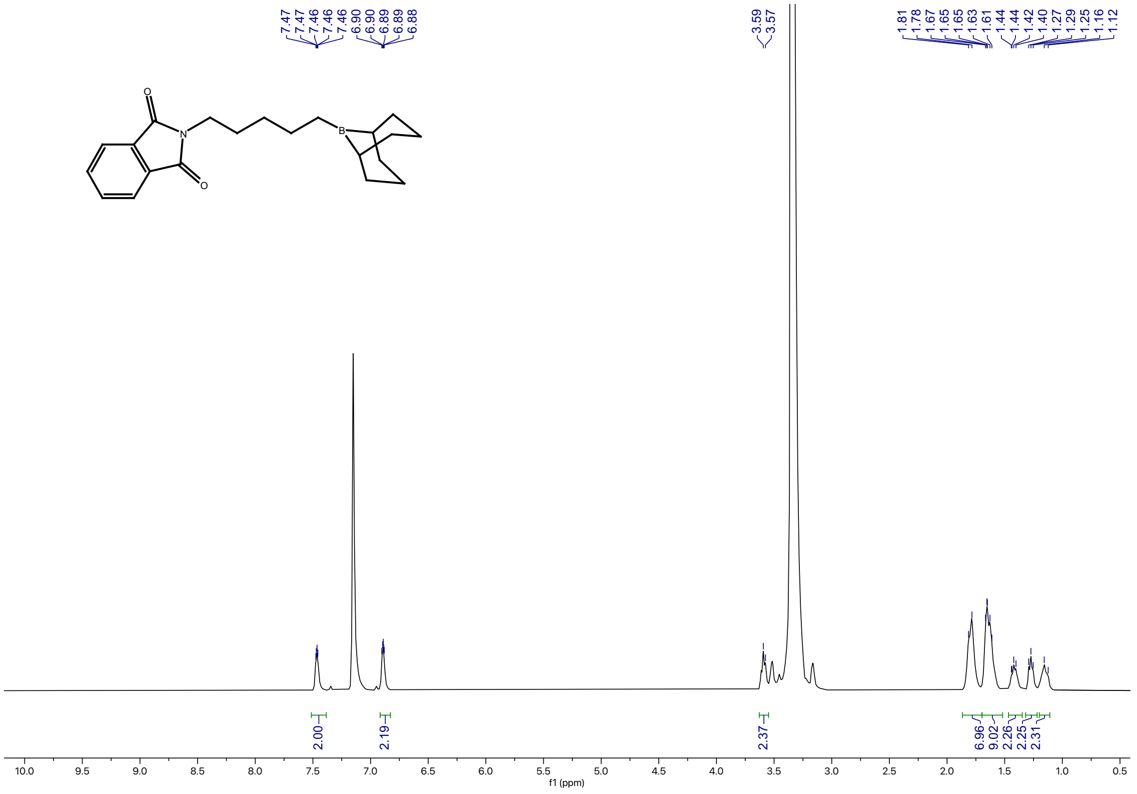

^{1}H NMR (400 MHz, C_6D_6) of **S3x**:

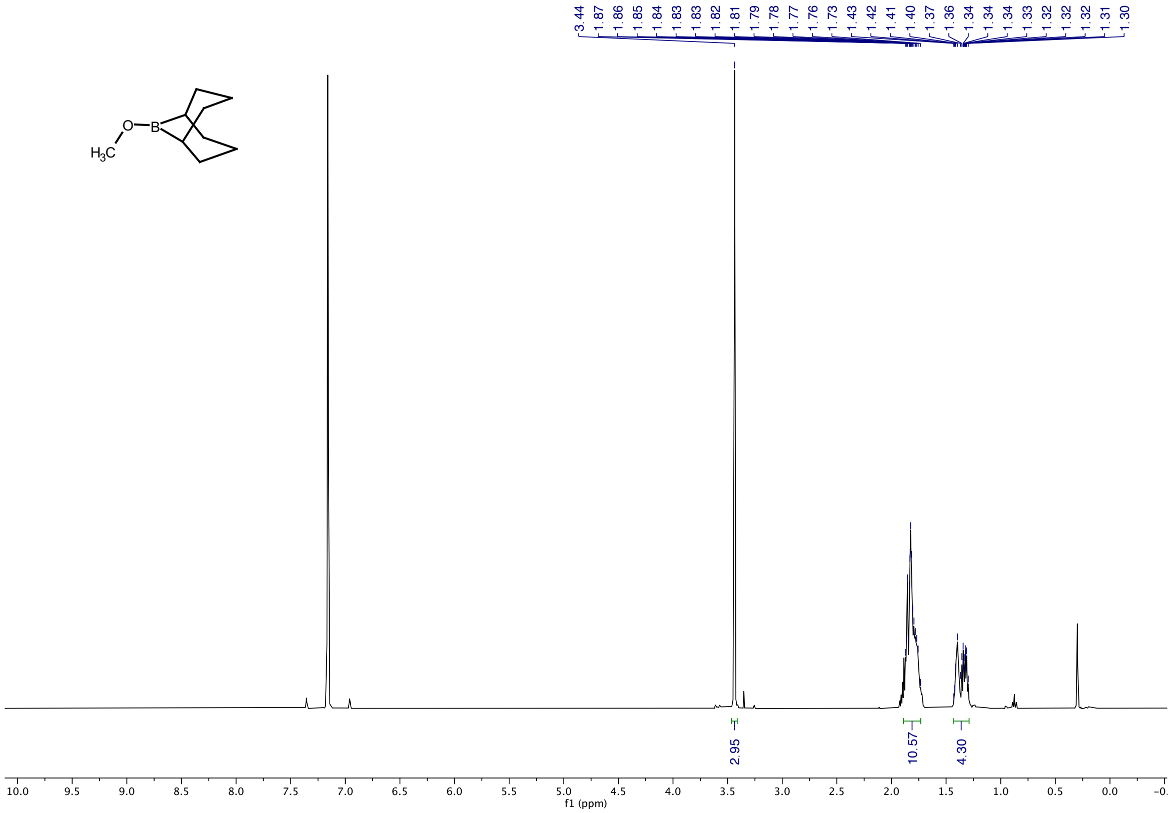

^{1}H NMR (400 MHz, $CDCl_3$) of **4a**:

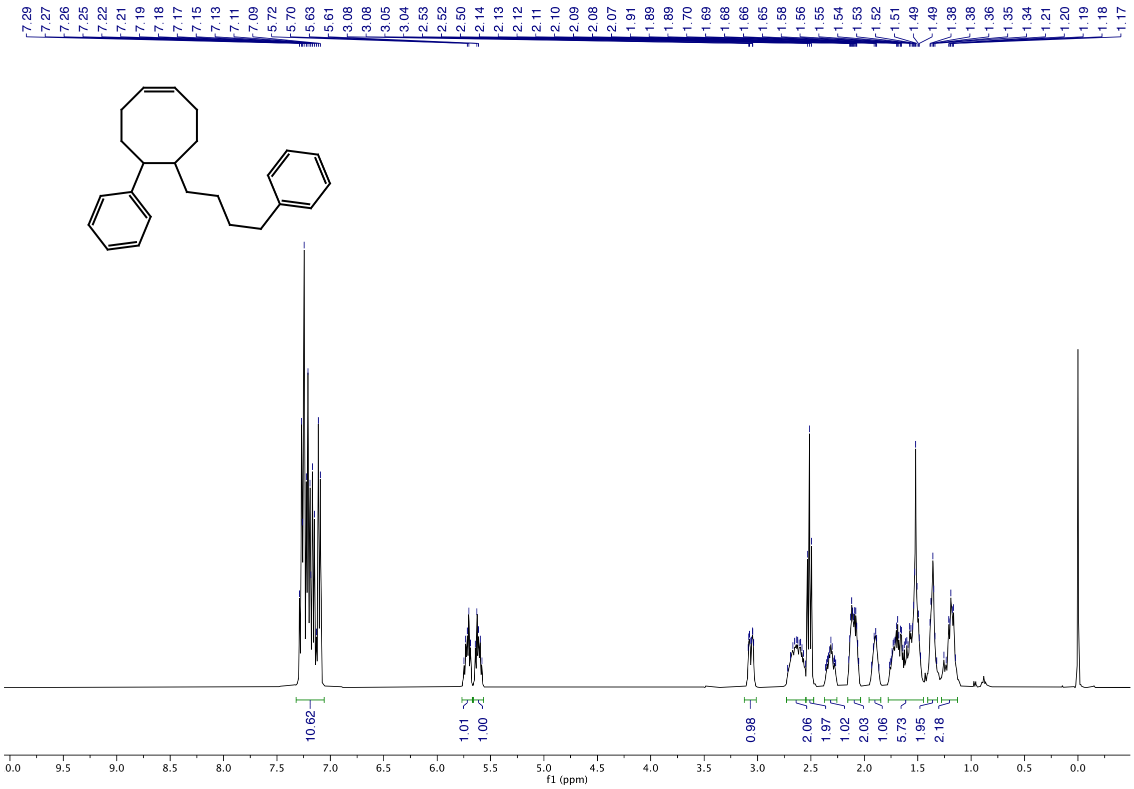

^{13}C NMR (101 MHz, $CDCl_3$) of **4a**:

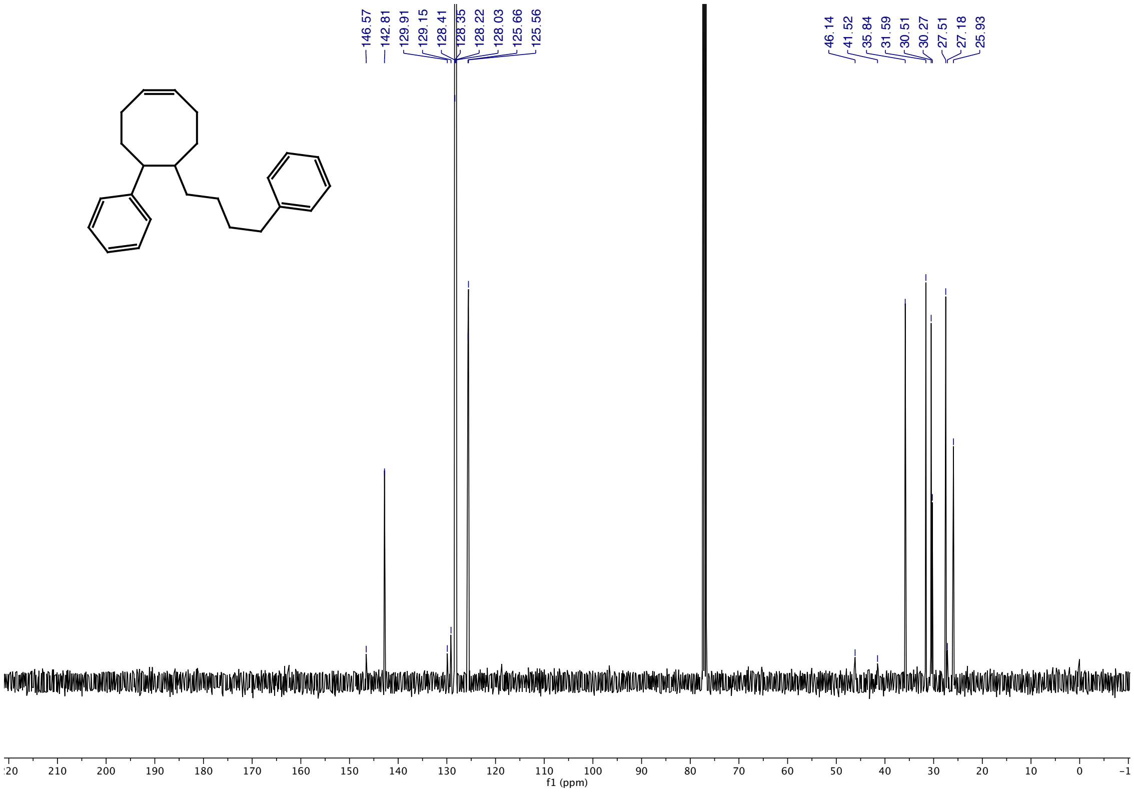

^{1}H NMR (400 MHz, $CDCl_3$) of **4b**:

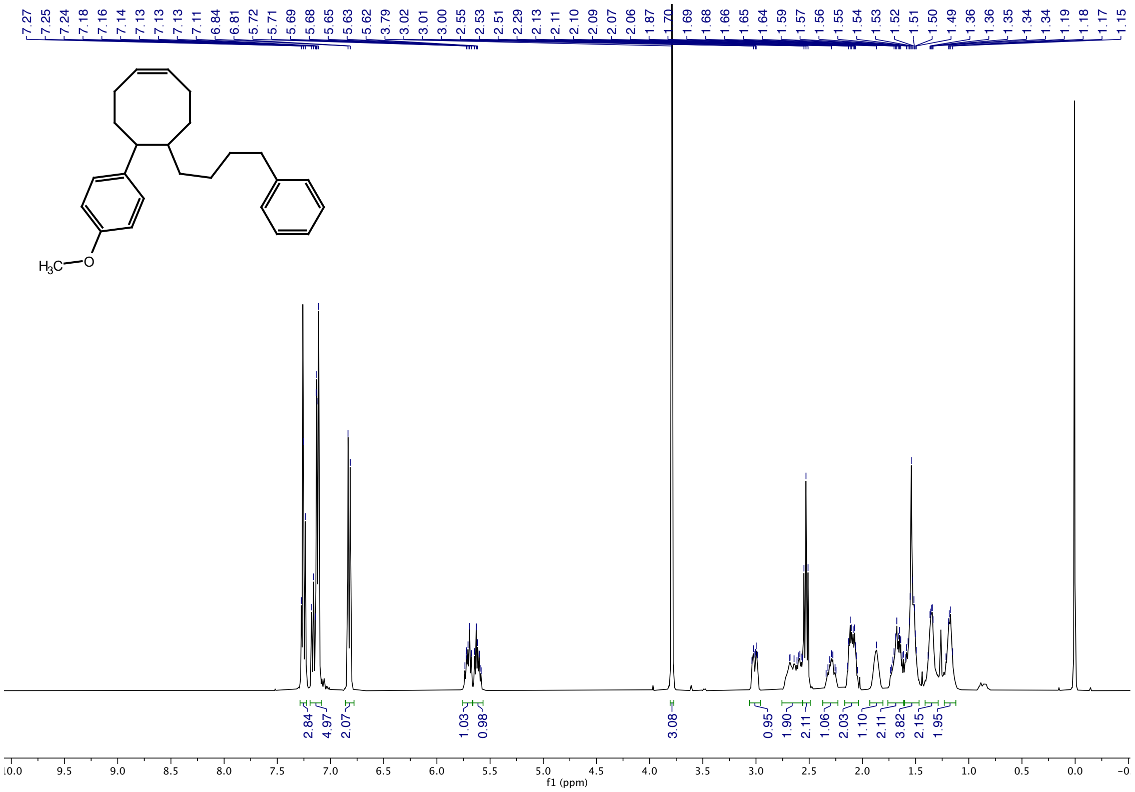

^{13}C NMR (101 MHz, $CDCl_3$) of **4b**:

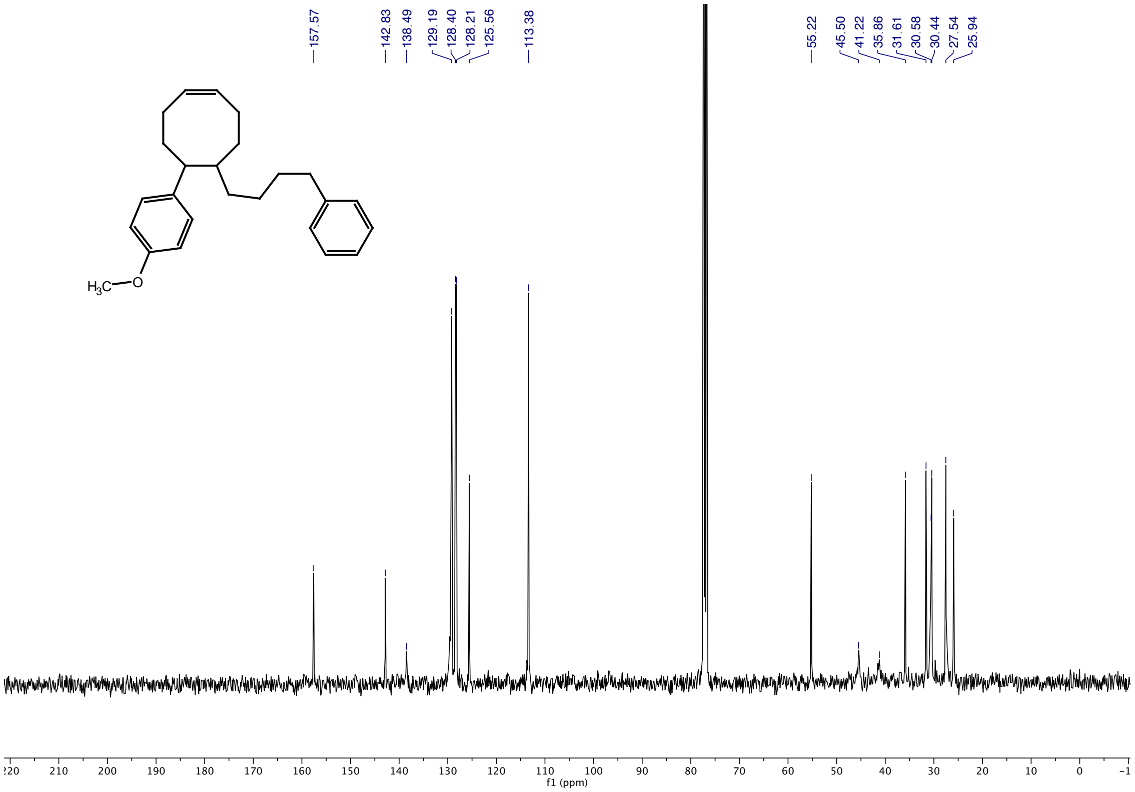

^{1}H NMR (400 MHz, $CDCl_3$) of **4c**:

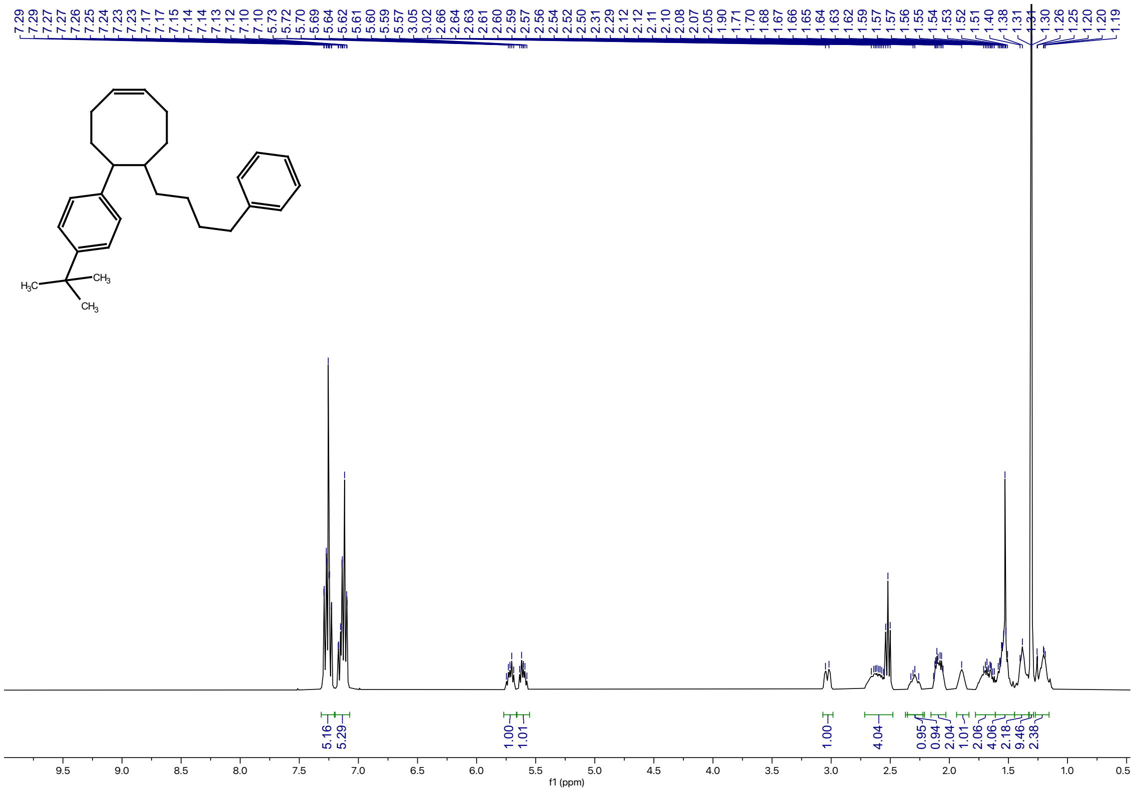

^{13}C NMR (101 MHz, $CDCl_3$) of **4c**:

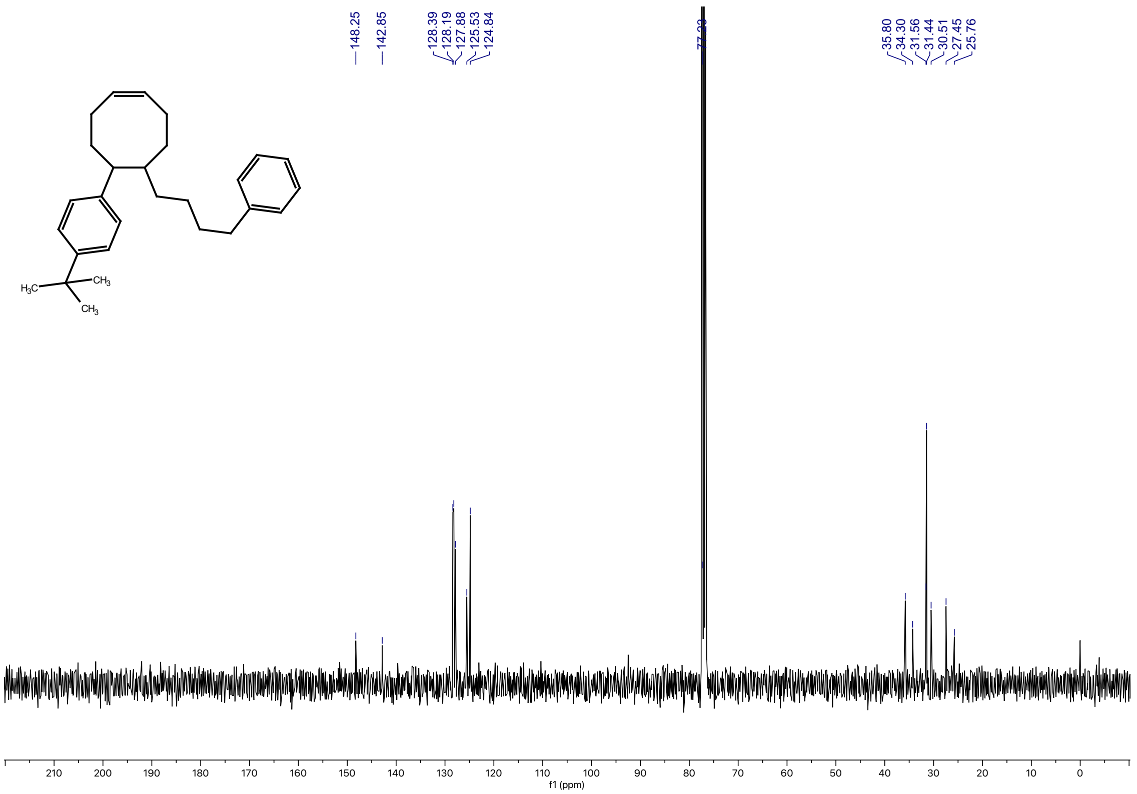

^{1}H NMR (400 MHz, $CDCl_3$) of **4d**:

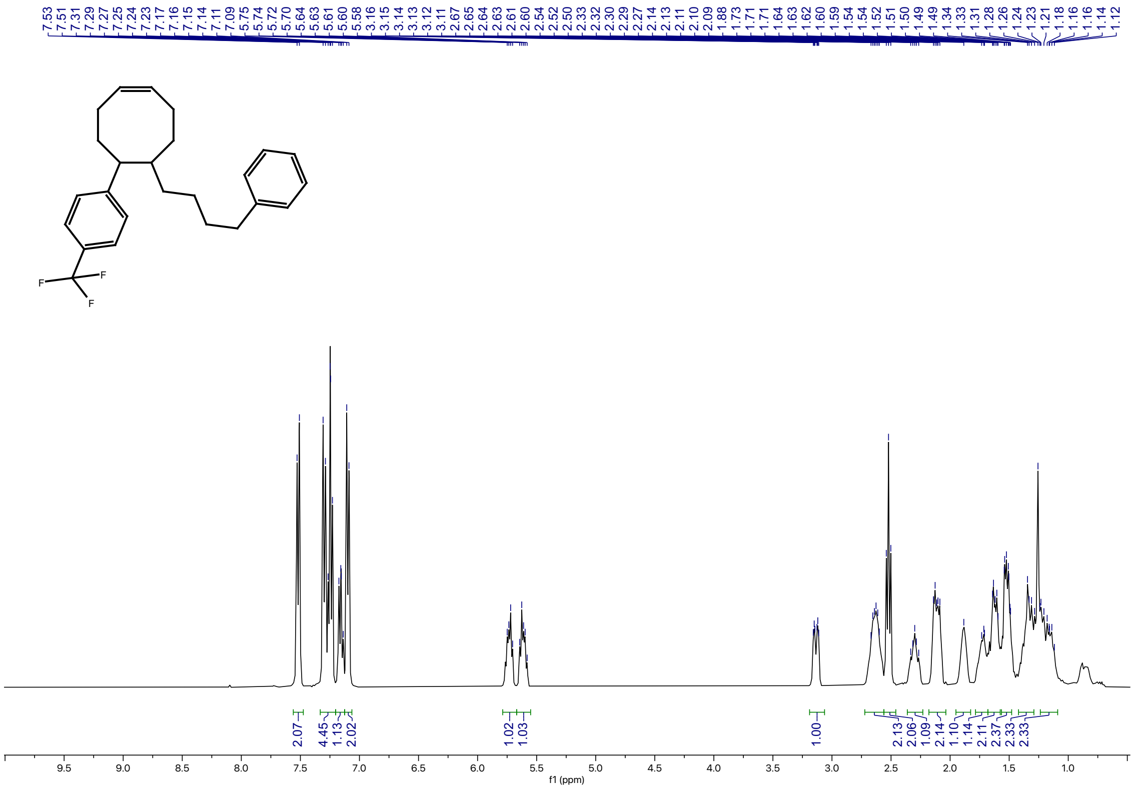

^{13}C NMR (101 MHz, $CDCl_3$) of **4d**:

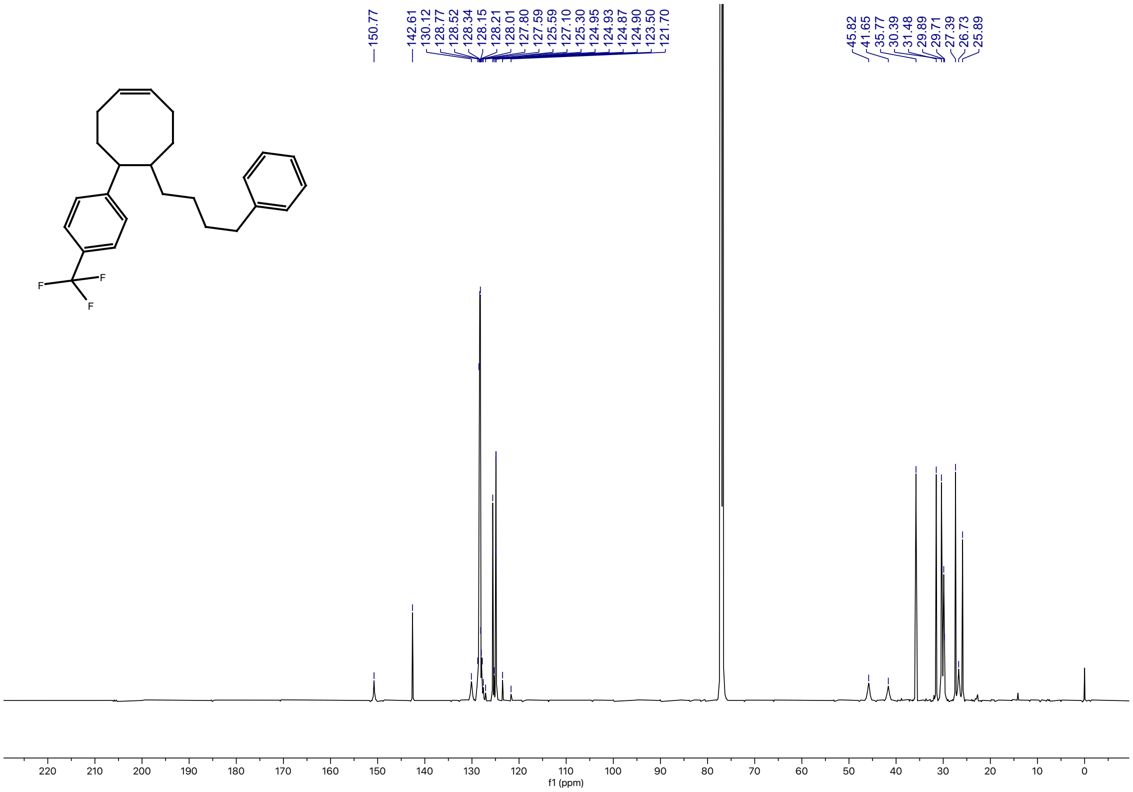

^{19}F NMR (376 MHz, $CDCl_3$) of **4d**:

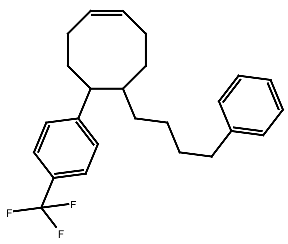

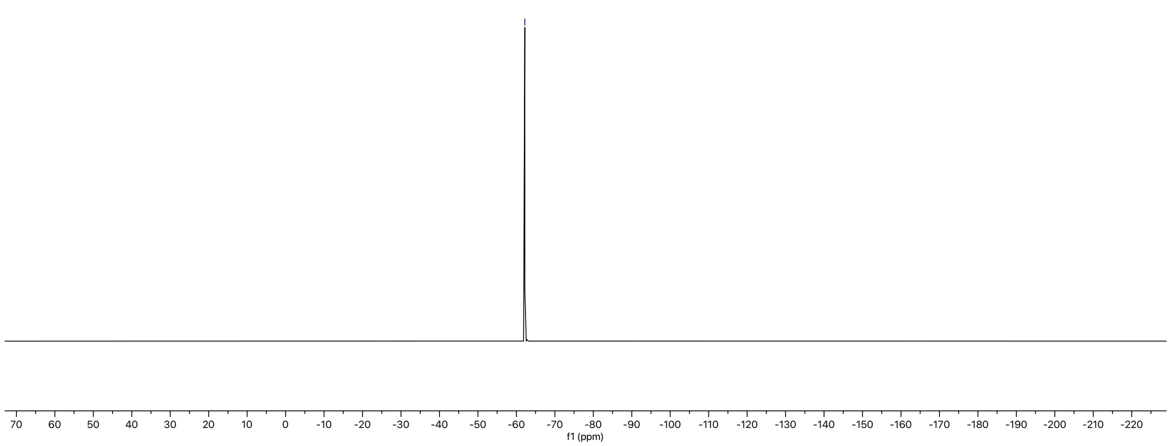

^{1}H NMR (400 MHz, $CDCl_3$) of **4e**:

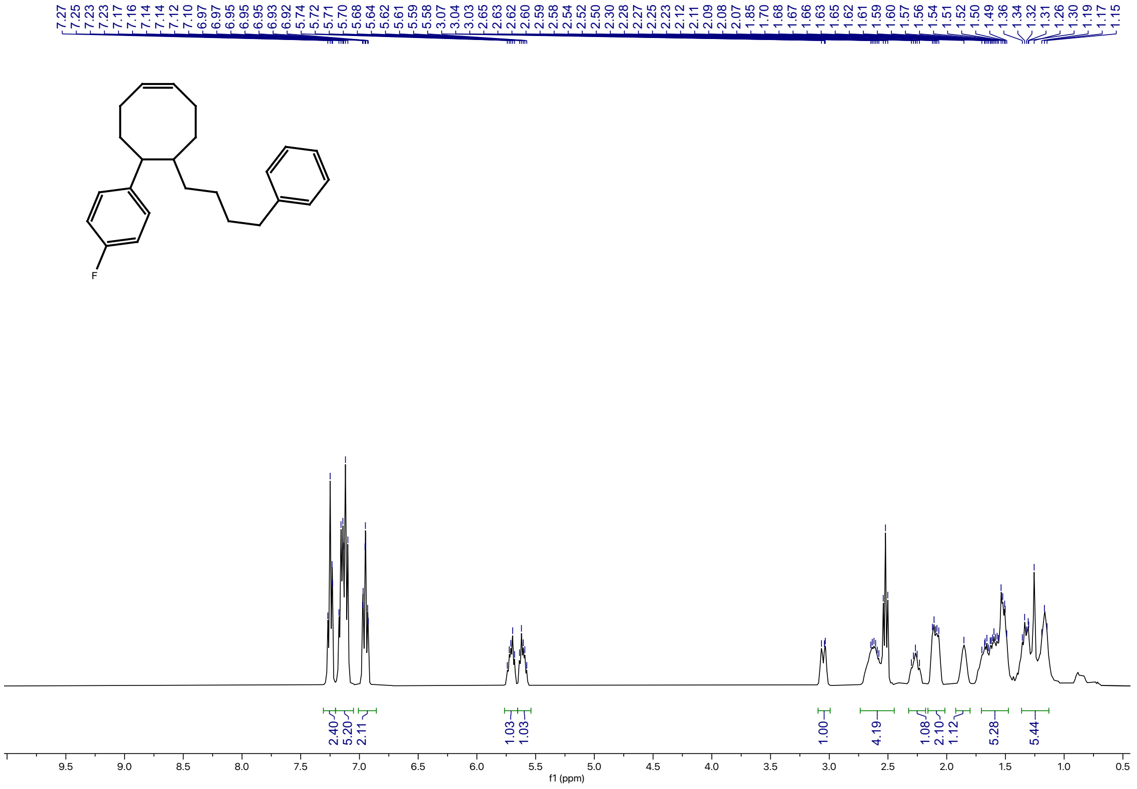

^{13}C NMR (101 MHz, $CDCl_3$) of **4e**:

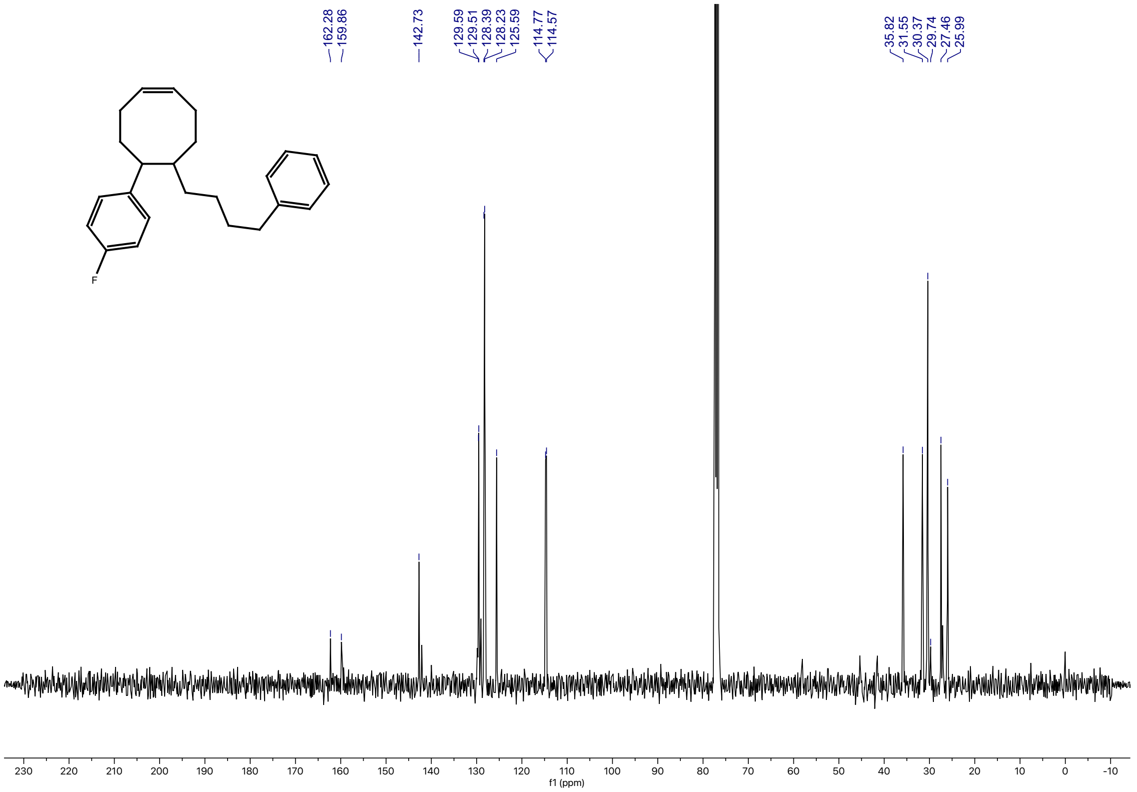

^{19}F NMR (376 MHz, $CDCl_3$) of **4e**:

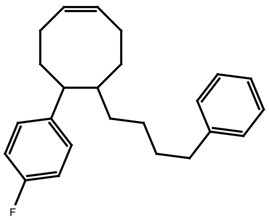

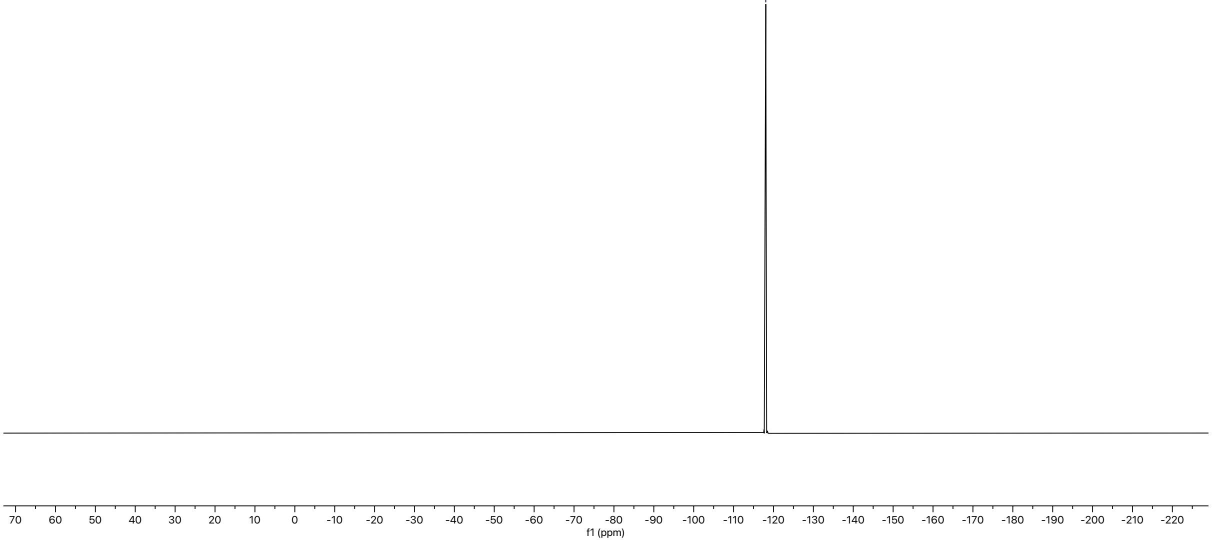

^{1}H NMR (400 MHz, $CDCl_3$) of **4f**:

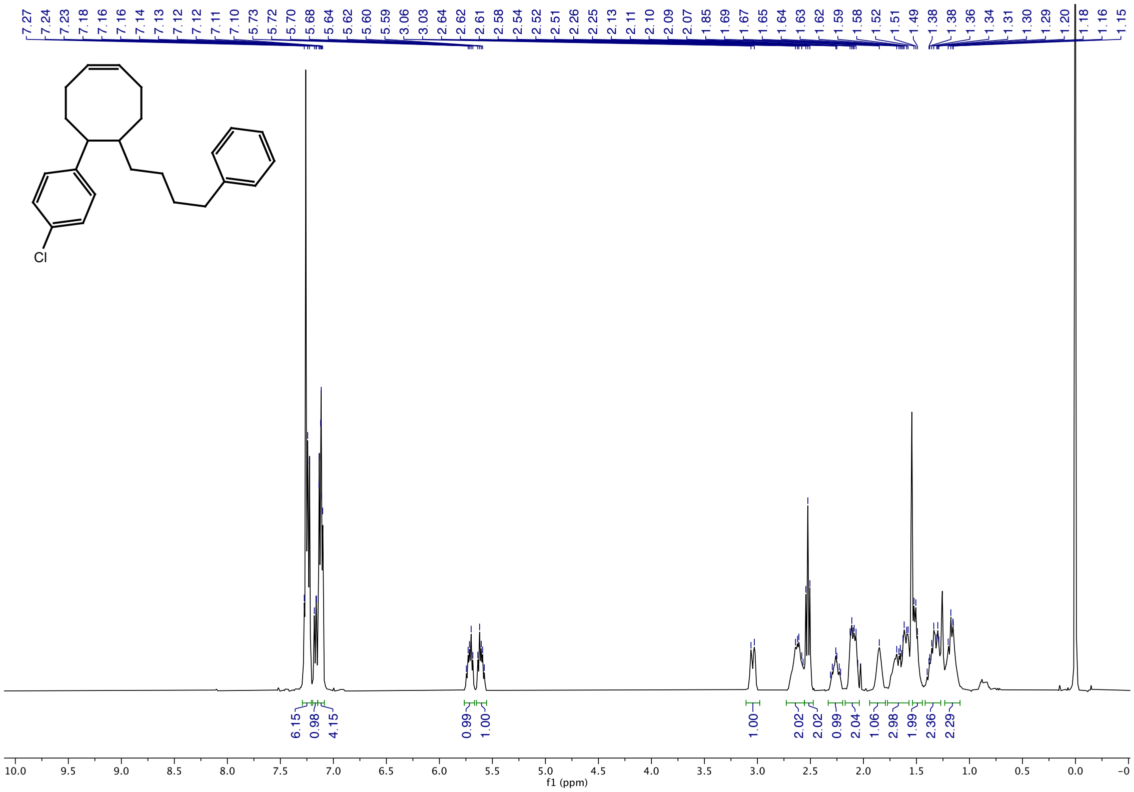

^{13}C NMR (101 MHz, $CDCl_3$) of **4f**:

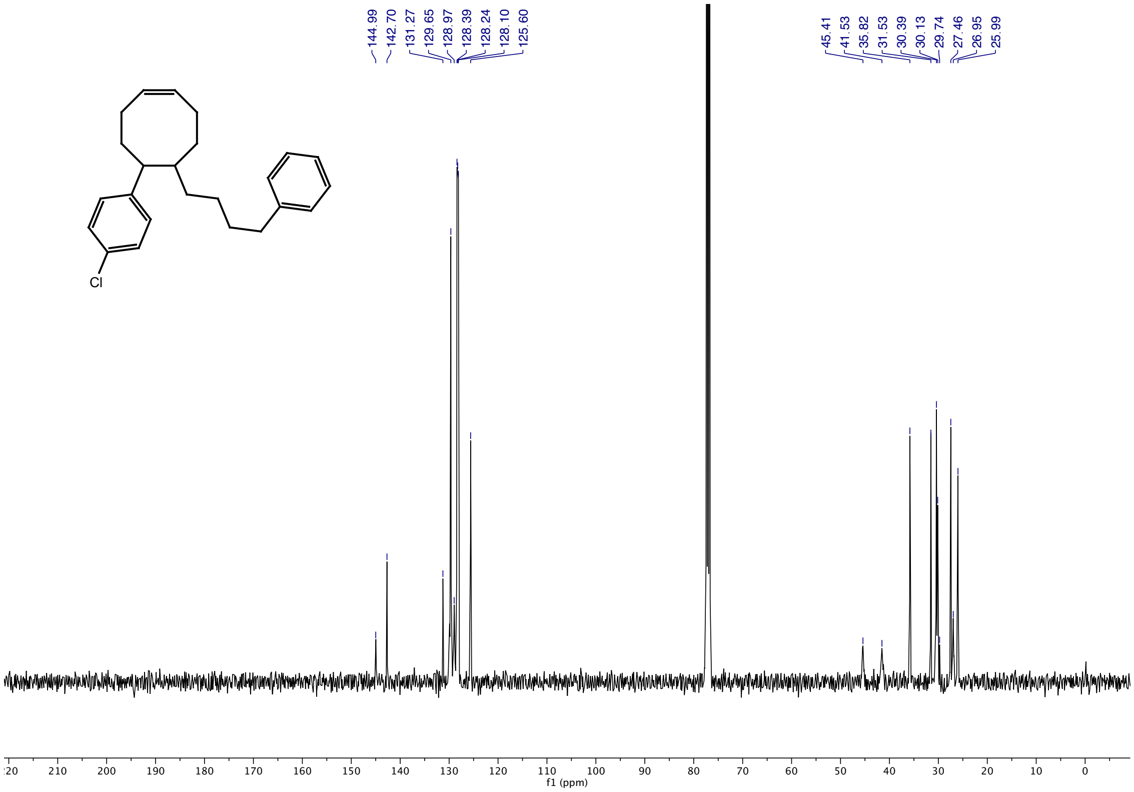

¹H NMR (400 MHz, $CDCl_3$) of **4g**:

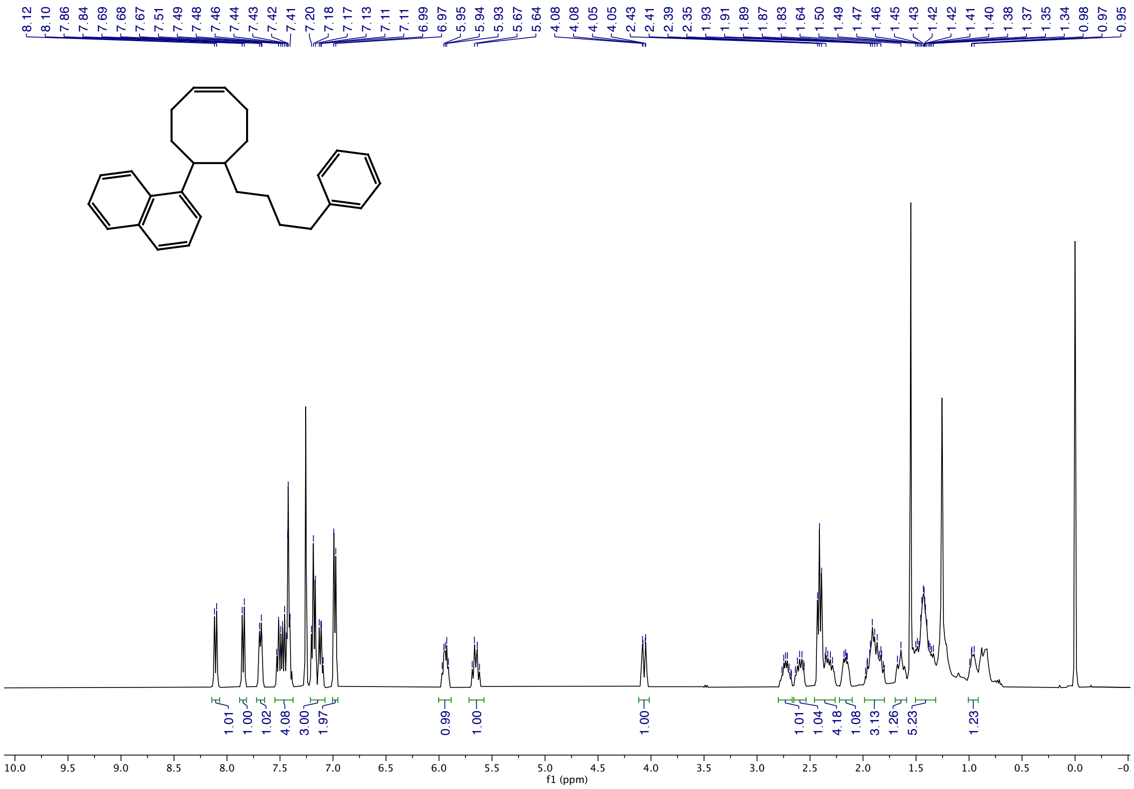

¹³C NMR (101 MHz, $CDCl_3$) of **4g**:

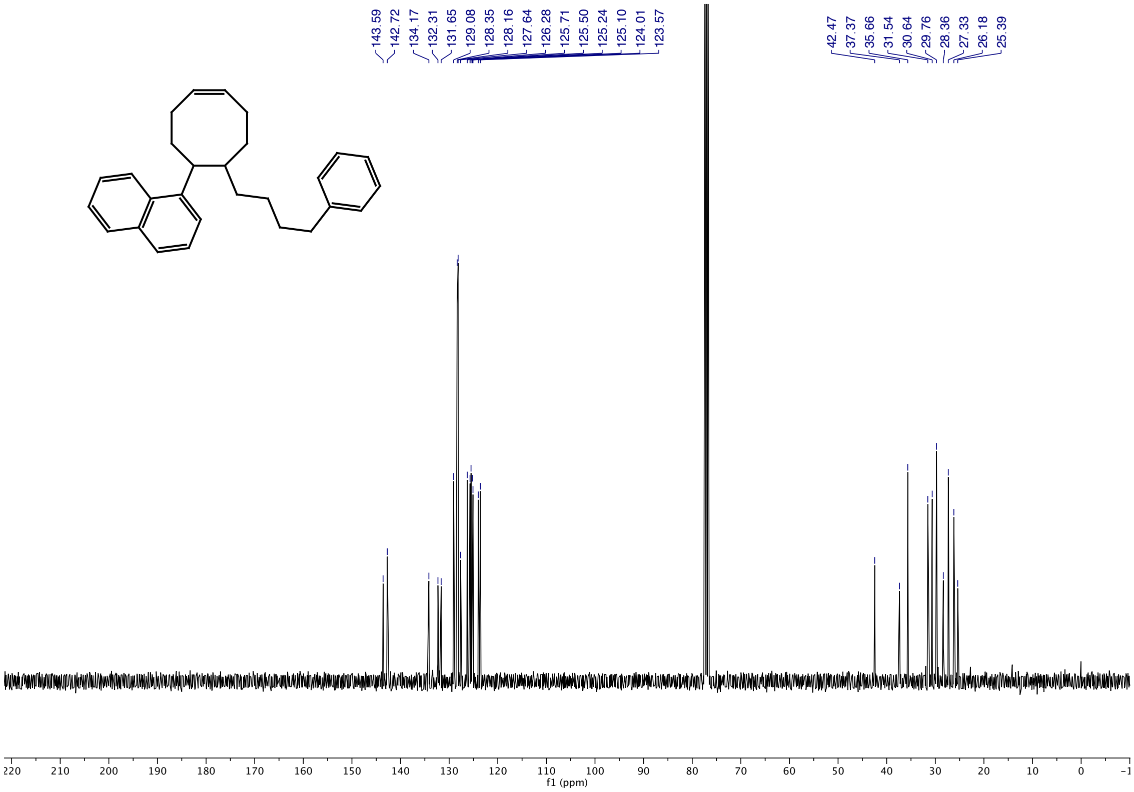

^{1}H NMR (400 MHz, $CDCl_3$) of **4h**:

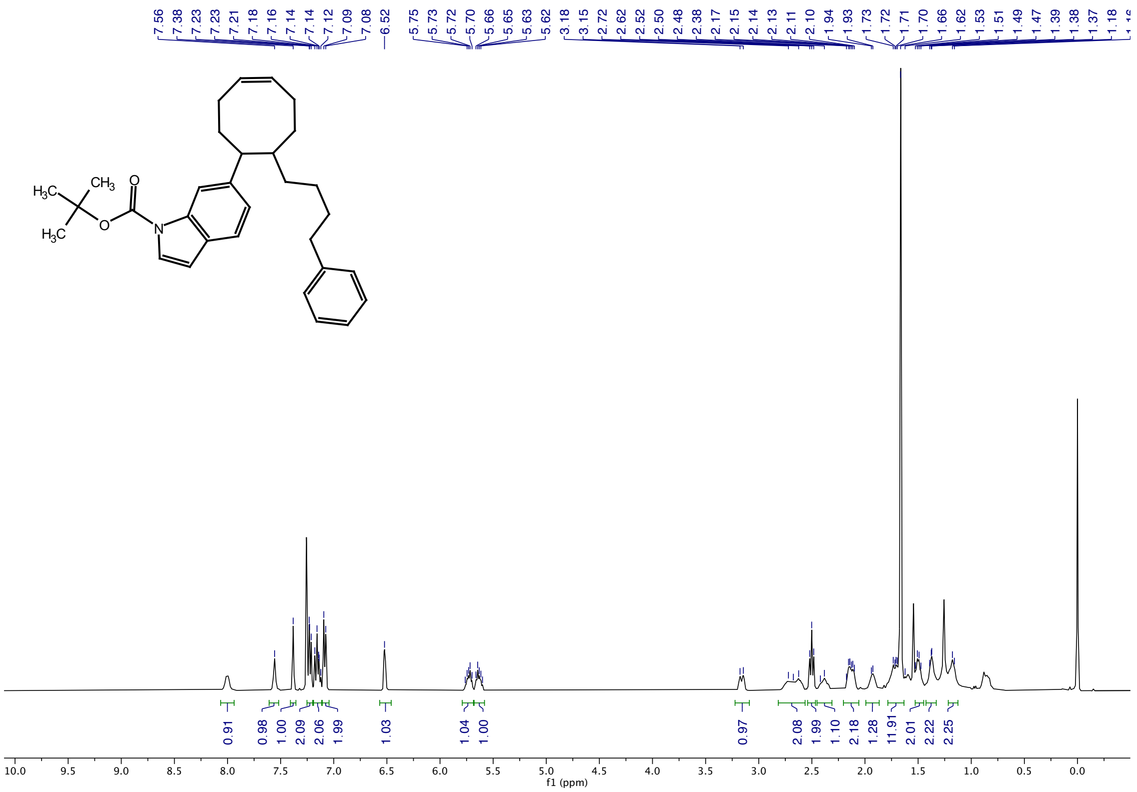

^{13}C NMR (101 MHz, $CDCl_3$) of **4h**:

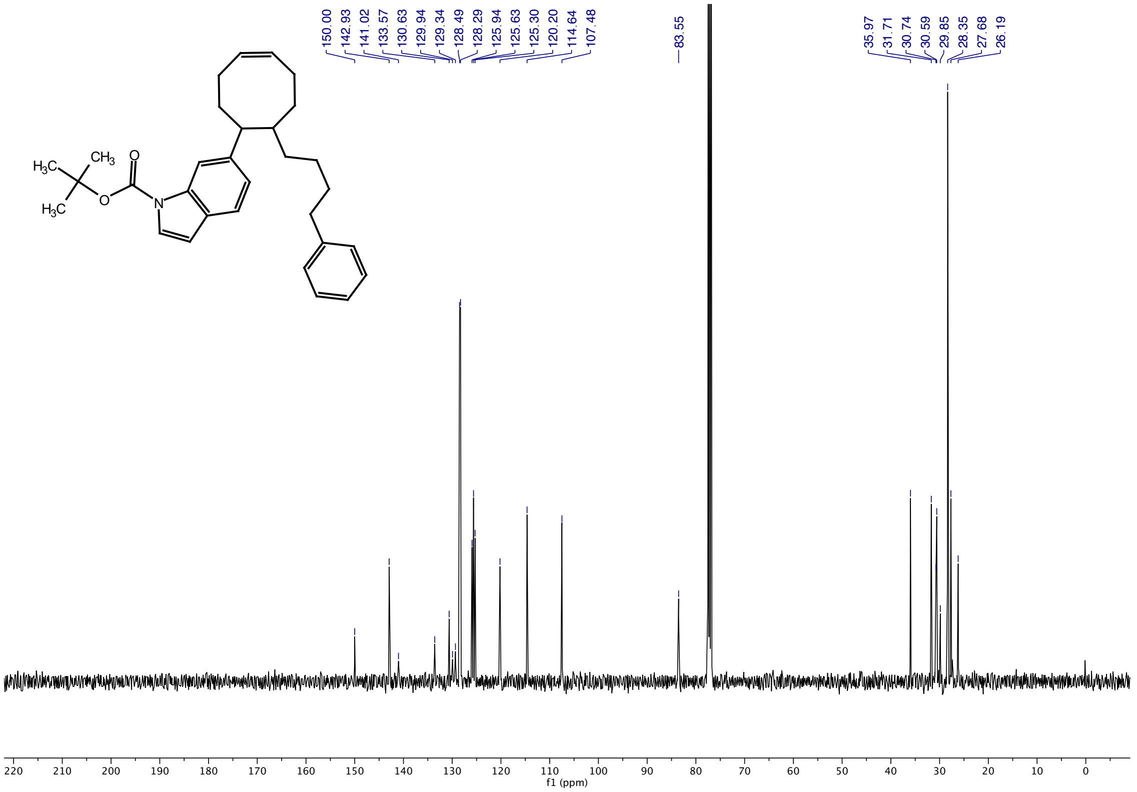

^{1}H NMR (400 MHz, $CDCl_3$) of **4i**:

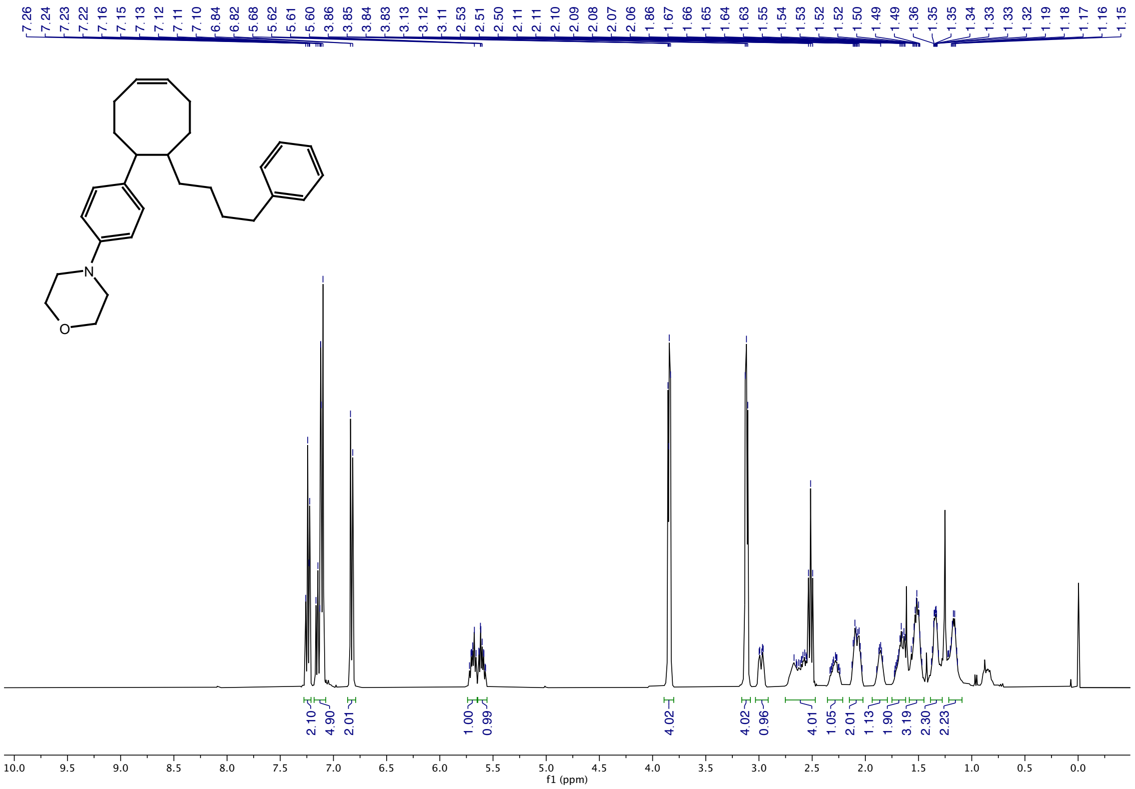

^{13}C NMR (101 MHz, $CDCl_3$) of **4i**:

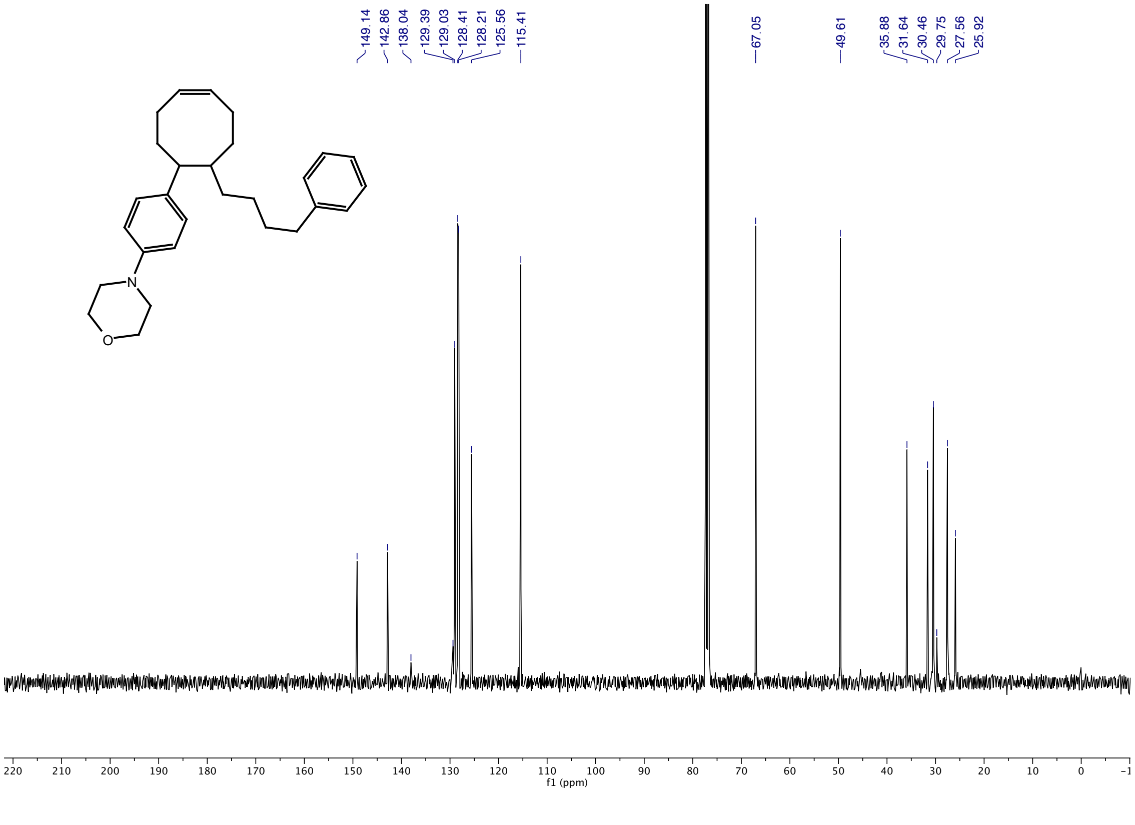

^{1}H NMR (400 MHz, $CDCl_3$) of **4j**:

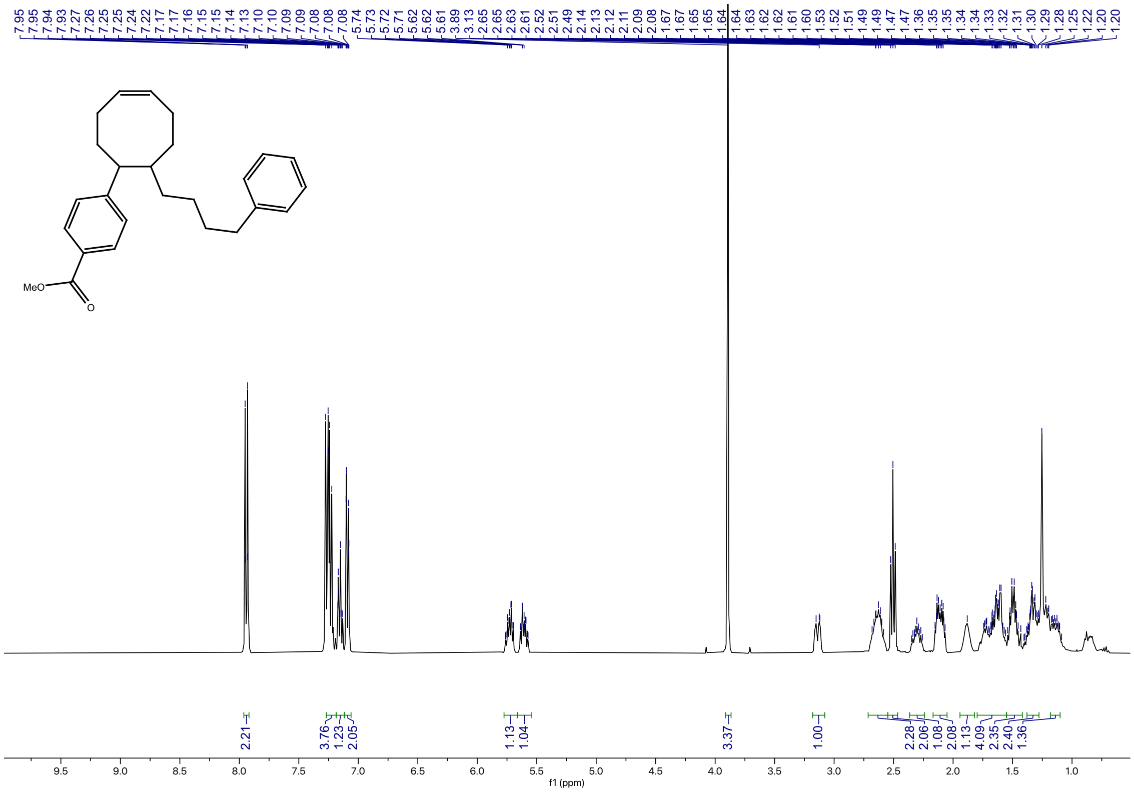

^{13}C NMR (100 MHz, $CDCl_3$) of **4j**:

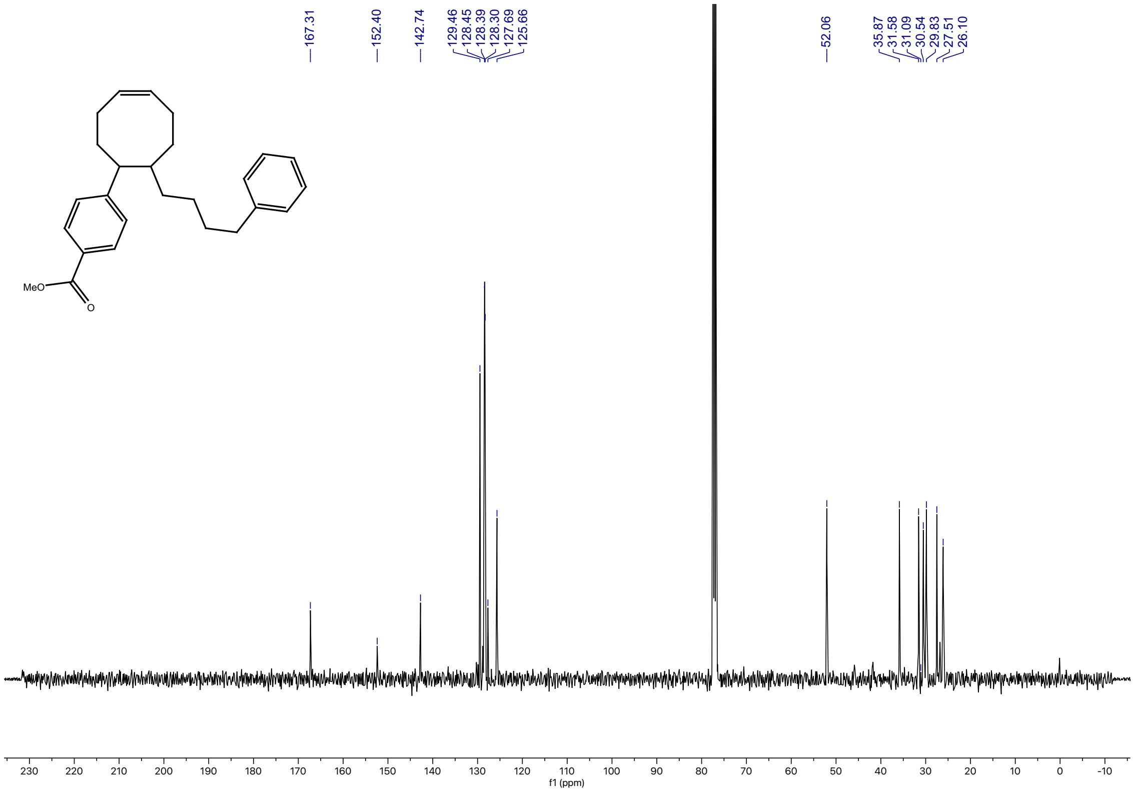

^{1}H NMR (600 MHz, $CDCl_3$) of **4k**:

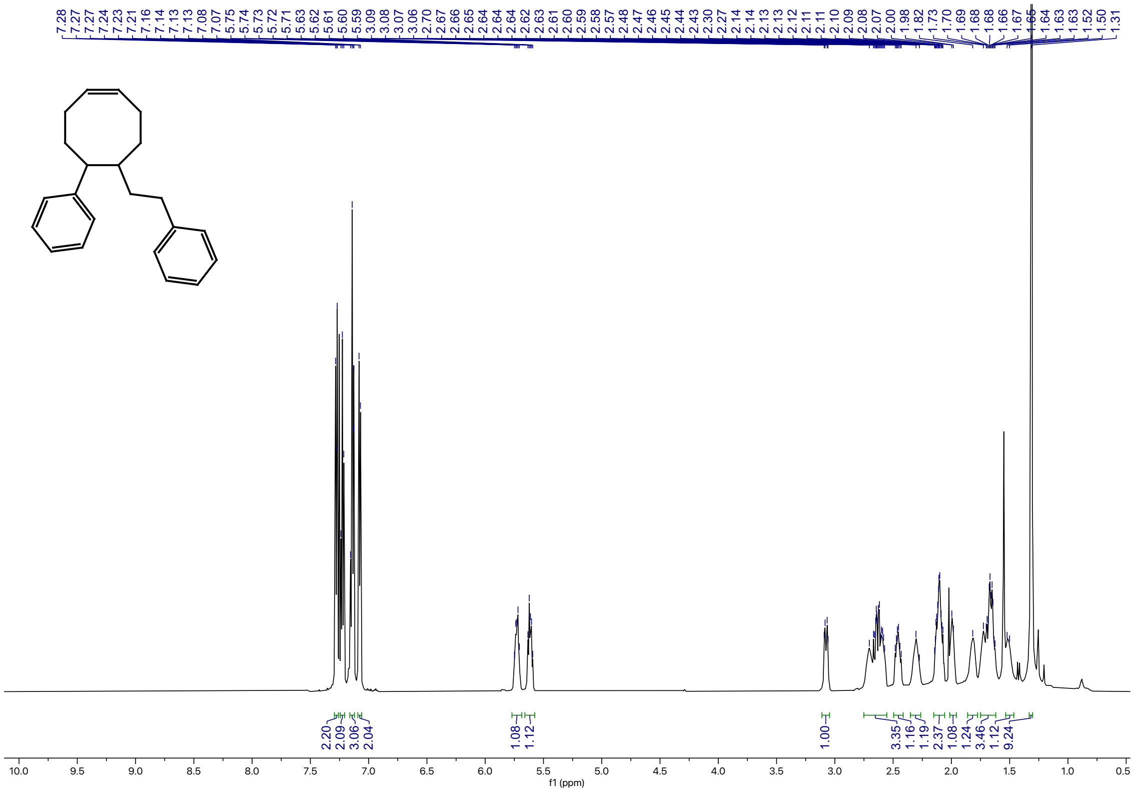

^{13}C NMR (151 MHz, $CDCl_3$) of **4k**:

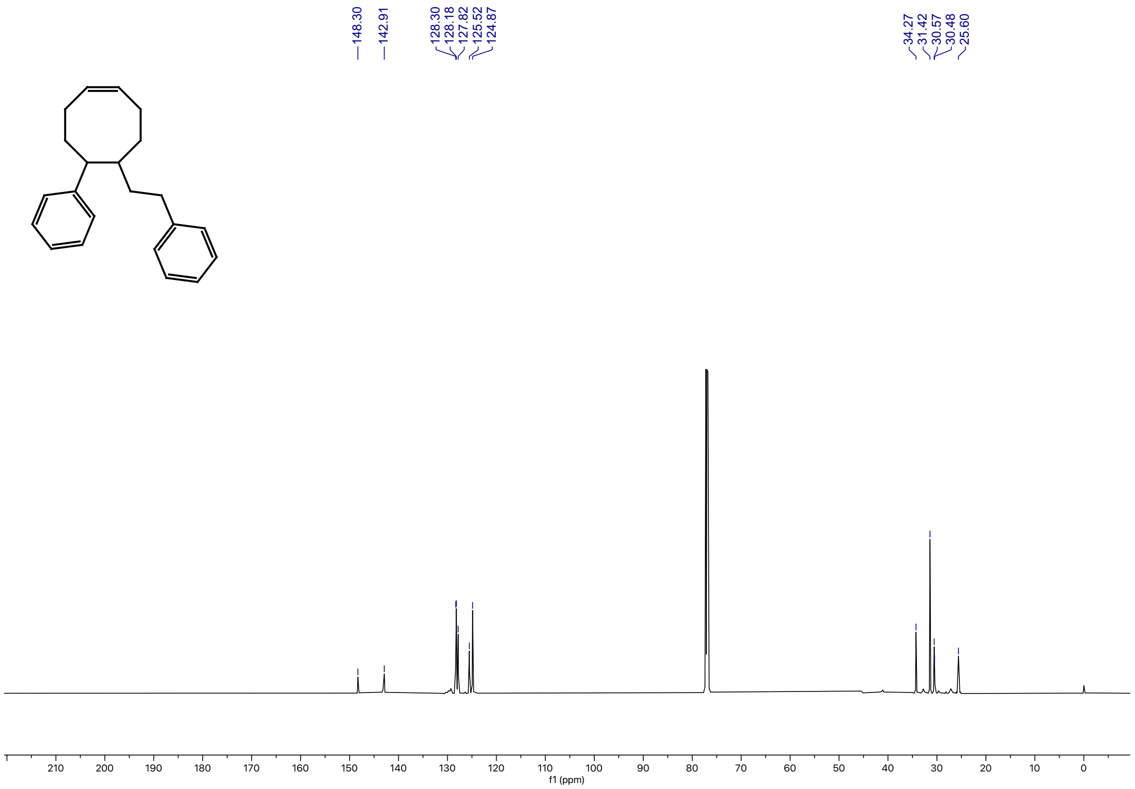

¹H NMR (400 MHz, $CDCl_3$) of **4l**:

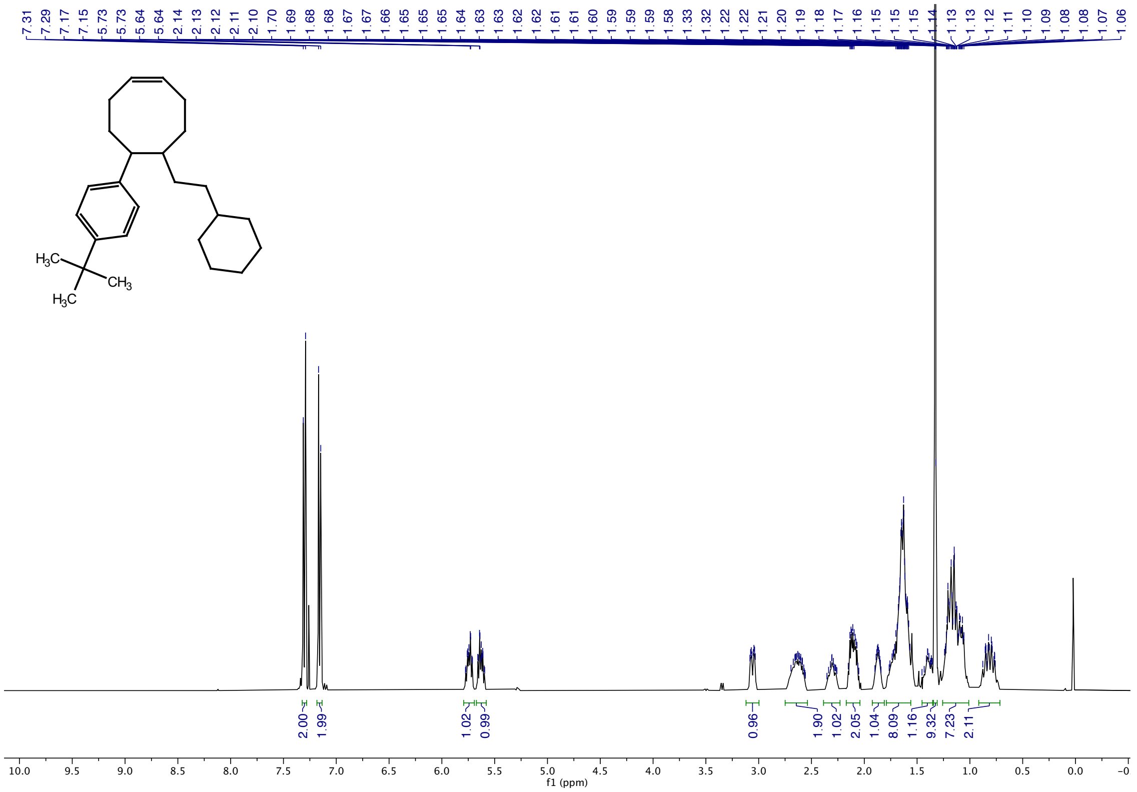

¹³C NMR (101 MHz, $CDCl_3$) of **4l**:

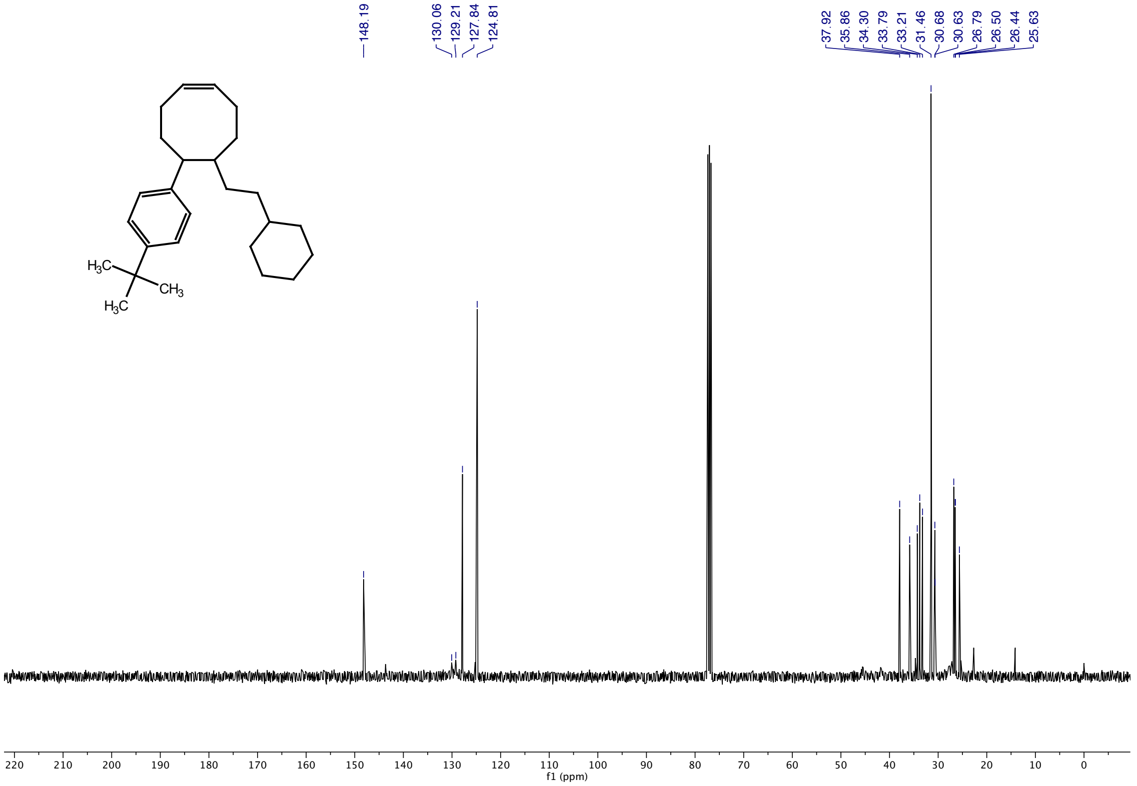

^{1}H NMR (400 MHz, $CDCl_3$) of **4m**:

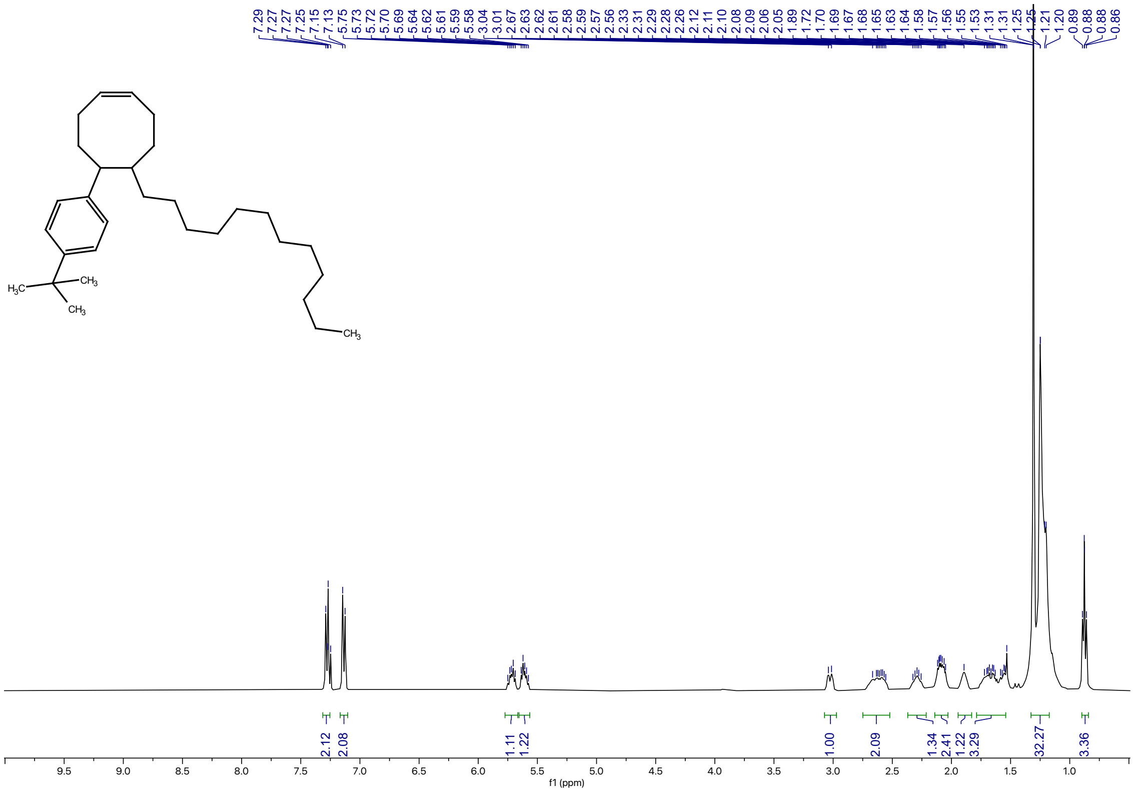

^{13}C NMR (101 MHz, $CDCl_3$) of **4m**:

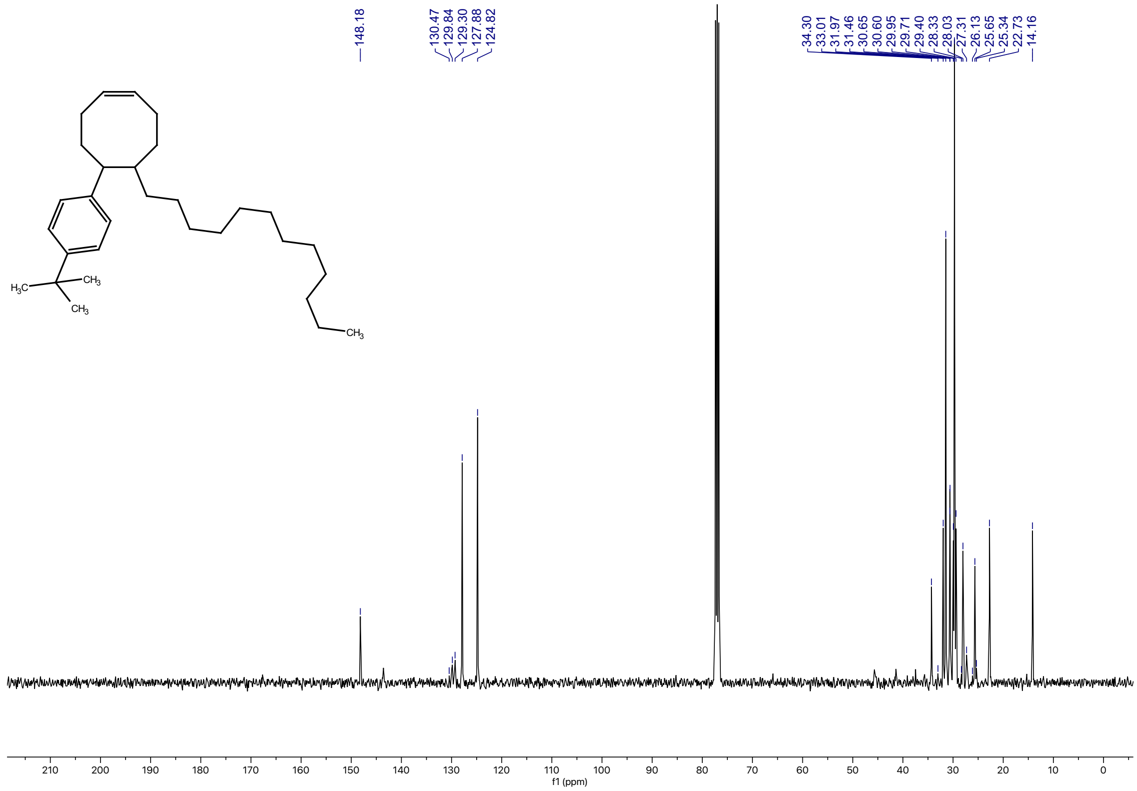

¹H NMR (400 MHz, $CDCl_3$) of **4n**:

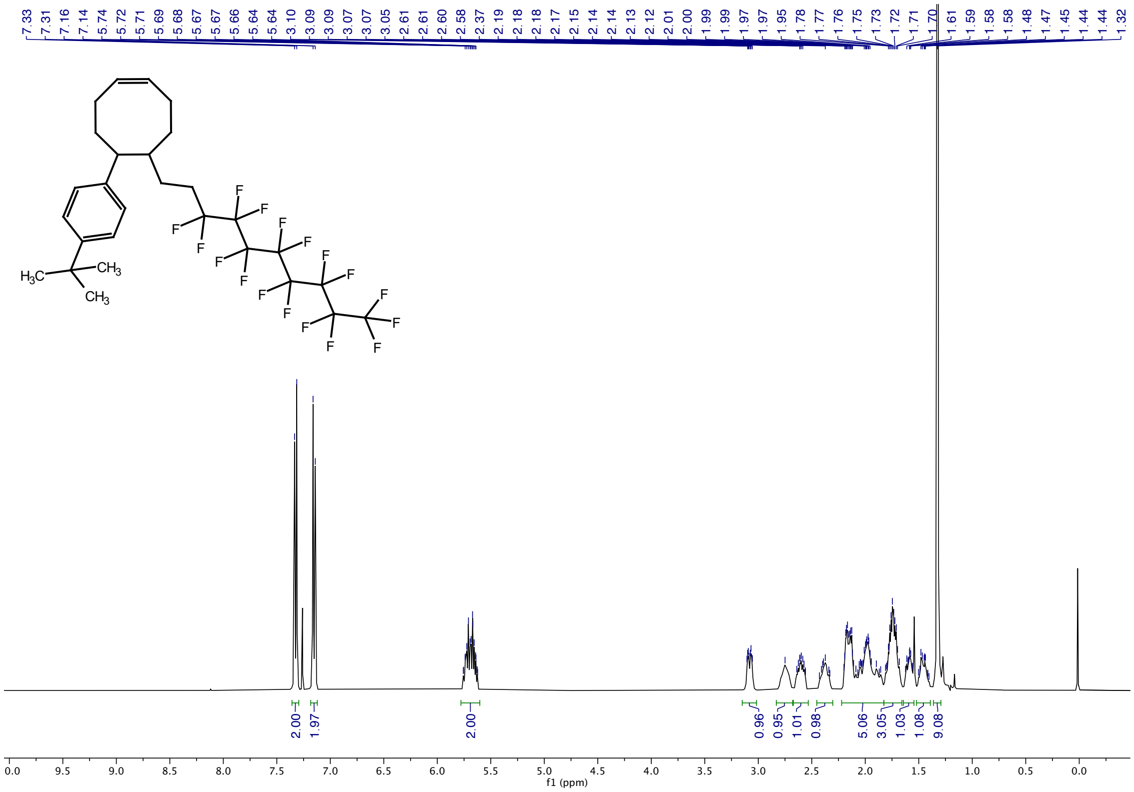

¹³C NMR (101 MHz, $CDCl_3$) of **4n**:

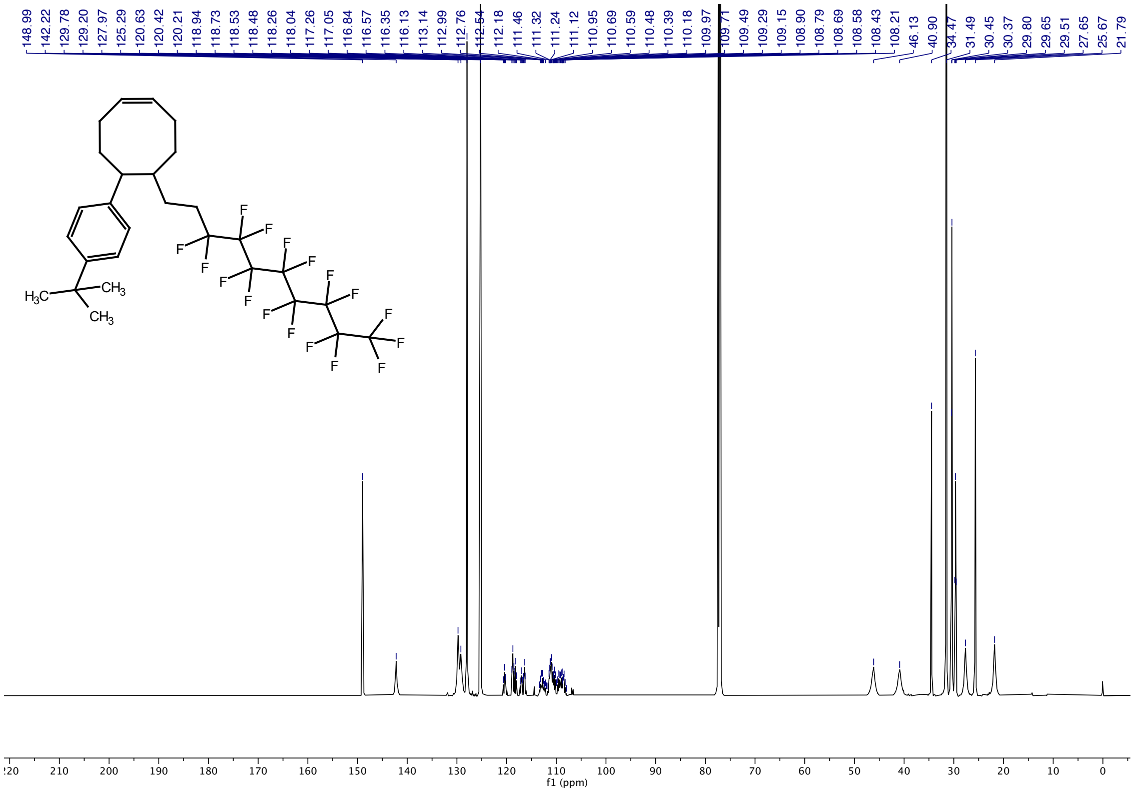

^{19}F NMR (376 MHz, $CDCl_3$) of **4n**:

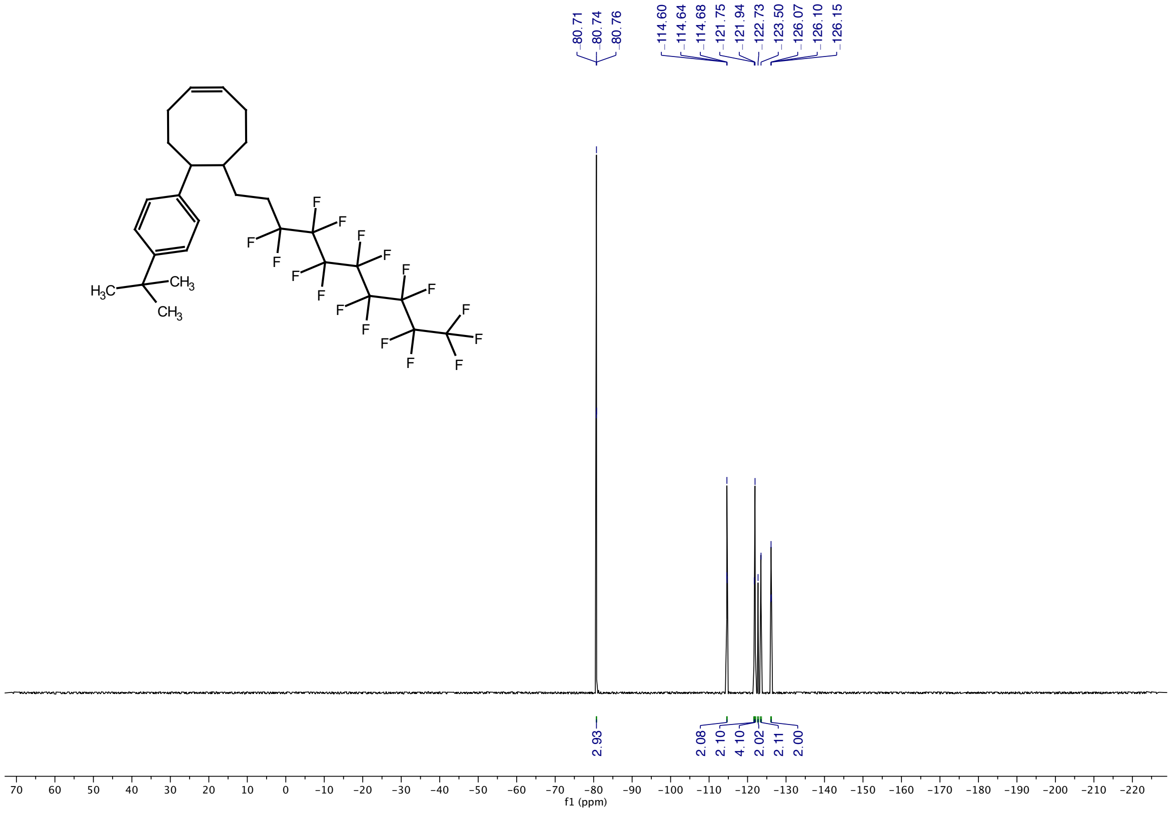

^{1}H NMR (600 MHz, $CDCl_3$) of **4n'**:

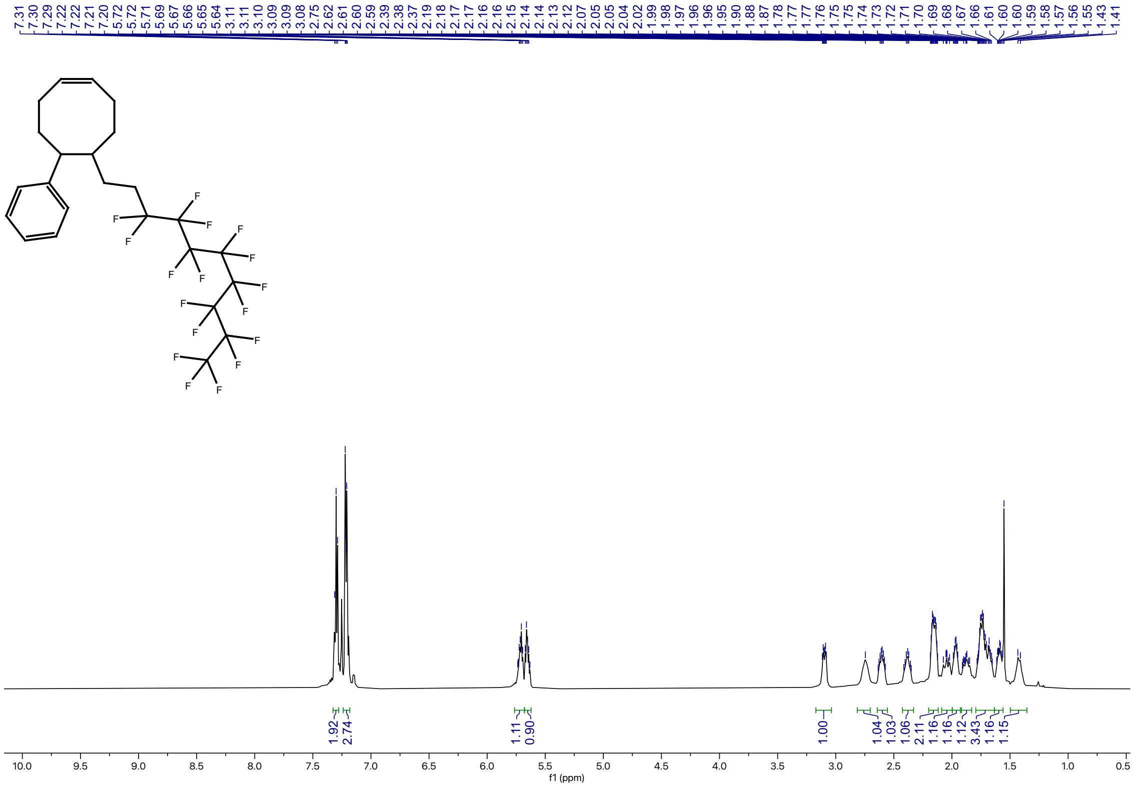

^{13}C NMR (151 MHz, $CDCl_3$) of **4n'**:

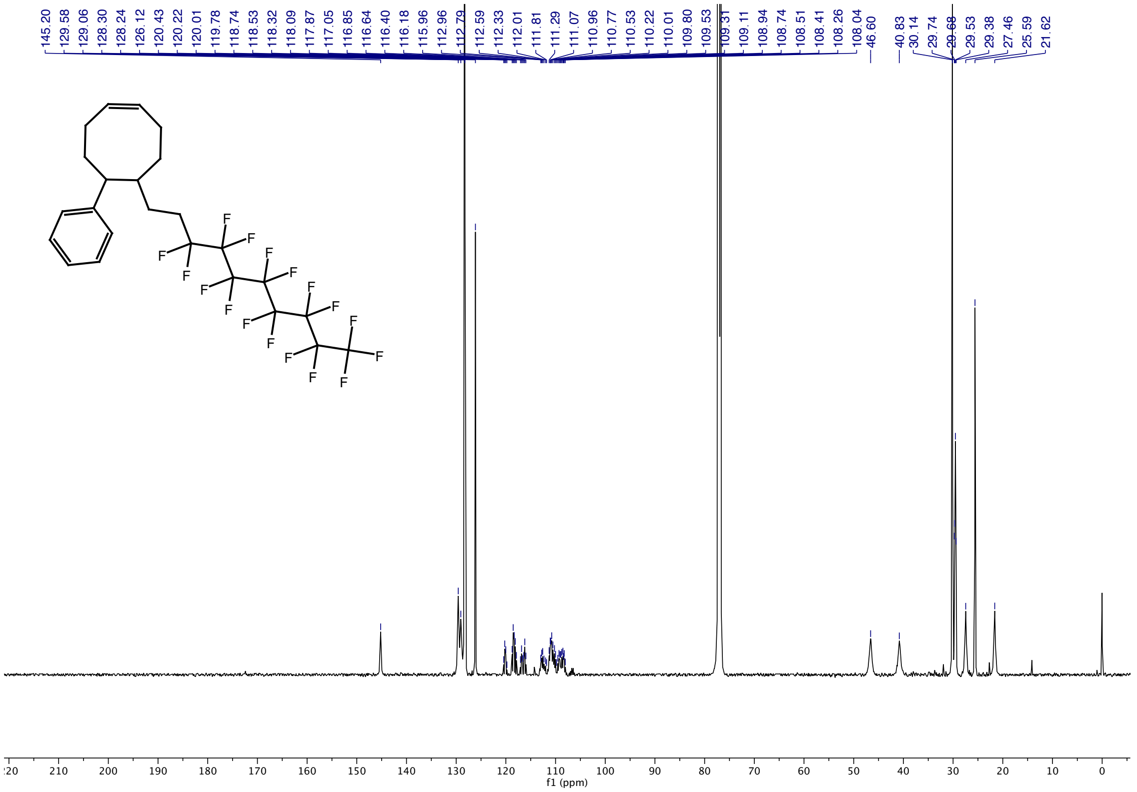

^{19}F NMR (376 MHz, $CDCl_3$) of **4n'**:

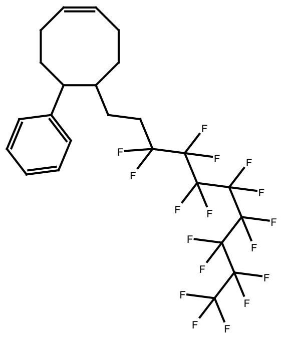

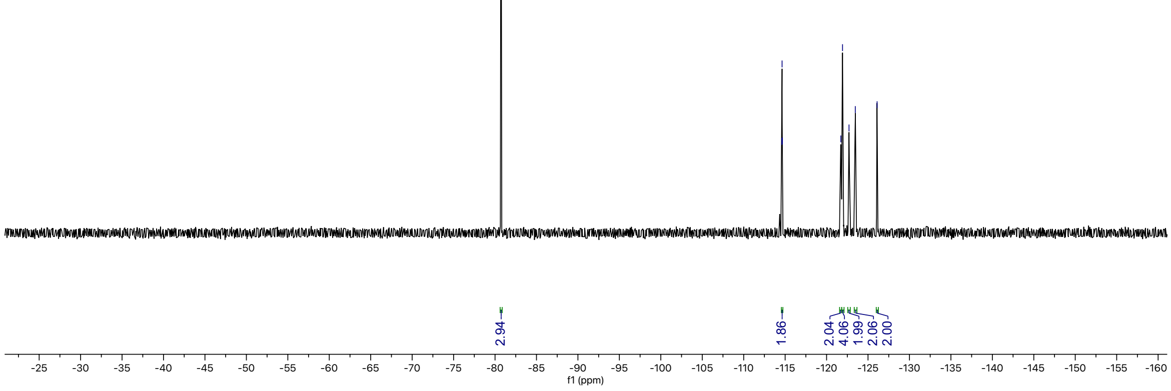

^{1}H NMR (400 MHz, $CDCl_3$) of **4o**:

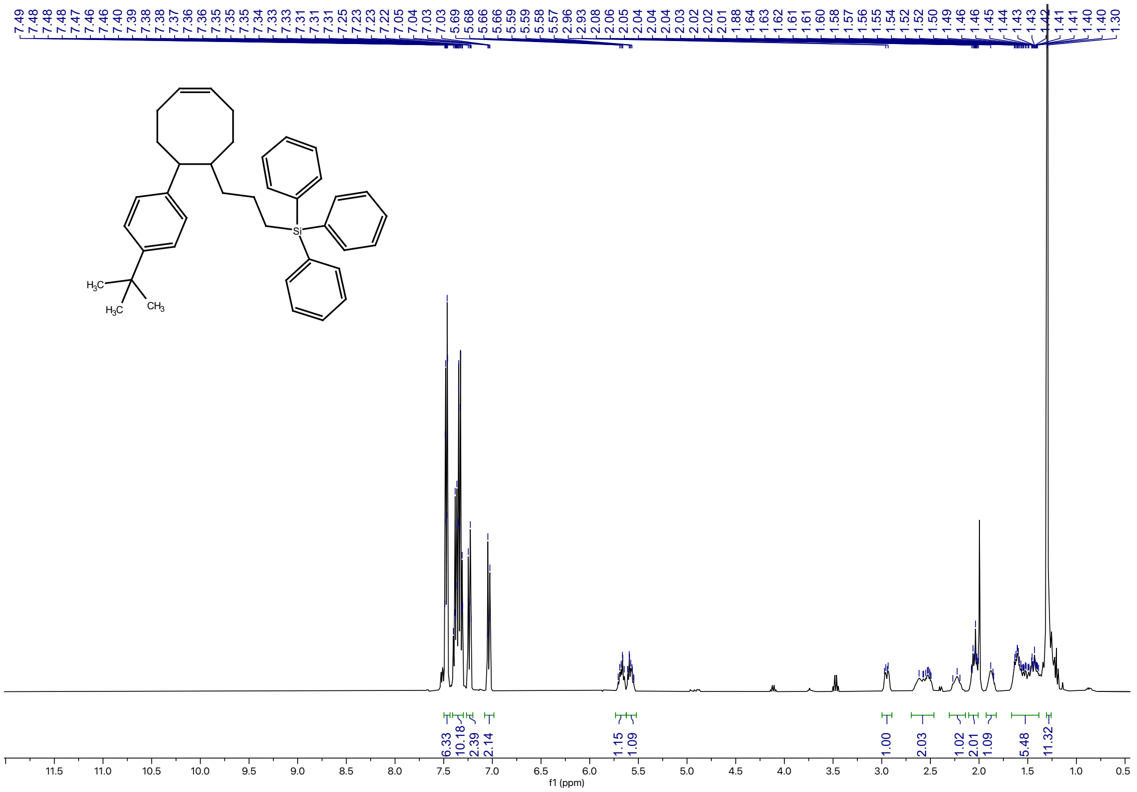

^{13}C NMR (101 MHz, $CDCl_3$) of **4o**:

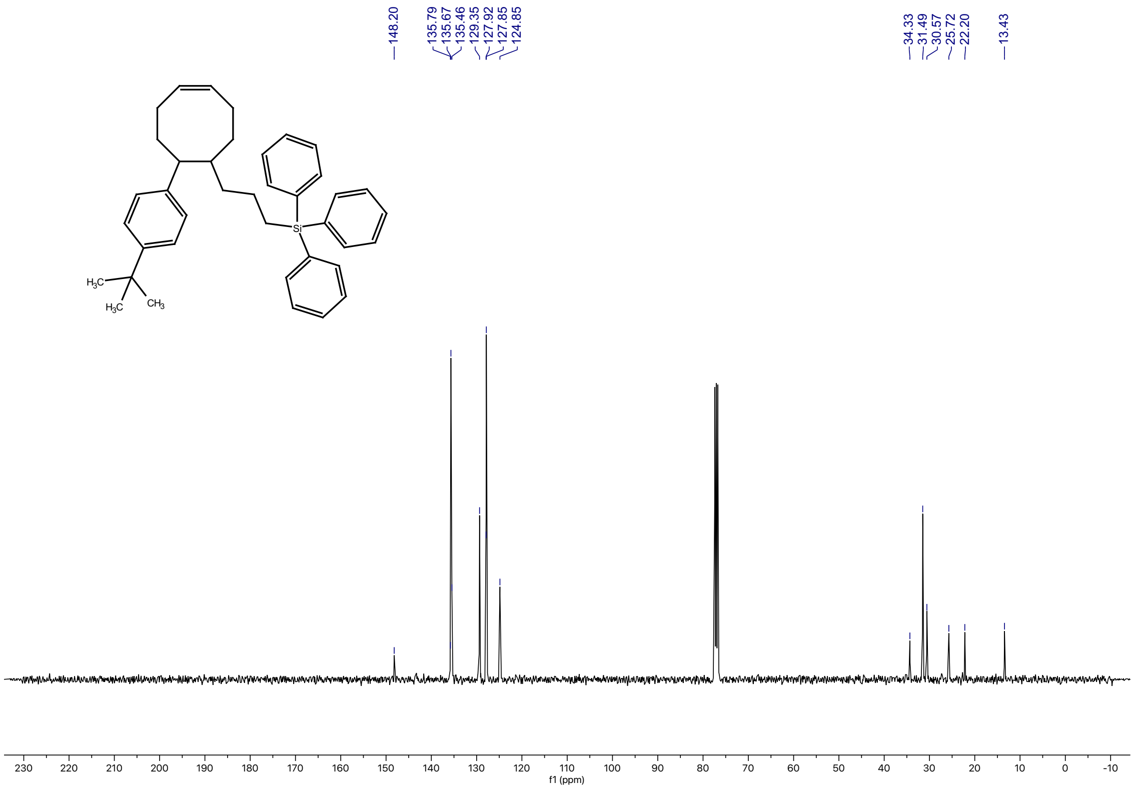

^{1}H NMR (600 MHz, $CDCl_3$) of **4o-epoxide**:

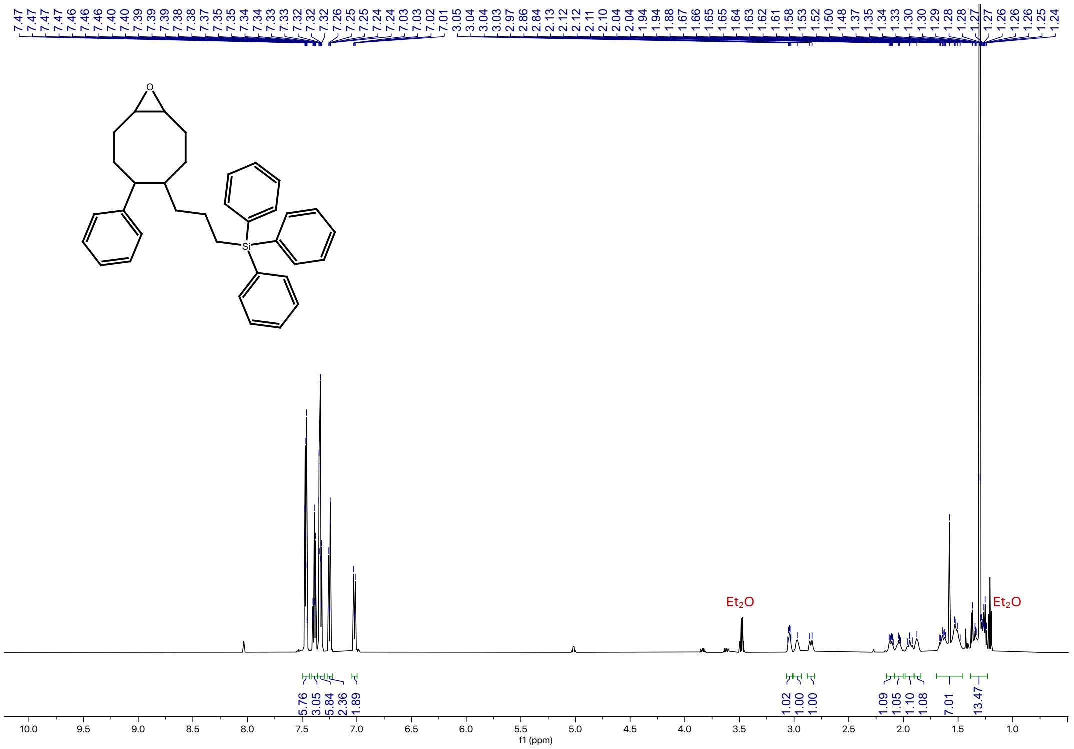

^{13}C NMR (151 MHz, $CDCl_3$) of **4o-epoxide:**

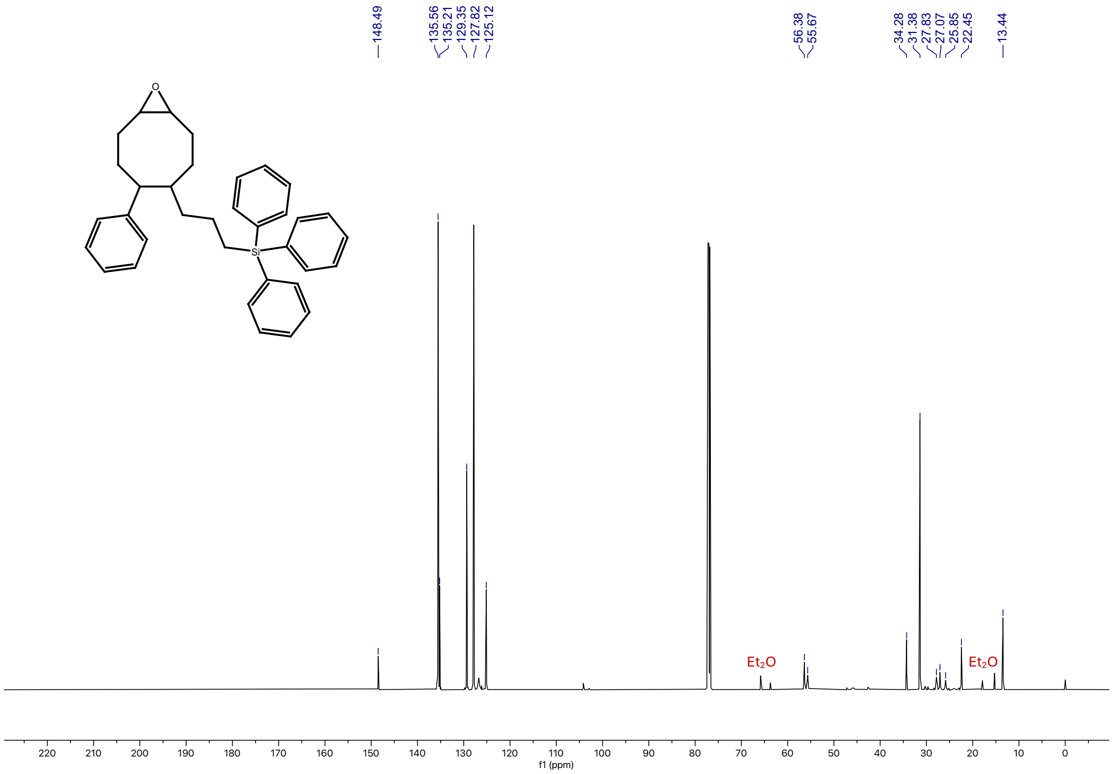

^{1}H NMR (400 MHz, $CDCl_3$) of **4p**:

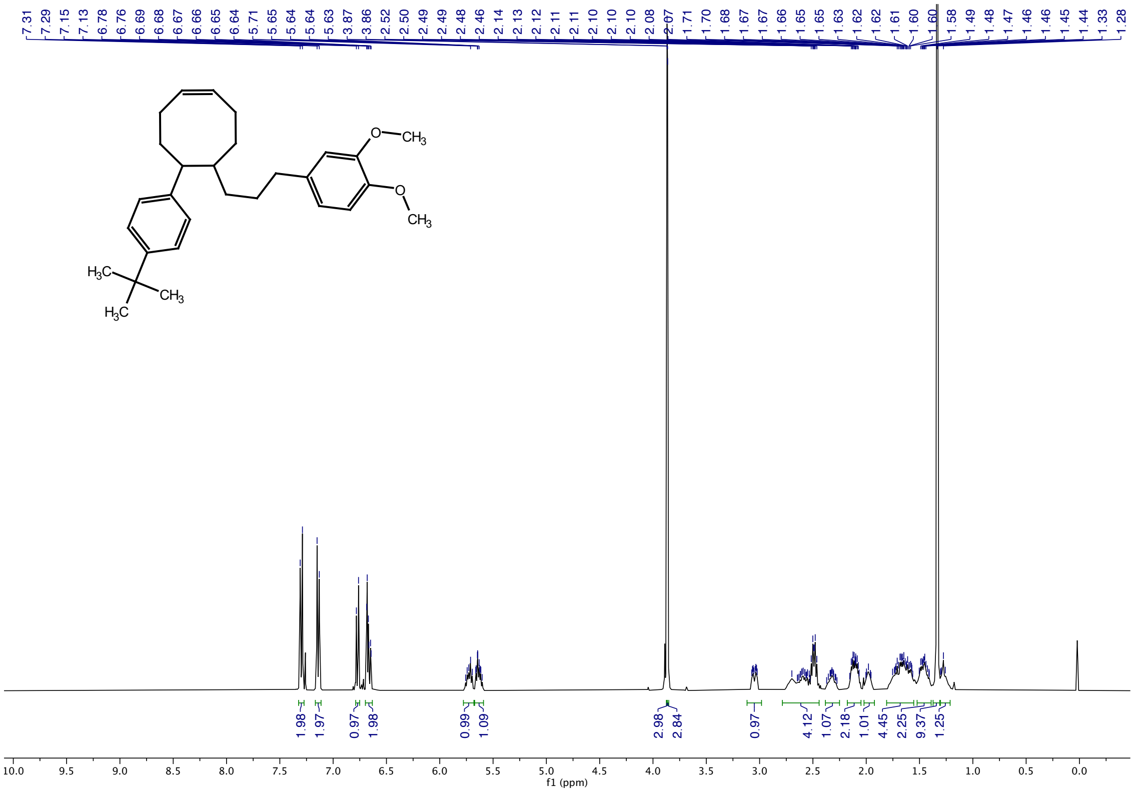

^{13}C NMR (101 MHz, $CDCl_3$) of **4p**:

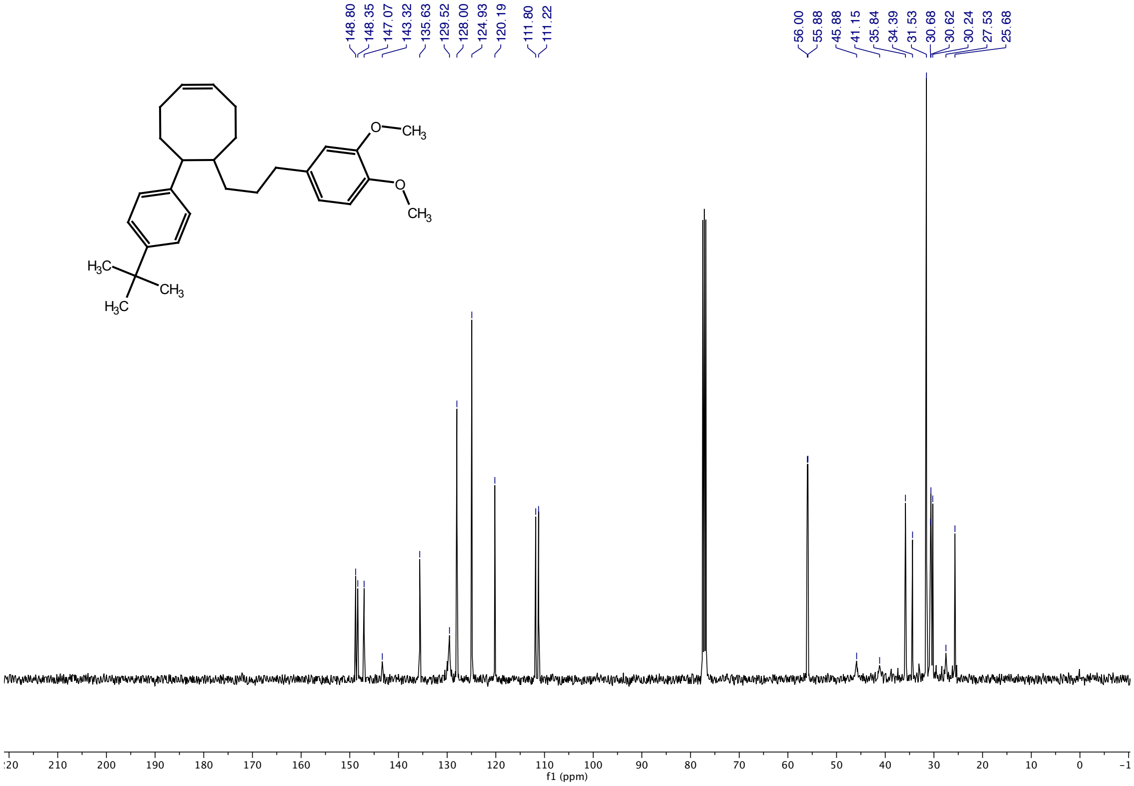

^{1}H NMR (400 MHz, $CDCl_3$) of **4q**:

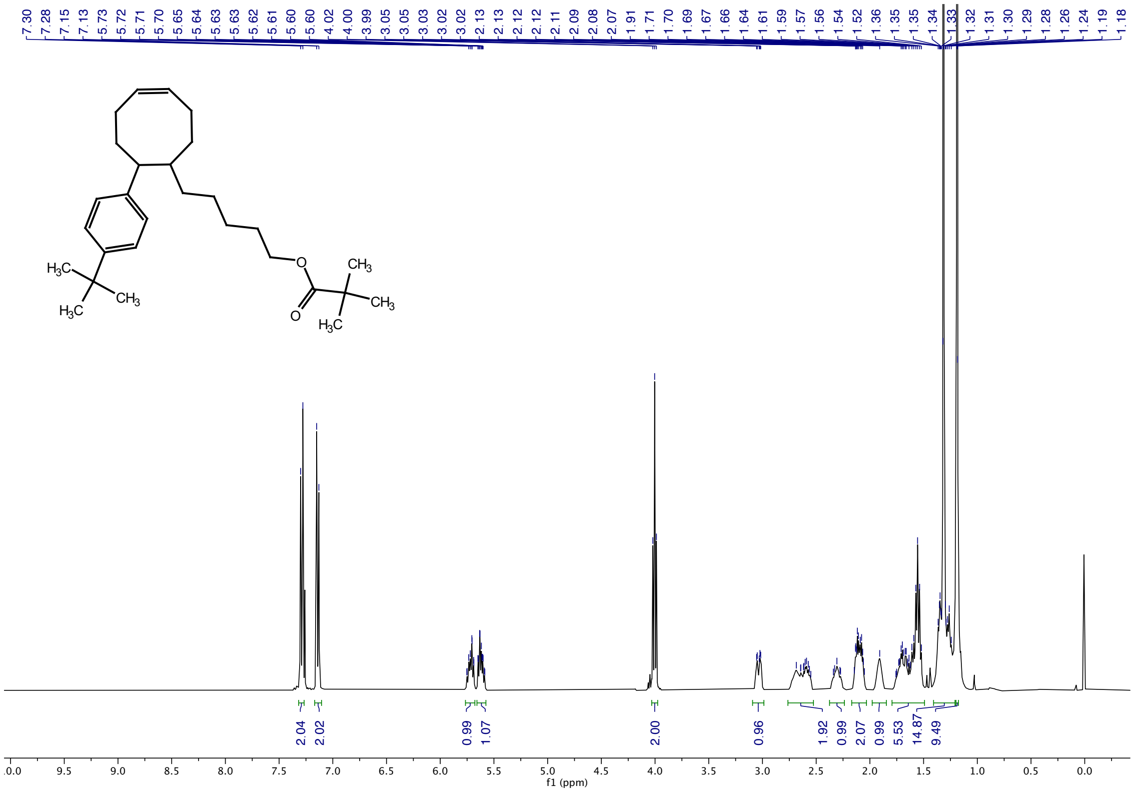

^{13}C NMR (101 MHz, $CDCl_3$) of **4q**:

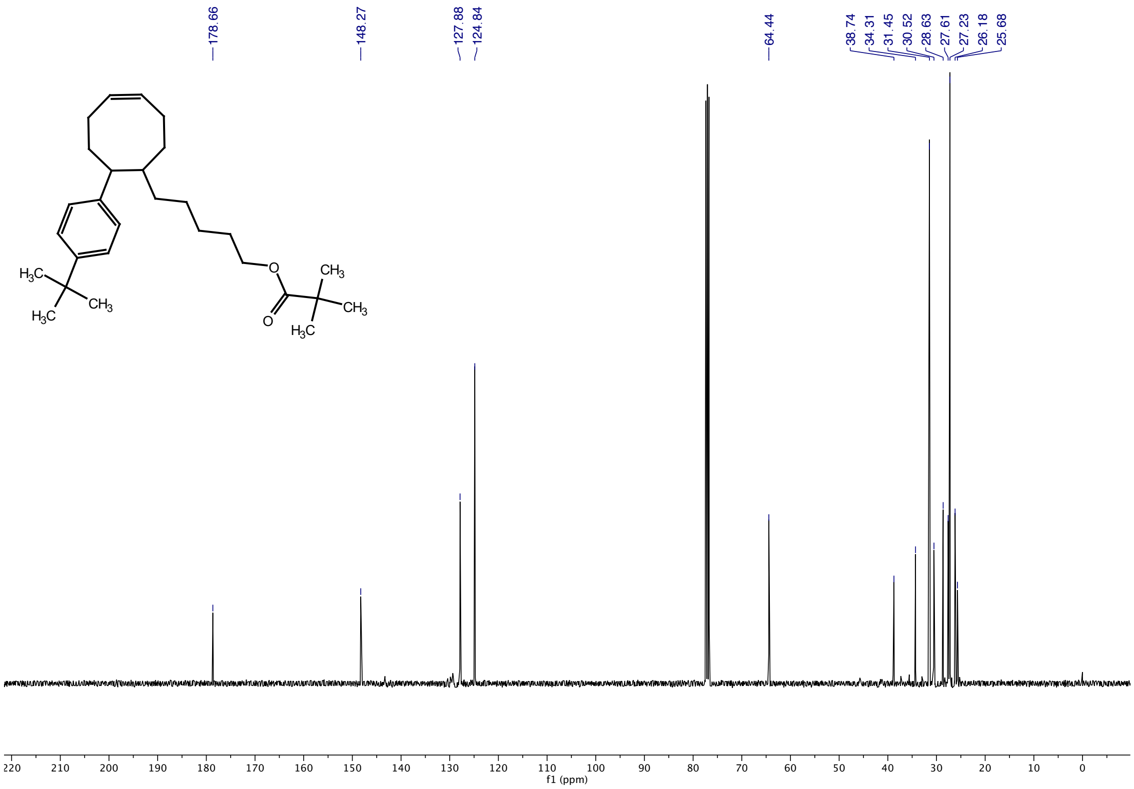

^{1}H NMR (400 MHz, $CDCl_3$) of **4r**:

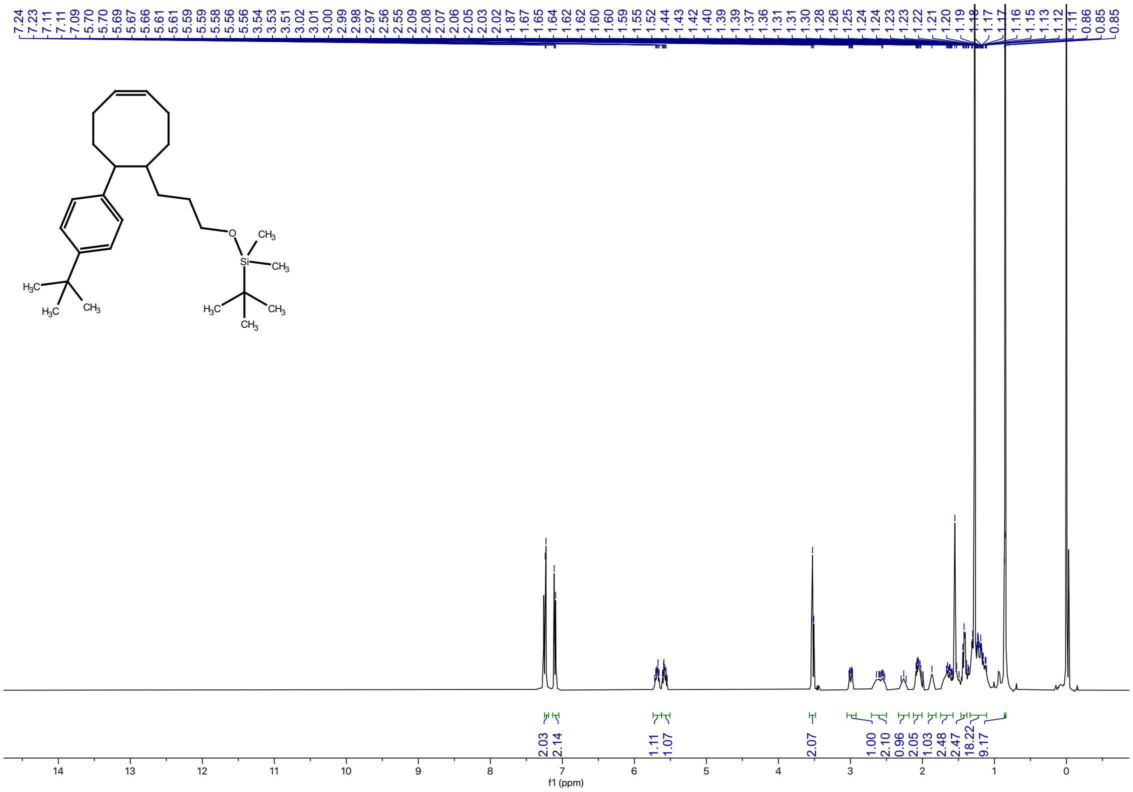

^{13}C NMR (101 MHz, $CDCl_3$) of **4r**:

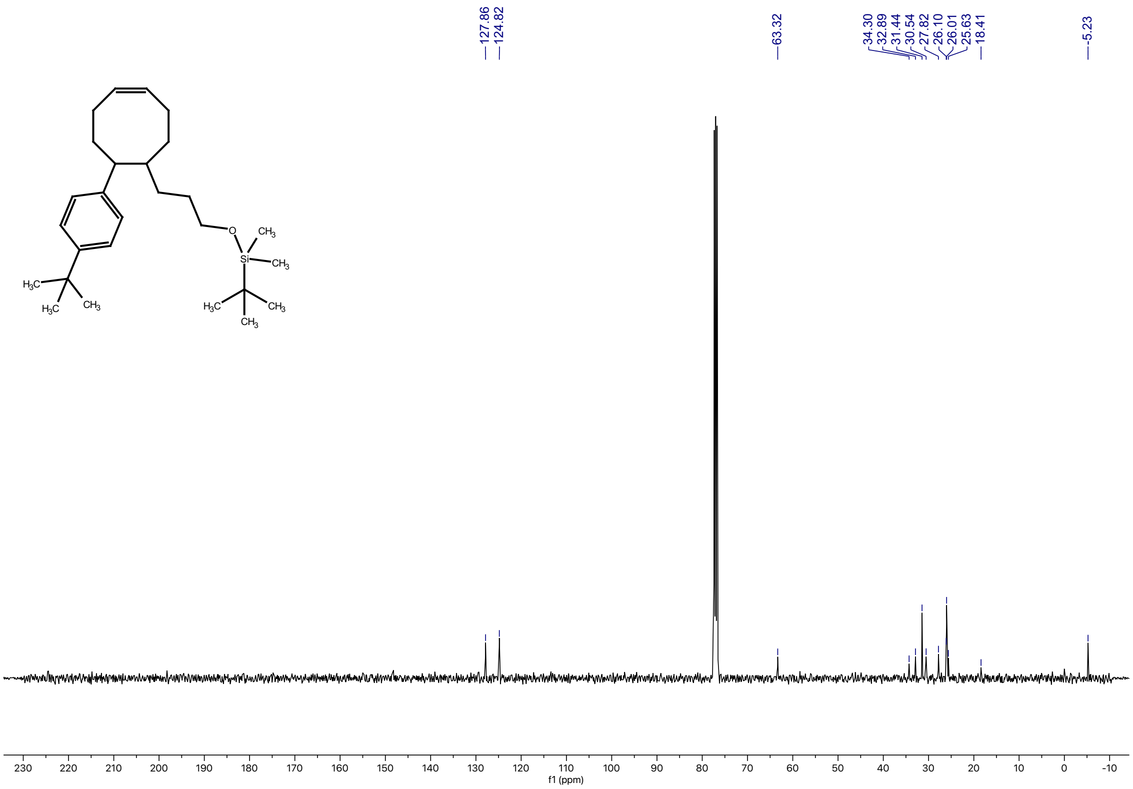

^{1}H NMR (400 MHz, $CDCl_3$) of **4s**:

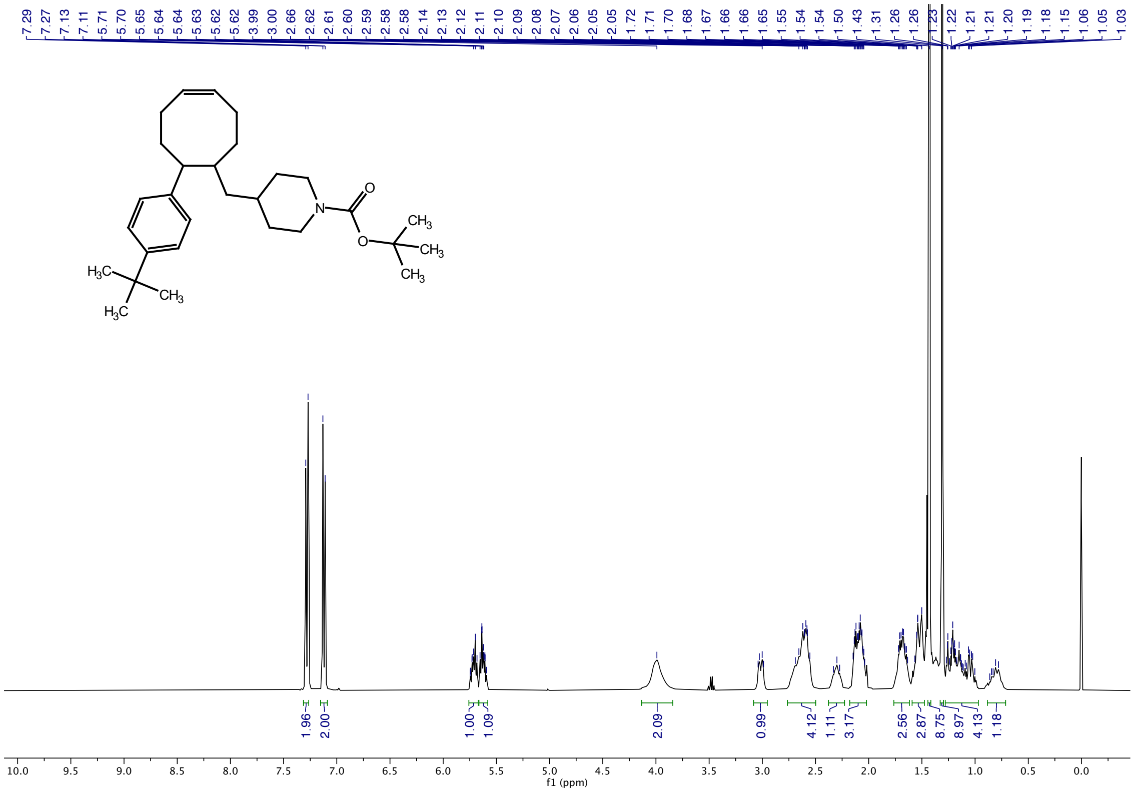

^{13}C NMR (101 MHz, $CDCl_3$) of **4s**:

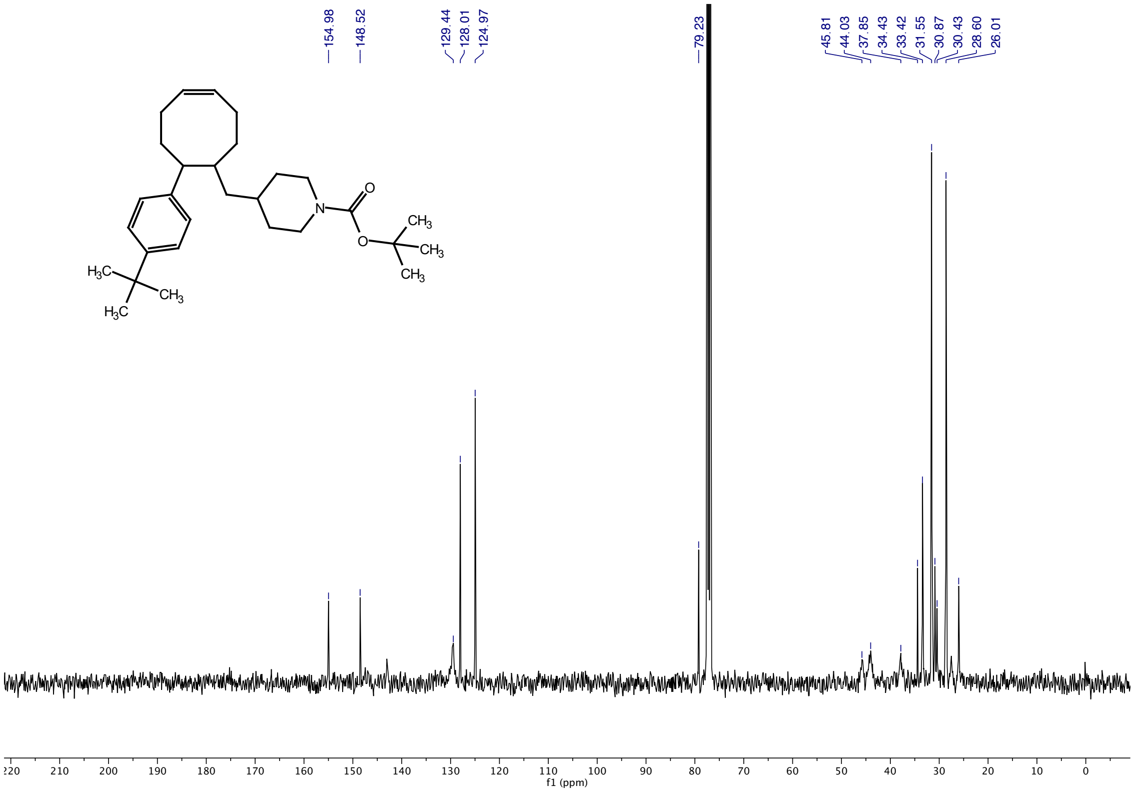

^{1}H NMR (400 MHz, $CDCl_3$) of **4s'**:

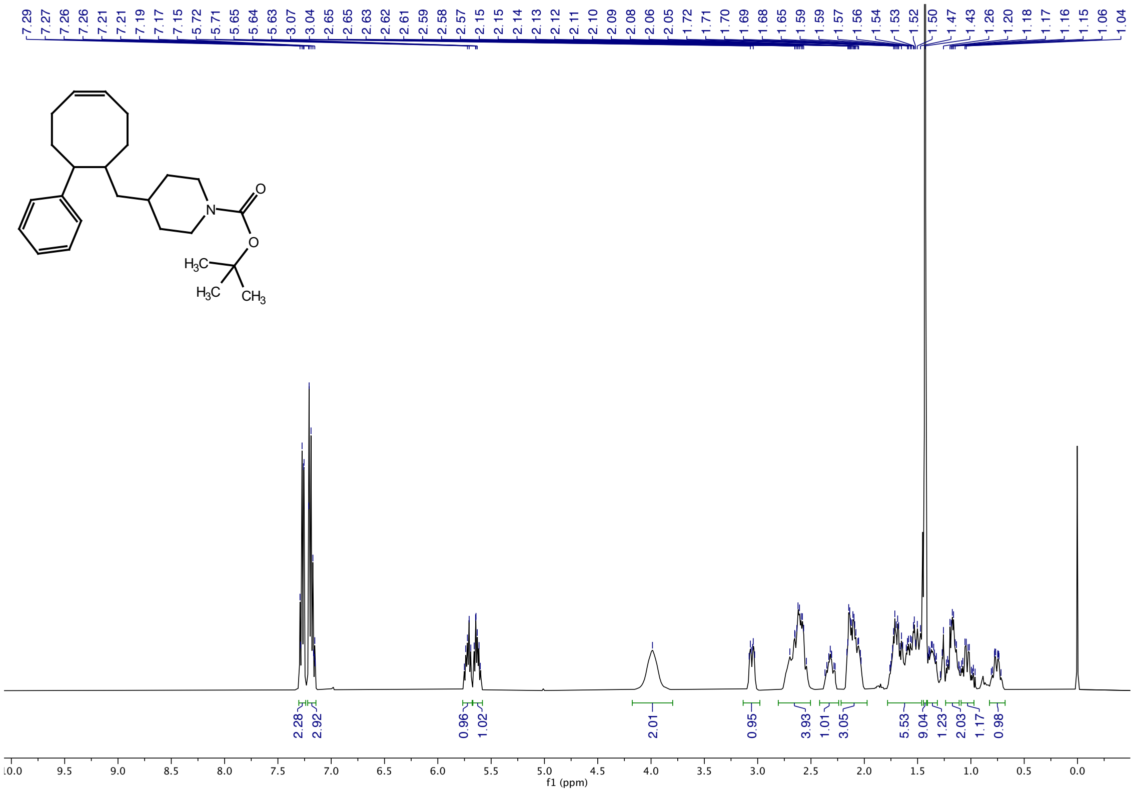

^{13}C NMR (101 MHz, $CDCl_3$) of **4s'**:

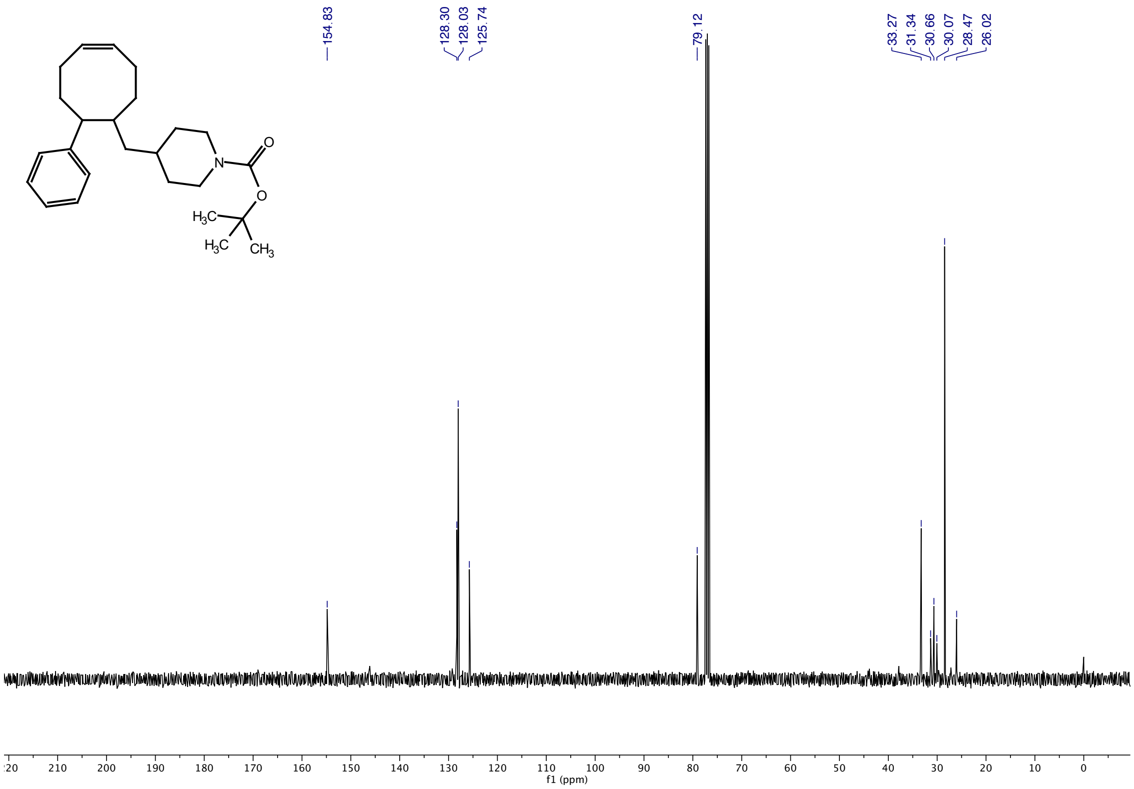

^{1}H NMR (400 MHz, $CDCl_3$) of **4t**:

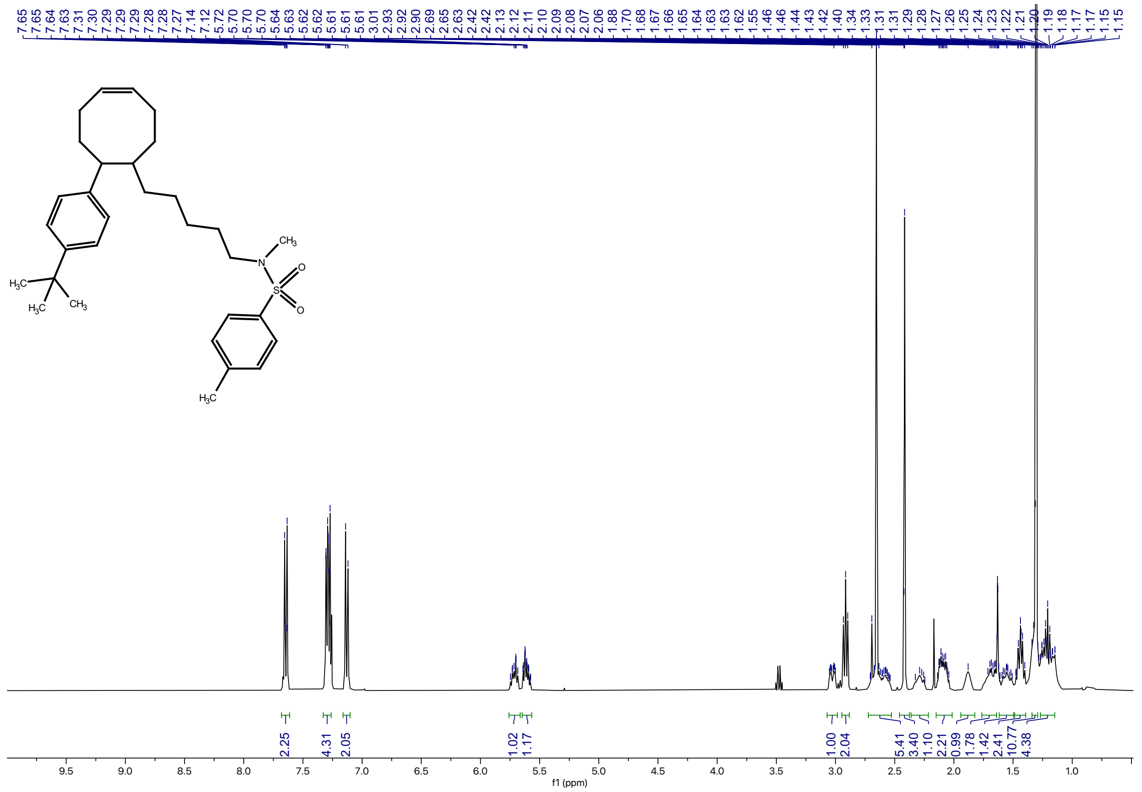

^{13}C NMR (101 MHz, $CDCl_3$) of **4t**:

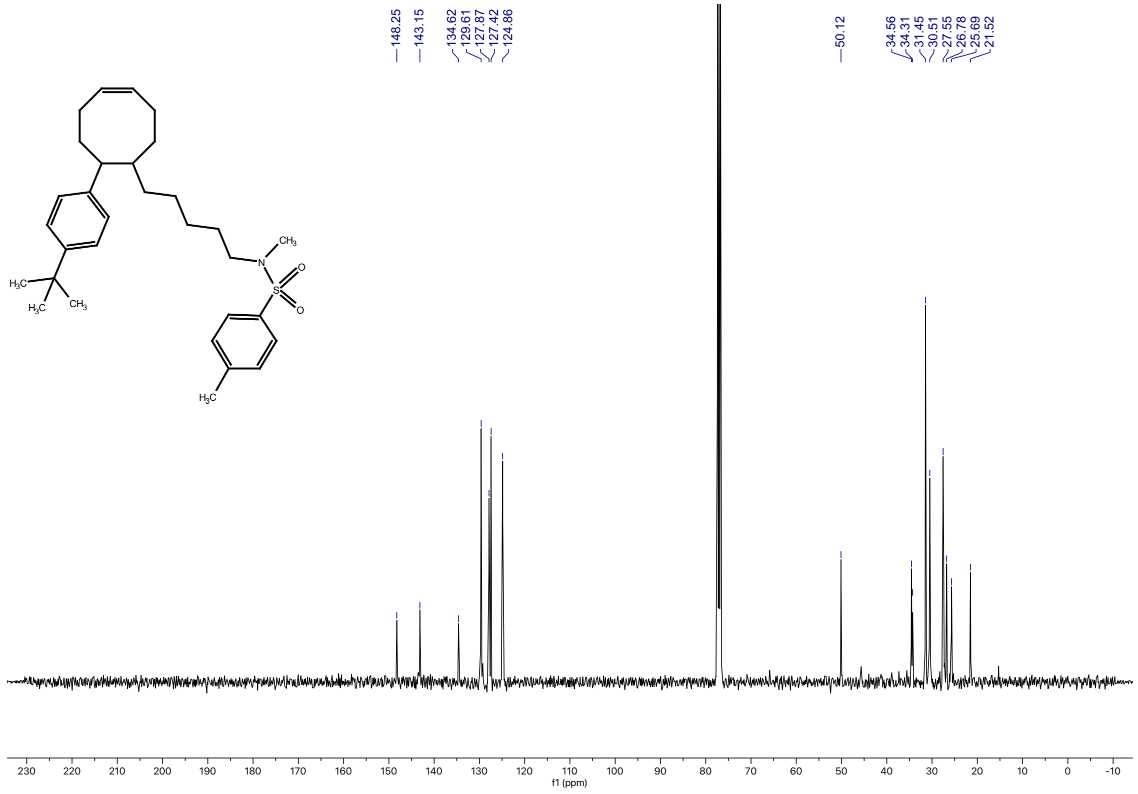

^{1}H NMR (400 MHz, $CDCl_3$) of **4u**:

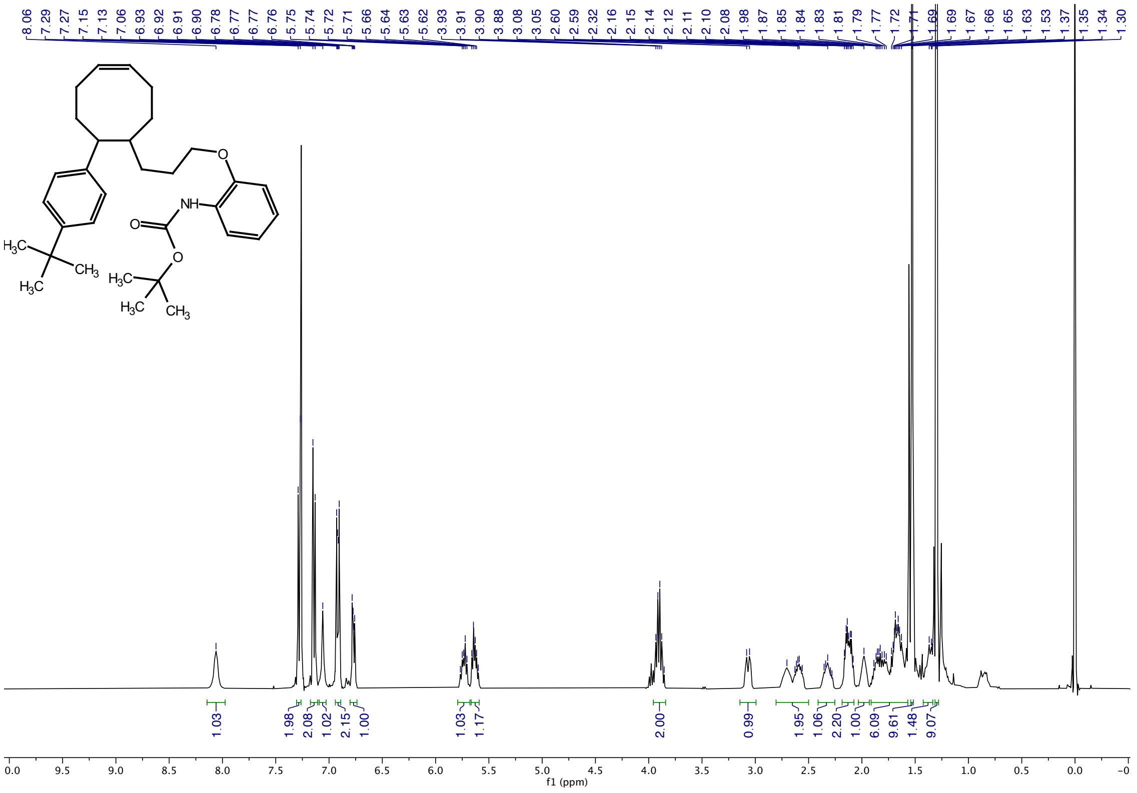

^{13}C NMR (101 MHz, $CDCl_3$) of **4u**:

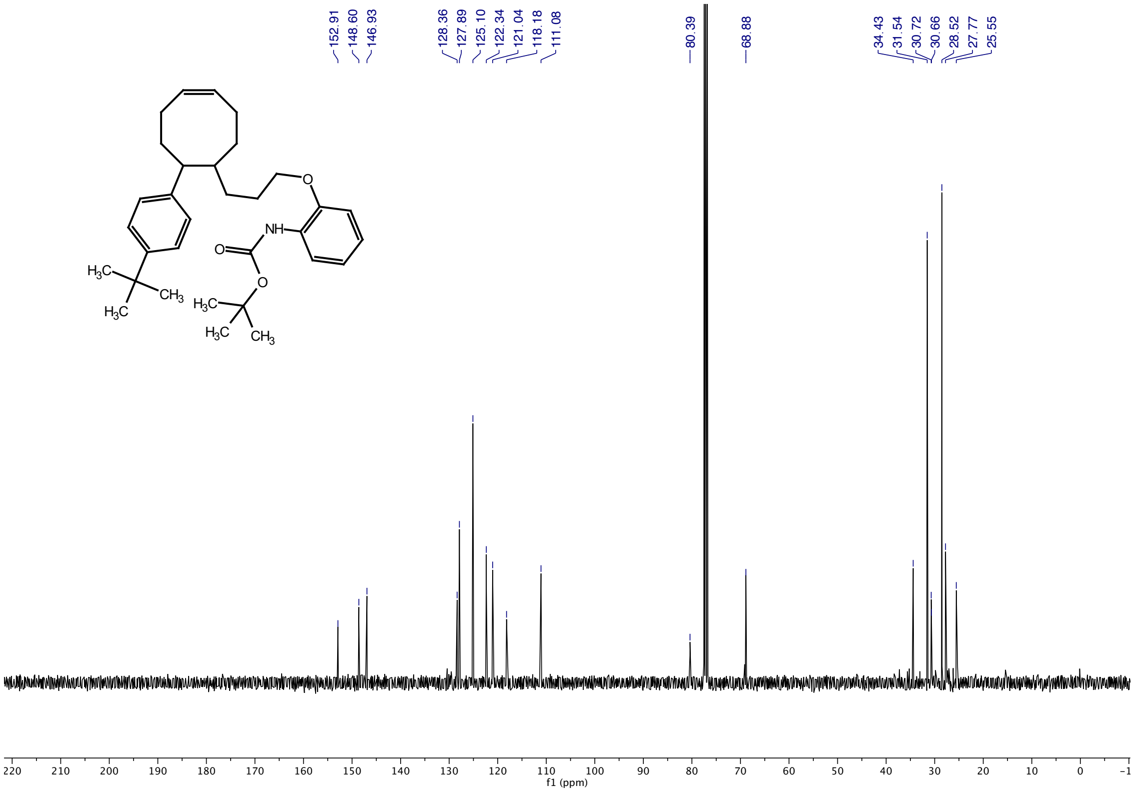

^{1}H NMR (400 MHz, $CDCl_3$) of **4v**:

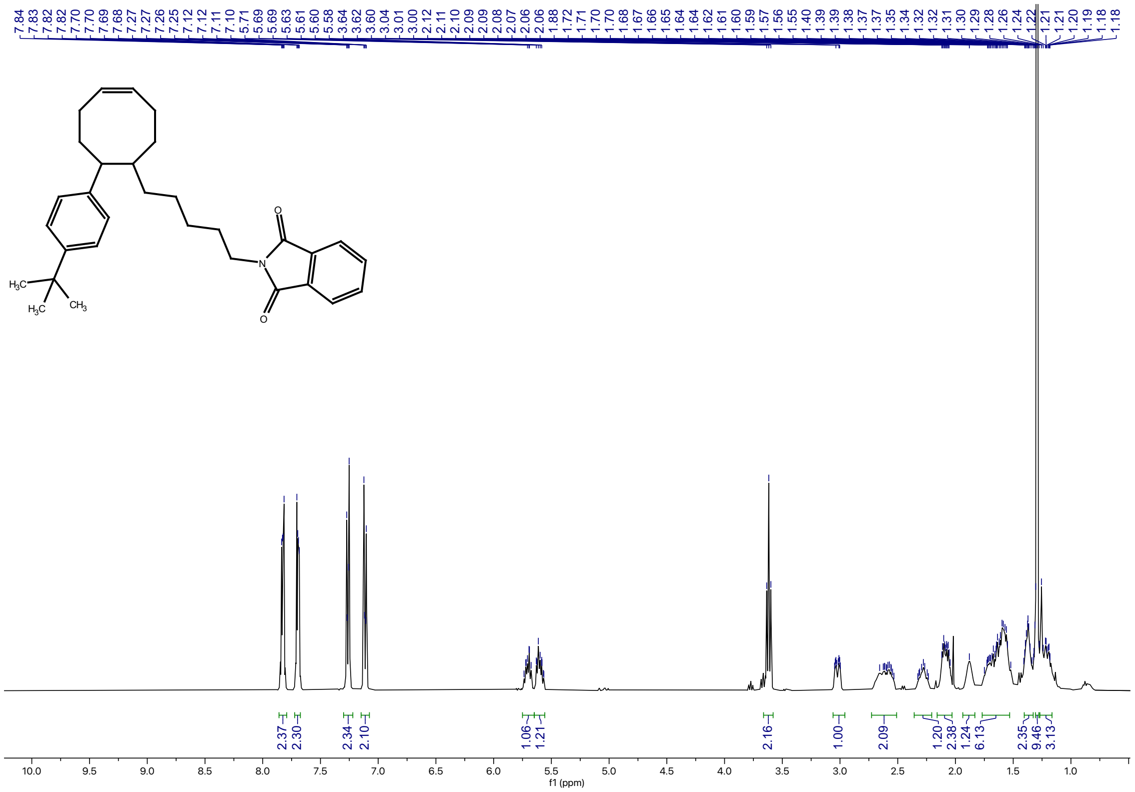

^{13}C NMR (101 MHz, $CDCl_3$) of **4v**:

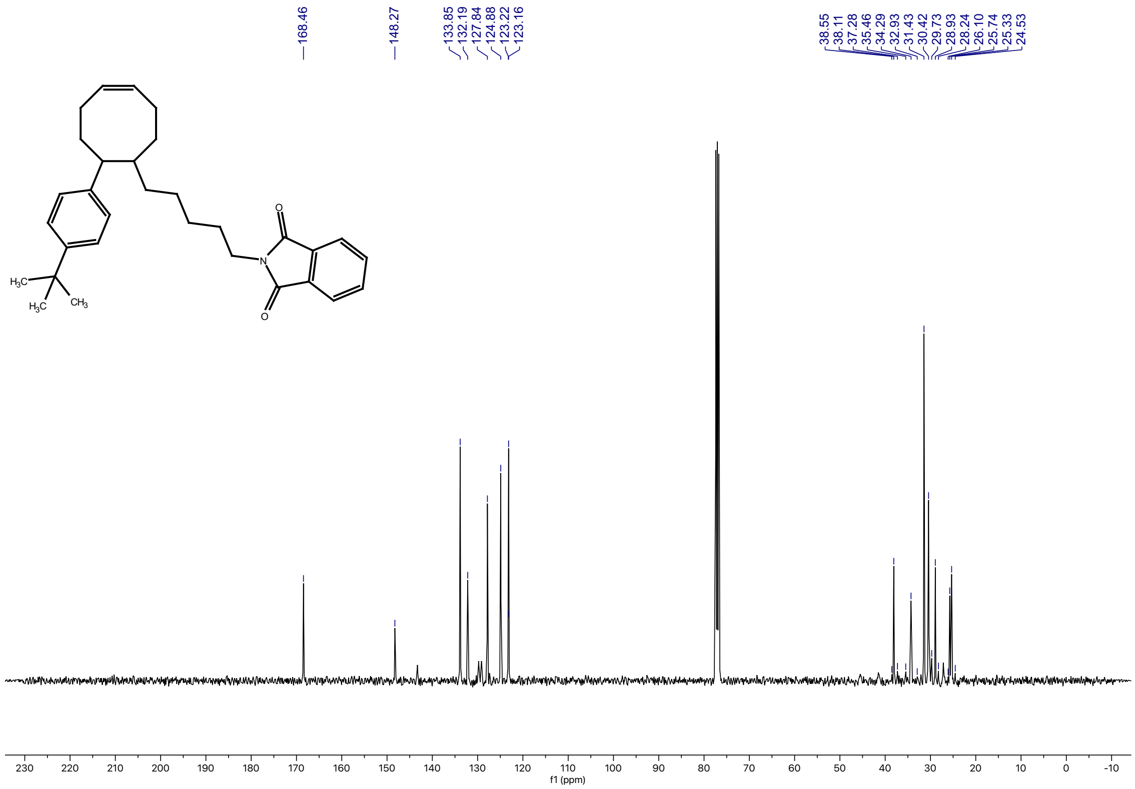

^{1}H NMR (400 MHz, $CDCl_3$) of **4x**:

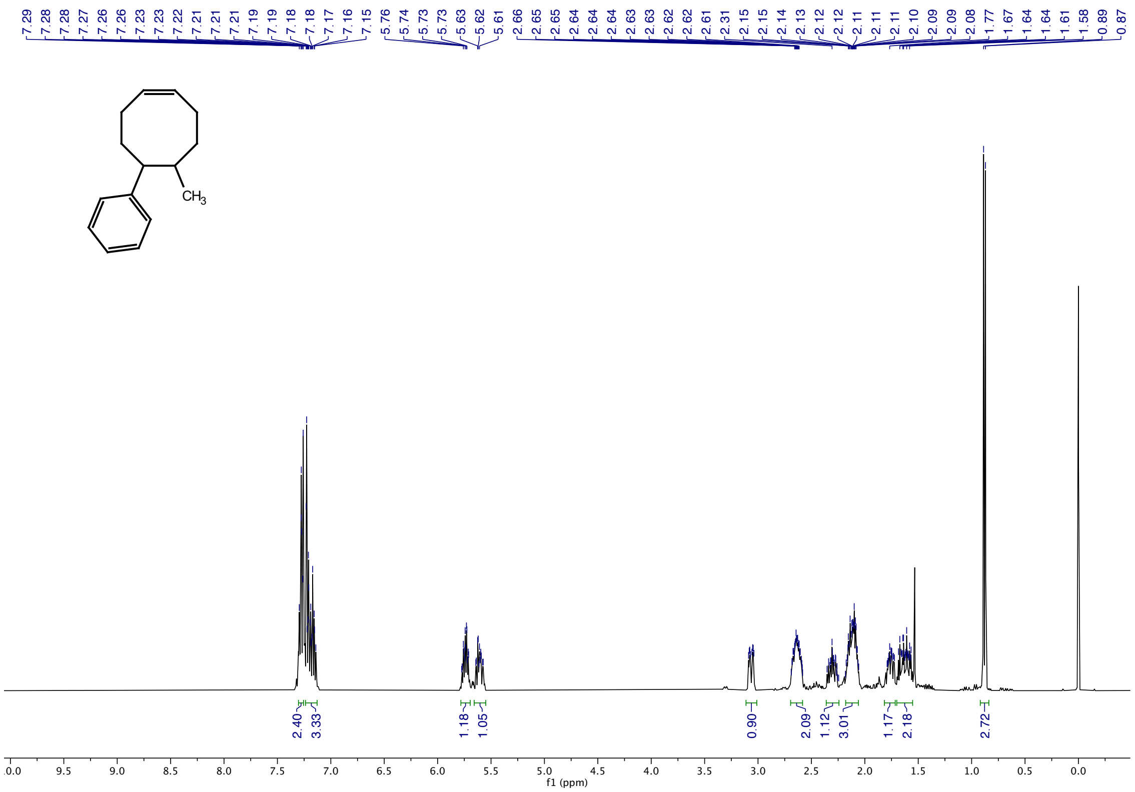

^{13}C NMR (101 MHz, $CDCl_3$) of **4x**:

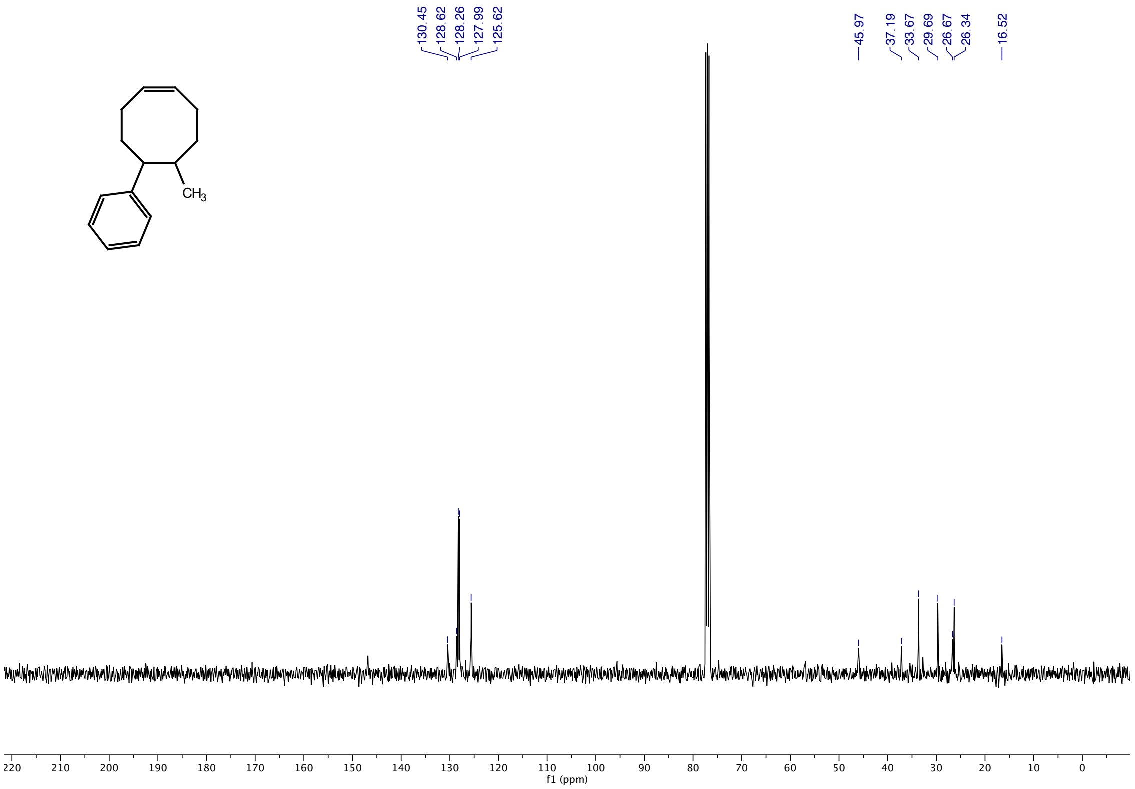

^{1}H NMR (500 MHz, $CDCl_3$) of **P4a**:

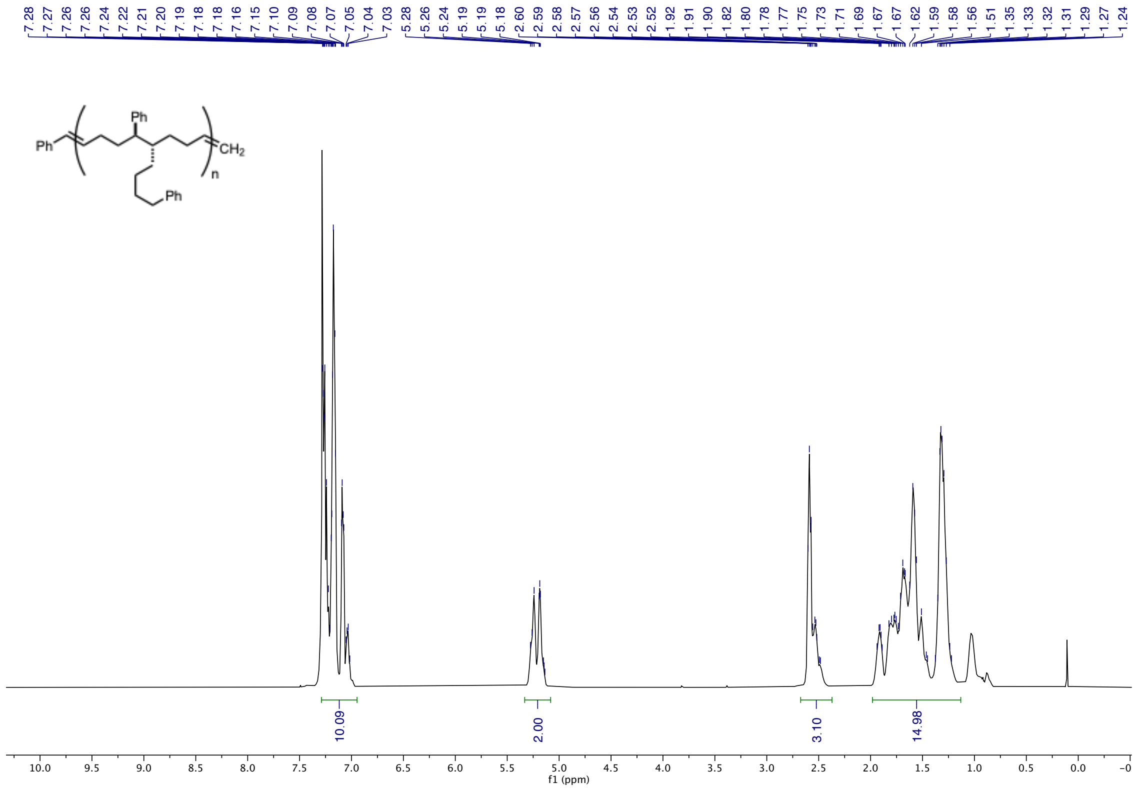

^{13}C NMR (126 MHz, $CDCl_3$) of **P4a**:

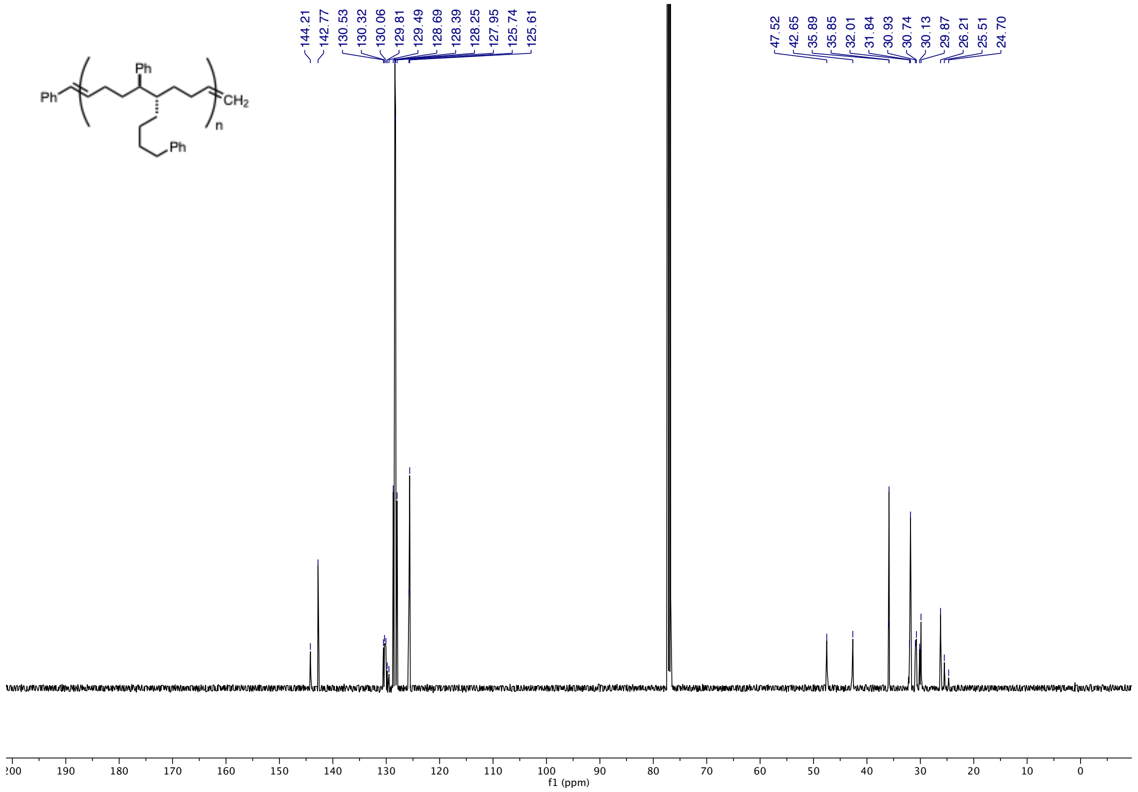

^{1}H NMR (500 MHz, $CDCl_3$) of **P4i**:

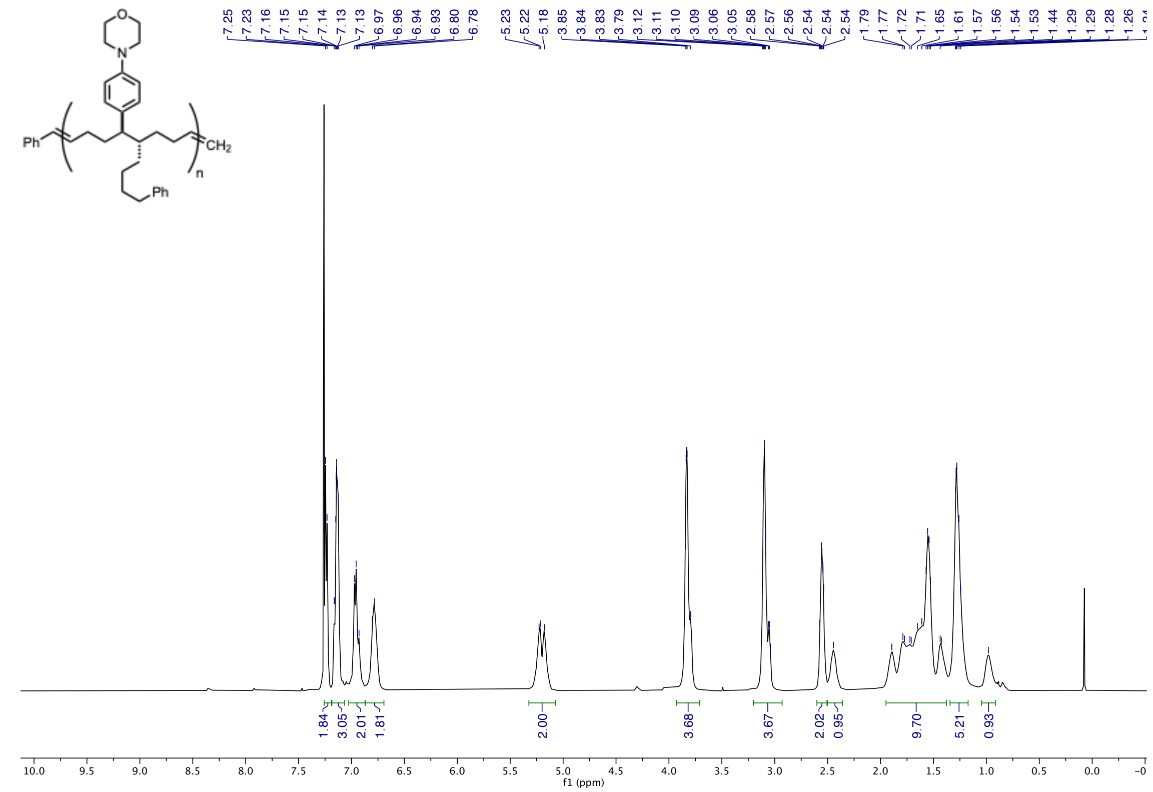

^{13}C NMR (126 MHz, $CDCl_3$) of **P4i**:

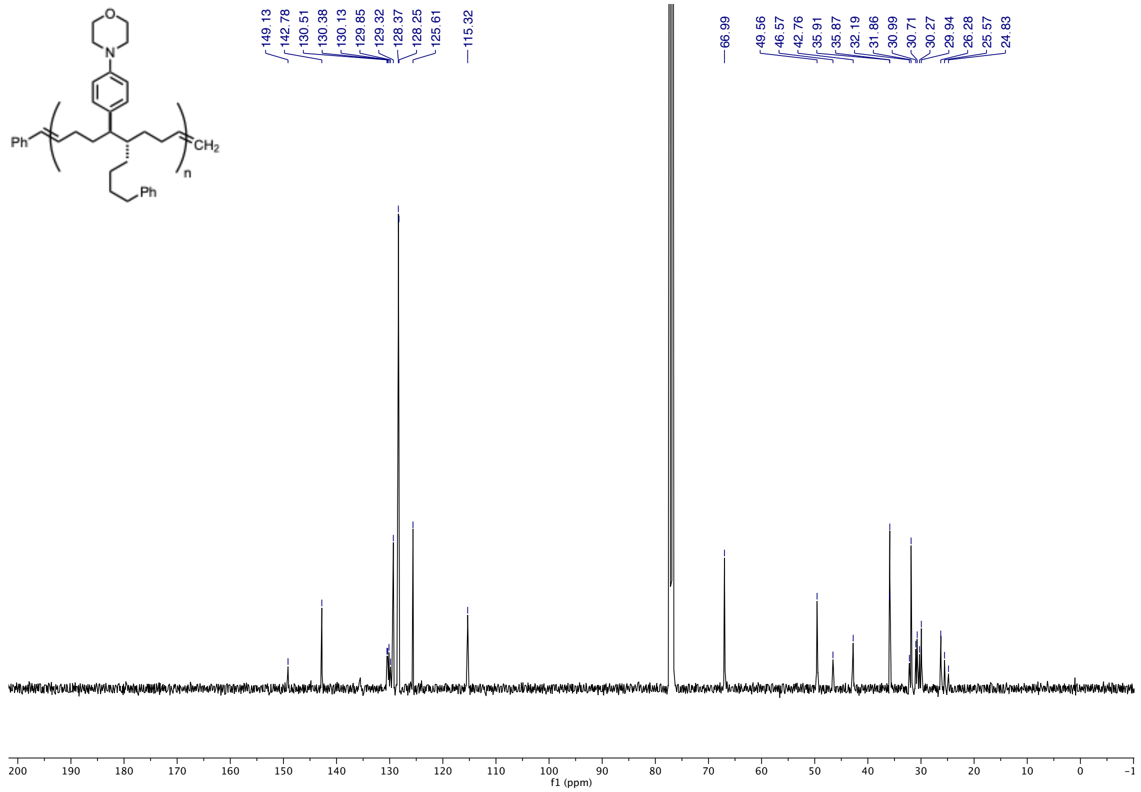

^{1}H NMR (500 MHz, $CDCl_3$) of **P4n'**:

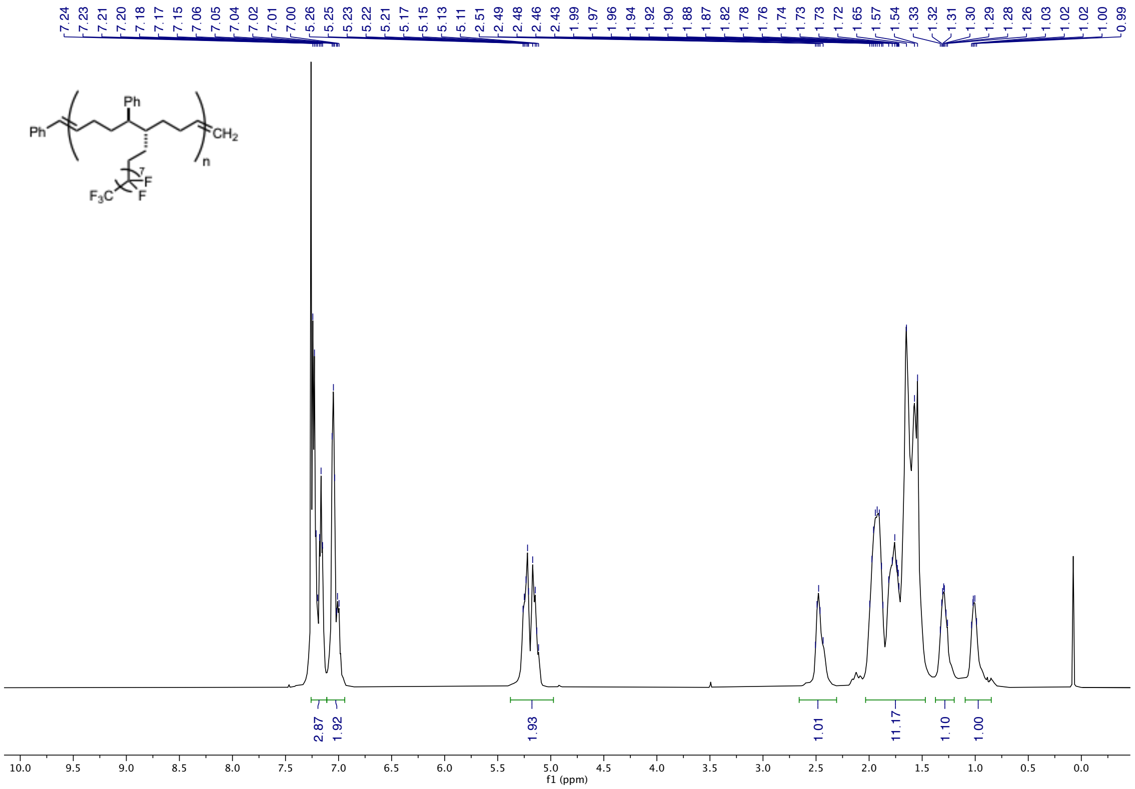

^{13}C NMR (126 MHz, $CDCl_3$) of **P4n'**:

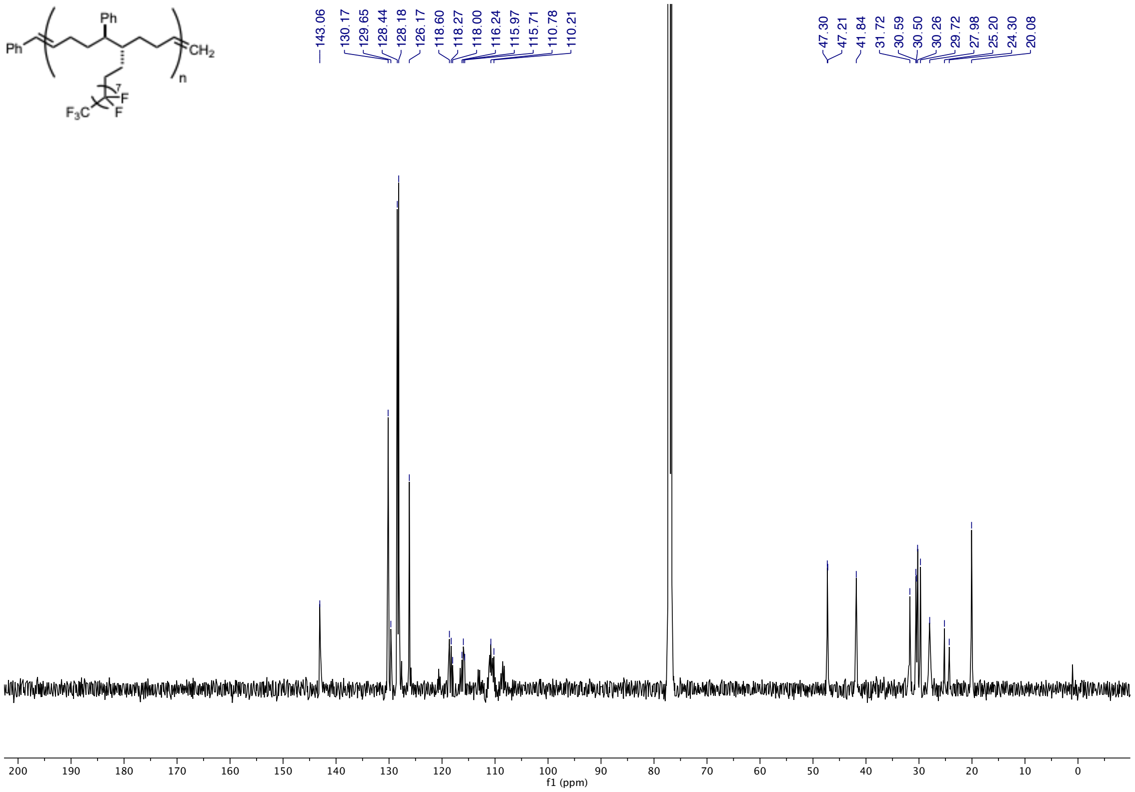

^{1}H NMR (500 MHz, $CDCl_3$) of **P4s'**:

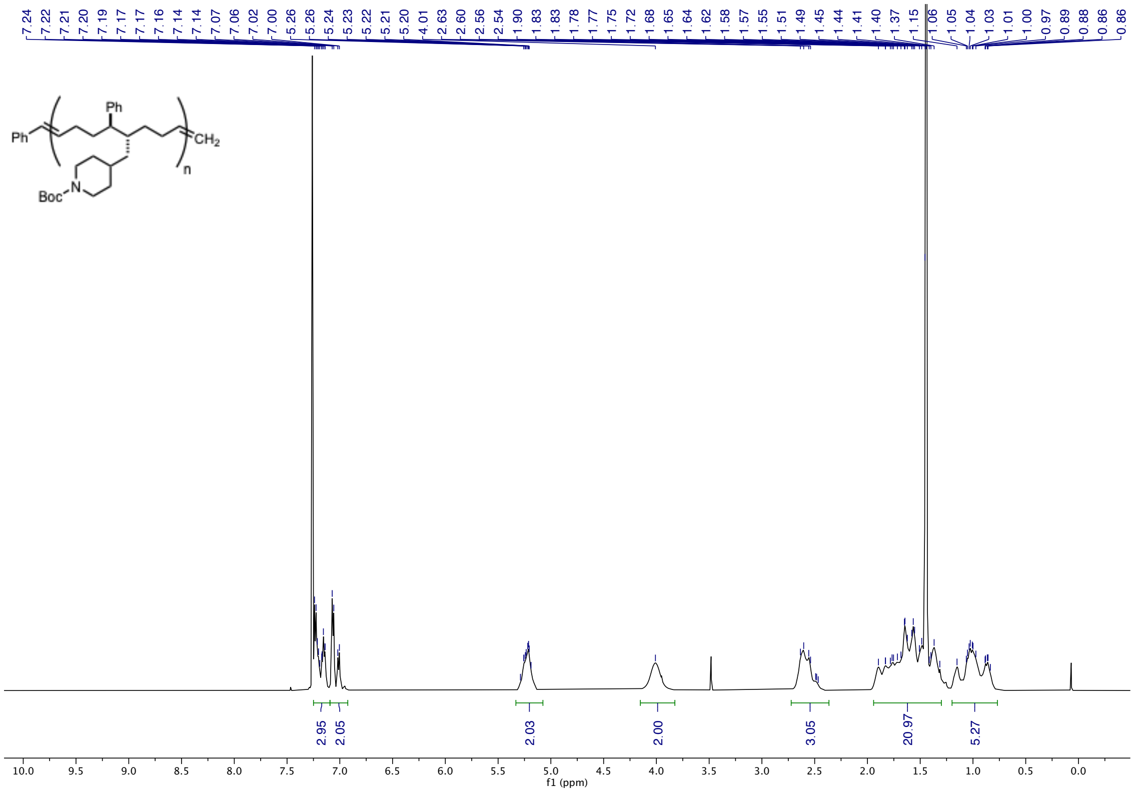

^{13}C NMR (126 MHz, $CDCl_3$) of **P4s'**:

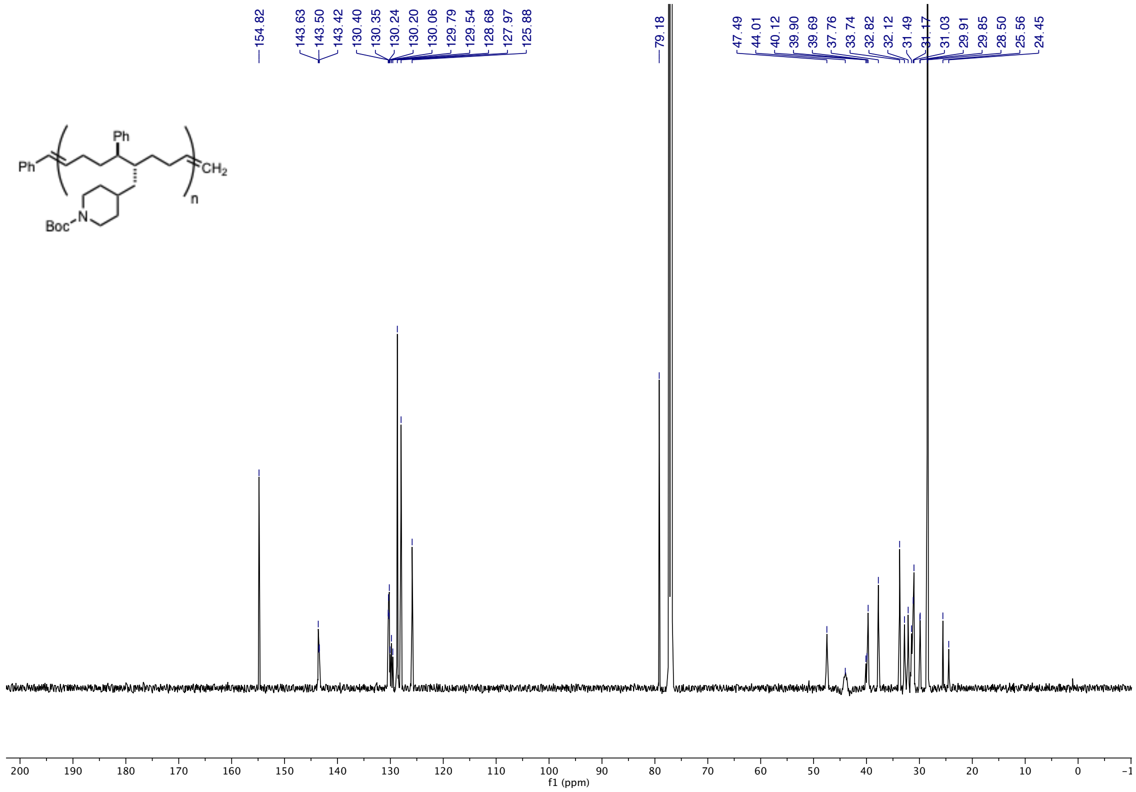

^{1}**H NMR** (500 MHz, $CDCl_3$) of **P4x**:

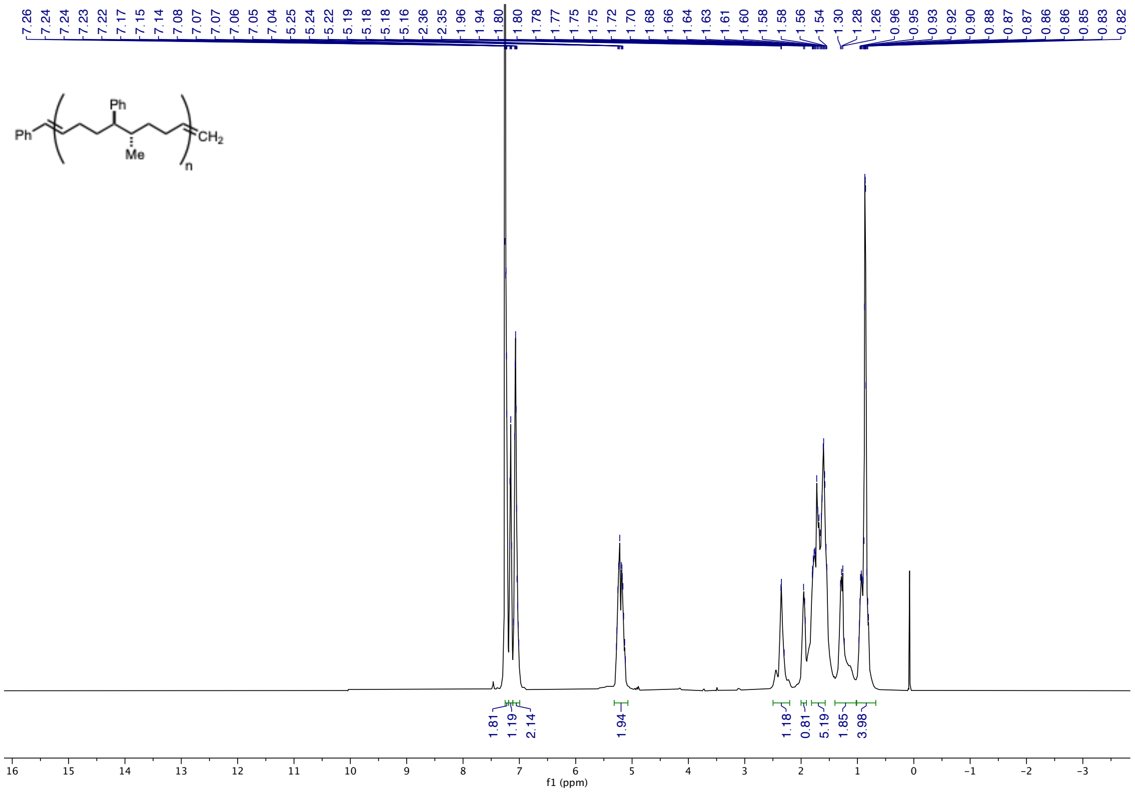

^{13}**C NMR** (126 MHz, $CDCl_3$) of **P4x**:

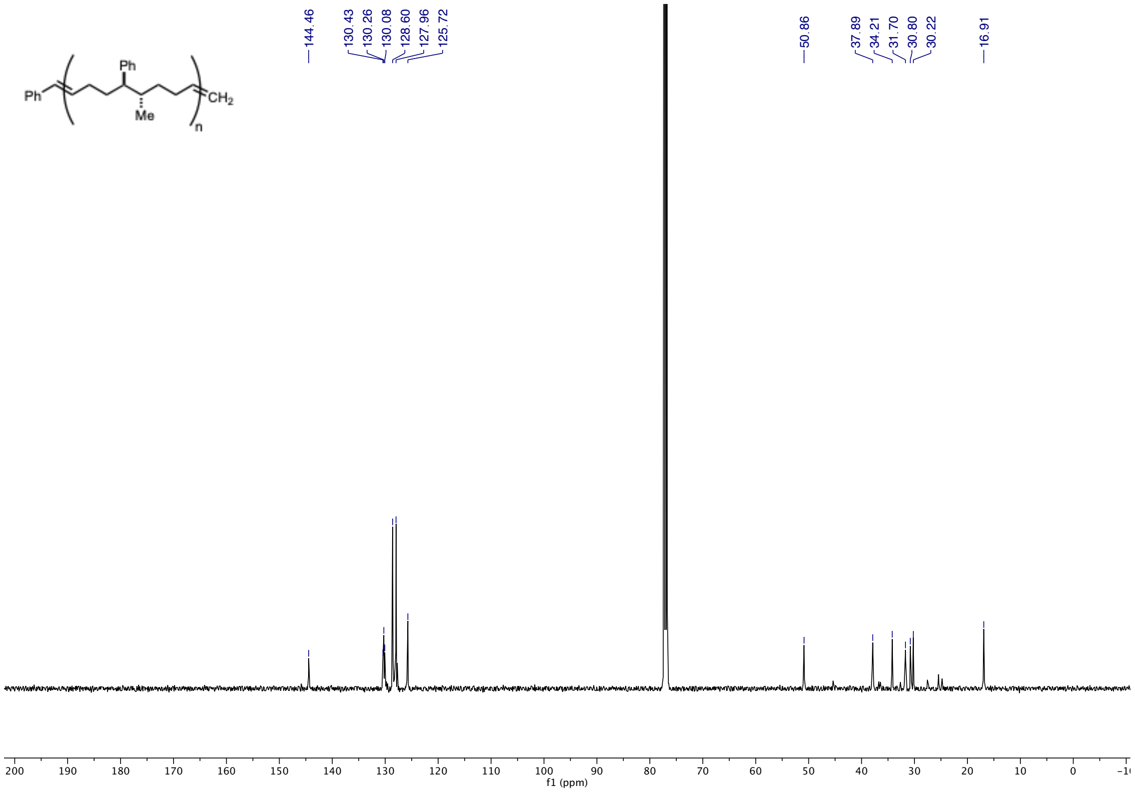

^{1}H NMR (500 MHz, $CDCl_3$) of **P4x-H_2**:

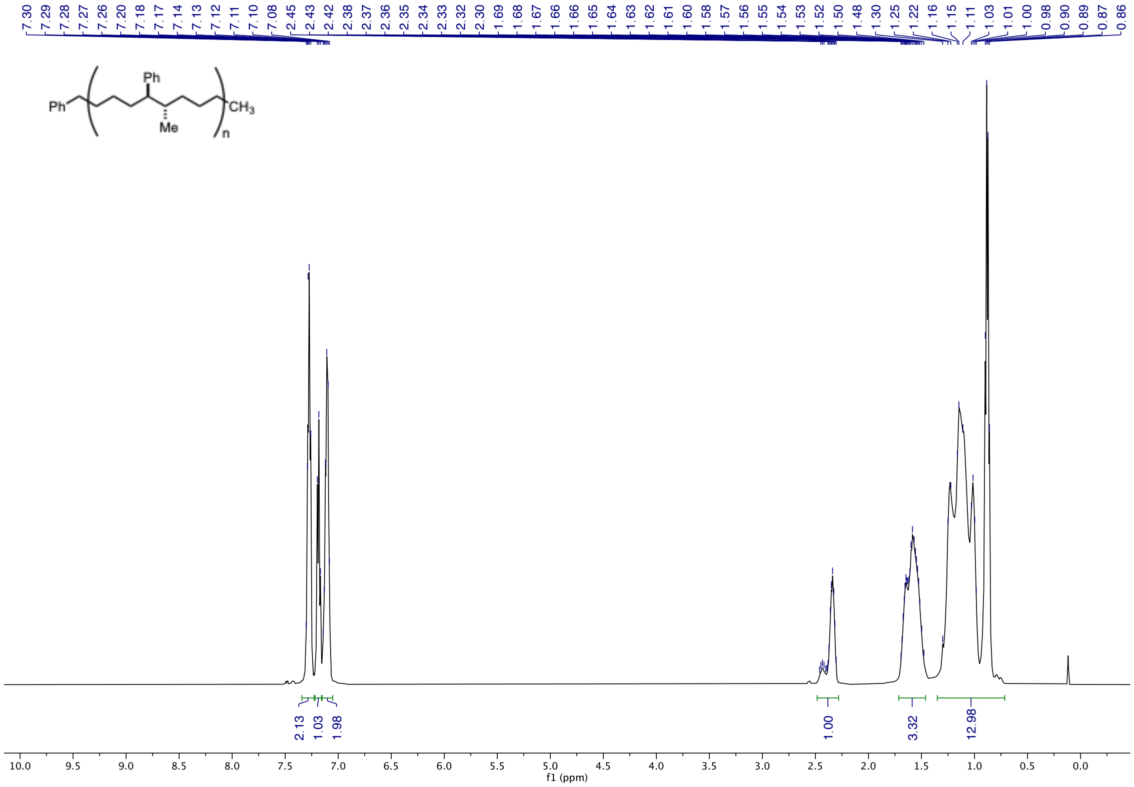

^{13}C NMR (126 MHz, $CDCl_3$) of **P4x-H_2**:

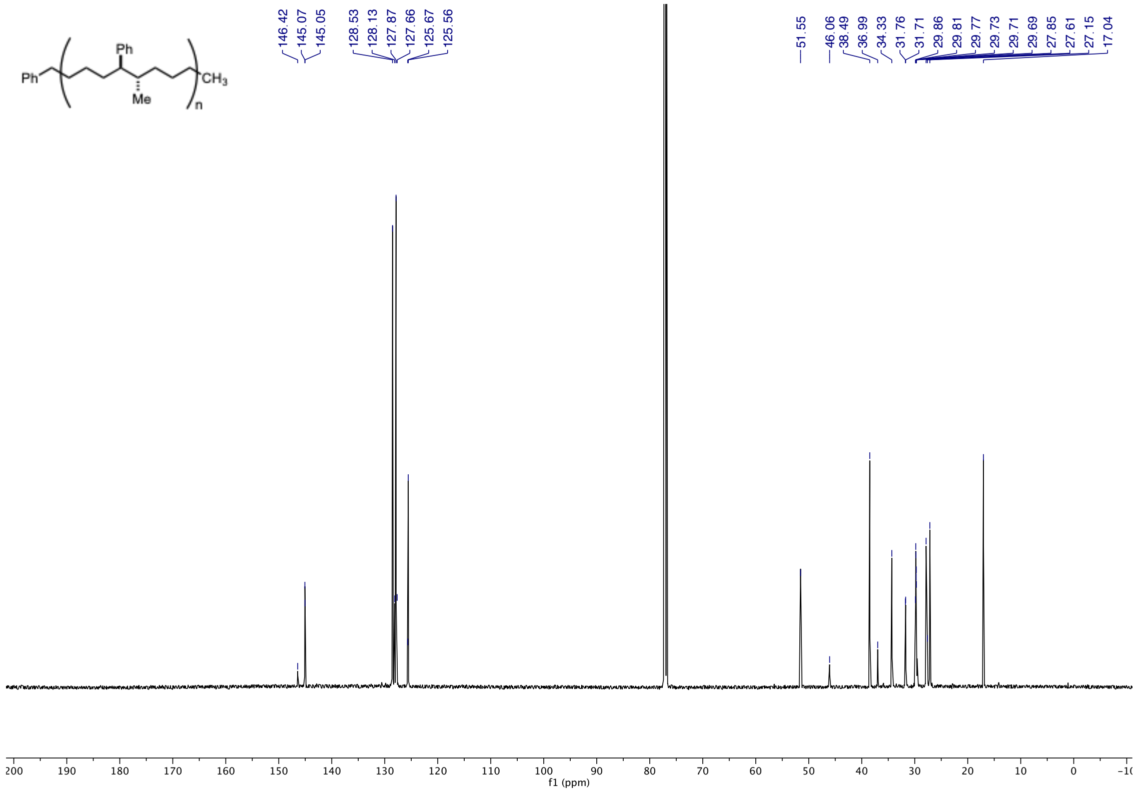

^{1}H NMR (500 MHz, CD_3OD) of **4s'-TFA**:

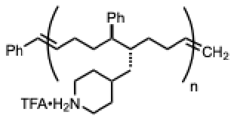

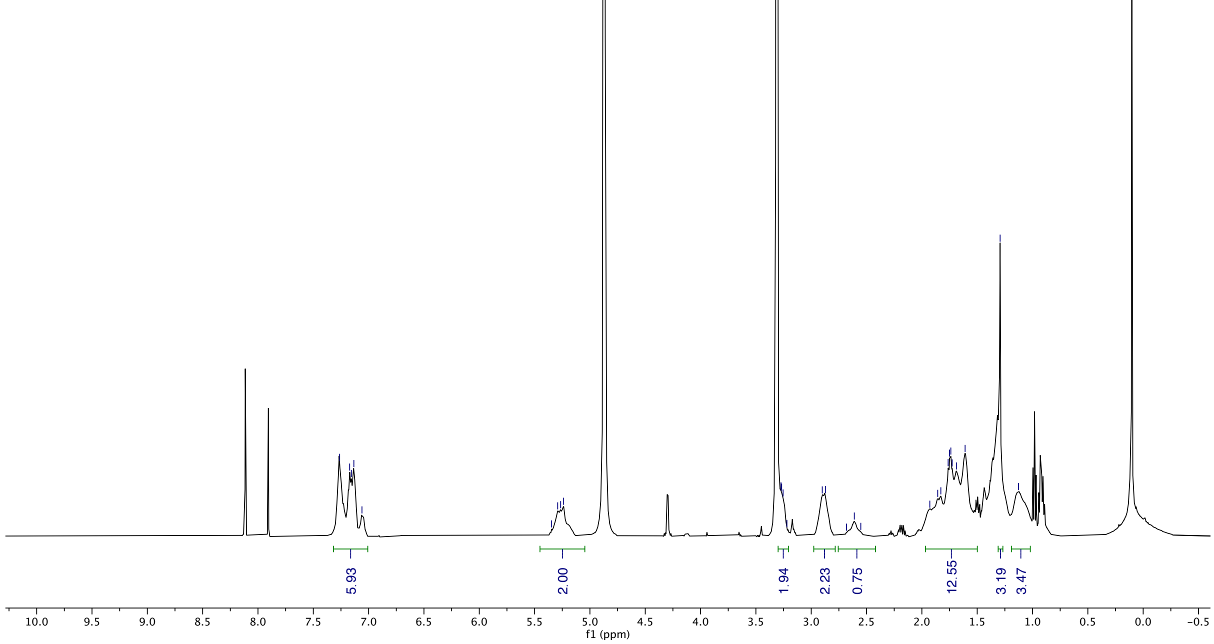

Supplement: Supplementary file 1 [file oc5c02173_si_001.pdf]
