# Supplementary material for: B‑Alkyl-borabicyclo[3.3.1]nonane Reagents Promote Closed-Shell Nickel-Catalyzed Alkylarylation Toward Encoded Cyclooctene Monomers
Source: ACS Cent Sci. 2026 Feb 20;12(3):324–32. doi: 10.1021/acscentsci.5c02173 (PMC13022722; doi:10.1021/acscentsci.5c02173)
Supplement: Supplementary file 3 [file oc5c02173_si_003.pdf]

oc-2025-021737.R1

Name: Peer Review Information for "Alkyl-9-BBN Reagents Promote Closed-Shell Nickel-Catalyzed Alkylarylation Towards Encoded Cyclooctene Monomers"

First Round of Reviewer Comments

Reviewer: 1

Comments to the Author

The manuscript by Engle and colleagues reports a closed-shell nickel-catalyzed three-component alkylarylation reaction of 1,5-cyclooctadiene. By employing alkyl-9-BBN reagents as effective alkyl nucleophiles in combination with aryl iodides, the authors achieve successful construction of 5,6-alkylarylated cyclooctenes (AACOEs). These products are suitable for ring-opening metathesis polymerization, leading to previously unexplored polymeric materials. This work builds upon the authors' earlier research on iterative diarylation of 1,5-cyclooctadiene (ref. 28), with an interesting advancement being the incorporation of C(sp3) fragments from B-alkyl-9-BBN reagents. The method exhibits broad functional group tolerance and accommodates alkyl chains of various lengths. Notably, DFT calculations on the nickel catalytic cycle reveal that destabilization of the ground state prior to transmetalation facilitates the observed reactivity of B-alkyl-9-BBN reagents, in contrast to the less efficient alkylboronic esters. The prospective utility of this methodology is further highlighted by successful product diversification and polymerization applications. Leveraging ring-opening metathesis polymerization of the AACOEs, the resulting polymers show properties that are significantly influenced by the size and chemical nature of the encoded side chains. Both the manuscript and the supporting information are meticulously prepared. Therefore, I recommend publication in ACS Central Science after the authors address the following minor points:

1) A key review on C(sp3)–C(sp3) cross-coupling using alkyl-9-BBN reagents should be cited: M. Sigman et al., Chem. Rev. 2011, 111, 1417–1492.

2) Given nickel's strong ability for oxidative addition, could the authors comment on why this reaction protocol does not work with aryl bromides?

3) Besides 1,5-cyclooctadiene, other olefins were screened in the three-component reaction (Table S4). Cyclooctene afforded the three-component product in 12% yield. Is the major byproduct in this case the two-component coupling product between the aryl iodide and B-alkyl-9-BBN? Moreover, could this three-component system be extended to other unactivated olefins by modifying conditions such as the ligand?

4) In the SI, the 13C NMR spectra for fluorine-containing compounds (products 4d, 4n, and 4n') are presented incorrectly: the carbon signals are split, and the corresponding coupling constants (J) should be provided.

Reviewer: 2

Comments to the Author

This work by Liu, Gutekunst, and Engle is a rigorous examination of the reaction development and optimization of a new set of cyclooctadiene-derived materials with aryl and alkyl side chains. A few issues should be addressed before publication.

- I am unconvinced if the authors framing of this method as a pathway to "encoded" monomers. While the authors suggest that these polymers could be thought of sequence-specific polymerizations of butadiene, alpha olefins, and styrene, the authors do not show that there is any regiocontrol over the ring opening of these monomers (distal substitution would not likely result in good control). If the authors are hoping to elevate this as a sequence polymerization, they may need to show that the better control over "monomer" order relative to a random copolymerization (of butadiene, alpha olefins, and styrene).

- Figure 1A shows a transition state for what seems to be the second aryl group addition. Labeling this more explicitly either in the figure or the caption would be helpful.

- Has functionalization of both alkenes been observed?

- Figure 2 can often be difficult to understand. After introducing the first condition, we then pivot to look at 2B, and then continue with looking at the other entries in the table, which itself can be difficult to read. Perhaps a better way to present these data would be to add columns for the different variables (temperature, [M] identity, Ph-X, etc), which would allow less to be crammed into the scheme above the table and a more linear presentation of the

data. By making a full page table, you can also list yield 4a (%) instead of having each entry have a %, making the table itself look cleaner.

- Is it necessary to include stirring rate in the schemes? This is not a convention I typically follow, distrust the idea that it is necessary unless this reaction is heterogeneous. If it is, in fact, this should be mentioned in the discussion.

- The potential energy diagrams in Figure 4 can be challenging to decipher. The authors could consider coloring their compound labels (8a, 8b, etc.) the same as their lines and potentially switch from red to another color as red is also the color of the Ph group.

- On page 5, line 55, the authors state "On the other hand, this also led to greater

control over the polymerization process." While they discuss at length the polymerization after this, the meaning of this line remains unclear. Control over polymerization often implies good MWD control, and the dispersities are high ~2.0.

Reviewer: 3

Comments to the Author

This manuscript presents a significant and innovative contribution to the fields of nickel catalysis, alkene difunctionalization, and polymer science. The authors describe a closed-shell, polar mechanism for the nickel-catalyzed alkylarylation of 1,5-cyclooctadiene (COD) using alkyl-9-BBN reagents, overcoming previous limitations associated with radical-based pathways. The work is well-executed, combining method development, mechanistic studies via DFT, and polymer applications. The manuscript is suitable for publication in ACS Central Science after addressing the following issues shown below.

1)In the report, 1,5 - cyclooctadiene (COD) was used as the feedstock with good efficiency. Did the author also try other cyclo - dienes as the feedstock in the difunctionalization and polymer application?

2)The Supporting Information mentions ligand screening without significant improvement. It might be useful to briefly summarize the key findings (e.g., why DMFU is optimal) in the main text?

3)The SEC data show dispersities (Đ) of 1.5–2.2, indicating some chain transfer or termination events. The author was suggested to notify the possible origins of this broadening, which would be informative.

4)It is recommended to cite relevant literature on nickel catalysis references (e,g. Nat. Synth. (2025), https://doi.org/10.1038/s44160-025-00840-5; Nat. Commun. 16, 7218 (2025); Angew. Chem. Int. Ed. 2021, 60, 21360–21367), as they provide valuable support for this article.

5)Could the authors comment on whether this side reaction is more prevalent among certain substrate classes and if any strategies were attempted to suppress it? (Given that the formation of the two - component side product is noted.)

6)There are still some typos, such as Figure 2A: The entry numbers in the table do not align perfectly with the described deviations (e.g., entry 3 refers to“without DMFU”but is listed as entry 4 in some descriptions); Page 5, Figure 4: The caption mentions “See SI for details,” but the label is cut off. Ensure all references to SI are correctly linked.

Author's Response to Peer Review Comments:

January 9, 2026

Keary M. Engle, Ph.D.
Professor, Department of Chemistry
John and Susan Diekman Dean of Graduate & Postdoctoral Studies

10550 North Torrey Pines Road
La Jolla, California 92037
Mail: BCC 169
Email: keary@scripps.edu
Tel: 858.784.7885

Dear Editor:

Thank you for your assistance with our manuscript (#oc-2025-021737). We have made the requested changes and are resubmitting the manuscript titled, “Alkyl-9-BBN Reagents Promote Closed-Shell Nickel-Catalyzed Alkylarylation Towards Encoded Cyclooctene Monomers” that was reviewed for publication as an Article for *ACS Central Science*. The review comments are included below along with our point-by-point responses (in blue color).

Reviewer 1:

The reviewer has raised 4 major points to address that are listed below. Our responses to their questions are provided below each point:

“1) A key review on C(sp3)–C(sp3) cross-coupling using alkyl-9-BBN reagents should be cited: M. Sigman et al., Chem. Rev. 2011, 111, 1417–1492.”

Our reply: We thank the reviewer for their evaluation of our work. We have included the review as a reference in the introduction to alkyl-9BBN in Suzuki–Miyaura cross-couplings (ref 38, page 2).

“2) Given nickel’s strong ability for oxidative addition, could the authors comment on why this reaction protocol does not work with aryl bromides?”

Our reply: We thank the reviewer for this question. Consumption of aryl bromide is observed (~50%) under the reaction conditions, which could indicate that oxidative addition is occurring. However, we do not observe desired three-component coupling product nor the side product from the two-component coupling. The use of aryl bromides and aryl chlorides in these three-component reactions is an interest and focus in our lab.

“3) Besides 1,5-cyclooctadiene, other olefins were screened in the three-component reaction (Table S4). Cyclooctene afforded the three-component product in 12% yield. Is the major byproduct in this case the two-component coupling product between the aryl iodide and B-alkyl-9-BBN?

Moreover, could this three-component system be extended to other unactivated olefins by modifying conditions such as the ligand?”

Our reply: We thank the reviewer for this question. Yes, the major side product in the reaction with cyclooctene is the two-component Suzuki–Miyaura coupling product. Additionally, a substantial amount of remaining aryl iodide is observed. In response, we have added a comment about the cyclooctene substrate in the SI Table S4: “[a] The main

products after end reaction is the two-component Suzuki– Miyaura coupling and remaining aryl iodide."

We appreciate the reviewer's suggestions to extend our work to other olefins. We are currently exploring alkyl-9-BBN in other systems with unactivated alkenes and are optimizing ligand scaffolds to enhance yield and selectivity. However, these findings are outside of the scope of this manuscript and will be published independently.

"4) In the SI, the ^{13}C NMR spectra for fluorine-containing compounds (products 4d, 4n, and 4n') are presented incorrectly: the carbon signals are split, and the corresponding coupling constants (J) should be provided."

Our reply: We appreciate the feedback and meticulous evaluation. We have rerun the ^{13}C NMR of **4d**, **4n**, and **4n'** with more scans and on a higher magnetic field instrument. With these new ^{13}C NMR spectra, we were able to assign the splitting of **4d**, and some of the splitting patterns in **4n** and **4n'**, although some regions are assigned as multiplets. Additionally, we have clarified the coupling constants in both ^{13}C and ^{19}F NMRs ($J_{C–F}$ *and* $J_{F–F}$ respectively). We have updated the characterization data and ^{13}C NMR spectra for all three products in the SI.

Reviewer 2:

The reviewer listed their comments and questions in bullet points. We have addressed their feedback below each point:

"- I am unconvinced if the authors framing of this method as a pathway to "encoded" monomers. While the authors suggest that these polymers could be thought of sequence-specific polymerizations of butadiene, alpha olefins, and styrene, the authors do not show that there is any regiocontrol over the ring opening of these monomers (distal substitution would not likely result in good control). If the authors are hoping to elevate this as a sequence polymerization, they may need to show that the better control over "monomer" order relative to a random copolymerization (of butadiene, alpha olefins, and styrene)." Our reply: We thank the reviewer for their feedback. We agree that these materials lack regiocontrol in the ring-opening polymerization, but this "encoded" monomer strategy enables complete compositional control over the terpolymer with a perfect 1:1:1 ratio of butadiene, α-olefins and styrene monomers. This combination of monomers would be challenging to directly copolymerize through traditional means while maintaining a regular distribution along the polymer chain. The ability to polymerize these monomers regioselectively is an area of future research in our laboratories. To avoid potential confusion by the reader with respect to the regioregularity of the synthesized polymers, the text has been updated as follows: "These

materials represent encoded terpolymers derived from styrene, α-olefin, and butadiene monomers. The terpolymers lack long-range regioregularity with respect to the styrene and α-olefin subunits but maintain a perfectly stoichiometric composition and regular distribution of subunits that would be difficult to obtain through direct polymerization.[55]" (page 5) Reference on direct terpolymerization:

(55) Kaita, S.; Takano, S.; Tardif, O.; Kamigaito, M.; Kanomi, S.; Jinnai, H. Advanced Polyolefins: Ethylene, Styrene, and Butadiene Terpolymers with Toughness, Self-Healing, and Enhanced Adhesion via Bifunctional Dual-Site Polymerization. *ChemRxiv* **2025**. DOI:10.26434/chemrxiv-2025-4jx6j. This content is a preprint and has not been peer-reviewed

Additionally, we have changed the bullet point in Figure 1C to: "*compositionally defined terpolymer*"

"- Figure 1A shows a transition state for what seems to be the second aryl group addition. Labeling this more explicitly either in the figure or the caption would be helpful."
Our reply: We appreciate the reviewer's feedback and have edited the caption for Figure 1: "Background and synopsis of work. **A.** The diarylation protocol of COD using transannular directivity to control the selectivity. **B.** Literature precedent on alkylation of alkenes. **C.** Our work presenting a closed-shell nickel catalyzed alkylarylation method."

"- Has functionalization of both alkenes been observed?"
Our reply: We thank the reviewer for their question. We have not observed functionalization of both alkenes. This result is similar to our previous findings in the diarylation of 1,5-COD (ref 28). We have added a note about this in the text "Notably, tetrafunctionalization of **2a** is not observed." (page 2)

"- Figure 2 can often be difficult to understand. After introducing the first condition, we then pivot to look at 2B, and then continue with looking at the other entries in the table, which itself can be difficult to read. Perhaps a better way to present these data would be to add columns for the different variables (temperature, [M] identity, Ph-X, etc), which would allow less to be crammed into the scheme above the table and a more linear presentation of the data. By making a full page table, you can also list yield 4a (%) instead of having each entry have a %, making the table itself look cleaner." Our reply: We appreciate the feedback and have edited Figure 2 accordingly.

"- Is it necessary to include stirring rate in the schemes? This is not a convention I typically follow, distrust the idea that it is necessary unless this reaction is heterogeneous. If it is, in fact, this should be mentioned in the discussion."

Our reply: We appreciate the reviewer's comments. During the optimization of the reaction conditions, we observed slightly higher yields and more consistent reaction outcomes at a high stirring rate. In previous work for the diarylation of 1,5-COD, a thorough analysis of the reaction kinetics was performed which showed that the reaction was heterogeneous. However, for this work we did not investigate the kinetics. We have added a comment in the discussion on optimization conditions: "The reaction yield was optimal at 35 °C and a stir rate above 1300 rpm pointing to the heterogeneous nature of the reaction and the importance of mass transfer effects." (page 2). Additionally, we have added an entry in the optimization table with a 500 rpm stir rate to show the decrease in yield (Figure 2B, entry 5).

"- The potential energy diagrams in Figure 4 can be challenging to decipher. The authors could consider coloring their compound labels (8a, 8b, etc.) the same as their lines and potentially switch from red to another color as red is also the color of the Ph group."

Our reply: We thank the reviewer for their suggestion and have edited the colors in Figure 4.

"- On page 5, line 55, the authors state "On the other hand, this also led to greater control over the polymerization process." While they discuss at length the polymerization after this, the meaning of this line remains unclear. Control over polymerization often implies good MWD control, and the dispersities are high ~2.0."

Our reply: We appreciate the reviewer's feedback. The comment on improved control over the polymerization process was in reference to the previously published DACOE monomers, but we agree the specific improvement achieved was not clear. The sentence has been adjusted to clarify the ability to target molecular weights with monomer-to-initiator ratios, which historically has been achieved through chaintransfer agents with cyclooctene derivatives. "On the other hand, this also led to greater control over the polymerization process in which polymer molecular weight could be readily targeted by varying the monomer to initiator ratio." (page 5).

Reviewer 3:

The reviewer summarized their feedback in a numbered list. We have provided a reply below each of their questions/comments:

"1) In the report, 1,5 - cyclooctadiene (COD) was used as the feedstock with good efficiency. Did the author also try other cyclo - dienes as the feedstock in the difunctionalization and polymer

application?" Our reply: We thank the reviewer for their question. We tried a range of different cyclic dienes, however, all gave <15% yield in the reaction. Furthermore, isomerization of the alkene is observed in most of these substrates giving a complex mixture of products and side products. We have included the data in the SI Table S4.

"*2) The Supporting Information mentions ligand screening without significant improvement. It might be useful to briefly summarize the key findings (e.g., why DMFU is optimal) in the main text?"*

Our reply: We have included a brief description in the text: "Generally, fumarate-derived ligands with similar steric and electronic profile to DMFU performed comparably, whereas no reactivity was observed with other ligand classes." (page 2)

"3) The SEC data show dispersities (Đ) of 1.5–2.2, indicating some chain transfer or termination events.

The author was suggested to notify the possible origins of this broadening, which would be informative." Our reply: The broadening of the molecular weight dispersity is believed to derive from chain transfer reaction, rather than termination reactions, given the monomodal nature of the elution peaks and the relatively unhindered double bonds in the polymer backbone that would be susceptible to secondary metathesis. The text reads as follows: "The obtained polymers exhibited dispersities (*Đ*) ranging from 1.5–2.2 and monomodal elution peaks by SEC, consistent with chain transfer reactions occurring on the unhindered backbone alkenes during polymerization." (page 6)

"4) It is recommended to cite relevant literature on nickel catalysis references (e,g. Nat. Synth. (2025), https://doi.org/10.1038/s44160-025-00840-5; Nat. Commun. 16, 7218 (2025); Angew. Chem. Int. Ed. 2021, 60, 21360–21367), as they provide valuable support for this article."

Our reply: We appreciate the reviewer's suggestion and have included these references in the introduction (Ref 34).

"5) Could the authors comment on whether this side reaction is more prevalent among certain substrate classes and if any strategies were attempted to suppress it? (Given that the formation of the two - component side product is noted.)"

Our reply: We thank the reviewer for the question. The two-component coupling is more prevalent in reactions with low yielding substrates, such as electron-deficient aryl iodides. We have added a sentence in the manuscript: "Full conversion of aryl iodide is observed regardless of the product yield with the twocomponent coupling being the major side product."

We have attempted to suppress this side product by ligand optimization which did not improve the yield. Additionally, we increased the amount of COD from 3 equiv to 5 equiv which improved the selectivity for desired product formation. We did not observe noticeable improvement in yield above 5 equiv of COD.

"6) There are still some typos, such as Figure 2A: The entry numbers in the table do not align perfectly with the described deviations (e.g., entry 3 refers to "without DMFU" but is listed as entry 4 in some descriptions); Page 5, Figure 4: The caption mentions "See SI for details," but the label is cut off. Ensure all references to SI are correctly linked."

Our reply: We thank the reviewer for making us aware of these typos. We have corrected them and other typos that we found in the manuscript.

We thank the reviewers for their instructive comments and thoughtful questions. As you will see from the text above, we have done our best to address their concerns. Thank you for your consideration of this manuscript.

Sincerely,

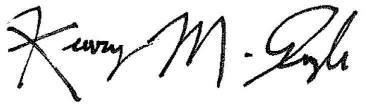

Keary M. Engle, Ph.D.
Professor, Department of Chemistry
John and Susan Diekman Dean of Graduate and Postdoctoral
Studies The Scripps Research Institute

oc-2025-021737.R2

Name: Peer Review Information for "Alkyl-9-BBN Reagents Promote Closed-Shell Nickel-Catalyzed Alkylarylation Towards Encoded Cyclooctene Monomers"

Second Round of Reviewer Comments

Reviewer: 1

Comments to the Author

In this revision, Liu, Gutekunst, Engle, and coauthors provided a series of convincing studies to support the reaction and related mechanism. They addressed all the previous concerns properly. I recommend the publication of this manuscript as it is.

A minor point in SI: It is recommended that the authors specify the type of NMR spectrum (1H, 13C, or 19F), compound number, and deuterated solvent for all data in the NMR spectra section.

Reviewer: 2

Comments to the Author

The authors did an excellent job incorporating the proposed revisions from the reviewers. The language added to the text is clarifying, and the changes to the figures and table make the work much easier to follow. The manuscript is suitable for publication in ACS Central Science.

Reviewer: 3

Comments to the Author

Engle and co-workers have tried best to fulfill the referees' suggestions on this manuscript. Therefore, I recommend its acceptance for publication in ACS Central Science.

Author's Response to Peer Review Comments:

Dear Editorial Office,

(1) We have added the email addressees of the corresponding authors on the first page of the manuscript, as requested.

(2) Panels within figures have been separately captioned, as requested.

(3) Captions have been added to the NMR spectra, as requested.

Thank you for your assistance with this manuscript.

Sincerely,

Keary Engle
